# Enhancing Human Spermine Synthase Activity by Engineered Mutations

**DOI:** 10.1371/journal.pcbi.1002924

**Published:** 2013-02-28

**Authors:** Zhe Zhang, Yueli Zheng, Margo Petukh, Anthony Pegg, Yoshihiko Ikeguchi, Emil Alexov

**Affiliations:** 1 Computational Biophysics and Bioinformatics, Department of Physics, Clemson University, Clemson, South Carolina, United States of America; 2 Department of Cellular and Molecular Physiology, Milton S. Hershey Medical Center, Pennsylvania State University College of Medicine, Hershey, Pennsylvania, United States of America; 3 Laboratory of Bio-analytical Chemistry, Department of Pharmaceutical Sciences, Faculty of Pharmaceutical Sciences, Josai University, Saitama, Japan; UNC Charlotte, United States of America

## Abstract

Spermine synthase (SMS) is an enzyme which function is to convert spermidine into spermine. It was shown that gene defects resulting in amino acid changes of the wild type SMS cause Snyder-Robinson syndrome, which is a mild-to-moderate mental disability associated with osteoporosis, facial asymmetry, thin habitus, hypotonia, and a nonspecific movement disorder. These disease-causing missense mutations were demonstrated, both in silico and in vitro, to affect the wild type function of SMS by either destabilizing the SMS dimer/monomer or directly affecting the hydrogen bond network of the active site of SMS. In contrast to these studies, here we report an artificial engineering of a more efficient SMS variant by transferring sequence information from another organism. It is confirmed experimentally that the variant, bearing four amino acid substitutions, is catalytically more active than the wild type. The increased functionality is attributed to enhanced monomer stability, lowering the pKa of proton donor catalytic residue, optimized spatial distribution of the electrostatic potential around the SMS with respect to substrates, and increase of the frequency of mechanical vibration of the clefts presumed to be the gates toward the active sites. The study demonstrates that wild type SMS is not particularly evolutionarily optimized with respect to the reaction spermidine → spermine. Having in mind that currently there are no variations (non-synonymous single nucleotide polymorphism, nsSNP) detected in healthy individuals, it can be speculated that the human SMS function is precisely tuned toward its wild type and any deviation is unwanted and disease-causing.

## Author Summary

Proteins are constantly subjected to evolutionary pressure to assure the organism's survival and reproduction. At the same time, the proteins' amino acid sequence undergoes mutations, some of which may cause diseases while others may be reflecting natural differences within the population (non-synonymous single nucleotide polymorphism, nsSNP). In this study we examine the human spermine synthase (HsSMS), for which currently there are no nsSNPs, while rare disease mutations are known to cause Snyder-Robinson syndrome. What is so special with this protein? Maybe the HsSMS is so well optimized for its function that any change of the wild type sequence should be degrading its performance. To check such a possibility, we engineered a mutant of HsSMS with enhanced stability, electrostatic and mechanical properties. The mutant was confirmed experimentally to be a better enzyme than the wild type. Thus, the HsSMS is not evolutionally optimized with respect to its enzymatic reaction, its amino acid sequence differs only in sick individuals and so far its sequence was found to be identical in all healthy individuals. Therefore, it can be speculated that the HsSMS function is precisely tuned toward the wild type characteristics such so any deviation is unwanted and is disease-causing.

## Introduction

Polyamines are widely present in many organisms including mammals, plants as well as some bacteria, and play important roles in normal cell growth, differentiation, programmed death, and tissue repair [1], [2]. A particular polyamine of interest in this manuscript, the spermine (SPM), was shown to be synthesized by the aminopropyltransferase enzyme - spermine synthase (SMS) through conversion of spermidine (SPD) to SPM [3]. Further structural and biochemical studies have revealed that SMS exists as a dimer and that the dimerization is crucial for its normal function [2], [4]. Structurally each monomer of SMS contains three domains: an N-terminal domain to form the dimer contacts, a central domain serving as a lid for the C-terminal domain, and a C-terminal catalytic domain [2], [4]. In wild type SMS, the amine substrate is coordinated via interactions between its terminal nitrogen atoms and acidic residues in the protein and by hydrophobic interactions of SMS with the alkane portions of the polyamine [2].

The importance of SMS for normal cell function is manifested by the fact that a malfunctioning of SMS results in the deficiency of SPM and affects the normal development in mice, humans and other organisms. In humans, this deficiency results in the Snyder-Robinson syndrome (SRS), which is a disease caused by the X-linked SMS gene defects. Patients with SMS show mild-to-moderate mental retardation, osteoporosis, facial asymmetry, thin habitus, hypotonia, and a nonspecific movement disorder [5]. The malfunction of SMS in patients with SRS was attributed to several missense mutations as c.267G>A (p.G56S) [6], c.496T>G (p.V132G) [7], c.550T>C (p.I150T) [8]–[10] and c.1084A>G (p.Y328C) (Zhang et al., in preparation). Computational modeling has revealed that these missense mutations affect SMS stability, flexibility, interactions, and formation of the dimer structures [9]–[12].

As mentioned above, the spermidine synthase (SRM) is widespread from E. coli, mammals, and plants to yeast. It is an aminopropyltransferase with a very high degree of specificity for putrescine as the amine acceptor and synthases SPD [13]. It was shown that the SRM of Thermotoga maritima, which is the only bacterium known to grow at a high temperature as well as 90°C [14], is remarkably stable to thermal denaturation particularly in the presence of the amine acceptor substrate putrescine with a half-life larger than 25 h at 90°C. In contrast, the human SRM (HsSRM) and SMS (HsSMS) are less stable under the same temperature [4], [15]. The goal of this study is to probe the effect of transferring sequence information from Thermotoga maritima SRM (TmSRM) and SMS (TmSMS) to HsSMS and to reveal the effect of the amino acid substitutions on its stability and function. Previous *in silico* studies have indicated that mutations of the wild type amino acids may not be degrading the native properties (monomer stability, dimer affinity, hydrogen bond network and ionized states) of HsSMS even if they are introduced at sites known to harbor disease-causing mutations [10]. Here the mutation sites are chosen not to coincide with disease-causing mutation sites and the amino acid substitutions are aimed at enhancing wild type activity of HsSMS.

## Results

### I) Computational results

#### a) Results of MSA

**Figure 1:**
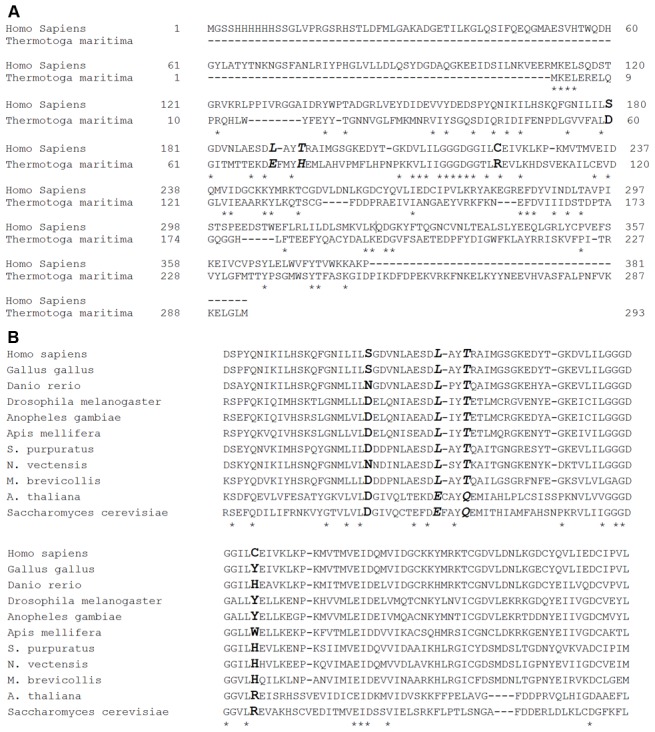
Multiple sequence alignment (MSA). (A) MSA between HsSMS and TmSRM. Conserved residues are indicated by star “*”, and the four mutations are represented in bold italic letters; (B) MSA among different SMS species. The star “*” indicates the conserved residue among different species, and the residues corresponding to the mutation sites are marked in bold italic letters.

The sequences of HsSMS and TmSRM were aligned at the starting point of the investigation. The alignment is shown in Figure 1A, where conserved residues are marked by star “*” and the bold italic letters indicate the four mutation sites. The query coverage is 50% and E value is 5e-13 given by Protein-BLAST [16]. As seen below, the sequences of HsSMS and TmSRM share many similarities but are also significantly different at many sites. The alignment (Figure 1A) was mapped onto HsSMS 3D structure and all sites being either invariant (with respect to amino acid substitution) or buried were removed from the candidate list. The remaining candidates, which are surface exposed residues in HsSMS (Table S1), were subjected to additional considerations, as described in supplementary material, reducing the candidate sites to twenty five. The next step was to utilize multiple sequence alignment (MSA) from different species and from homologous sequences to further reduce the list of candidates. The MSA among different species is shown in Figure 1B. The whole comparison is quite long (Supplementary materials Table S2), thus we only list the “four mutations” section in the figure. MSA of all homologous to HsSMS protein is shown in supplementary materials Table S3. The frequency of residue appearance for each of the four mutations sites is shown in Table 1.

**Table 1:** Frequency and percentage of residue appearance at the mutation sites among 500 homologous proteins.

Mutation Site	Residues and Appearance					
**S165D**	Residues	Asp	Ser	Asn	Tyr	Gaps
	Freq. (%)	428 (85.6%)	57 (11.4%)	10 (2.0%)	1 (0.2%)	4 (0.8%)
**L175E**	Residues	Glu	Leu	Thr	Ile	Gaps
	Freq. (%)	380 (76.0%)	103 (20.6%)	4 (0.8%)	4 (0.8%)	2 (0.4%)
**T178H**	Residues	Gln	His	Thr	Asp	Gaps
	Freq. (%)	203 (40.6%)	167 (33.4%)	114 (22.8%)	10 (2.0%)	2 (0.4%)
**C206R**	Residues	Arg	Cys	Tyr	Trp	Gaps
	Freq. (%)	386 (77.2%)	45 (9.0%)	38 (7.6%)	11 (2.2%)	1 (0.2%)

Further refinement of the candidate list was based on the MSA (Figure 1B and Table S2). Sites located next to the conserved site were given preference with respect to isolated non-conserved sites (Table S2). This resulted in eight residues: N149, N160, S165, L175, C206, Q223, M224, and C347.

Additionally N149 is just next to a disease-causing mutation site I150 [9], [10] and quite close to the active site. Because of that, it is considered that its mutation could abolish the function of HsSMS and this candidate was removed from the list.

Frequency and percentage of residue appearance at the mutation sites among 500 homologous proteins suggested the final selection: if the TmSRM→HsSMS substitution is found to appear less than 50% in the MSA, the candidate was deleted. Thus, N160, M224 and C347 were deleted because N160→V, M224→L and C347→F substitutions were found in less than 50% of cases. The site Q223 is gap (Table S3) and was deleted as well.

As result, only three sites were kept in the candidate list, namely S165→D, L175→E and C206→R to be mutated to the corresponding residues in TmSRM. In addition, the site T178 was included in the list because it is correlated with L175. The corresponding mutation is T178→H. Finally four mutations were selected (S165D, L175E, T178H and C206R) and for the purpose of this investigation, several tags are introduced, i.e. SDmut for S165D; CRmut for C206R; Pmut for the double mutant “L175E and T178H”; and Fmut for the 4a.a. mutant “S165D, L175E, T178H and C206R”.

#### b) Effect on ionized states due to the four mutations

pKa calculations indicated only few titratable groups are affected by the mutations (Table 2). Particularly, the active site Asp201 is predicted to experience a large pKa shift due to the “pair” mutations (L175E and T178H). This shift was predicted for both Pmut (L175E and T178H) and Fmut (S165D, L175E, T178H and C206R) structures. No effect was calculated upon S165D and C206R substitutions and because of that only results obtained Pmut are shown.

**Table 2:** pKa value of Glu175, His178 and Asp201 in C monomer of Pmut.

Residue	WT	Pmut (L175E & T178H)
**Glu175**	N/A	2.926
**His178**	N/A	3.573
**Asp201**	0	14

The mutation at position 175 results in fully ionized Glu residue, simply because E175 side chain is fully solvent accessible. In contrast, the side chain of H178 is fully buried inside the protein, thus it is predicted to be deprotonated. Asp201 is fully ionized in the WT structure which is exactly the same as suggested in our previous work [9], but it is fully protonated in the mutant structure. Detailed analysis addressing structural reasons for these pKa shifts will be presented in the Discussion.

#### c) Effect on monomer stability due to the four mutations

In the following three sections we reported the computational results with respect to effect of mutations on monomer stability, dimer affinity, change of the electrostatic potential distribution.

Table 3 shows the calculated results for folding energy changes due to the mutations. The negative folding energy change indicates that the mutation results in a less stable monomer, while positive change indicates that the mutant is more stable than the WT.

**Table 3:** The results of structure – based (3C6K) folding energy calculation on monomer stability changes under three force fields.

Mutants ID	Monomer ID	AMBER98 (kcal/mol)	CHARMM27 (kcal/mol)	OPLSaa (kcal/mol)	Ave three force fields (kcal/mol)	Ave C & D (kcal/mol)	Adjustment according to sMMGB [53] * (kcal/mol)
**SDmut (S165D)**	C	51.07	109.92	90.89	83.96	83.28	6.66
	D	42.60	107.56	97.66	82.60		
**CRmut (C206R)**	C	194.73	276.20	56.20	175.71	181.32	15.77
	D	203.56	276.14	81.06	186.92		
**Pmut (L175E/T178H)**	C	3.48	106.25	139.76	83.16	90.05	7.29
	D	9.07	114.50	167.26	96.94		
**Sum**	C	249.28	492.37	286.85	342.83	354.65	31.89
	D	255.23	498.20	345.98	366.46		
**Fmut (S165D/L175E/T178/C206R)**	C	249.59	508.35	292.48	350.14	355.42	31.97
	D	274.07	493.99	314.02	360.69		

* Two parameters were used to adjust the prediction according to the experimental data. For details of sMMGB refer to [53].

The energy calculations predict that all mutants are much more stable than WT, especially the Fmut which is estimated to stabilize monomer structure by more than 30 kcal/mol. The last number is clearly an overestimation and should not be assumed to be confirmed experimentally, but rather should be considered as a tendency. In addition, all three force fields gave the same trend of change, i.e. increasing the monomer stability upon the mutations. It should be pointed out that the calculations with C monomer and D monomer always gave quite close results, demonstrating the robustness of the computational protocol.

Table 3 provides a row termed “Sum”, which shows the sum of predicted energy changes taken from individual mutants (note that that the pair 175–178 is taken together). The motivation of carrying calculations for “sum” is to test the additivity. Thus, one can see from Table 3 that the sum of individual energy change is almost the same as the combined effect of four mutations (last row in Table 3). Thus the effect of these mutations in current research is additive indicating that sites 165, 206 and 175 plus 178 do not interact with each other.

Furthermore the webserver Eris was also used for the prediction of folding energy change. Since Eris has a difficulty in modeling the disulfide bond, thus Cys related mutations are not supported. Therefore only SDmut and Pmut were submitted. The results are shown in Table 4. With the protocol of Eris, this result implied that SDmut and Pmut have more stable structure, which agreed with the prediction by sMMGB method.

**Table 4:** The prediction of folding free energy change due to the mutations by Eris.

Mutants ID	Monomer ID	ΔΔG (kcal/mol)	Ave. C & D (kcal/mol)
**SDmut (S165D)**	C	1.44	1.15
	D	0.85	
**Pmut (L175E/T178H)**	C	0.51	0.84
	D	1.16	

#### d) Effect on dimer affinity due to the four mutations

Dimer formation was shown to be crucial for the function of HsSMS. Therefore we carried analysis of the effects of mutations on dimer affinity. Results are shown in Table 5. Positive values indicate that the mutation increases the dimer affinity while the negative values indicate that the mutant has a less stable dimer formation.

**Table 5:** The results of structure – based (3C6K) binding energy calculation on dimer affinity changes under three force fields.

Mutants ID	AMBER98 (kcal/mol)	CHARMM27 (kcal/mol)	OPLSaa (kcal/mol)	Ave three force fields (kcal/mol)
**SDmut (S165D)**	−1.71	−1.61	−0.22	−1.18
**CRmut (C206R)**	3.79	0.78	1.41	1.99
**Pmut (L175E/T178H)**	0.50	−1.47	−1.20	−0.73
**Sum**	2.58	−2.3	−0.01	0.08
**Fmut (S165D/L175E/T178H/C206R)**	0.36	−2.11	−0.07	−0.61

It can be seen that all of the predicted binding energy changes listed in the table are very small. According to our previous work [10], such small binding energy change is considered not to have effect on dimer affinity. Such a conclusion is expected from structural perspective, since all four mutation sites are located within the C-domain and quite far away from the dimer interface. Note that the original experimental work has shown that the N-terminal domain is the cause of dimerization [4].

#### e) Effect on potential distribution due to the four mutations

**Figure 2:**
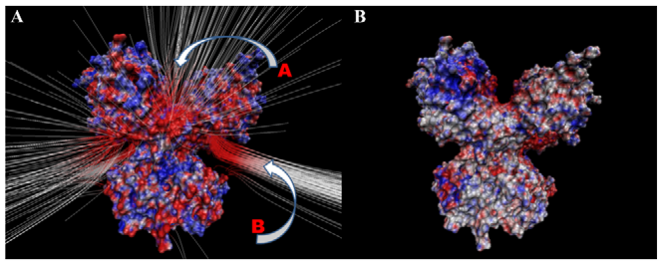
Potential distribution. (A) Electrostatic field lines for the WT HsSMS; Drawing method: FieldLines; GardientMag: 1.45; Min Length: 35.31; Max Length: 50.90; Coloring Scale Data Range: −1; 1; (B) Potential difference (mutant – WT) mapped onto HsSMS surface; Coloring method: Volume; 1. Drawing method: Surf; Coloring Scale Data Range: −1; 1 (blue – positive, red – negative).

One plausible reason why HsSMS functions as a dimer could be that the dimerization is necessary to provide guidance and steer the positively charged SPD/SPM in and out the active site. It can be speculated that there are two paths shown in Figure 2A as path “A” and “B”. The electrostatic field lines form a kind of funnel leading to the vicinity of the active sites. The path “A” is in the cleft between HsSMS domains and is a single path. In contrast, the path “B” is perpendicular to the dimer axis and is symmetrical (doubled) with an angle of symmetry 180° (Figure 2A). Neither of these paths, “A” or “B”, exist if one uses in the modeling the HsSMS monomer only, i.e. they are product of the dimerization. The mutations further increase the magnitude of the electrostatic potential along the both paths as it is illustrated in Figure 2B, where the change of the potential due to the mutations is shown. The potential difference is mapped onto molecular surface of the dimer. Red color patch indicates that the potential is more negative in the dimer as compared with the WT, and blue color the opposite. It can be seen that patches corresponding to both paths, “A” and “B”, are more negative in the mutant than in the WT, providing support of the hypothesis that the mutant enhanced activity is caused by better steering of the substrates to the active site.

#### f) Results of normal mode analysis

The ANM webserver calculates 36 smallest eigenvalues and each of them corresponded to a vibration mode. Out of them, the first 20 non-zero eigenvalues correspond to the 20 vibrational modes and are visualized in Jmol at the ANM site. Considering the two plausible pathways of SPD/SPM and inspecting the corresponding motions, it was found that the first three modes correspond to relevant domain motions (See supplementary material Figure S1). The results for these normal modes calculated with different force field minimized structures are shown in Table 6. From the table, one can see that Fmut has a slightly higher frequency of domain motion than the WT. The change of the domain vibrational frequency is expected to affect the reaction by modulating the approach time of the substrates as they travel along either of the electrostatic funnels.

**Table 6:** The results of normal mode analysis.

Structures	AMBER98 (GHz)	CHARMM27 (GHz)	OPLSaa (GHz)	Ave three force fields (GHz)
**Modes**	Mode1/2/3	Mode1/2/3	Mode1/2/3	Mode1/2/3
**WT**	29.21/34.77/39.55	24.93/27.26/32.77	28.98/34.05/37.96	27.71/32.02/36.76
**Fmut (S165D/L175E/T178H/C206R)**	30.00/35.56/40.76	24.38/27.34/32.71	29.26/33.66/37.59	27.88/32.19/37.02

### II) Experimental results

The HsSMS is highly specific for SPD as an amine acceptor. Kinetic analysis of the HsSMS reaction indicated that the Km values for SPD and dcAdoMet are 0.8 mM and 0.45 µM, respectively, and that the activity is 3776±494 nmol/h/mg (Table 7). On the other hand, the activity of four mutants HsSMS (Fmut) is over ten times higher than that of WT, whereas Km for both substrate of dcAdoMet and SPD are much less affected by the mutations. Still the decrease of the K_m_ for SPD indicates that the mutant affinity toward SPD increases in HsSMS (Fmut), while the affinity toward dcAdoMet decreases. The dramatic increase of the activity, therefore, cannot be attributed to the change of the affinity of mutant HsSMS to the reactants. Rather it should be associated with a change of the maximum rate (V_m_), which in turn may indicate better ability of reactants to find their way to the active site and to bind there.

**Table 7:** Comparison of SMS activity between WT and Fmut.

Protein	Activity (nmol/h/mg)	Km for dcAdoMet (µM)	Km for SPD (mM)
**WT**	3776±494	0.5	0.8
**Fmut (S165D/L175E/T178H/C206R)**	40418±3247	0.9	0.6

## Discussion

**Figure 3:**
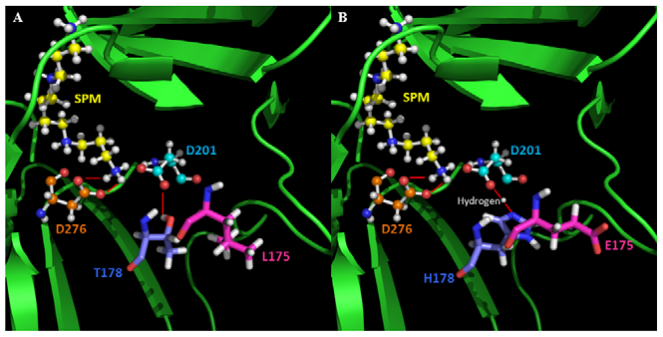
Interaction networks among product SPM, active sites: D201 & D276, and pair mutation sites. (A) WT; (B) Pmut. Pair mutation sites were shown with sticks: magenta represented L175 in WT and E175 in mutant; blue represents T178 in WT and H178 in mutant. The active sites (D201 and D276) and SPM are shown in ball and sticks: yellow represents SPM; orange represents D276 and cyan represents D201. The short red lines indicate hydrogen bonds.

The experimental data shows that the designed HsSMS mutant is more active than the WT HsSMS and computational investigations indicate that biophysical characteristics of the mutant are altered as compared with the WT characteristics. What are the structural origins for the predicted changes? The MSA among homologous proteins implied that the sites 175 and 178 are correlated. In the WT HsSMS, L175 and T178 are quite close, and the polar hydrogen of T178 makes a hydrogen bond (hydrogen bond length 1.81 Å) with OD2 of Asp 201 (Figure 3A), and thus providing support for the ionized form of Asp 201. The substitution with a charged residue Glu at site 175 introduces extra negative charge, but the side chain of E175 is almost fully exposed to the water phase and thus is solvated. However, the substitution causes slight backbone reengagement and at the same time, the negative potential of E175 suppresses the ionized form of D201 and makes D201 protonated. There is no hydrogen acceptor in the vicinity of D201 and Thr at position 178 is replaced with hydrogen donor/acceptor with a longer side chain, His, to make a hydrogen bond with the polar hydrogen of D201 (Figure 3B). The negative charge of E175 also supports the tautameric orientation of the side chain of His at position 178 by orienting the polar proton of H178 toward the negatively charged OE atoms of E175 (Figure 3B). It can be speculated that by favoring the protonated form of D201, the mutations weaken the interactions between the product, the spermine, and the protein moiety and thus facilitates the release of the product (note that the product, the spermine, has one extra positive charge as compared with the reactant, the spermidine). In addition, the protonation of D201 lowers a bit the pKa value of D276 (from pKa(WT) = 9.7 to pKa(Fmut) = 9.4) and thus reduces the work need to be done to protonate D276 upon substrate binding. It can be speculated that it will enhance the turnover of the reaction and will increase the reaction rate.

**Figure 4:**
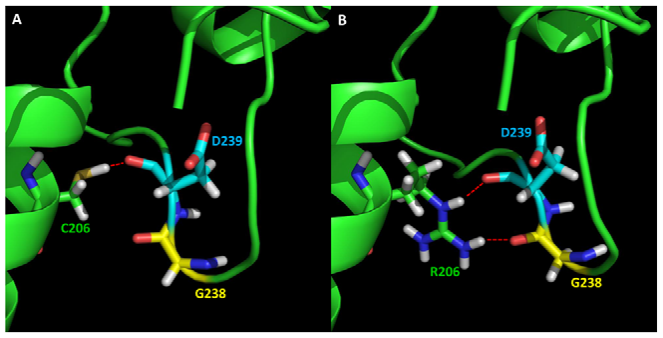
Effects on hydrogen networks surrounding the mutation site C206. (A) WT; (B) Mutant C206R. The residues are presented in sticks: green sticks represent C206 in WT and R206 in the mutant; yellow sticks represent G238; and cyan sticks represent D239. The red dash indicates the hydrogen bonds.

The energy calculations indicated that all mutations stabilize the monomeric structure of HsSMS. The couple, E175 and H178, increases stability by lowering the desolvation penalty for D201, which is protonated in the mutant, and by providing stabilizing hydrogen bonds. The other two mutations, S165D and C206R, also stabilize the monomer, especially C206R is predicted to increase the monomer stability by 15.77 kcal/mol. In the WT structure, C206 forms a hydrogen bond with D239 (Figure 4A); while in the CRmut structure, R206 forms hydrogen bonds with the backbone oxygens of both D239 and G238 (Figure 4B). The extra hydrogen bond is the main reason for increased stability of CRmut. The S165D stabilizes the monomer as well, because the WT residue, Ser 165 is not involved in any specific interactions, while in the mutant, the Asp 165 interacts with Lys 163 and Lys 167 while being exposed, does not pay any desolvation penalty.

At the same time, the analysis showed that neither of the mutations nor the Fmut affects the HsSMS dimer affinity. Structurally they are far away from the dimer interface and are situated in the C-terminal domain. Because of that, from structural perspective they are not expected to alter the dimer affinity. The fact that computation protocol predicted no effect is reassuring and indicates that numerical protocol is robust.

How the increased stability may be related to enhanced activity is not an obvious question. Typically a decrease of the stability (or folding free energy) caused by mutation is considered to be bad for the function of proteins and frequently associated with diseases [17]–[22]. However, the opposite effect of increasing stability was also shown to cause disease [23], [24]. Perhaps the key factor of estimating the relations between stability and function is the understanding the mechanism of the corresponding reaction. The HsSMS needs to bind two reactants and to release two products, while being a dimer. The less conformational changes are involved in the reaction, the faster the reaction will be. It can be speculated that such predicted overall rigidification will enhance the reaction rate, while allowing the necessary local and small conformational changes still to be allowed to carry the conversion SPD→SPM. Similar effect, an increase of the reaction rate upon mutations to be associated with structural stabilization was reported before [25], [26].

One of the most important finding is that the four mutations, Fmut, result in a structure which carries more negative charge than the WT. The distribution of the extra negative charge is not uniform and results in stronger (negative) potential within the dimer cleft (path “A”) and within a patch at the bottom of the C-terminal domain (path “B”) (Figure 2A, Figure 2B). There is no experimental data to provide insights why the dimerization is necessary for the HsSMS function, except the original work which showed that if N-domain is removed the HsSMS does not form dimer and does not function [4]. However, each of the HsSMS monomers carries an active site and in principle should be capable to function as a monomer. Perhaps the dimerization is needed not for the reaction itself, but to promote the delivery of the reactants and the release of the products of the reaction. Electrostatic calculations support such a notion. Indeed in both the WT and the mutant, we identified two plausible electrostatic funnels, marked as path “A” and “B” in Figure 2A, however the potential is more negative in the mutant. It can be speculated that this extra negative potential provides better steering of the SPD to enter the dimer cleft and to increase the concentration of SPD in the proximity of the active sites. Indeed, the experimental data suggests that the affinity of HsSMS to SPD is higher in the mutant (Table 7). In addition, the dramatic increase of the reaction rate could be attributed to the increase of the maximal rate (V_m_) in the mutant, which in turn can be effect of the increased effective concentration of SPD at the entrance of the active site.

From the normal mode analysis, we can see the Fmut has a slightly higher frequency of domain motion than the WT, the difference being 170∼260 MHz. Such difference most probably will affect the rate and efficiency of the reaction by affecting the substrate approach to and release from the active site. Following the original work of Zhou and coworkers [27], it can be speculated that HsSMS dimer forms a conformation gate. During the reaction, the substrate, the SPD, follows the electrostatic funnels and makes repeated attempts to go inside the active pocket. As pointed by Zhou and co-workers [27], the higher the frequency of the gate is, the better is the chance that the substrate will successfully pass the gate, since the gate will be more frequently open when the SPD happens to be at the entrance. The same speculation can be made for the release of the products, i.e. the increased frequency of gate switching should increase the efficiency of product releasing [27].

Taking all these effects together, seems to us that increased activity in the mutant is due to a combinations of factors, ranging from enhanced access of the substrates to the active site via better electrostatic guiding and gate switching, structural stabilization and better protonation environment for the reaction. This indicates the complexity of the reaction process taking place in SMS and shows the complex interplay between structural and thermodynamic factors.

At the same time, it should be noticed that the amino acid sequence of HsSMS currently does not have known variants. A search in various databases (as for example dbSNP at NCBI, NIH [28]–[30]) resulted in no hits. The only mutations found in human population are the missense mutations causing Snyder-Robinson syndrome [6]–[9]. Our recent computations analysis [10] indicated that HsSMS should be able to tolerate mutations even at the disease-causing sites; however, such variants are not seen in the human population yet. Combining these observations with the result of this manuscript, it can be speculated that the function of HsSMS is precisely tuned toward its wild type value to satisfy some currently unknown constraints in vivo. Perhaps, any deviation from the wild type sequence is unwanted and disease-causing. However, caution should be used in the interpretation of the *in silico* results, since the threshold indicating a deviation is perhaps specific for each protein and reaction involved.

## Methods

### I) *In silico* modeling

#### a) Protein structure

The wild-type (WT) structure of human spermine synthase protein crystallized with SPD (PDB ID 3C6K) [4] were downloaded from the Protein Data Bank (PDB) website. There are four molecules (A B C and D) forming two dimers (A–B dimer and C–D dimer) in each asymmetrical unit. In the simulations only C–D dimer was used as a biological unit, whereas the A–B dimer is removed because of significant van der Waals (vdW) clashes presented in the X-ray structure. The residues not resolved in the X-ray structures were rebuilt with the program Profix in Jackal package (http://wiki.c2b2.columbia.edu/honiglab_public/index.php/Software:Jackal).

Comparing the protein sequence of HsSMS and TmSRM, four mutations were made in HsSMS: p. S165D p. L175E, p. T178H, and p. C206R (they are p. S180D, p.L190E, p.T193H and p.C221R in the amino acid sequence, respectively). The selection was based on several considerations: a) mutations sites should be on the surface of the HsSMS so that their substitution would not to cause any significant structural perturbations. Thus, S165D, L175E and C206R were selected since they are located at the surface of HsSMS; b) any residue predicted should be correlated with the above three to address the cooperativity. Thus, the T178H was selected since it is predicted to be correlated with L175E; c) Since the goal is to alter the properties of HsSMS, only substitutions resulting in a change of the biophysical properties such as the polarity or the hydrophobicity between the WT residue and the mutant residue were considered; d) In order to avoid abolishing the function of HsSMS, only sites that were not conserved in multiple sequence alignments were analyzed. However, preference was given to non-conserved sites neighboring other well-conserved sites since it was expected that such a substitution would have a larger effect on HsSMS than a substitution at an isolated non-conserved site; (e) Preference was given to amino acid substitutions that occurred with the highest frequency in the multiple sequence alignment. Taking in all these considerations, the four amino acid substitutions mentioned above are subject of the investigations described in this work, with the goal of enhancing the WT properties of HsSMS.

**Figure 5:**
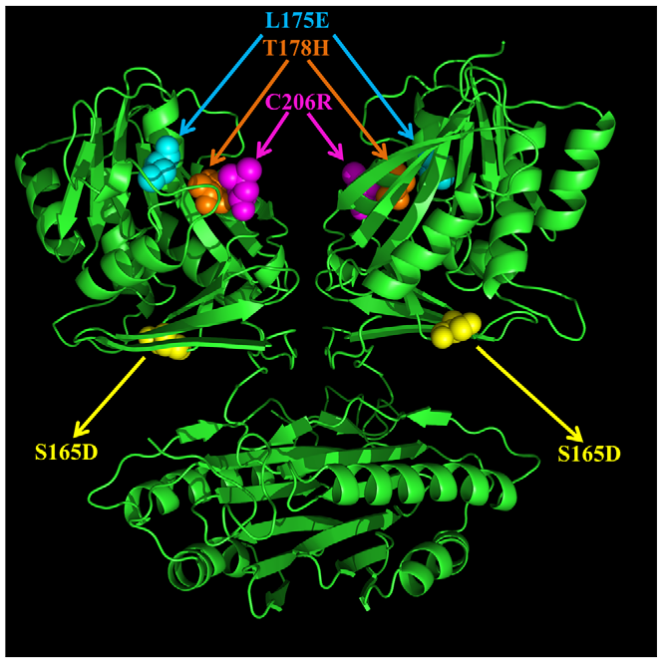
3D structure of HsSMS dimer in ribbon presentation. Four mutation sites are shown with ball representation: Yellow: S165D; Magenta: C206R; Cyan: L175E; and Orange: T178H.

The mutant structures were built *in silico* by side chain replacement through the program SCAP in Jackal package [31]. Several mutant structures were generated in both C chain and D chain: a) the mutant with a single mutation S165D, which is named “SDmut”; b) the mutant with a single mutation C206R, which is named “CRmut”; c) the double mutant with two mutations L175E and T178H together. Making such “PAIR” mutations was prompted by the analysis of multiple sequence alignment (MSA), which will be introduced in a latter section. This mutant is named “Pmut”; d) the mutant with all of the above four mutations S165D, C206R, L175E and T178H. This mutant was named “Fmut”. The mutation sites are shown in Figure 5.

#### b) Multiple sequence alignment

To investigate the evolutionary relationship of the above four mutations among different species and homologous proteins, the multiple sequence alignment (MSA) was performed by Constraint-based Multiple Alignment Tool (COBALT) [32], which is currently provided by National Center for Biotechnology Information (NCBI) [33]. Three datasets were selected to do such an investigation: a) MSA only between HsSMS and TmSRM; b) MSA among SMS from different species; c) MSA among all the homologous to HsSMS proteins.

Protein sequences between HsSMS and TmSRM were aligned by COBALT since these four mutations were suggested by TmSRM. Additionally this comparison is intended to give a global view of sequence differences between these two species.

The pioneering work on HsSMS [4] analyzed MSA of representative members of SMS family among different species: human (Homo sapiens; NP_004586), chicken (Gallus gallus; NP_001025974), zebrafish (Danio rerio; NP_571831), fruit fly (Drosophila melanogaster; NP_729798), mosquito (Anopheles gambiae; XP_315341), bee (Apis mellifera; XP_393567), sea urchin (S. purpuratus; XP_789223), sea anemone (N. vectensis; XP_001636780), M. brevicollis (Joint Genome Institute protein ID 30201), A. thaliana (NP_568785), and Saccharomyces cerevisiae (AAC19368). In the present work, we use the same species mentioned above. The FASTA sequences were downloaded from NCBI and uploaded to COBALT for the MSA analysis.

MSA among the homologous proteins were also investigated with the same tools. Protein-BLAST (Basic Local Alignment Search Tool) [16] was used to search the homologous proteins. The max target sequences were set as 500 to include the sequences of all of the above SMS from difference species along with the sequence of other homologues proteins.

#### c) pKa calculations

Since all of the above four mutations involve titratable group, pKa calculations were carried out to investigate the ionized states of each titratable group in both WT and mutants. All titratable groups were analyzed, because it is known that mutations may affect the ionized states of neighboring residues by either perturbing the original dielectric boundary of the protein or altering the hydrogen bond network [34], [35]. The differences of pKa value of each titratable group between the WT and mutants were calculated by Eq. (1):
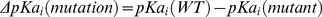
(1)where *ΔpKa_i_ (mutation)* is the pKa change of amino acid *i* due to the mutations; while *pKa_i_ (WT)* and *pKa_i_ (mutant)* are the pKa values of amino acid *i* in the WT and mutants respectively.

pKa was calculated by Multi Confirmation Continuum Electrostatics (MCCE) [36]–[38], version 2.4 (http://134.74.90.158). The default parameters were used and the dielectric constant of protein was set as 8.0. For both of the WT and mutants, the calculation was performed on monomer C, monomer D and dimer CD respectively. It should be mentioned that standard rigid body pKa's calculations are very sensitive to the initial structure used, especially in terms of side chain orientations. The importance of accurate predictions of side chain rotamers for molecular biology is illustrated by numerous studies [39]–[44] including developing tools such as SCWRL [45], [46] and SIDEpro [47]. MCCE takes advantage of these developments and uses Dunbrack conformer libraries [40], [42] to sample the side chain rotamers within the calculations of the pKa's of titratable residues and thus the predictions are independent of the initial side chain positions.

#### d) Energy calculations

The effects of the mutations were modeled on two energies, folding energy and binding energy. In order to save the calculating time and reduce unwanted artifacts, we removed N-domain (Residue number: 2–109) in the calculation since all of these four mutations were located in the C-domain and far away from the N-domain (Figure 5) [9], [10]. For each of the energy calculations in the present work, the TINKER package [48] was applied and three force fields (Amber 98 [49], Charmm27 [50] and OPLSaa [51]) were used and results averaged. The solvent was modeled with the Still Generalized Born model [52], and the internal dielectric constant was set up to 1.0. The structures of the dimer and the corresponding monomers were energy minimized with the “minimize” module of TINKER package utilizing Clemson's high performance computing cluster, Palmetto (HPC). The minimization was done via Limited Memory BFGS Quasi-Newton Optimization algorithm within the TINKER package [48]. The convergence criteria applied was RMS gradient per atom equal to 0.01. After successful energy minimization, the potential energies for the minimized structures were obtained with “analyze” module of TINKER package.

Change of folding energy due to mutations corresponds to the change of stability of monomers. Negative folding energy change indicates that the mutant monomers are less stable compared with WT monomers, while positive energy change means increasing stability of mutant monomers. In order to calculate the folding energy change due to the missense mutations, we applied the scaled Molecular Mechanics Generalized Born (sMMGB) method described in our previous works [9], [10], [53]. It should be mentioned that this approach uses three different force fields (AMBER98, CHARMM27, and OPLSaa) to deliver the predictions and was shown to perform better that any of these force field alone [53]:
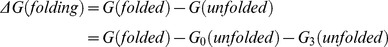
(2)where *G(folded)* is the potential energy calculated with the energy minimized structure through simulating with TINKER, and *G(unfolded)* is the potential energy of unfolded state. The unfolded state energy is separated into *G_0_(unfolded)* and *G_3_(unfolded)*. *G_3_(unfolded)* is calculated on a three-residue segment taken out from the corresponding WT and mutant structures, whereas *G_0_(unfolded)* is considered to be mutation independent and cancels out in Eq. (3).
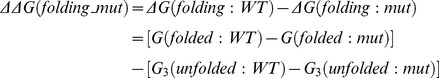
(3)


In addition, Eris [54]–[56], a webserver based on the Medusa force field [54], was also used to predict the folding energy change due to these mutations. With the experience of our previous work [9], [53], we applied a “fixed backbone” prediction method without a “backbone pre-relaxation” for Eris.

Change of binding energy due to mutation corresponds to the change of dimer affinity. Negative change of binding energy represents that affinity of mutant dimers decreased comparing to that of WT dimers. The binding energy (*ΔΔ*G(binding)) was defined as the differences between the potential energy of the dimer and the potential energies of the corresponding monomers as described in our previous works [9], [10], [35]. The energy components include mechanical energy (MM), electrostatic interactions and solvation energy. Thus, the binding free energy is calculated as:
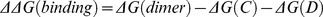
(4)where ΔG(dimer) is the potential energy of the dimer, ΔG(C) and ΔG(D) are the potential energies of monomer “C” and “D” respectively. The binding energies were calculated for WT structures and all mutant structures (SDmut, CRmut, Pmut and Fmut). The binding energy difference between WT and mutant structures was calculated as follows:
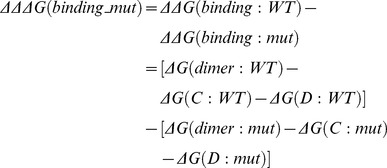
(5)where *ΔΔ*G(binding: WT) is the binding energy of the WT monomers and *ΔΔ*G(binding: mutant) is the binding energy of the corresponding mutant monomer chains.

#### e) Potential map generation and analysis

Since HsSMS reaction involves highly charged substrates, it can be expected that electrostatics and potential distribution play significant roles in the HsSMS functions. To assess the effects of the mutations on the electrostatic properties of SMS, the wild type and mutant HsSMS proteins were subjected to continuum electrostatic potential calculations using DelPhi [57]. The following parameters were used: scale – 1 grid/Å; percent of protein filling of the cube – 70%; a dielectric constant of 2 for the protein and 80 for the solvent; the ionic strength 0.15 M; the water probe radius 1.4 Å; and the Stern ion exclusion layer 2.0 Å. The results were outputted into a file in CUBE format (http://compbio.clemson.edu/delphi.php) that represents the electrostatic potential at each grid point within the grid. To determine the changes in electrostatic potential due to the mutation, the electrostatic potential map for wild type protein was subtracted from one of mutant type. The results were then visualized with VMD software [58]. The *red* color represents area with negative potential difference, and *blue* color represents that with positive one. Since both substrates are positively charged, the enhancement of the negative potential is expected to facilitate their approach to active site of SMS.

#### f) Normal mode analysis

The synthesis of SPM requires the substrate SPD to come inside the enzyme and after the catalytic reaction, the product SPM will be released. Bearing in mind that domain formation is shown to be critical for HsSMS function, it is natural to assume that cleft formed between HsSMS units in the dimer is the pathway of substrates. Because of that, the frequency of the cleft “opening” and “closing” is expected to be related to the efficiency of the catalytic reaction. This mechanism, described as “conformational gating”, was investigated by Zhou et al. in the case of acetylcholinesterase [27]. It was shown that the frequency of the gate opening and closing affects the substrate entering to the active site.

The theory and methodology of normal mode (NM) is well developed and described elsewhere [59], [60]. In the NM model, the protein is presented as an elastic network [61] and each residue of protein is modeled as a node in the network and the connections between nodes are described as elastic potentials. In particular, the anisotropic network model (ANM) [61], [62] considers the molecule as a collection of N sites (one site for each residue, resulting in ensemble of 3N-6 independent modes). An important parameter in the ANM analysis is the force constant γ, describing the strength of intramolecular potentials. The optimal value of γ has been obtained by comparing the theoretically predicted mean-square fluctuations of alpha-carbons with those indicated by the X-ray crystallographic B-factor and it was found that the best value is 1.0±0.5 kcal/(mol·Å^2^) [61]. In our analysis we used 1.0 kcal/(mol·Å^2^) as the optimal value.

The cutoff distance *r_c_* is another parameter in the ANM model which requires optimization. It specifies the threshold distance within which the residues/atoms are considered to interact with each other. In this work, *r_c_* and distance weight for interaction between C_α_ atoms are set as the default parameters shown on the website (*r_c_* = 15 Å and the distance weight is 0). All of the TINKER minimized structures were submitted to the ANM webserver (http://ignmtest.ccbb.pitt.edu/cgi-bin/anm/anm1.cgi) [62]. Analysis of the predicted vibrational modes with respect to the plausible substrate pathways (pathways A and B in Figure 2) made us select mode 1, mode 2, and mode 3 for further investigations. The predicted motions (the directions of the vibrational vectors) and their plausible implications for the SPD/SPM pathways can be seen in the supplementary material (Figure S1). The corresponding frequencies were calculated as:
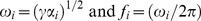
(6)where γ is the force constant; 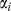 is the *i*th eigenvalue, for 1≤*i*≤3N-6 (for 3D or N-1 for 1D) and 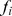 is the frequency.

### II) Experiments

#### a) Expression and purification of HsSMS

A DNA fragment encoding HsSMS was amplified by PCR and subcloned into the pQE-30 vector downstream of the polyhistidine coding region [63]. The resulting plasmids were used to transform XL1-Blue cells. Recombinant human SS was purified by immobilized metal affinity chromatography using TALON affinity resin (Clontech Laboratories, Palo Alto, CA, U.S.A.), in accordance with manufacturer's instruction.

#### b) Assay of HsSMS activity

Activity was measured by following the production of [^35^S]MTA from [^35^S]dcAdoMet in 100 mM sodium phosphate buffer (pH 7.5), in the presence of SPD [64]. The reaction was stopped by acidification and [^35^S]MTA separated from [^35^S]dcAdoMet using phosphocellulose columns. All assays were conducted with an amount of protein and for a period of time in which product formation was linear with time. Reactions were run with an amount of enzyme that gave a linear rate of MTA production over the assay time period. Assays were conducted for 1 h at 37°C. All assays were carried out in triplicate, and results agreed within ±5%.

#### c) Production of HsSMS mutants

The four amino acid mutated HsSMS was generated by PCR and subcloned into the pQE30 vector. The following oligodeoxynucleotides (with mismaches underlined) were used to generate the mutant indicated:

5′-ATCACTCTCTGCCAAATTAACATCCCCATCAAGGATGAGAAT-3′ (antisense) for S165D

5′-TTGGCAGAGAGTGATGAGGCATATCACCGGGCCATCATG-3′ (sense) for L175E and T178H

5′-GGAGGCATATTGCGTGAAATAGTCAAA-3′ (sense) for C206R

5′-TTTGACTATTTCACGCAATATGCCTCC-3′ (antisense) for C206R

The entire coding sequence of each of the SMS mutants was verified by DNA sequencing to ensure that no other mutations were introduced during PCR. The entire coding region of both plasmids was verified by DNA sequencing carried out by the Macromolecular Core Facility, Hershey Medical Center. The SMS mutant proteins were purified as described above before assays.

## Supporting Information

Figure S1 The first three vibrational modes calculated with the ANM server. The plausible pathways of substrates SPD/SPM are indicated with the black arrows. The letter code of pathways “A” (the cleft between C-terminal domains) and “B” (the cleft between adjacent C- and N-terminal domains) corresponds to Figure 2 in the main body of the manuscript. The directions of vibrational vectors of the three vibrational modes are shown with orange arrows.
(DOCX)

Table S1 Candidate sites for engineered mutations selected based on 3D structure of HsSMS.
(DOCX)

Table S2 The MSA among different species. The highly conserved residues are marked with “*” in the alignment.
(DOCX)

Table S3 MSA among 500 homologous proteins.
(DOCX)

## References

[1] Gerner EW, Meyskens FL Jr (2004) Polyamines and cancer: old molecules, new understanding. Nat Rev Cancer 4: 781–792. 15510159 10.1038/nrc1454

[2] Pegg AE, Michael AJ (2010) Spermine synthase. Cell Mol Life Sci 67: 113–121. 19859664 10.1007/s00018-009-0165-5 PMC2822986

[3] Cason AL, Ikeguchi Y, Skinner C, Wood TC, Holden KR, et al (2003) X-linked spermine synthase gene (SMS) defect: the first polyamine deficiency syndrome. European journal of human genetics 11: 937–944. 14508504 10.1038/sj.ejhg.5201072

[4] Wu H, Min J, Zeng H, McCloskey DE, Ikeguchi Y, et al (2008) Crystal structure of human spermine synthase: implications of substrate binding and catalytic mechanism. J Biol Chem 283: 16135–16146. 18367445 10.1074/jbc.M710323200 PMC3259631

[5] Bas DC, Rogers DM, Jensen JH (2008) Very fast prediction and rationalization of pKa values for protein–ligand complexes. Proteins: Structure, Function, and Bioinformatics 73: 765–783. 10.1002/prot.22102 18498103

[6] de Alencastro G, McCloskey DE, Kliemann SE, Maranduba CM, Pegg AE, et al (2008) New SMS mutation leads to a striking reduction in spermine synthase protein function and a severe form of Snyder-Robinson X-linked recessive mental retardation syndrome. J Med Genet 45: 539–543. 18550699 10.1136/jmg.2007.056713

[7] Becerra-Solano LE, Butler J, Castaneda-Cisneros G, McCloskey DE, Wang X, et al (2009) A missense mutation, p.V132G, in the X-linked spermine synthase gene (SMS) causes Snyder-Robinson syndrome. Am J Med Genet A 149A: 328–335. 19206178 10.1002/ajmg.a.32641 PMC2653108

[8] Schwartz CE, Wang X, Stevenson RE, Pegg AE (2011) Spermine synthase deficiency resulting in X-linked intellectual disability (Snyder-Robinson syndrome). Methods Mol Biol 720: 437–445. 21318891 10.1007/978-1-61779-034-8_28

[9] Zhang Z, Teng S, Wang L, Schwartz CE, Alexov E (2010) Computational analysis of missense mutations causing Snyder-Robinson syndrome. Hum Mutat 31: 1043–1049. 20556796 10.1002/humu.21310 PMC2932761

[10] Zhang Z, Norris J, Schwartz C, Alexov E (2011) In silico and in vitro investigations of the mutability of disease-causing missense mutation sites in spermine synthase. PLoS One 6: e20373. 21647366 10.1371/journal.pone.0020373 PMC3103547

[11] Zhang Z, Witham S, Alexov E (2011) On the role of electrostatics in protein-protein interactions. Phys Biol 8: 035001. 21572182 10.1088/1478-3975/8/3/035001 PMC3137121

[12] Zhang Z, Miteva MA, Wang L, Alexov E (2012) Analyzing effects of naturally occurring missense mutations. Comput Math Methods Med 2012: 805827. 22577471 10.1155/2012/805827 PMC3346971

[13] Ikeguchi Y, Bewley MC, Pegg AE (2006) Aminopropyltransferases: function, structure and genetics. Journal of biochemistry 139: 1–9. 16428313 10.1093/jb/mvj019

[14] Huber R, Langworthy TA, Konig H, Thomm M, Woese CR, et al (1986) Thermotoga-Maritima Sp-Nov Represents a New Genus of Unique Extremely Thermophilic Eubacteria Growing up to 90-Degrees-C. Arch Microbiol 144: 324–333.

[15] Wu H, Min J, Ikeguchi Y, Zeng H, Dong A, et al (2007) Structure and mechanism of spermidine synthases. Biochemistry 46: 8331–8339. 17585781 10.1021/bi602498k

[16] Altschul SF, Gish W, Miller W, Myers EW, Lipman DJ (1990) Basic local alignment search tool. J Mol Biol 215: 403–410. 2231712 10.1016/S0022-2836(05)80360-2

[17] Tan Y, Luo R (2009) Structural and functional implications of p53 missense cancer mutations. PMC Biophys 2: 5. 19558684 10.1186/1757-5036-2-5 PMC2709103

[18] Boeckler FM, Joerger AC, Jaggi G, Rutherford TJ, Veprintsev DB, et al (2008) Targeted rescue of a destabilized mutant of p53 by an in silico screened drug. Proc Natl Acad Sci U S A 105: 10360–10365. 18650397 10.1073/pnas.0805326105 PMC2492497

[19] Steen M, Miteva M, Villoutreix BO, Yamazaki T, Dahlback B (2003) Factor V New Brunswick: Ala221Val associated with FV deficiency reproduced in vitro and functionally characterized. Blood 102: 1316–1322. 12714495 10.1182/blood-2003-01-0116

[20] Minutolo C, Nadra AD, Fernandez C, Taboas M, Buzzalino N, et al (2011) Structure-based analysis of five novel disease-causing mutations in 21-hydroxylase-deficient patients. PLoS One 6: e15899. 21264314 10.1371/journal.pone.0015899 PMC3019215

[21] Miteva MA, Brugge JM, Rosing J, Nicolaes GA, Villoutreix BO (2004) Theoretical and experimental study of the D2194G mutation in the C2 domain of coagulation factor V. Biophys J 86: 488–498. 14695293 10.1016/S0006-3495(04)74127-2 PMC1303816

[22] Howes J, Shimizu Y, Feige MJ, Hendershot LM (2012) C-terminal mutations destabilize SIL1/BAP and can cause Marinesco-Sjogren syndrome. J Biol Chem 287: 8552–8560. 22219183 10.1074/jbc.M111.333286 PMC3318681

[23] Witham S, Takano K, Schwartz C, Alexov E (2011) A missense mutation in CLIC2 associated with intellectual disability is predicted by in silico modeling to affect protein stability and dynamics. Proteins 79: 2444–54. 21630357 10.1002/prot.23065 PMC3132293

[24] Takano K, Liu D, Tarpey P, Gallant E, Lam A, et al (2012) An X-linked channelopathy with cardiomegaly due to a CLIC2 mutation enhancing ryanodine receptor channel activity. Hum Mol Genet 21: 10. 22814392 10.1093/hmg/dds292 PMC3459470

[25] Persson E, Bak H, Ostergaard A, Olsen OH (2004) Augmented intrinsic activity of Factor VIIa by replacement of residues 305, 314, 337 and 374: evidence of two unique mutational mechanisms of activity enhancement. Biochem J 379: 497–503. 14686879 10.1042/BJ20031596 PMC1224069

[26] Huang JW, Cheng YS, Ko TP, Lin CY, Lai HL, et al (2012) Rational design to improve thermostability and specific activity of the truncated Fibrobacter succinogenes 1,3-1,4-beta-D-glucanase. Appl Microbiol Biotechnol 94: 111–121. 21959377 10.1007/s00253-011-3586-7

[27] Zhou HX, Wlodek ST, McCammon JA (1998) Conformation gating as a mechanism for enzyme specificity. Proc Natl Acad Sci U S A 95: 9280–9283. 9689071 10.1073/pnas.95.16.9280 PMC21329

[28] Sherry ST, Ward MH, Kholodov M, Baker J, Phan L, et al (2001) dbSNP: the NCBI database of genetic variation. Nucleic Acids Res 29: 308–311. 11125122 10.1093/nar/29.1.308 PMC29783

[29] Smigielski EM, Sirotkin K, Ward M, Sherry ST (2000) dbSNP: a database of single nucleotide polymorphisms. Nucleic Acids Res 28: 352–355. 10592272 10.1093/nar/28.1.352 PMC102496

[30] Sherry ST, Ward M, Sirotkin K (1999) dbSNP-database for single nucleotide polymorphisms and other classes of minor genetic variation. Genome Res 9: 677–679. 10447503

[31] Xiang Z, Honig B (2001) Extending the accuracy limits of prediction for side-chain conformations. J Mol Biol 311: 421–430. 11478870 10.1006/jmbi.2001.4865

[32] Papadopoulos JS, Agarwala R (2007) COBALT: constraint-based alignment tool for multiple protein sequences. Bioinformatics 23: 1073–1079. 17332019 10.1093/bioinformatics/btm076

[33] Sayers EW, Barrett T, Benson DA, Bolton E, Bryant SH, et al (2012) Database resources of the National Center for Biotechnology Information. Nucleic Acids Res 40: D13–25. 22140104 10.1093/nar/gkr1184 PMC3245031

[34] Talley K, Ng K, Shroder M, Kundrotas P, Alexov E (2008) On the electrostatic component of the binding free energy. PMC Biophysics 1: 2. 19351424 10.1186/1757-5036-1-2 PMC2666630

[35] Teng S, Madej T, Panchenko A, Alexov E (2009) Modeling effects of human single nucleotide polymorphisms on protein-protein interactions. Biophys J 96: 2178–2188. 19289044 10.1016/j.bpj.2008.12.3904 PMC2717281

[36] Alexov E, Gunner M (1999) Calculated Protein and Proton Motions Coupled to Electron Transfer: Electron Transfer from QA- to QB in Bacterial Photosynthetic Reaction Centers. Biochemistry 38: 8253–8270. 10387071 10.1021/bi982700a

[37] Georgescu RE, Alexov EG, Gunner MR (2002) Combining conformational flexibility and continuum electrostatics for calculating pK(a)s in proteins. Biophys J 83: 1731–1748. 12324397 10.1016/S0006-3495(02)73940-4 PMC1302268

[38] Song Y, Mao J, Gunner MR (2009) MCCE2: Improving Protein pKa Calculations with Extensive Side Chain Rotamer Sampling. Comp Chem 30: 2231–2247. 10.1002/jcc.21222 PMC2735604 19274707

[39] Dunbrack RL Jr, Karplus M (1993) Backbone-dependent rotamer library for proteins. Application to side-chain prediction. J Mol Biol 230: 543–574. 8464064 10.1006/jmbi.1993.1170

[40] Dunbrack RL Jr, Cohen FE (1997) Bayesian statistical analysis of protein side-chain rotamer preferences. Protein Sci 6: 1661–1681. 9260279 10.1002/pro.5560060807 PMC2143774

[41] Bower MJ, Cohen FE, Dunbrack RL Jr (1997) Prediction of protein side-chain rotamers from a backbone-dependent rotamer library: a new homology modeling tool. J Mol Biol 267: 1268–1282. 9150411 10.1006/jmbi.1997.0926

[42] Dunbrack RL Jr, Karplus M (1994) Conformational analysis of the backbone-dependent rotamer preferences of protein sidechains. Nat Struct Biol 1: 334–340. 7664040 10.1038/nsb0594-334

[43] Shapovalov MV, Dunbrack RL Jr (2007) Statistical and conformational analysis of the electron density of protein side chains. Proteins 66: 279–303. 17080462 10.1002/prot.21150

[44] Wang Q, Canutescu AA, Dunbrack RL Jr (2008) SCWRL and MolIDE: computer programs for side-chain conformation prediction and homology modeling. Nat Protoc 3: 1832–1847. 18989261 10.1038/nprot.2008.184 PMC2682191

[45] Krivov GG, Shapovalov MV, Dunbrack RL Jr (2009) Improved prediction of protein side-chain conformations with SCWRL4. Proteins 77: 778–795. 19603484 10.1002/prot.22488 PMC2885146

[46] Canutescu AA, Shelenkov AA, Dunbrack RL Jr (2003) A graph-theory algorithm for rapid protein side-chain prediction. Protein Sci 12: 2001–2014. 12930999 10.1110/ps.03154503 PMC2323997

[47] Nagata K, Randall A, Baldi P (2012) SIDEpro: a novel machine learning approach for the fast and accurate prediction of side-chain conformations. Proteins 80: 142–153. 22072531 10.1002/prot.23170 PMC3240718

[48] Ponder JW (1999) TINKER-software tools for molecular design, version 4.2. Washington University School of Medicine: Saint Louis. Available: http://dasher.wustl.edu/tinker/.

[49] Case DA, Cheatham TE 3rd, Darden T, Gohlke H, Luo R, et al (2005) The Amber biomolecular simulation programs. J Comput Chem 26: 1668–1688. 16200636 10.1002/jcc.20290 PMC1989667

[50] Brooks BR, Brooks CL 3rd, Mackerell AD Jr, Nilsson L, Petrella RJ, et al (2009) CHARMM: the biomolecular simulation program. J Comput Chem 30: 1545–1614. 19444816 10.1002/jcc.21287 PMC2810661

[51] Jorgensen WL, Tiradorives J (1988) The Opls Potential Functions for Proteins - Energy Minimizations for Crystals of Cyclic-Peptides and Crambin. Journal of the American Chemical Society 110: 1657–1666. 27557051 10.1021/ja00214a001

[52] Still WC, Tempczyk A, Hawley RC, Hendrickson T (1990) Semianalytical Treatment of Solvation for Molecular Mechanics and Dynamics. Journal of the American Chemical Society 112: 6127–6129.

[53] Zhang Z, Wang L, Gao Y, Zhang J, Zhenirovskyy M, et al (2012) Predicting folding free energy changes upon single point mutations. Bioinformatics 28: 664–671. 22238268 10.1093/bioinformatics/bts005 PMC3289912

[54] Ding F, Dokholyan NV (2006) Emergence of protein fold families through rational design. Plos Computational Biology 2: 725–733. 10.1371/journal.pcbi.0020085 PMC1487181 16839198

[55] Yin S, Ding F, Dokholyan NV (2007) Eris: an automated estimator of protein stability. Nat Methods 4: 466–467. 17538626 10.1038/nmeth0607-466

[56] Yin S, Ding F, Dokholyan NV (2007) Modeling backbone flexibility improves protein stability estimation. Structure 15: 1567–1576. 18073107 10.1016/j.str.2007.09.024

[57] Li L, Li C, Sarkar S, Zhang J, Witham S, et al (2012) DelPhi: a comprehensive suite for DelPhi software and associated resources. BMC Biophys 5: 9. 22583952 10.1186/2046-1682-5-9 PMC3463482

[58] Humphrey W, Dalke A, Schulten K (1996) VMD: visual molecular dynamics. Journal of molecular graphics 14: 33–38. 8744570 10.1016/0263-7855(96)00018-5

[59] Gibrat JF, Go N (1990) Normal mode analysis of human lysozyme: study of the relative motion of the two domains and characterization of the harmonic motion. Proteins 8: 258–279. 2281087 10.1002/prot.340080308

[60] Brooks B, Karplus M (1985) Normal modes for specific motions of macromolecules: application to the hinge-bending mode of lysozyme. Proc Natl Acad Sci U S A 82: 4995–4999. 3860838 10.1073/pnas.82.15.4995 PMC390485

[61] Atilgan AR, Durell SR, Jernigan RL, Demirel MC, Keskin O, et al (2001) Anisotropy of fluctuation dynamics of proteins with an elastic network model. Biophys J 80: 505–515. 11159421 10.1016/S0006-3495(01)76033-X PMC1301252

[62] Eyal E, Yang LW, Bahar I (2006) Anisotropic network model: systematic evaluation and a new web interface. Bioinformatics 22: 2619–2627. 16928735 10.1093/bioinformatics/btl448

[63] Ikeguchi Y, Mackintosh CA, McCloskey DE, Pegg AE (2003) Effect of spermine synthase on the sensitivity of cells to anti-tumour agents. Biochem J 373: 885–892. 12737625 10.1042/BJ20030246 PMC1223546

[64] Wiest L, Pegg AE (1998) Assay of spermidine and spermine synthases. Methods Mol Biol 79: 51–57. 9463817 10.1385/0-89603-448-8:51

