# Supplementary material for: Enhancing Human Spermine Synthase Activity by Engineered Mutations
Source: PLoS Comput Biol. 2013 Feb 28;9(2):e1002924. doi: 10.1371/journal.pcbi.1002924 (PMC3585406; doi:10.1371/journal.pcbi.1002924)

**Figure S1:** The first three vibrational modes calculated with the ANM server. The plausible pathways of substrates SPD/SPM are indicated with the black arrows. The letter code of pathways “A” (the cleft between C-terminal domains) and “B” (the cleft between adjacent C- and N-terminal domains) corresponds to Figure 2 in the main body of the manuscript. The directions of vibrational vectors of the three vibrational modes are shown with orange arrows.


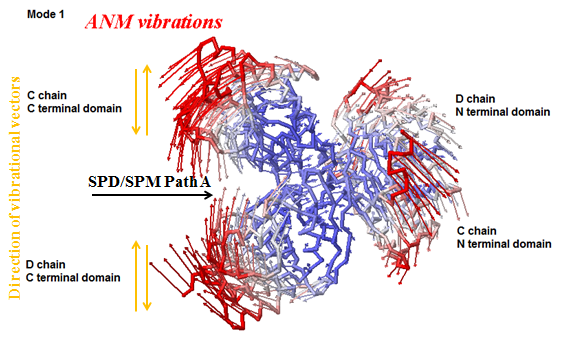


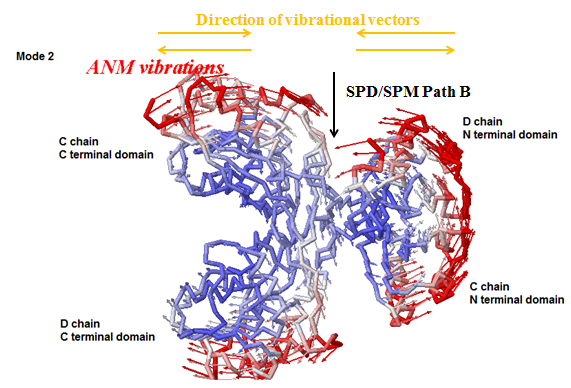


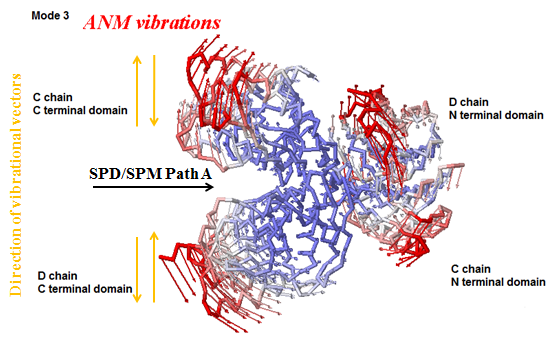

Supplement: Figure S1 — The first three vibrational modes calculated with the ANM server. The plausible pathways of substrates SPD/SPM are indicated with the black arrows. The letter code of pathways “A” (the cleft between C-terminal domains) and “B” (the cleft between adjacent C- and N-terminal domains) corresponds to Figure 2 in the main body of the manuscript. The directions of vibrational vectors of the three vibrational modes are shown with orange arrows. (DOCX) [file pcbi.1002924.s001.docx]
