# Supplementary material for: Enhancing Human Spermine Synthase Activity by Engineered Mutations
Source: PLoS Comput Biol. 2013 Feb 28;9(2):e1002924. doi: 10.1371/journal.pcbi.1002924 (PMC3585406; doi:10.1371/journal.pcbi.1002924)
Supplement: Table S1 — Candidate sites for engineered mutations selected based on 3D structure of HsSMS. (DOCX) [file pcbi.1002924.s002.docx]

**Table S1:** Candidate sites for engineered mutations selected based on 3D structure of HsSMS. All of the residues listed in the following table are located at the surface of the protein. The invariant residues are removed.

D137, E138, D142, E143, D144, N149, K151, K156, Q157, N160, S165, L175, R179, S184, G185, K186, E187, D188, T190, D193, C206, E207, L211, K212, K214, M215, Q223, M224, D227, R234, C237, D242, N243, K245, D247, E254, K261, R262, K265, E266, R268, T284, P286, E287, D289, E293, R296, L297, D300, K304, K311, N320, T322, E323, S326, L327, E330, R334, E340, C347, A364, K365, P366

Significant fraction of the candidate mutation sites are occupied by charged group (Asp, Glu, Lys and Arg) which participate in complex network of pair-wise interactions, including salt-bridges. To avoid its disruption, these sites with charged group were removed from the candidate list. The remaining list of twenty five sites is provided below:

N149, Q157, N160, S165, L175, S184, G185, T190, C206, L211, M215, Q223, M224, C237, N243, T284, P286, L297, N320, T322, S326, L327, C347, A364 and P366.

Further refinement of the candidate list was based on the MSA (Table S2). Sites located next to conserved site were given preference with respect to isolated non-conserved sites (Table S2). This resulted in eight residues: N149, N160, S165, L175, C206, Q223, M224, and C347.

Additionally N149 is just next to a disease-causing mutation site I150 (Zhang Z, Teng S, Wang L, Schwartz CE, Alexov E (2010) Hum Mutat 31: 1043-1049; Zhang Z, Norris J, Schwartz C, Alexov E (2011) PloS one 6: e20373) and quite close to the active site. Because of that it is considered that its mutation could abolish the function of HsSMS and this candidate was removed from the list.

Frequency and percentage of residue appearance at the mutation sites among 500 homologous proteins suggested the final selection: if the TmSRM🡪HsSMS substitution is found to appear less than 50% in the MSA, the candidate was deleted. Thus, N160, M224 and C347 were deleted because N160🡪V, M224🡪L and C347F substitutions were found in less than 50% of cases. The site Q223 is gap (Table S3) and was deleted as well.

As result, only three sites were kelp the candidate list, namely S165🡪D, L175🡪E and C206🡪R to be mutated to the corresponding residues in TmSRM. In addition, the site T178 was included in the list because it is correlated with L175. The corresponding mutation is T178🡪H.
