# Supplementary material for: Enhancing Human Spermine Synthase Activity by Engineered Mutations
Source: PLoS Comput Biol. 2013 Feb 28;9(2):e1002924. doi: 10.1371/journal.pcbi.1002924 (PMC3585406; doi:10.1371/journal.pcbi.1002924)
Supplement: Table S2 — The MSA among different species. The highly conserved residues are marked with “*” in the alignment. (DOCX) [file pcbi.1002924.s003.docx]

**Table S2: The MSA among different species. The highly conserved residues are marked with “*” in the alignment.**

Homo sapiens MGSSHHHHHHSSGLVPRGSRHSTLDFMLGAKADGETILKG-LQSIFQEQGMAESVHTWQD

Gallus gallus MAA---------------ARHSTLDFMLGATADCNAVLKA-LQPVFQEQGMTETVHNWED

Danio rerio M-A---------------VLHYTLDFKLRAPADVSATVRG-LQSIFQEQEMTENVHDSEG

Drosophila melanogaster MAAQ-------TILFDF-TLDKDKTADEEARLQVAKILRNELEQLFPQLELAYSMESPEN

Anopheles gambiae MSAN-------SILLDF-SLDPARIIDEVSRKDIVRVCKEGLEKYLAGLKISYDMLTTD-

Apis mellifera MVAH-------TVLLDF-TVPSNVIVDVEKRSTLKLAITNVLQEYFDSLKPL--TESSID

S. purpuratus MDVR-------TVLLDFRVESEILDNLCQTGSKINEQLKGALSSV--GLGDNIQVHSSGE

N. vectensis M------------------------------------------FVF--VGPN-----SGH

M. brevicollis MAAL------------------TLASRACLNAWLLCLLCTDI-SFFQAQGPK--------

A. thaliana MEG--------------------------------------------DVGIGLVCQNTMD

Saccharomyces cerevisiae -----------------------------------------------------MVNNSQ-

Homo sapiens HGYLATYTNKNGSFANLRIYPHGLVLLDLQSYDGDAQGKEEIDSILNKVEERMKELSQD-

Gallus gallus HGYLATYIKKNGSFANLRIHPHGLVLVDLQSYNDHTKGREETDQLLNKVEERMKELFHG-

Danio rerio HGYLATFIGKNSRFAILRMHSHGLVTFDLQCLEGDDAA--QVDNLLNALEKKLKALLDG-

Drosophila melanogaster GYFAVLHEN-KDTVITCRIFQHGLLTLNVEYFLPDGKEP-------SISFDTMRTMELIL

Anopheles gambiae GYLCILSETGTGTIVTIRFFEQGLITINVEYYRKDGDEA-------KISFEQIRELENNL

Apis mellifera GSFLVLYTGPRGSLITVRGYTEGLITINIEYYKRDEEEA-------LLDFEQWRYLEADV

S. purpuratus QLFICSF--GHARSAMFSGHRPDLVTLTFQ-YDRKAKPSPTNENLPVLEEGSMEALKEEL

N. vectensis WT-LRSF--KNGHACL------DIVTVGAE--------SNNNDWYNNRYEES-KAIKESL

M. brevicollis -SALALLTSPNSPHSVQIKAFEETVTADISLSLAAGEAAPDLTDALERVRATATESLKA-

A. thaliana --------GKASNGNGLEKTVPSCCLKAMACVPEDDAK----------------------

Saccharomyces cerevisiae ------------------------------------------------------------

Homo sapiens ----STGRVKRLPPIVRGGAI-DRYWPTA---------------DGRLVEYDIDEVVYDE

Gallus gallus ----NLKRVKRLPAILRGGVI-DRYWPTA---------------DGRLVEYDIDEVVYDE

Danio rerio ----NIQRIKRLPALIRGSDV-DRYWPTA---------------DGRLMEYDIDEVVYEK

Drosophila melanogaster RQKFDSDRSKYLPPIKRGGYI-DIYMTSS---------------DERIIEYDIDKVVFEA

Anopheles gambiae VQKLKFNHGQSLPPLQRGP-L-TRYFPTA---------------DERVIEYDIDRVLFDK

Apis mellifera AMALNSQRSKRLPPVRRGT-IYDLYLTLS---------------DDRLLEYDIDKLVFEA

S. purpuratus KKVFNPKKCKLFPEISRGAAV-DRYIPTA---------------DRRLVEYDFDAATFEA

N. vectensis AKVFATEKSRALPPIIRKGPL-NPYLPTV---------------DNLIMQYDIDREIVNV

M. brevicollis APVQHLDATTLYPPVVRGGSF-PTVVPSS---------------DGLWIQYDFDELVFHE

A. thaliana -----------CHSTVVSGWF-SEPHPRSGKKGGKAVYFNNPMWPGEAHSLKVEKVLFKD

Saccharomyces cerevisiae ------------HPYIKDGWF-REINDKS--------------FPGQAFTMTVDSILYEA

Homo sapiens DSPYQNIKILHSKQFGNILIL**S**GDVNLAESD***L***-AY***T***RAIMGSGKEDYT-GKDVLILGGGD

Gallus gallus DSPFQNIKILHSKQFGNILIL**S**GDVNLAESD***L***-AY***T***RAIMGSGKEDYT-GKEVLILGGGD

Danio rerio DSAYQNIKILHSRQFGNMLIL**N**GDVNLAESD***L***-PY***T***QAIMGSGKEHYA-GKEVLILGGGD

Drosophila melanogaster RSPFQKIQIMHSKTLGNMLLL**D**ELQNIAESD***L***-IY***T***ETLMCRGVENYE-GKEICILGGGD

Anopheles gambiae RSEFQKIQIVHSRSLGNMLVL**D**ELQNIAEAD***L***-IY***T***ETLMCRGKEDYA-GKEICILGGGD

Apis mellifera RSPYQKVQIVHSKSLGNLLVL**D**ELQNISEAD***L***-IY***T***ETLMQRGKENYT-GKEIVILGGGD

S. purpuratus DSEYQNVKIMHSPQYGNMLIL**D**DDPNLAESD***L***-AY***T***QAITGNGRESYT-GKEVLILGGGD

N. vectensis DSKYQNIKILHSNQFGNMLVL**N**NDINLAESD***L***-SY***T***KAITGNGKENYK-DKTVLILGGGD

M. brevicollis KSPYQDVKIYHSRQFGNMLLL**D**DDPNLAESD***L***-AY***T***QAILGSGRFNFE-GKSVLVLGAGD

A. thaliana KSDFQEVLVFESATYGKVLVL**D**GIVQLTEKD***E***CAY***Q***EMIAHLPLCSISSPKNVLVVGGGD

Saccharomyces cerevisiae RSEFQDILIFRNKVYGTVLVL**D**GIVQCTEFD***E***FAY***Q***EMITHIAMFAHSNPKRVLIIGGGD

* * * * * * * * * * **

Homo sapiens GGIL**C**EIVKLKP-KMVTMVEIDQMVIDGCKKYMRKTCGDVLDNLKGDCYQVLIEDCIPVL

Gallus gallus GGIL**Y**EIVKLKP-KMVTMVEIDQMVIDGCKKYMRKTCGDVLDNLKGECYQVLIEDCIPVL

Danio rerio GGIL**H**EAVKLKP-KMITMVEIDELVIDGCRKHMRKTCGNVLDNLKGDCYEILVQDCVPVL

Drosophila melanogaster GALL**Y**ELLKENP-KHVVMLEIDELVMQTCNKYLNVICGDVLEKRKGDQYEIIVGDCVEYL

Anopheles gambiae GALL**Y**ELLKEGP-KMVVMLEIDEIVMQACNKYMNTICGDVLEKRTDDNYEIIVGDCMVYL

Apis mellifera GGLL**W**ELLKEKP-KFVTMLEIDDVVIKACSQHMRSICGNCLDKRKGENYEIIVGDCAKTL

S. purpuratus GGIL**H**EVLKENP-KSIIMVEIDQVVIDAAIKHLRGICYDSMDSLTGDNYQVKVADCIPIM

N. vectensis GGIL**H**HVLKEEP-KQVIMAEIDQMVVDLAVKHLRGICGDSMDSLIGPNYEVIIGDCVEIM

M. brevicollis GGVL**H**QILKLNP-ANVIMIEIDEVVINAARKHLRGICFDSMDNLTGPNYEIRVKDCLGEM

A. thaliana GGVL**R**EISRHSSVEVIDICEIDKMVIDVSKKFFPELAVG----FDDPRVQLHIGDAAEFL

Saccharomyces cerevisiae GGVL**R**EVAKHSCVEDITMVEIDSSVIELSRKFLPTLSNGA---FDDERLDLKLCDGFKFL

* * *** * *

Homo sapiens KRYAKEG--REFDYVINDLTAVPISTSPEEDSTWEFLRLILDLSMK----VLKQDGKYFT

Gallus gallus KRYAKEG--RMFDYVINDLTAVPISTSPEEDSTWEFLRLILDLSMK----VLKQDGKYFT

Danio rerio KKFAEQG--RTFDYVINDLTAVPISTAPEEDSTWEFLRLILDLSIR----VLRPGGKYFT

Drosophila melanogaster KKFIAEG--RKFDYVFGDLTDIPITDAPEGET-WDFIRTIFEHSFK----VLKPDGKYLT

Anopheles gambiae RKYIKEG--RKFDYVFGDLTDIPISDTPTGEI-WDFIRTILESSFQ----VLKPDGKFMT

Apis mellifera AHMIEEG--RQFDYVFGDLTDIPISPTPHGDA-WDFIRLILNSSMK----VLKSTGKYMT

S. purpuratus QQYIADG--KMFDYIINDLTAIPITTEPRGSQ-WDFLHLILDLSMQ----LLKPTGKYFT

N. vectensis NKCINKG--KLFDYVINDLTAIPITTEARGDQ-WDFLQLILDLSMK----VLSPTGKYFT

M. brevicollis DTLQAQGM--QFDYIINDLTAVPVSTSLA-----GSDWEFLQIVLNRAMPLLAPHGKYLT

A. thaliana RKSPEG----KYDAIIVDSSD-PVGPALA-----LVEKPFFETLAR----ALKPGGVLCN

Saccharomyces cerevisiae QDIGASDVHKKFDVIITDSSD-PEGPAEA-----FFQERYFELLKD----ALNPNGVVIM

* * * * *

Homo sapiens QG-NCVNLTEALSLYEEQLGRLYCPVEFSKEIVCVPSYLELWVFYTVWKK-AKP------

Gallus gallus QG-NCINLTDALTLYEEQLSRLYCPVEFSKEIVCVPSYMELWVFYTIWKKQTEV------

Danio rerio QG-NCVNLTDALSEYEKLLGRLSCKVDFSKEVVCVPSYMELWVFYTIWKK----------

Drosophila melanogaster HG-NGSTCKVQLRLFEEQLNLLRPKVKFTTTKAFVPSFMEEWLFYQV-------TFA---

Anopheles gambiae HG-NGVSCPESLRMYEDQLAKLTPKVTYTKSSAFVPSFMEEWVFYQVQREVANATESV--

Apis mellifera HG-NGASCPQSLEMYEQVLSQLCVPVTFTKDSAFVPSFFEDWVFYQVSLK----------

S. purpuratus QG-NGFNNKSALAMYEEQLGKLKYKVSFSKENICVPSYLEMWVFYEIWKEAET-------

N. vectensis QG-NSFNKLDSLTMFEGQLKKLECPVEFSKETVCVPSYHELWVFYEIWKTNKS-------

M. brevicollis QG-GSANMPAATRCYEDIVHAHPAGLDFTKESVCVPSFHEMWLFYTLWKTN---------

A. thaliana MA-ESMWLHTHLIEDMISICRQTFK-SVHYAWSSVPTYPSGVIGFVLCSTEGPAVDFKNP

Saccharomyces cerevisiae QSSENFWLNLKYLHDLKNTAKKVFP-NTEYCYTMVPTYTSGQLGLIVCSNNANI-----P

**

Homo sapiens ----------------------------------------------

Gallus gallus ----------------------------------------------

Danio rerio ----------------------------------------------

Drosophila melanogaster ----------------------------------------------

Anopheles gambiae ----------------------------------------------

Apis mellifera ----------------------------------------------

S. purpuratus ----------------------------------------------

N. vectensis ----------------------------------------------

M. brevicollis ----------------------------------------------

A. thaliana IN-PIEKLDGAMTHKRELKFYNSDMHRAAFALPTFLRREVASLLAS

Saccharomyces cerevisiae LNIPQRKI--SEQEQGKLKYYNPQIHSSAFVLPTWADKVINE----
