# Supplementary material for: Enhancing Human Spermine Synthase Activity by Engineered Mutations
Source: PLoS Comput Biol. 2013 Feb 28;9(2):e1002924. doi: 10.1371/journal.pcbi.1002924 (PMC3585406; doi:10.1371/journal.pcbi.1002924)
Supplement: Table S3 — MSA among 500 homologous proteins. (DOCX) [file pcbi.1002924.s004.docx]

**Table S3**: MSA among 500 homologous proteins

lcl|84191 ------------------------------------------------------------

gi|168177377 ------------------------------------------------------------

gi|21264341 ------------------------------------------------------------

gi|791051 ------------------------------------------------------------

gi|332860397 ------------------------------------------------------------

gi|119619392 ------------------------------------------------------------

gi|402909680 ------------------------------------------------------------

gi|332864823 ------------------------------------------------------------

gi|354474372 ------------------------------------------------------------

gi|291407209 ------------------------------------------------------------

gi|297303473 ------------------------------------------------------------

gi|403263885 ------------------------------------------------------------

gi|395753759 ------------------------------------------------------------

gi|355704663 ------------------------------------------------------------

gi|4164136 ------------------------------------------------------------

gi|149030940 ------------------------------------------------------------

gi|76559933 ------------------------------------------------------------

gi|78369282 ------------------------------------------------------------

gi|118600998 ------------------------------------------------------------

gi|345806962 ------------------------------------------------------------

gi|154425706 ------------------------------------------------------------

gi|26340516 ------------------------------------------------------------

gi|384948950 ------------------------------------------------------------

gi|397497639 ------------------------------------------------------------

gi|348561339 ------------------------------------------------------------

gi|354473432 ------------------------------------------------------------

gi|344288661 MAGAPPDPARGAAIGDARRGAGAHVSTTSAASPRPPSGGARPRHTLTRVMRLRLKLQPME

gi|338728955 ------------------------------------------------------------

gi|71895235 ------------------------------------------------------------

gi|297678474 ------------------------------------------------------------

gi|301756254 ------------------------------------------------------------

gi|296470508 ------------------------------------------------------------

gi|351712477 ------------------------------------------------------------

gi|344242683 ------------------------------------------------------------

gi|387018788 ------------------------------------------------------------

gi|327268286 ------------------------------------------------------------

gi|334329503 MEY--PCVRNSKDNATESKNLSRELVGPLAAIAKATAITAAPRTLSLRIPVISSGMKTLE

gi|350595577 ------------------------------------------------------------

gi|351706018 ------------------------------------------------------------

gi|395518817 ------------------------------------------------------------

gi|149042406 ------------------------------------------------------------

gi|326913546 ------------------------------------------------------------

gi|224042691 ------------------------------------------------------------

gi|148708890 ------------------------------------------------------------

gi|345327044 ------------------------------------------------------------

gi|385862226 ------------------------------------------------------------

gi|354482601 ------------------------------------------------------------

gi|281352943 ------------------------------------------------------------

gi|18858203 ------------------------------------------------------------

gi|395838143 ------------------------------------------------------------

gi|213511272 ------------------------------------------------------------

gi|348518728 ------------------------------------------------------------

gi|386643030 ------------------------------------------------------------

gi|402909682 ------------------------------------------------------------

gi|4007861 ------------------------------------------------------------

gi|351706975 ------------------------------------------------------------

gi|4007046 ------------------------------------------------------------

gi|114687978 ------------------------------------------------------------

gi|2916761 ------------------------------------------------------------

gi|194373711 ------------------------------------------------------------

gi|332860399 ------------------------------------------------------------

gi|125978661 ------------------------------------------------------------

gi|225581217 ------------------------------------------------------------

gi|195376249 ------------------------------------------------------------

gi|195126140 ------------------------------------------------------------

gi|194747133 ------------------------------------------------------------

gi|195012869 ------------------------------------------------------------

gi|24663238 ------------------------------------------------------------

gi|194869781 ------------------------------------------------------------

gi|195327091 ------------------------------------------------------------

gi|390176333 ------------------------------------------------------------

gi|21358269 ------------------------------------------------------------

gi|74211624 ------------------------------------------------------------

gi|195589784 ------------------------------------------------------------

gi|289742457 ------------------------------------------------------------

gi|157118336 ------------------------------------------------------------

gi|158293995 ------------------------------------------------------------

gi|170032109 ------------------------------------------------------------

gi|327290877 ------------------------------------------------------------

gi|4927220 ------------------------------------------------------------

gi|195493801 ------------------------------------------------------------

gi|242024750 ------------------------------------------------------------

gi|312372613 ------------------------------------------------------------

gi|119619391 ------------------------------------------------------------

gi|260818384 ------------------------------------------------------------

gi|340709405 ------------------------------------------------------------

gi|350407163 ------------------------------------------------------------

gi|383856563 ------------------------------------------------------------

gi|357613964 ------------------------------------------------------------

gi|156542873 ------------------------------------------------------------

gi|332025522 ------------------------------------------------------------

gi|110750077 ------------------------------------------------------------

gi|91085293 ------------------------------------------------------------

gi|307188353 ------------------------------------------------------------

gi|390338494 ------------------------------------------------------------

gi|195440770 ------------------------------------------------------------

gi|380023489 ------------------------------------------------------------

gi|156394332 ------------------------------------------------------------

gi|167538084 ------------------------------------------------------------

gi|326435685 ------------------------------------------------------------

gi|47227186 ------------------------------------------------------------

gi|321461882 ------------------------------------------------------------

gi|344241635 ------------------------------------------------------------

gi|389613513 ------------------------------------------------------------

gi|313219992 ------------------------------------------------------------

gi|313225743 ------------------------------------------------------------

gi|198418193 ------------------------------------------------------------

gi|391343942 ------------------------------------------------------------

gi|196014283 ------------------------------------------------------------

gi|307204802 ------------------------------------------------------------

gi|225718096 ------------------------------------------------------------

gi|324096424 ------------------------------------------------------------

gi|327352193 --------------MSNRLQWKRRNTQTDIIQYHIIPRYTAPVPRNRSATSLFFCFIFFL

gi|226293104 ------------------------------------------------------------

gi|154277382 ------------------------------------------------------------

gi|212532035 ------------------------------------------------------------

gi|242117904 ------------------------------------------------------------

gi|134077286 ------------------------------------------------------------

gi|348570938 ----------------M-------------------------------------------

gi|389628860 ------------------------------------------------------------

gi|119495240 ------------------------------------------------------------

gi|67516811 ------------------------------------------------------------

gi|242774578 ------------------------------------------------------------

gi|225683737 ------------------------------------------------------------

gi|255716758 ------------------------------------------------------------

gi|395218408 ----------------MN--ALGRHILVEFYDCSPELMNDVMHIENSMVAAAETAGATVI

gi|348514634 ------------------------------------------------------------

gi|347828643 ------------------------------------------------------------

gi|361131873 ------------------------------------------------------------

gi|392864754 --------------MTALLSKGSRLPCHLAGQFFFLNR---PVPHPQPSGAILGFS----

gi|115492019 ------------------------------------------------------------

gi|212532039 ------------------------------------------------------------

gi|340914898 ------------------------------------------------------------

gi|156044358 ------------------------------------------------------------

gi|342887616 ------------------------------------------------------------

gi|302307177 ------------------------------------------------------------

gi|367038823 ------------------------------------------------------------

gi|358371985 ------------------------------------------------------------

gi|317030551 ------------------------------------------------------------

gi|303318439 --------------MTALLSQGSRLPCHLAGQFFFLNR---PVPHPQPSGAILRFF----

gi|85100119 ------------------------------------------------------------

gi|380088740 ------------------------------------------------------------

gi|395840954 ----------------M-------------------------------------------

gi|16758208 ----------------M-------------------------------------------

gi|327287238 ----------------M-------------------------------------------

gi|146414243 ------------------------------------------------------------

gi|190348490 ------------------------------------------------------------

gi|367025809 ------------------------------------------------------------

gi|121701283 ------------------------------------------------------------

gi|119592082 ----------------M-------------------------------------------

gi|70995928 ------------------------------------------------------------

gi|6678131 ----------------M-------------------------------------------

gi|531202 ----------------M-------------------------------------------

gi|114553926 ----------------M-------------------------------------------

gi|63253298 ----------------M-------------------------------------------

gi|122921244 --------------GSM-------------------------------------------

gi|116197943 ------------------------------------------------------------

gi|378733877 ------------------------------------------------------------

gi|26390475 ----------------M-------------------------------------------

gi|402852915 ----------------M-------------------------------------------

gi|347447556 --------------GHM-------------------------------------------

gi|383873318 ----------------M-------------------------------------------

gi|213409616 ------------------------------------------------------------

gi|32766339 ------------------------------------------------------------

gi|387515006 ------------------------------------------------------------

gi|46137467 ------------------------------------------------------------

gi|320583104 ------------------------------------------------------------

gi|350585594 ----------------M-------------------------------------------

gi|315053415 ------------------------------------------------------------

gi|322706886 ------------------------------------------------------------

gi|402082530 ------------------------------------------------------------

gi|317137159 ------------------------------------------------------------

gi|147902014 ------------------------------------------------------------

gi|344283521 ----------------M-------------------------------------------

gi|76637216 ----------------M-------------------------------------------

gi|255937259 ------------------------------------------------------------

gi|366998257 ------------------------------------------------------------

gi|320588782 ------------------------------------------------------------

gi|254580735 ------------------------------------------------------------

gi|301620173 ---------------ML-------------------------------------------

gi|258572568 ------------------------------------------------------------

gi|225710012 ------------------------------------------------------------

gi|19112807 ------------------------------------------------------------

gi|398411809 ------------------------------------------------------------

gi|379728455 -----------------------------------------------MVNAALEAQATVI

gi|149239971 ------------------------------------------------------------

gi|119175541 ------------------------------------------------------------

gi|320039089 ------------------------------------------------------------

gi|74138635 ----------------M-------------------------------------------

gi|238489149 ------------------------------------------------------------

gi|255725052 ------------------------------------------------------------

gi|322701141 ------------------------------------------------------------

gi|359378073 ------------------------------------------------------------

gi|358378881 ------------------------------------------------------------

gi|50286095 ------------------------------------------------------------

gi|358391288 ------------------------------------------------------------

gi|401623242 ------------------------------------------------------------

gi|6325326 ------------------------------------------------------------

gi|151942852 ------------------------------------------------------------

gi|302912624 ------------------------------------------------------------

gi|261190484 ------------------------------------------------------------

gi|336262713 ------------------------------------------------------------

gi|296814308 ------------------------------------------------------------

gi|126138914 ------------------------------------------------------------

gi|50420767 ------------------------------------------------------------

gi|68482416 ------------------------------------------------------------

gi|169615146 ------------------------------------------------------------

gi|396485345 ------------------------------------------------------------

gi|241951762 ------------------------------------------------------------

gi|400597126 ------------------------------------------------------------

gi|345561897 ------------------------------------------------------------

gi|310793583 ------------------------------------------------------------

gi|340520373 ------------------------------------------------------------

gi|47228825 ------------------------------------------------------------

gi|156843587 ------------------------------------------------------------

gi|330907629 ------------------------------------------------------------

gi|189207669 ------------------------------------------------------------

gi|50554613 ------------------------------------------------------------

gi|375086054 ------------------------------------------------------------

gi|366996717 ------------------------------------------------------------

gi|380353232 ------------------------------------------------------------

gi|154304795 ------------------------------------------------------------

gi|171693823 ------------------------------------------------------------

gi|260781328 ------------------------------------------------------------

gi|344234284 ------------------------------------------------------------

gi|260951089 ------------------------------------------------------------

gi|367012209 ------------------------------------------------------------

gi|403214093 ------------------------------------------------------------

gi|354548134 ------------------------------------------------------------

gi|372463636 ------------------------------------------------------------

gi|284929129 ----------------MN--SLGRHILVEFFGCSSEILNNVSVIESSMLVAAQEAGATVI

gi|291533315 ------------------------------------------------------------

gi|384501417 ------------------------------------------------------------

gi|401838282 ------------------------------------------------------------

gi|344302668 ------------------------------------------------------------

gi|365982711 ------------------------------------------------------------

gi|296421657 ------------------------------------------------------------

gi|149695532 ------------------------------------------------------------

gi|50307673 ------------------------------------------------------------

gi|50303991 ------------------------------------------------------------

gi|380483699 ------------------------------------------------------------

gi|325660851 ------------------------------------------------------------

gi|160932658 ------------------------------------------------------------

gi|363751094 ------------------------------------------------------------

gi|254569166 ------------------------------------------------------------

gi|268612001 ------------------------------------------------------------

gi|225018465 ------------------------------------------------------------

gi|326427880 ------------------------------------------------------------

gi|297617720 ------------------------------------------------------------

gi|295665965 ------------------------------------------------------------

gi|317131721 ------------------------------------------------------------

gi|346467789 ------------------------------------------------------------

gi|328766899 ------------------------------------------------------------

gi|323486581 ------------------------------------------------------------

gi|326476682 ------------------------------------------------------------

gi|164660446 ------------------------------------------------------------

gi|169351543 ------------------------------------------------------------

gi|346326304 ------------------------------------------------------------

gi|167519370 ------------------------------------------------------------

gi|321468222 ------------------------------------------------------------

gi|404329717 ------------------------------------------------------------

gi|283798449 ------------------------------------------------------------

gi|300121366 ------------------------------------------------------------

gi|227112962 ------------------------------------------------------------

gi|388582960 ------------------------------------------------------------

gi|225713606 ------------------------------------------------------------

gi|346466755 ------------------------------------------------------------

gi|355720872 ------------------------------------------------------------

gi|167755034 ------------------------------------------------------------

gi|193215178 ----------------MSGKSLGKHILVEFYDCTPEVLDDVIHIERSMVKAAEIANATII

gi|403059603 ------------------------------------------------------------

gi|388854692 ------------------------------------------------------------

gi|227329706 ------------------------------------------------------------

gi|253689498 ------------------------------------------------------------

gi|300715359 ------------------------------------------------------------

gi|343427335 ------------------------------------------------------------

gi|1167999 --------------MKR-------------------------------------------

gi|261369006 ------------------------------------------------------------

gi|327405610 ----------------MSN-ALGNHILVEFMGCDPHIMNDVSSIERDMVDAALKAGATVI

gi|254573952 ------------------------------------------------------------

gi|251788647 ------------------------------------------------------------

gi|327309372 ------------------------------------------------------------

gi|313896579 ------------------------------------------------------------

gi|342871552 ------------------------------------------------------------

gi|295101693 ------------------------------------------------------------

gi|302665792 ------------------------------------------------------------

gi|71023471 ------------------------------------------------------------

gi|50122255 ------------------------------------------------------------

gi|320583760 ------------------------------------------------------------

gi|328351802 ------------------------------------------------------------

gi|331082383 ------------------------------------------------------------

gi|320529049 ------------------------------------------------------------

gi|255658393 ------------------------------------------------------------

gi|302500029 ------------------------------------------------------------

gi|307132173 ------------------------------------------------------------

gi|357057706 ------------------------------------------------------------

gi|271501669 ------------------------------------------------------------

gi|348689589 ------------------------------------------------------------

gi|387510768 ------------------------------------------------------------

gi|260588048 ------------------------------------------------------------

gi|160943874 ------------------------------------------------------------

gi|188532978 ------------------------------------------------------------

gi|145362282 --------------MIFSVV---RSSLPYIFRFTSHQNHH---LSQTLVPPLSHFSEIFT

gi|394757460 ------------------------------------------------------------

gi|397614365 ------------------------------------------------------------

gi|170025697 ------------------------------------------------------------

gi|401624651 ------------------------------------------------------------

gi|51595070 ------------------------------------------------------------

gi|257439175 ------------------------------------------------------------

gi|238926770 ------------------------------------------------------------

gi|392423567 ------------------------------------------------------------

gi|151941307 ------------------------------------------------------------

gi|270263031 ------------------------------------------------------------

gi|153813543 ------------------------------------------------------------

gi|21957501 ------------------------------------------------------------

gi|325184913 ------------------------------------------------------------

gi|292670864 ------------------------------------------------------------

gi|108808904 ------------------------------------------------------------

gi|154623414 ------------------------------------------------------------

gi|259148132 ------------------------------------------------------------

gi|402833951 ------------------------------------------------------------

gi|242240254 ------------------------------------------------------------

gi|196007934 ------------------------------------------------------------

gi|391628126 ------------------------------------------------------------

gi|323308143 ------------------------------------------------------------

gi|377578674 ------------------------------------------------------------

gi|402570735 ------------------------------------------------------------

gi|355378207 ------------------------------------------------------------

gi|401565378 ------------------------------------------------------------

gi|6323175 ------------------------------------------------------------

gi|354559941 ------------------------------------------------------------

gi|359463278 -------MLLPFSSKPMN--SLGRHILVEFYGGSAEVLNDVLLIETNMLRAAKESGATII

gi|295108885 ------------------------------------------------------------

gi|158319435 ------------------------------------------------------------

gi|334128073 ------------------------------------------------------------

gi|312162112 --------------MA--------------------ENNN---NSSS-------------

gi|313114511 ------------------------------------------------------------

gi|349579866 ------------------------------------------------------------

gi|116785514 --------------MA--------------------ENNN---NSNS-------------

gi|333929035 ------------------------------------------------------------

gi|260887129 ------------------------------------------------------------

gi|386825267 ------------------------------------------------------------

gi|37524842 ------------------------------------------------------------

gi|198427208 ------------------------------------------------------------

gi|291545477 ------------------------------------------------------------

gi|182418090 ------------------------------------------------------------

gi|259907494 ------------------------------------------------------------

gi|291549279 ------------------------------------------------------------

gi|157372235 ------------------------------------------------------------

gi|297845432 --------------MICSVL---RSSLPYIFSFTSHQNHH---LSSKTLIPLSHFSEIFT

gi|12230014 ------------------------------------------------------------

gi|170032648 ------------------------------------------------------------

gi|198453209 ------------------------------------------------------------

gi|284049217 ------------------------------------------------------------

gi|238754470 ------------------------------------------------------------

gi|257126567 ------------------------------------------------------------

gi|385785731 ------------------------------------------------------------

gi|238759889 ------------------------------------------------------------

gi|238750530 ------------------------------------------------------------

gi|6094336 ------------------------------------------------------------

gi|332160447 ------------------------------------------------------------

gi|377851579 ------------------------------------------------------------

gi|388391237 ------------------------------------------------------------

gi|91209189 ------------------------------------------------------------

gi|169837854 ------------------------------------------------------------

gi|317046947 ------------------------------------------------------------

gi|256273016 ------------------------------------------------------------

gi|218547577 ------------------------------------------------------------

gi|374579091 ------------------------------------------------------------

gi|373245798 ------------------------------------------------------------

gi|387605598 ------------------------------------------------------------

gi|187935464 ------------------------------------------------------------

gi|384470785 ------------------------------------------------------------

gi|329298049 ------------------------------------------------------------

gi|110803121 ------------------------------------------------------------

gi|212639268 ------------------------------------------------------------

gi|20807781 ------------------------------------------------------------

gi|253988276 ------------------------------------------------------------

gi|315294649 ------------------------------------------------------------

gi|383191575 ------------------------------------------------------------

gi|386228114 ------------------------------------------------------------

gi|330009512 ------------------------------------------------------------

gi|296086541 ------------------------------------------------------------

gi|392297662 ------------------------------------------------------------

gi|292489273 ------------------------------------------------------------

gi|225424695 --------------MAE--H---T------------------------------------

gi|24111564 ------------------------------------------------------------

gi|194433428 ------------------------------------------------------------

gi|333978511 ------------------------------------------------------------

gi|378194398 ------------------------------------------------------------

gi|238791204 ------------------------------------------------------------

gi|297841771 -------------------------------------------MSST-------------

gi|15799805 ------------------------------------------------------------

gi|110799842 ------------------------------------------------------------

gi|324112468 ------------------------------------------------------------

gi|260890661 ------------------------------------------------------------

gi|401840422 ------------------------------------------------------------

gi|312970216 ------------------------------------------------------------

gi|16128114 ------------------------------------------------------------

gi|386248282 ------------------------------------------------------------

gi|366159436 ------------------------------------------------------------

gi|323975716 ------------------------------------------------------------

gi|384503184 --------------MAE--E---SGVVVSV------------------------------

gi|18309532 ------------------------------------------------------------

gi|372276039 ------------------------------------------------------------

gi|333009123 ------------------------------------------------------------

gi|332097889 ------------------------------------------------------------

gi|381405644 ------------------------------------------------------------

gi|303325156 ------------------------------------------------------------

gi|377919674 ------------------------------------------------------------

gi|300905534 ------------------------------------------------------------

gi|15223115 -------------------------------------------MSST-------------

gi|145336078 --------------MIFSVV---RSSLPYIFRFTSHQNHH---LSQTLVPPLSHFSEIFT

gi|206578746 ------------------------------------------------------------

gi|42524959 ----------------MK--ALGRHILVEFSGCNAEVLNDVSIIERSMVEAAQIAGATVI

gi|323157811 ------------------------------------------------------------

gi|391275496 ------------------------------------------------------------

gi|195446427 ------------------------------------------------------------

gi|251778321 ------------------------------------------------------------

gi|152968708 ------------------------------------------------------------

gi|371605278 ------------------------------------------------------------

gi|345395892 ------------------------------------------------------------

gi|385302583 ------------------------------------------------------------

gi|386206487 ------------------------------------------------------------

gi|238761600 ------------------------------------------------------------

gi|194746211 ------------------------------------------------------------

gi|331226164 ------------------------------------------------------------

gi|340742010 ------------------------------------------------------------

gi|50552163 ------------------------------------------------------------

gi|238786761 ------------------------------------------------------------

gi|386033187 ------------------------------------------------------------

gi|6094327 ------------------------------------------------------------

gi|82775410 ------------------------------------------------------------

gi|110762382 ------------------------------------------------------------

gi|333011508 ------------------------------------------------------------

gi|322834377 ------------------------------------------------------------

gi|374992883 ------------------------------------------------------------

gi|308185706 ------------------------------------------------------------

gi|157147448 ------------------------------------------------------------

gi|12229958 --------------MAA-PE---NT--LH-------------------------------

gi|33340515 ------------------------------------------------------------

gi|295098625 ------------------------------------------------------------

gi|380021423 ------------------------------------------------------------

gi|238797200 ------------------------------------------------------------

gi|293394516 ------------------------------------------------------------

gi|99083515 --------------MAA--E---ND--VV-------------------------------

gi|388608380 ------------------------------------------------------------

gi|320173653 ------------------------------------------------------------

gi|161612515 ------------------------------------------------------------

gi|378073834 ------------------------------------------------------------

gi|161504706 ------------------------------------------------------------

gi|195499508 ------------------------------------------------------------

gi|2821961 ------------------------------------------------------------

gi|359376594 ------------------------------------------------------------

gi|374297740 ------------------------------------------------------------

gi|239827059 ------------------------------------------------------------

gi|403383804 ------------------------------------------------------------

gi|304396607 ------------------------------------------------------------

gi|397166809 ------------------------------------------------------------

gi|123441066 ------------------------------------------------------------

gi|82542724 ------------------------------------------------------------

gi|12229957 --------------MAA--E---ST---V-------------------------------

gi|376394468 ------------------------------------------------------------

gi|218687999 ------------------------------------------------------------

gi|170768493 ------------------------------------------------------------

gi|365156921 ------------------------------------------------------------

gi|195330342 ------------------------------------------------------------

gi|340356265 ------------------------------------------------------------

gi|388336724 ------------------------------------------------------------

gi|188589068 ------------------------------------------------------------

gi|376400744 ------------------------------------------------------------

gi|28317069 ------------------------------------------------------------

gi|389842198 ------------------------------------------------------------

gi|260596552 ------------------------------------------------------------

gi|6094335 --------------MAD--A---V------------------------------------

gi|365988413 ------------------------------------------------------------

gi|194446066 ------------------------------------------------------------

gi|350536257 --------------MAD--E---CAAFMK-------------------------------

gi|357485917 --------------MAAAPE---NTTLLE-------------------------------

gi|296127808 ------------------------------------------------------------

gi|74310739 ------------------------------------------------------------

gi|323166003 ------------------------------------------------------------

gi|126153731 --------------VAA-------------------------------------------

gi|291280945 ------------------------------------------------------------

gi|49425361 --------------MAA--E---L------------------------------------

gi|334341918 ------------------------------------------------------------

gi|187731639 ------------------------------------------------------------

gi|156935354 ------------------------------------------------------------

gi|376377905 ------------------------------------------------------------

gi|392391837 ------------------------------------------------------------

gi|331645238 ------------------------------------------------------------

gi|378578181 ------------------------------------------------------------

gi|24645443 ------------------------------------------------------------

gi|335578432 ------------------------------------------------------------

gi|374383060 ------------------------------------------------------------

gi|358248320 --------------MAA--E---NV--VE-------------------------------

gi|300919677 ------------------------------------------------------------

gi|194902914 ------------------------------------------------------------

gi|226492509 --------------MAWR-A---NPKPSQAEGKRGCERGA---PSHYPAAASPHTPPSRA

gi|391290786 ------------------------------------------------------------

lcl|84191 ------------------------------------------------------------

gi|168177377 ------------------------------------------------------------

gi|21264341 ------------------------------------------------------------

gi|791051 ------------------------------------------------------------

gi|332860397 ------------------------------------------------------------

gi|119619392 ------------------------------------------------------------

gi|402909680 ------------------------------------------------------------

gi|332864823 ------------------------------------------------------------

gi|354474372 ------------------------------------------------------------

gi|291407209 -----------------------------------------MRWSC-MSVRECLFKCSPV

gi|297303473 ------------------------------------------------------------

gi|403263885 -----------------------------------------MAGAASLSLAQSAAIGDAR

gi|395753759 ------------------------------------------------------------

gi|355704663 ------------------------------------------------------------

gi|4164136 ------------------------------------------------------------

gi|149030940 ------------------------------------------------------------

gi|76559933 ------------------------------------------------------------

gi|78369282 ------------------------------------------------------------

gi|118600998 ------------------------------------------------------------

gi|345806962 ------------------------------------------------------------

gi|154425706 ------------------------------------------------------------

gi|26340516 ------------------------------------------------------------

gi|384948950 ------------------------------------------------------------

gi|397497639 ------------------------------------------------------------

gi|348561339 ---------------------------------------MLFESPAGGFLSGYRARADPP

gi|354473432 ------------------------------------------------------------

gi|344288661 REGARRPPSLRPGGGPGGAPRGRTNARGAGGAPAGGPRLQVPFRAATGGAWGGAPWGPPS

gi|338728955 ------------------------------------------------------------

gi|71895235 ------------------------------------------------------------

gi|297678474 ------------------------------------------------------------

gi|301756254 -------------------------------------MYLKYASGVGLTAEKQVGPCEEV

gi|296470508 ------------------------------------------------------------

gi|351712477 ------------------------------------------------------------

gi|344242683 ------------------------------------------------------------

gi|387018788 ------------------------------------------------------------

gi|327268286 ------------------------------------------------------------

gi|334329503 L--SKQAKRTRQCSLTHTRALTQTHAH-THALTVAHAAEASKQAAAAAAASTRHGTGLYL

gi|350595577 ------------------------------------------------------------

gi|351706018 ------------------------------------------------------------

gi|395518817 ------------------------------------------------------------

gi|149042406 ------------------------------------------------------------

gi|326913546 ------------------------------------------------------------

gi|224042691 ------------------------------------------------------------

gi|148708890 ------------------------------------------------------------

gi|345327044 -------------------------------------MKAPTNSLSEDASGGGGGFWERG

gi|385862226 ------------------------------------------------------------

gi|354482601 ------------------------------------------------------------

gi|281352943 ------------------------------------------------------------

gi|18858203 ------------------------------------------------------------

gi|395838143 ------------------------------------------------------------

gi|213511272 ------------------------------------------------------------

gi|348518728 ------------------------------------------------------------

gi|386643030 ------------------------------------------------------------

gi|402909682 ------------------------------------------------------------

gi|4007861 ------------------------------------------------------------

gi|351706975 ------------------------------------------------------------

gi|4007046 ------------------------------------------------------------

gi|114687978 ------------------------------------------------------------

gi|2916761 ------------------------------------------------------------

gi|194373711 ------------------------------------------------------------

gi|332860399 ------------------------------------------------------------

gi|125978661 ------------------------------------------------------------

gi|225581217 ------------------------------------------------------------

gi|195376249 ------------------------------------------------------------

gi|195126140 ------------------------------------------------------------

gi|194747133 ------------------------------------------------------------

gi|195012869 ------------------------------------------------------------

gi|24663238 ------------------------------------------------------------

gi|194869781 ------------------------------------------------------------

gi|195327091 ------------------------------------------------------------

gi|390176333 ------------------------------------------------------------

gi|21358269 ------------------------------------------------------------

gi|74211624 ------------------------------------------------------------

gi|195589784 ------------------------------------------------------------

gi|289742457 ------------------------------------------------------------

gi|157118336 ------------------------------------------------------------

gi|158293995 ------------------------------------------------------------

gi|170032109 ------------------------------------------------------------

gi|327290877 ------------------------------------------------------------

gi|4927220 ------------------------------------------------------------

gi|195493801 ------------------------------------------------------------

gi|242024750 ------------------------------------------------------------

gi|312372613 ------------------------------------------------------------

gi|119619391 ------------------------------------------------------------

gi|260818384 ------------------------------------------------------------

gi|340709405 ------------------------------------------------------------

gi|350407163 ------------------------------------------------------------

gi|383856563 ------------------------------------------------------------

gi|357613964 ------------------------------------------------------------

gi|156542873 ------------------------------------------------------------

gi|332025522 ------------------------------------------------------------

gi|110750077 ------------------------------------------------------------

gi|91085293 ------------------------------------------------------------

gi|307188353 ------------------------------------------------------------

gi|390338494 ------------------------------------------------------------

gi|195440770 ------------------------------------------------------------

gi|380023489 ------------------------------------------------------------

gi|156394332 ------------------------------------------------------------

gi|167538084 ------------------------------------------------------------

gi|326435685 ------------------------------------------------------------

gi|47227186 ------------------------------------------------------------

gi|321461882 ------------------------------------------------------------

gi|344241635 ------------------------------------------------------------

gi|389613513 ------------------------------------------------------------

gi|313219992 ------------------------------------------------------------

gi|313225743 ------------------------------------------------------------

gi|198418193 ------------------------------------------------------------

gi|391343942 ------------------------------------------------------------

gi|196014283 ------------------------------------------------------------

gi|307204802 ------------------------------------------------------------

gi|225718096 ------------------------------------------------------------

gi|324096424 ------------------------------------------------------------

gi|327352193 FLLRMPS---HGRRPLSLRLYKPPFRTSCSSRLLSC------TPSIFRPLTT-----LQK

gi|226293104 ------------------------------------------------------------

gi|154277382 ------------------------------------------------------------

gi|212532035 ----MIP---RCLLSFRGNSVTRFSCHQLTPLL-RS------FPRNKSTFT------TTT

gi|242117904 --------------------------------------------------------M---

gi|134077286 ------------------------------------------------------------

gi|348570938 ------------------------ESGLDGPT-----------------AP----GP---

gi|389628860 ------------MS----------------------------------------------

gi|119495240 ------------------------------------------------------------

gi|67516811 ------------------------------------------------------------

gi|242774578 ------------------------------------------------------------

gi|225683737 ------------------------------------------------------------

gi|255716758 ------------------------------------------------------------

gi|395218408 NSTFHHF---SPYGVSGVVVIQESHLAIHTWPEYGY------AAVDLFTCGDSVDPWVSY

gi|348514634 --------------------------------------------------------M---

gi|347828643 ------------MSEI-------------THP----------------------------

gi|361131873 ------------MSDI-------------THP----------------------------

gi|392864754 -------------------------------HTLSQ------APLSRQTTPT--------

gi|115492019 ------------------------------------------------------------

gi|212532039 ----MIP---RCLLSFRGNSVTRFSCHQLTPLL-RS------FPRNKSTFT------TTT

gi|340914898 ------------MSSE--------------------------------------------

gi|156044358 ------------MSEI-------------THP----------------------------

gi|342887616 ----------------------------------------------------------MS

gi|302307177 ------------------------------------------------------------

gi|367038823 ------------M-----------------------------------------------

gi|358371985 ------------------------------------------------------------

gi|317030551 ------------------------------------------------------------

gi|303318439 -------------------------------HTLSQ------APLSRQTTPT--------

gi|85100119 ------------MS----------------------------------------------

gi|380088740 ------------MS----------------------------------------------

gi|395840954 ------------------------ESGPDGPD-----------------PS----GP---

gi|16758208 ------------------------EPGPDGPA-----------------AP----GP---

gi|327287238 ------------------------EPG----------------------LE----GA---

gi|146414243 ---------------MFCYASLFISLFLFHSPSSFR------QELSFLHQHHFKNFQQQS

gi|190348490 ---------------MFCYASLFISLFLFHSPSSFR------QELSFSHQHHFKNFQQQS

gi|367025809 ------------M-----------------------------------------------

gi|121701283 ------------------------------------------------------------

gi|119592082 ------------------------EPGPDGPA-----------------AS----GP---

gi|70995928 ------------------------------------------------------------

gi|6678131 ------------------------EPGPDGPA-----------------AP----GP---

gi|531202 ------------------------EPGPDGPA-----------------AS----GP---

gi|114553926 ------------------------EPGPDGPA-----------------AS----GP---

gi|63253298 ------------------------EPGPDGPA-----------------AS----GP---

gi|122921244 ------------------------EPGPDGPA-----------------AS----GP---

gi|116197943 ------------M-----------------------------------------------

gi|378733877 ------------------------------------------------------------

gi|26390475 ------------------------EPGPDGPA-----------------AP----GP---

gi|402852915 ------------------------EPGPDGPA-----------------AS----GP---

gi|347447556 ------------------------EPGPDGPA-----------------AS----GP---

gi|383873318 ------------------------EPGPDGPA-----------------AS----GP---

gi|213409616 ----------------------------------------------------------MS

gi|32766339 --------------------------------------------------------M---

gi|387515006 ------------------------------------------------------------

gi|46137467 ----------------------------------------------------------MS

gi|320583104 ------------------------------------------------------------

gi|350585594 ------------------------EPGPDGPA-----------------AP----GP---

gi|315053415 ------------------------------------------------------------

gi|322706886 ------------MKKLLSSQLQRSFTSPLFASSAFL------VPRFCTPYPI-SRYSNKF

gi|402082530 ------------MS----------------------------------------------

gi|317137159 ------------------------------------------------------------

gi|147902014 -------------------------------------------------ME----KP---

gi|344283521 ------------------------DSGPDGPV-----------------AP----GP---

gi|76637216 ------------------------EPGPDGPA-----------------AP----GP---

gi|255937259 ------------------------------------------------------------

gi|366998257 ------------------------------------------------------------

gi|320588782 ------------MS----------------------------------------------

gi|254580735 ------------------------------------------------------------

gi|301620173 ------------------------RAFCRGAN-----------------ME----KP---

gi|258572568 ------------------------------------------------------------

gi|225710012 ------------------------------------------------------------

gi|19112807 ----------------------------------------------------------MS

gi|398411809 ------------------------------------------------------------

gi|379728455 NATFHHF---SPYGVSGVVVIQESHLAIHTWPEYGY------AAVDLFTCGDEVNPWISY

gi|149239971 ------------------------------------------------------------

gi|119175541 ------------------------------------------------------------

gi|320039089 ------------------------------------------------------------

gi|74138635 ------------------------EPGPDGPA-----------------AP----GP---

gi|238489149 ----MNP---HLARASSSLSLSSLLFSSFSSSLSRR------LPSRAFRHTLFRSHFSHS

gi|255725052 ------------------------------------------------------------

gi|322701141 ------------------------------------------------------------

gi|359378073 ------------------------------------------------------------

gi|358378881 ------------MS----------------------------------------------

gi|50286095 ------------------------------------------------------------

gi|358391288 ------------MS----------------------------------------------

gi|401623242 ------------------------------------------------------------

gi|6325326 ------------------------------------------------------------

gi|151942852 ------------------------------------------------------------

gi|302912624 ----------------------------------------------------------MS

gi|261190484 ------------------------------------------------------------

gi|336262713 ------------MS----------------------------------------------

gi|296814308 ------------------------------------------------------------

gi|126138914 ------------------------------------------------------------

gi|50420767 ------------------------------------------------------------

gi|68482416 ------------------------------------------------------------

gi|169615146 ------------------------------------------------------------

gi|396485345 ------------------------------------------------------------

gi|241951762 ------------------------------------------------------------

gi|400597126 ------------------------------------------------------------

gi|345561897 ------------------------------------------------------------

gi|310793583 ------------MS----------------------------------------------

gi|340520373 ------------MS----------------------------------------------

gi|47228825 --------------------------------------------------------M---

gi|156843587 ------------------------------------------------------------

gi|330907629 ------------------------------------------------------------

gi|189207669 ------------------------------------------------------------

gi|50554613 ------------------------------------------------------------

gi|375086054 ------------------------------------------------------------

gi|366996717 ------------------------------------------------------------

gi|380353232 ------------------------------------------------------------

gi|154304795 ------------MSEY-------------FHSILLF------FPDIGVFYEG-HDRN---

gi|171693823 ------------MG----------------------------------------------

gi|260781328 ------------------------------------------------------------

gi|344234284 ------------------------------------------------------------

gi|260951089 ------------------------------------------------------------

gi|367012209 ------------------------------------------------------------

gi|403214093 ------------------------------------------------------------

gi|354548134 ------------------------------------------------------------

gi|372463636 ------------------------------------------------------------

gi|284929129 NSTFHHF---SPFGVSGVVVIQESHLAIHTWPEYRY------AAVDLFTCGDTVNPWISF

gi|291533315 ------------------------------------------------------------

gi|384501417 ------------------------------------------------------------

gi|401838282 ------------------------------------------------------------

gi|344302668 ------------------------------------------------------------

gi|365982711 ------------------------------------------------------------

gi|296421657 ------------------------------------------------------------

gi|149695532 ------------------------------------------------------------

gi|50307673 -----------------------------------------------MSS----K-----

gi|50303991 ------------------------------------------------------------

gi|380483699 ------------------------------------------------------------

gi|325660851 ------------------------------------------------------------

gi|160932658 ------------------------------------------------------------

gi|363751094 ----------------------------------------------------------MS

gi|254569166 ------------------------------------------------------------

gi|268612001 ------------------------------------------------------------

gi|225018465 ------------------------------------------------------------

gi|326427880 ------------------------------------------------------------

gi|297617720 ------------------------------------------------------------

gi|295665965 ----MSS---RSLPSLTSFLYIPSSSCSYIPRL-----------RCLSTFVL-RPPIQQI

gi|317131721 ------------------------------------------------------------

gi|346467789 ------------------------------------------------------------

gi|328766899 -----------------------------------------------------------M

gi|323486581 ------------------------------------------------------------

gi|326476682 ------------------------------------------------------------

gi|164660446 ------------------------------------------------------------

gi|169351543 ------------------------------------------------------------

gi|346326304 ------------------------------------------------------------

gi|167519370 ------------------------------------------------------------

gi|321468222 ------------------------------------------------------------

gi|404329717 ------------------------------------------------------------

gi|283798449 ------------------------------------------------------------

gi|300121366 ------------------------------------------------------------

gi|227112962 ------------------------------------------------------------

gi|388582960 ------------------------------------------------------------

gi|225713606 ------------------------------------------------------------

gi|346466755 ------------------------------------------------------------

gi|355720872 ------------------------------------------------------------

gi|167755034 ------------------------------------------------------------

gi|193215178 NTGFHHF---SPYGVSGVVVIQESHLTIHTWPEYGY------ASVDIFSCGDTVDPWAAY

gi|403059603 ------------------------------------------------------------

gi|388854692 ------------------------------------------------------------

gi|227329706 ------------------------------------------------------------

gi|253689498 ------------------------------------------------------------

gi|300715359 ------------------------------------------------------------

gi|343427335 ------------------------------------------------------------

gi|1167999 ------------------------EFRRTTGP-----------------LP----GP---

gi|261369006 ------------------------------------------------------------

gi|327405610 NSTFHHF---SPYGVSGVVVIQESHLAIHTWPEYGY------AAVDLFTCGE-MDAWISF

gi|254573952 -----------------------------------------------MSEIS--K-----

gi|251788647 ------------------------------------------------------------

gi|327309372 ------------------------------------------------------------

gi|313896579 ------------------------------------------------------------

gi|342871552 ----------------------------------------------------------MS

gi|295101693 ------------------------------------------------------------

gi|302665792 ------------------------------------------------------------

gi|71023471 ------------------------------------------------------------

gi|50122255 ------------------------------------------------------------

gi|320583760 -----------------------------------------------MPL----------

gi|328351802 ------------------------------------------------------------

gi|331082383 ------------------------------------------------------------

gi|320529049 ------------------------------------------------------------

gi|255658393 ------------------------------------------------------------

gi|302500029 ------------------------------------------------------------

gi|307132173 ------------------------------------------------------------

gi|357057706 ------------------------------------------------------------

gi|271501669 ------------------------------------------------------------

gi|348689589 ------------------------------------------------------------

gi|387510768 -----------------------------------------------MTQ---------E

gi|260588048 ------------------------------------------------------------

gi|160943874 ------------------------------------------------------------

gi|188532978 ------------------------------------------------------------

gi|145362282 RAITMDA---KETSATDL--KRPREEDDNGGA-------------ATMETE---NGD-QK

gi|394757460 ------------------------------------------------------------

gi|397614365 ------------------------------------------------------------

gi|170025697 ------------------------------------------------------------

gi|401624651 -----------------------------------------------MVKSS--N-----

gi|51595070 ------------------------------------------------------------

gi|257439175 ------------------------------------------------------------

gi|238926770 ------------------------------------------------------------

gi|392423567 ------------------------------------------------------------

gi|151941307 -----------------------------------------------MVNNS--Q-----

gi|270263031 ------------------------------------------------------------

gi|153813543 ------------------------------------------------------------

gi|21957501 ------------------------------------------------------------

gi|325184913 ------------------------------------------------------------

gi|292670864 ------------------------------------------------------------

gi|108808904 ------------------------------------------------------------

gi|154623414 ----MDP-----CSATDL--KRPREEDANSAA-------------AS-------NGD-PT

gi|259148132 -----------------------------------------------MVNNS--Q-----

gi|402833951 ------------------------------------------------------------

gi|242240254 ------------------------------------------------------------

gi|196007934 -------------------------MSQKTES-----------------TQ----LP---

gi|391628126 ------------------------------------------------------------

gi|323308143 -----------------------------------------------MVNNS--Q-----

gi|377578674 ------------------------------------------------------------

gi|402570735 ------------------------------------------------------------

gi|355378207 ------------------------------------------------------------

gi|401565378 ------------------------------------------------------------

gi|6323175 -----------------------------------------------MVNNS--Q-----

gi|354559941 ------------------------------------------------------------

gi|359463278 TSQFHRF---PPIGVSGFIVLQESHFAIHTWPEYGY------AALDLFTCGMSVNPWIAY

gi|295108885 ------------------------------------------------------------

gi|158319435 ------------------------------------------------------------

gi|334128073 ------------------------------------------------------------

gi|312162112 ------------NNGFDLPIKRVRPQEEGEDHPLSC------VD--AMGGV---DGG---

gi|313114511 ------------------------------------------------------------

gi|349579866 -----------------------------------------------MVNNS--Q-----

gi|116785514 ------------NNGCDLPIKRVRPQEEEED---------------AMDGV---DGG---

gi|333929035 ------------------------------------------------------------

gi|260887129 ------------------------------------------------------------

gi|386825267 ------------------------------------------------------------

gi|37524842 ------------------------------------------------------------

gi|198427208 -----------------------------------------------------------M

gi|291545477 ------------------------------------------------------------

gi|182418090 ------------------------------------------------------------

gi|259907494 ------------------------------------------------------------

gi|291549279 ------------------------------------------------------------

gi|157372235 ------------------------------------------------------------

gi|297845432 RALTMDA---KESSATDL--KRPREEDDNGAA-------------ATMETE---NGD-QK

gi|12230014 ----MDA---KETSATDL--KRPREEDDNGGA-------------ATMETE---NGD-QK

gi|170032648 -----------------------------------------------MNP----------

gi|198453209 ------------------------------------------------------------

gi|284049217 ------------------------------------------------------------

gi|238754470 ------------------------------------------------------------

gi|257126567 ------------------------------------------------------------

gi|385785731 ------------------------------------------------------------

gi|238759889 ------------------------------------------------------------

gi|238750530 ------------------------------------------------------------

gi|6094336 -------------------------------M------------EAANHNN---NGCTNE

gi|332160447 ------------------------------------------------------------

gi|377851579 ------------------------------------------------------------

gi|388391237 ------------------------------------------------------------

gi|91209189 ------------------------------------------------------------

gi|169837854 ------------------------------------------------------------

gi|317046947 ------------------------------------------------------------

gi|256273016 -----------------------------------------------MVNNS--Q-----

gi|218547577 ------------------------------------------------------------

gi|374579091 ------------------------------------------------------------

gi|373245798 ------------------------------------------------------------

gi|387605598 ------------------------------------------------------------

gi|187935464 ------------------------------------------------------------

gi|384470785 ------------------------------------------------------------

gi|329298049 ------------------------------------------------------------

gi|110803121 ------------------------------------------------------------

gi|212639268 ------------------------------------------------------------

gi|20807781 ------------------------------------------------------------

gi|253988276 ------------------------------------------------------------

gi|315294649 ------------------------------------------------------------

gi|383191575 ------------------------------------------------------------

gi|386228114 ------------------------------------------------------------

gi|330009512 ------------------------------------------------------------

gi|296086541 ------------------------------------------------------------

gi|392297662 -----------------------------------------------MVNNS--Q-----

gi|292489273 ------------------------------------------------------------

gi|225424695 -----------------------REGE-----------------------E---NGVSGC

gi|24111564 ------------------------------------------------------------

gi|194433428 ------------------------------------------------------------

gi|333978511 ------------------------------------------------------------

gi|378194398 ------------------------------------------------------------

gi|238791204 ------------------------------------------------------------

gi|297841771 ----------QEASVTDLPVKRPREAEEDNNG-------------AAMETE---NGGGEI

gi|15799805 ------------------------------------------------------------

gi|110799842 ------------------------------------------------------------

gi|324112468 ------------------------------------------------------------

gi|260890661 ------------------------------------------------------------

gi|401840422 -----------------------------------------------MFNNS--N-----

gi|312970216 ------------------------------------------------------------

gi|16128114 ------------------------------------------------------------

gi|386248282 ------------------------------------------------------------

gi|366159436 ------------------------------------------------------------

gi|323975716 ------------------------------------------------------------

gi|384503184 --------------PTDFPVKRPSEGEDN----------------GVVSVE---MEDTNN

gi|18309532 ------------------------------------------------------------

gi|372276039 ------------------------------------------------------------

gi|333009123 ------------------------------------------------------------

gi|332097889 ------------------------------------------------------------

gi|381405644 ------------------------------------------------------------

gi|303325156 ------------------------------------------------------------

gi|377919674 ------------------------------------------------------------

gi|300905534 ------------------------------------------------------------

gi|15223115 ----------QEASVTDLPVKRPREAEEDNNG-------------GAMETE---NGGGEI

gi|145336078 RAITMDA---KETSATDL--KRPREEDDNGGA-------------ATMETE---NGD-QK

gi|206578746 ------------------------------------------------------------

gi|42524959 NSTFHHF---SPWGVSGVVVIQESHLAIHTWPEYRY------AAVDLFTCGDSVDPWVSF

gi|323157811 ------------------------------------------------------------

gi|391275496 ------------------------------------------------------------

gi|195446427 ------------------------------------------------------------

gi|251778321 ------------------------------------------------------------

gi|152968708 ------------------------------------------------------------

gi|371605278 ------------------------------------------------------------

gi|345395892 ------------------------------------------------------------

gi|385302583 ------------------------------------------------------------

gi|386206487 ------------------------------------------------------------

gi|238761600 ------------------------------------------------------------

gi|194746211 ------------------------------------------------------------

gi|331226164 ------------------------------------------------------------

gi|340742010 ------------------------------------------------------------

gi|50552163 -----------------------------------------------MTVKQ--LE----

gi|238786761 ------------------------------------------------------------

gi|386033187 ------------------------------------------------------------

gi|6094327 -------------------------------M------------EEANNKE---------

gi|82775410 ------------------------------------------------------------

gi|110762382 ------------------------------------------------------------

gi|333011508 ------------------------------------------------------------

gi|322834377 ------------------------------------------------------------

gi|374992883 ------------------------------------------------------------

gi|308185706 ------------------------------------------------------------

gi|157147448 ------------------------------------------------------------

gi|12229958 --------------STDSPLKRQREDEV------NG------VSDTL----------SKE

gi|33340515 -------------------------------M------------EAANHNND--NGCTNE

gi|295098625 ------------------------------------------------------------

gi|380021423 ------------------------------------------------------------

gi|238797200 ------------------------------------------------------------

gi|293394516 ------------------------------------------------------------

gi|99083515 --------------SSDLHVKRQRDEEE------NG------VSVSTVSMDM--EG-CKD

gi|388608380 ------------------------------------------------------------

gi|320173653 ------------------------------------------------------------

gi|161612515 ------------------------------------------------------------

gi|378073834 ------------------------------------------------------------

gi|161504706 ------------------------------------------------------------

gi|195499508 ------------------------------------------------------------

gi|2821961 --------------------TRP-------------------------------------

gi|359376594 ------------------------------------------------------------

gi|374297740 ------------------------------------------------------------

gi|239827059 ------------------------------------------------------------

gi|403383804 ------------------------------------------------------------

gi|304396607 ------------------------------------------------------------

gi|397166809 ------------------------------------------------------------

gi|123441066 ------------------------------------------------------------

gi|82542724 ------------------------------------------------------------

gi|12229957 --------------ELESSMKEHRDDDEKS----NG------FSVSAVSMDV--EGGDKD

gi|376394468 ------------------------------------------------------------

gi|218687999 ------------------------------------------------------------

gi|170768493 ------------------------------------------------------------

gi|365156921 ------------------------------------------------------------

gi|195330342 ------------------------------------------------------------

gi|340356265 ------------------------------------------------------------

gi|388336724 ------------------------------------------------------------

gi|188589068 ------------------------------------------------------------

gi|376400744 ------------------------------------------------------------

gi|28317069 -----------------------------------------------LFKRR--FECGIK

gi|389842198 ------------------------------------------------------------

gi|260596552 ------------------------------------------------------------

gi|6094335 -----------------------NSNSKE----------------EAAQLP---DGVS--

gi|365988413 -----------------------------------------------MTTAT--TEPPAK

gi|194446066 ------------------------------------------------------------

gi|350536257 --------------GTELPVKRPREEEAETEM------------EAANNSN---NGCEKE

gi|357485917 --------------STDFPLKRQREEEDEDQLPHNG------FSETV----------PKD

gi|296127808 ------------------------------------------------------------

gi|74310739 ------------------------------------------------------------

gi|323166003 ------------------------------------------------------------

gi|126153731 ----------------DLPVKRQREEEEEDQ--QNG------LSQND----------SST

gi|291280945 ------------------------------------------------------------

gi|49425361 -----------------------SLGSAA----------------SKPQTE---NGVSDS

gi|334341918 ------------------------------------------------------------

gi|187731639 ------------------------------------------------------------

gi|156935354 ------------------------------------------------------------

gi|376377905 ------------------------------------------------------------

gi|392391837 ------------------------------------------------------------

gi|331645238 ------------------------------------------------------------

gi|378578181 ------------------------------------------------------------

gi|24645443 ------------------------------------------------------------

gi|335578432 ------------------------------------------------------------

gi|374383060 ------------------------------------------------------------

gi|358248320 --------------FTDLPVKRPREEEEEEQT--NG------VSNDAVSTL------PND

gi|300919677 ------------------------------------------------------------

gi|194902914 ------------------------------------------------------------

gi|226492509 QGFGMEA---------DAAAKRTRESGEDATA----------------------------

gi|391290786 ------------------------------------------------------------

lcl|84191 -----------------------------------------------MGSSHHHHHHSSG

gi|168177377 -----------------------------------------------MGSSHHHHHHSSG

gi|21264341 ------------------------------------------------------------

gi|791051 ------------------------------------------------------------

gi|332860397 ------------------------------------------------------------

gi|119619392 ------------------------------------------------------------

gi|402909680 ------------------------------------------------------------

gi|332864823 ------------------------------------------------------------

gi|354474372 ------------------------------------------------------------

gi|291407209 R---ALEGMAMQTAIEVRPEAVAVWLR---------------------------------

gi|297303473 ------------------------------------------------------------

gi|403263885 RGXAALPGPGSPAALPRGLAAFALPLGESPGCRVAA-DPSWDEGCRDCGRTRRDPRGEPG

gi|395753759 ---------------------FALPARGVPGLPSAGGPPPWDEGCRVCGRARRDPRGEPG

gi|355704663 ------------------------------------------------------------

gi|4164136 ------------------------------------------------------------

gi|149030940 ------------------------------------------------------------

gi|76559933 ------------------------------------------------------------

gi|78369282 ------------------------------------------------------------

gi|118600998 ------------------------------------------------------------

gi|345806962 ------------------------------------------------------------

gi|154425706 ------------------------------------------------------------

gi|26340516 ------------------------------------------------------------

gi|384948950 ------------------------------------------------------------

gi|397497639 ------------------------------------------------------------

gi|348561339 LGLRCPSVLERARRDPRGTPGRGVGGRAS----FRGTLATFCRACG-----RVNFPSAPG

gi|354473432 ------------------------------------MARKCWNG-EGLAQPSSLPSLNSK

gi|344288661 VGSRPFRSPEGPAPPPAGLGRMEVPGSPPPRAEGPPPGTPLFAARG--SPPRPGGPGAAS

gi|338728955 -----------------------------------------------MVRPYPGPLLSNG

gi|71895235 ------------------------------------------------------------

gi|297678474 ------------------------------------------------------------

gi|301756254 RGGTFAGRCCLQRRIQAAEQDWLHHLQAQCKMKTRGPLFKIVENFK-KILASSEPAEINA

gi|296470508 ------------------------------------------------------------

gi|351712477 ------------------------------------------------------------

gi|344242683 ------------------------------------------------------------

gi|387018788 ------------------------------------------------------------

gi|327268286 ------------------------------------------------------------

gi|334329503 IMAAARHNTLDFQLCAKGGGGVWGGGRRLAGALRGQFQGVEQAGGPVLAACCRGHAHVAA

gi|350595577 -------------------------------MGAFSWKRPLHFG--------RPPRHSPA

gi|351706018 ------------------------------------------------------------

gi|395518817 ------------------------------------------------------------

gi|149042406 ------------------------------------------------------------

gi|326913546 ------------------------------------------------------------

gi|224042691 ------------------------------------------------------------

gi|148708890 ------------------------------------------------------------

gi|345327044 VHEVAMGQRSVLFSFEAGNLLEEEGSLESVELLERKEMTQGGSVAKMHSVALRRKVQLAG

gi|385862226 ------------------------------------------------------------

gi|354482601 ------------------------------------------------------------

gi|281352943 ------------------------------------------------------------

gi|18858203 ------------------------------------------------------------

gi|395838143 ------------------------------------------------------------

gi|213511272 ------------------------------------------------------------

gi|348518728 ------------------------------------------------------------

gi|386643030 ------------------------------------------------------------

gi|402909682 ------------------------------------------------------------

gi|4007861 ------------------------------------------------------------

gi|351706975 ------------------------------------------------------------

gi|4007046 ------------------------------------------------------------

gi|114687978 ------------------------------------------------------------

gi|2916761 ------------------------------------------------------------

gi|194373711 ------------------------------------------------------------

gi|332860399 ------------------------------------------------------------

gi|125978661 ------------------------------------------------------------

gi|225581217 ------------------------------------------------------------

gi|195376249 ------------------------------------------------------------

gi|195126140 ------------------------------------------------------------

gi|194747133 ------------------------------------------------------------

gi|195012869 ------------------------------------------------------------

gi|24663238 ------------------------------------------------------------

gi|194869781 ------------------------------------------------------------

gi|195327091 ------------------------------------------------------------

gi|390176333 ------------------------------------------------------------

gi|21358269 ------------------------------------------------------------

gi|74211624 ------------------------------------------------------------

gi|195589784 ------------------------------------------------------------

gi|289742457 ------------------------------------------------------------

gi|157118336 ------------------------------------------------------------

gi|158293995 ------------------------------------------------------------

gi|170032109 ------------------------------------------------------------

gi|327290877 ------------------------------------------------------------

gi|4927220 ------------------------------------------------------------

gi|195493801 ------------------------------------------------------------

gi|242024750 ------------------------------------------------------------

gi|312372613 ------------------------------------------------------------

gi|119619391 ------------------------------------------------------------

gi|260818384 ------------------------------------------------------------

gi|340709405 ------------------------------------------------------------

gi|350407163 ------------------------------------------------------------

gi|383856563 ------------------------------------------------------------

gi|357613964 ------------------------------------------------------------

gi|156542873 ------------------------------------------------------------

gi|332025522 ------------------------------------------------------------

gi|110750077 ------------------------------------------------------------

gi|91085293 ------------------------------------------------------------

gi|307188353 ------------------------------------------------------------

gi|390338494 ------------------------------------------------------------

gi|195440770 ------------------------------------------------------------

gi|380023489 ------------------------------------------------------------

gi|156394332 ------------------------------------------------------------

gi|167538084 ------------------------------------------------------------

gi|326435685 ------------------------------------------------------------

gi|47227186 ------------------------------------------------------------

gi|321461882 ------------------------------------------------------------

gi|344241635 ------------------------------------------------------------

gi|389613513 ------------------------------------------------------------

gi|313219992 ------------------------------------------------------------

gi|313225743 ------------------------------------------------------------

gi|198418193 ------------------------------------------------------------

gi|391343942 ------------------------------------------------------------

gi|196014283 ------------------------------------------------------------

gi|307204802 ------------------------------------------------------------

gi|225718096 ------------------------------------------------------------

gi|324096424 ------------------------------------------------------------

gi|327352193 RTMS---ELT--------------------------------------------------

gi|226293104 --MS---ELT--------------------------------------------------

gi|154277382 --MS---EPT--------------------------------------------------

gi|212532035 ITMS---EIT--------------------------------------------------

gi|242117904 ------------------------------------------------------------

gi|134077286 --MS---DIT--------------------------------------------------

gi|348570938 ------------------------------------------------------------

gi|389628860 ---G---EIT--------------------------------------------------

gi|119495240 --MS---EIT--------------------------------------------------

gi|67516811 --MS---EIT--------------------------------------------------

gi|242774578 --MS---EIT--------------------------------------------------

gi|225683737 --MS---ELT--------------------------------------------------

gi|255716758 --MS--QEIT--------------------------------------------------

gi|395218408 TYLKEAFQSG--------------------------------------------------

gi|348514634 ------------------------------------------------------------

gi|347828643 ------------------------------------------------------------

gi|361131873 ------------------------------------------------------------

gi|392864754 PTMT---EIT--------------------------------------------------

gi|115492019 --MS---DIT--------------------------------------------------

gi|212532039 ITMS---EIT--------------------------------------------------

gi|340914898 ---G---EIT--------------------------------------------------

gi|156044358 ------------------------------------------------------------

gi|342887616 SE-----EIT--------------------------------------------------

gi|302307177 --MS--DEIT--------------------------------------------------

gi|367038823 ---G---DIT--------------------------------------------------

gi|358371985 --MS---DIT--------------------------------------------------

gi|317030551 --MS---DIT--------------------------------------------------

gi|303318439 TTMT---EIT--------------------------------------------------

gi|85100119 -------EIA--------------------------------------------------

gi|380088740 -------EIT--------------------------------------------------

gi|395840954 ------------------------------------------------------------

gi|16758208 ------------------------------------------------------------

gi|327287238 ------------------------------------------------------------

gi|146414243 HTMS---ELS--------------------------------------------------

gi|190348490 HTMS---ELS--------------------------------------------------

gi|367025809 ---G---DIT--------------------------------------------------

gi|121701283 --MS---EIT--------------------------------------------------

gi|119592082 ------------------------------------------------------------

gi|70995928 --MS---EIT--------------------------------------------------

gi|6678131 ------------------------------------------------------------

gi|531202 ------------------------------------------------------------

gi|114553926 ------------------------------------------------------------

gi|63253298 ------------------------------------------------------------

gi|122921244 ------------------------------------------------------------

gi|116197943 ------------------------------------------------------------

gi|378733877 --MS---EIT--------------------------------------------------

gi|26390475 ------------------------------------------------------------

gi|402852915 ------------------------------------------------------------

gi|347447556 ------------------------------------------------------------

gi|383873318 ------------------------------------------------------------

gi|213409616 SESQ---ELT--------------------------------------------------

gi|32766339 ------------------------------------------------------------

gi|387515006 --MA--NQIT--------------------------------------------------

gi|46137467 NE-----EIT--------------------------------------------------

gi|320583104 --MS---ELS--------------------------------------------------

gi|350585594 ------------------------------------------------------------

gi|315053415 --MS---EIT--------------------------------------------------

gi|322706886 VKMS---EIV--------------------------------------------------

gi|402082530 ---D---EIV--------------------------------------------------

gi|317137159 --MS---EKT--------------------------------------------------

gi|147902014 ------------------------------------------------------------

gi|344283521 ------------------------------------------------------------

gi|76637216 ------------------------------------------------------------

gi|255937259 --MS---ELT--------------------------------------------------

gi|366998257 --MS--QEIT--------------------------------------------------

gi|320588782 ---N---EIT--------------------------------------------------

gi|254580735 --MS--QEIT--------------------------------------------------

gi|301620173 ------------------------------------------------------------

gi|258572568 --MT---EIT--------------------------------------------------

gi|225710012 ------------------------------------------------------------

gi|19112807 V--Q---ELS--------------------------------------------------

gi|398411809 --MA---EIT--------------------------------------------------

gi|379728455 DYLKTAFEAD--------------------------------------------------

gi|149239971 --MA--QELS--------------------------------------------------

gi|119175541 --MT---EIT--------------------------------------------------

gi|320039089 --MT---EIT--------------------------------------------------

gi|74138635 ------------------------------------------------------------

gi|238489149 ITMS---EKT--------------------------------------------------

gi|255725052 --MS--QELT--------------------------------------------------

gi|322701141 --MS---EIV--------------------------------------------------

gi|359378073 --MS--SELS--------------------------------------------------

gi|358378881 ---D---EIV--------------------------------------------------

gi|50286095 --MS---EIT--------------------------------------------------

gi|358391288 ---N---EIV--------------------------------------------------

gi|401623242 --MA--QEIT--------------------------------------------------

gi|6325326 --MA--QEIT--------------------------------------------------

gi|151942852 --MT--QEIT--------------------------------------------------

gi|302912624 SE-----EIT--------------------------------------------------

gi|261190484 ------------------------------------------------------------

gi|336262713 -------EIT--------------------------------------------------

gi|296814308 --MS---EIT--------------------------------------------------

gi|126138914 --MSASQELT--------------------------------------------------

gi|50420767 --MA--QELT--------------------------------------------------

gi|68482416 --MA--QELT--------------------------------------------------

gi|169615146 --MP---NIE--------------------------------------------------

gi|396485345 --MP---SIE--------------------------------------------------

gi|241951762 --MA--QELT--------------------------------------------------

gi|400597126 --MS---EIT--------------------------------------------------

gi|345561897 -MSE---ELT--------------------------------------------------

gi|310793583 -------EIV--------------------------------------------------

gi|340520373 ---G---EIV--------------------------------------------------

gi|47228825 ------------------------------------------------------------

gi|156843587 --MS--QEIT--------------------------------------------------

gi|330907629 --MP---SIE--------------------------------------------------

gi|189207669 --MP---SIE--------------------------------------------------

gi|50554613 --MT---ELT--------------------------------------------------

gi|375086054 ------------------------------------------------------------

gi|366996717 --MA--QEIT--------------------------------------------------

gi|380353232 --MS--EQLT--------------------------------------------------

gi|154304795 ------------------------------------------------------------

gi|171693823 -------DIT--------------------------------------------------

gi|260781328 ------------------------------------------------------------

gi|344234284 --MA--QELT--------------------------------------------------

gi|260951089 --MA--QELT--------------------------------------------------

gi|367012209 --MS-TQQIT--------------------------------------------------

gi|403214093 --MS-EQ-IT--------------------------------------------------

gi|354548134 --MS--EQLT--------------------------------------------------

gi|372463636 --MS-EQEIT--------------------------------------------------

gi|284929129 DRLKILFKAD--------------------------------------------------

gi|291533315 ------------------------------------------------------------

gi|384501417 --MA--APLS--------------------------------------------------

gi|401838282 --MA--QEIT--------------------------------------------------

gi|344302668 --MA--QELS--------------------------------------------------

gi|365982711 --MA-SQEIT--------------------------------------------------

gi|296421657 --MS---EAV--------------------------------------------------

gi|149695532 ------------------------------------------------------------

gi|50307673 ------------------------------------------------------------

gi|50303991 --MS---ELT--------------------------------------------------

gi|380483699 ------------------------------------------------------------

gi|325660851 ------------------------------------------------------------

gi|160932658 ------------------------------------------------------------

gi|363751094 QDAKESREIT--------------------------------------------------

gi|254569166 --MS---ELT--------------------------------------------------

gi|268612001 ------------------------------------------------------------

gi|225018465 ------------------------------------------------------------

gi|326427880 ------------------------------------------------------------

gi|297617720 --------MS--------------------------------------------------

gi|295665965 RRMS---ELT--------------------------------------------------

gi|317131721 ------------------------------------------------------------

gi|346467789 --VRRLASIQ--------------------------------------------------

gi|328766899 KHP---------------------------------------------------------

gi|323486581 ------------------------------------------------------------

gi|326476682 --MS---EIT--------------------------------------------------

gi|164660446 ------------------------------------------------------------

gi|169351543 ------------------------------------------------------------

gi|346326304 --MS---EIT--------------------------------------------------

gi|167519370 ---------M--------------------------------------------------

gi|321468222 ------------------------------------------------------------

gi|404329717 ------------------------------------------------------------

gi|283798449 ------------------------------------------------------------

gi|300121366 ------------------------------------------------------------

gi|227112962 ------------------------------------------------------------

gi|388582960 ------------------------------------------------------------

gi|225713606 ------------------------------------------------------------

gi|346466755 --FAVWHRFK--------------------------------------------------

gi|355720872 ------------------------------------------------------------

gi|167755034 ------------------------------------------------------------

gi|193215178 TSLKEALGAK--------------------------------------------------

gi|403059603 ------------------------------------------------------------

gi|388854692 ------------------------------------------------------------

gi|227329706 ------------------------------------------------------------

gi|253689498 ------------------------------------------------------------

gi|300715359 ------------------------------------------------------------

gi|343427335 ------------------------------------------------------------

gi|1167999 ------------------------------------------------------------

gi|261369006 ------------------------------------------------------------

gi|327405610 DYLKECFGAK--------------------------------------------------

gi|254573952 ---L-----T--------------------------------------------------

gi|251788647 ------------------------------------------------------------

gi|327309372 --MS---EIT--------------------------------------------------

gi|313896579 ------------------------------------------------------------

gi|342871552 SE-----ETT--------------------------------------------------

gi|295101693 ------------------------------------------------------------

gi|302665792 --MS---EIT--------------------------------------------------

gi|71023471 ------------------------------------------------------------

gi|50122255 ------------------------------------------------------------

gi|320583760 ---------T--------------------------------------------------

gi|328351802 ------------------------------------------------------------

gi|331082383 ------------------------------------------------------------

gi|320529049 ------------------------------------------------------------

gi|255658393 ------------------------------------------------------------

gi|302500029 --MS---EIT--------------------------------------------------

gi|307132173 ------------------------------------------------------------

gi|357057706 ------------------------------------------------------------

gi|271501669 ------------------------------------------------------------

gi|348689589 ------------------------------------------------------------

gi|387510768 DQLL-----S--------------------------------------------------

gi|260588048 ------------------------------------------------------------

gi|160943874 ------------------------------------------------------------

gi|188532978 ------------------------------------------------------------

gi|145362282 KEPA-----C--------------------------------------------------

gi|394757460 ------------------------------------------------------------

gi|397614365 --------ML--------------------------------------------------

gi|170025697 ------------------------------------------------------------

gi|401624651 ------------------------------------------------------------

gi|51595070 ------------------------------------------------------------

gi|257439175 ------------------------------------------------------------

gi|238926770 ------------------------------------------------------------

gi|392423567 ------------------------------------------------------------

gi|151941307 ------------------------------------------------------------

gi|270263031 ------------------------------------------------------------

gi|153813543 ------------------------------------------------------------

gi|21957501 ------------------------------------------------------------

gi|325184913 --------MA--------------------------------------------------

gi|292670864 ------------------------------------------------------------

gi|108808904 ------------------------------------------------------------

gi|154623414 KEPA-----C--------------------------------------------------

gi|259148132 ------------------------------------------------------------

gi|402833951 ------------------------------------------------------------

gi|242240254 ------------------------------------------------------------

gi|196007934 ------------------------------------------------------------

gi|391628126 ------------------------------------------------------------

gi|323308143 ------------------------------------------------------------

gi|377578674 ------------------------------------------------------------

gi|402570735 ------------------------------------------------------------

gi|355378207 ------------------------------------------------------------

gi|401565378 ------------------------------------------------------------

gi|6323175 ------------------------------------------------------------

gi|354559941 ------------------------------------------------------------

gi|359463278 EILKAAFQAD--------------------------------------------------

gi|295108885 ------------------------------------------------------------

gi|158319435 ------------------------------------------------------------

gi|334128073 ------------------------------------------------------------

gi|312162112 ------------------------------------------------------------

gi|313114511 ------------------------------------------------------------

gi|349579866 ------------------------------------------------------------

gi|116785514 ------------------------------------------------------------

gi|333929035 ------------------------------------------------------------

gi|260887129 ------------------------------------------------------------

gi|386825267 ------------------------------------------------------------

gi|37524842 ------------------------------------------------------------

gi|198427208 DEL---------------------------------------------------------

gi|291545477 ------------------------------------------------------------

gi|182418090 ------------------------------------------------------------

gi|259907494 ------------------------------------------------------------

gi|291549279 ------------------------------------------------------------

gi|157372235 ------------------------------------------------------------

gi|297845432 KEPA-----C--------------------------------------------------

gi|12230014 KEPA-----C--------------------------------------------------

gi|170032648 ------------------------------------------------------------

gi|198453209 -M----------------------------------------------------------

gi|284049217 ------------------------------------------------------------

gi|238754470 ------------------------------------------------------------

gi|257126567 ------------------------------------------------------------

gi|385785731 ------------------------------------------------------------

gi|238759889 ------------------------------------------------------------

gi|238750530 ------------------------------------------------------------

gi|6094336 --SP-----Y--------------------------------------------------

gi|332160447 ------------------------------------------------------------

gi|377851579 ------------------------------------------------------------

gi|388391237 ------------------------------------------------------------

gi|91209189 ------------------------------------------------------------

gi|169837854 ------------------------------------------------------------

gi|317046947 ------------------------------------------------------------

gi|256273016 ------------------------------------------------------------

gi|218547577 ------------------------------------------------------------

gi|374579091 ------------------------------------------------------------

gi|373245798 ------------------------------------------------------------

gi|387605598 ------------------------------------------------------------

gi|187935464 ------------------------------------------------------------

gi|384470785 ------------------------------------------------------------

gi|329298049 ------------------------------------------------------------

gi|110803121 ------------------------------------------------------------

gi|212639268 --------MT--------------------------------------------------

gi|20807781 ------------------------------------------------------------

gi|253988276 ------------------------------------------------------------

gi|315294649 ------------------------------------------------------------

gi|383191575 ------------------------------------------------------------

gi|386228114 ------------------------------------------------------------

gi|330009512 ------------------------------------------------------------

gi|296086541 ------------------------------------------------------------

gi|392297662 ------------------------------------------------------------

gi|292489273 ------------------------------------------------------------

gi|225424695 ------------------------------------------------------------

gi|24111564 ------------------------------------------------------------

gi|194433428 ------------------------------------------------------------

gi|333978511 ------------------------------------------------------------

gi|378194398 ------------------------------------------------------------

gi|238791204 ------------------------------------------------------------

gi|297841771 KEPS-----C--------------------------------------------------

gi|15799805 ------------------------------------------------------------

gi|110799842 ------------------------------------------------------------

gi|324112468 ------------------------------------------------------------

gi|260890661 ------------------------------------------------------------

gi|401840422 ------------------------------------------------------------

gi|312970216 ------------------------------------------------------------

gi|16128114 ------------------------------------------------------------

gi|386248282 ------------------------------------------------------------

gi|366159436 ------------------------------------------------------------

gi|323975716 ------------------------------------------------------------

gi|384503184 ------------------------------------------------------------

gi|18309532 ------------------------------------------------------------

gi|372276039 ------------------------------------------------------------

gi|333009123 ------------------------------------------------------------

gi|332097889 ------------------------------------------------------------

gi|381405644 ------------------------------------------------------------

gi|303325156 ------------------------------------------------------------

gi|377919674 ------------------------------------------------------------

gi|300905534 ------------------------------------------------------------

gi|15223115 KEPS-----C--------------------------------------------------

gi|145336078 KEPA-----C--------------------------------------------------

gi|206578746 ------------------------------------------------------------

gi|42524959 EHLKKAFQA---------------------------------------------------

gi|323157811 ------------------------------------------------------------

gi|391275496 ------------------------------------------------------------

gi|195446427 -M----------------------------------------------------------

gi|251778321 ------------------------------------------------------------

gi|152968708 ------------------------------------------------------------

gi|371605278 ------------------------------------------------------------

gi|345395892 ------------------------------------------------------------

gi|385302583 ------------------------------------------------------------

gi|386206487 ------------------------------------------------------------

gi|238761600 ------------------------------------------------------------

gi|194746211 -M----------------------------------------------------------

gi|331226164 ------------------------------------------------------------

gi|340742010 ------------------------------------------------------------

gi|50552163 ------------------------------------------------------------

gi|238786761 ------------------------------------------------------------

gi|386033187 ------------------------------------------------------------

gi|6094327 --SP-----Y--------------------------------------------------

gi|82775410 ------------------------------------------------------------

gi|110762382 ------------------------------------------------------------

gi|333011508 ------------------------------------------------------------

gi|322834377 ------------------------------------------------------------

gi|374992883 ------------------------------------------------------------

gi|308185706 ------------------------------------------------------------

gi|157147448 ------------------------------------------------------------

gi|12229958 PQPN-----G--------------------------------------------------

gi|33340515 --SP-----Y--------------------------------------------------

gi|295098625 ------------------------------------------------------------

gi|380021423 ------------------------------------------------------------

gi|238797200 ------------------------------------------------------------

gi|293394516 ------------------------------------------------------------

gi|99083515 SVHN-----G--------------------------------------------------

gi|388608380 ------------------------------------------------------------

gi|320173653 ------------------------------------------------------------

gi|161612515 ------------------------------------------------------------

gi|378073834 ------------------------------------------------------------

gi|161504706 ------------------------------------------------------------

gi|195499508 -M----------------------------------------------------------

gi|2821961 ------------------------------------------------------------

gi|359376594 ------------------------------------------------------------

gi|374297740 ------------------------------------------------------------

gi|239827059 ------------------------------------------------------------

gi|403383804 ------------------------------------------------------------

gi|304396607 ------------------------------------------------------------

gi|397166809 ------------------------------------------------------------

gi|123441066 ------------------------------------------------------------

gi|82542724 ------------------------------------------------------------

gi|12229957 PSGN-----G--------------------------------------------------

gi|376394468 ------------------------------------------------------------

gi|218687999 ------------------------------------------------------------

gi|170768493 ------------------------------------------------------------

gi|365156921 ------------------------------------------------------------

gi|195330342 -M----------------------------------------------------------

gi|340356265 ------------------------------------------------------------

gi|388336724 ------------------------------------------------------------

gi|188589068 ------------------------------------------------------------

gi|376400744 ------------------------------------------------------------

gi|28317069 QM----------------------------------------------------------

gi|389842198 ------------------------------------------------------------

gi|260596552 ------------------------------------------------------------

gi|6094335 ------------------------------------------------------------

gi|365988413 TTLP-----R--------------------------------------------------

gi|194446066 ------------------------------------------------------------

gi|350536257 ESSP-----Y--------------------------------------------------

gi|357485917 PQPN-----G--------------------------------------------------

gi|296127808 ------------------------------------------------------------

gi|74310739 ------------------------------------------------------------

gi|323166003 ------------------------------------------------------------

gi|126153731 PQ-------G--------------------------------------------------

gi|291280945 ------------------------------------------------------------

gi|49425361 ------------------------------------------------------------

gi|334341918 ------------------------------------------------------------

gi|187731639 ------------------------------------------------------------

gi|156935354 ------------------------------------------------------------

gi|376377905 ------------------------------------------------------------

gi|392391837 ------------------------------------------------------------

gi|331645238 ------------------------------------------------------------

gi|378578181 ------------------------------------------------------------

gi|24645443 -M----------------------------------------------------------

gi|335578432 ------------------------------------------------------------

gi|374383060 ------------------------------------------------------------

gi|358248320 PQYP-----G--------------------------------------------------

gi|300919677 ------------------------------------------------------------

gi|194902914 -M----------------------------------------------------------

gi|226492509 ---A-----G--------------------------------------------------

gi|391290786 ------------------------------------------------------------

lcl|84191 LVPRGSRH----------------------------------------------------

gi|168177377 LVPRGSRH----------------------------------------------------

gi|21264341 M--AAARH----------------------------------------------------

gi|791051 MPGAAARH----------------------------------------------------

gi|332860397 M--AAARH----------------------------------------------------

gi|119619392 M--AAARH----------------------------------------------------

gi|402909680 M--AAARH----------------------------------------------------

gi|332864823 M--AAARH----------------------------------------------------

gi|354474372 M--AAARH----------------------------------------------------

gi|291407209 -GVVIRCY----------------------------------------------------

gi|297303473 ------------------------------------------------------------

gi|403263885 LGACAAPG----------------------------------------------------

gi|395753759 LGACAERG----------------------------------------------------

gi|355704663 TI----------------------------------------------------------

gi|4164136 ------------------------------------------------------------

gi|149030940 M--AAARH----------------------------------------------------

gi|76559933 M--AAARH----------------------------------------------------

gi|78369282 M--AAARH----------------------------------------------------

gi|118600998 M--AAARH----------------------------------------------------

gi|345806962 M--AAARH----------------------------------------------------

gi|154425706 M--AAARH----------------------------------------------------

gi|26340516 M--AAARH----------------------------------------------------

gi|384948950 M--AAARH----------------------------------------------------

gi|397497639 ------------------------------------------------------------

gi|348561339 VSPGGARG----------------------------------------------------

gi|354473432 L-RELGSS----------------------------------------------------

gi|344288661 LPGAGGAP----------------------------------------------------

gi|338728955 C--GLLGA----------------------------------------------------

gi|71895235 ------------------------------------------------------------

gi|297678474 --MAAAGH----------------------------------------------------

gi|301756254 ISKTLTKV----------------------------------------------------

gi|296470508 M--AAARH----------------------------------------------------

gi|351712477 ------------------------------------------------------------

gi|344242683 M--AAARH----------------------------------------------------

gi|387018788 --MAAARH----------------------------------------------------

gi|327268286 --MAAARH----------------------------------------------------

gi|334329503 ATAAAFPHCLPCPALCRPVSSFPIANPPPLRVTPLYNIRWGWRVCISLHSPSSSPTLSAS

gi|350595577 LP-----S----------------------------------------------------

gi|351706018 --MAAVWH----------------------------------------------------

gi|395518817 ------------------------------------------------------------

gi|149042406 ------------------------------------------------------------

gi|326913546 ------------------------------------------------------------

gi|224042691 ------------------------------------------------------------

gi|148708890 ------------------------------------------------------------

gi|345327044 ESEFARNK----------------------------------------------------

gi|385862226 --MAAVQQ----------------------------------------------------

gi|354482601 --MAAARH----------------------------------------------------

gi|281352943 ------------------------------------------------------------

gi|18858203 ------------------------------------------------------------

gi|395838143 --MAAARH----------------------------------------------------

gi|213511272 ------------------------------------------------------------

gi|348518728 ------------------------------------------------------------

gi|386643030 --MAAARH----------------------------------------------------

gi|402909682 --MAAARH----------------------------------------------------

gi|4007861 ------------------------------------------------------------

gi|351706975 ------------------------------------------------------------

gi|4007046 ------------------------------------------------------------

gi|114687978 --MAAARH----------------------------------------------------

gi|2916761 --MAAARH----------------------------------------------------

gi|194373711 ------------------------------------------------------------

gi|332860399 ------------------------------------------------------------

gi|125978661 ------------------------------------------------------------

gi|225581217 ------------------------------------------------------------

gi|195376249 ------------------------------------------------------------

gi|195126140 ------------------------------------------------------------

gi|194747133 ------------------------------------------------------------

gi|195012869 ------------------------------------------------------------

gi|24663238 ------------------------------------------------------------

gi|194869781 ------------------------------------------------------------

gi|195327091 ------------------------------------------------------------

gi|390176333 ------------------------------------------------------------

gi|21358269 ------------------------------------------------------------

gi|74211624 --MAAARH----------------------------------------------------

gi|195589784 ------------------------------------------------------------

gi|289742457 ------------------------------------------------------------

gi|157118336 ------------------------------------------------------------

gi|158293995 ------------------------------------------------------------

gi|170032109 ------------------------------------------------------------

gi|327290877 ------------------------------------------------------------

gi|4927220 ------------------------------------------------------------

gi|195493801 ------------------------------------------------------------

gi|242024750 ------------------------------------------------------------

gi|312372613 ------------------------------------------------------------

gi|119619391 --MAAARH----------------------------------------------------

gi|260818384 ------------------------------------------------------------

gi|340709405 ------------------------------------------------------------

gi|350407163 ------------------------------------------------------------

gi|383856563 ------------------------------------------------------------

gi|357613964 ------------------------------------------------------------

gi|156542873 ------------------------------------------------------------

gi|332025522 ------------------------------------------------------------

gi|110750077 ------------------------------------------------------------

gi|91085293 ------------------------------------------------------------

gi|307188353 ------------------------------------------------------------

gi|390338494 ------------------------------------------------------------

gi|195440770 ------------------------------------------------------------

gi|380023489 ------------------------------------------------------------

gi|156394332 ------------------------------------------------------------

gi|167538084 ------------------------------------------------------------

gi|326435685 ------------------------------------------------------------

gi|47227186 ------------------------------------------------------------

gi|321461882 ------------------------------------------------------------

gi|344241635 ------------------------------------------------------------

gi|389613513 ------------------------------------------------------------

gi|313219992 ------------------------------------------------------------

gi|313225743 ------------------------------------------------------------

gi|198418193 ------------------------------------------------------------

gi|391343942 ------------------------------------------------------------

gi|196014283 ------------------------------------------------------------

gi|307204802 ------------------------------------------------------------

gi|225718096 ------------------------------------------------------------

gi|324096424 ------------------------------------------------------------

gi|327352193 -------HP---------------------------------------------------

gi|226293104 -------HP---------------------------------------------------

gi|154277382 -------HP---------------------------------------------------

gi|212532035 -------HP---------------------------------------------------

gi|242117904 --------D---------------------------------------------------

gi|134077286 -------HP---------------------------------------------------

gi|348570938 --------A---------------------------------------------------

gi|389628860 -------HE---------------------------------------------------

gi|119495240 -------HP---------------------------------------------------

gi|67516811 -------HP---------------------------------------------------

gi|242774578 -------HP---------------------------------------------------

gi|225683737 -------HP---------------------------------------------------

gi|255716758 -------HP---------------------------------------------------

gi|395218408 -------HG---------------------------------------------------

gi|348514634 --------D---------------------------------------------------

gi|347828643 ------------------------------------------------------------

gi|361131873 ------------------------------------------------------------

gi|392864754 -------HP---------------------------------------------------

gi|115492019 -------HP---------------------------------------------------

gi|212532039 -------HP---------------------------------------------------

gi|340914898 -------HP---------------------------------------------------

gi|156044358 ------------------------------------------------------------

gi|342887616 -------HP---------------------------------------------------

gi|302307177 -------HP---------------------------------------------------

gi|367038823 -------HP---------------------------------------------------

gi|358371985 -------HP---------------------------------------------------

gi|317030551 -------HP---------------------------------------------------

gi|303318439 -------HP---------------------------------------------------

gi|85100119 -------HP---------------------------------------------------

gi|380088740 -------HP---------------------------------------------------

gi|395840954 --------A---------------------------------------------------

gi|16758208 --------A---------------------------------------------------

gi|327287238 --------A---------------------------------------------------

gi|146414243 -------HP---------------------------------------------------

gi|190348490 -------HP---------------------------------------------------

gi|367025809 -------HA---------------------------------------------------

gi|121701283 -------HP---------------------------------------------------

gi|119592082 --------A---------------------------------------------------

gi|70995928 -------HP---------------------------------------------------

gi|6678131 --------A---------------------------------------------------

gi|531202 --------A---------------------------------------------------

gi|114553926 --------A---------------------------------------------------

gi|63253298 --------A---------------------------------------------------

gi|122921244 --------A---------------------------------------------------

gi|116197943 ------------------------------------------------------------

gi|378733877 -------HP---------------------------------------------------

gi|26390475 --------A---------------------------------------------------

gi|402852915 --------A---------------------------------------------------

gi|347447556 --------A---------------------------------------------------

gi|383873318 --------A---------------------------------------------------

gi|213409616 -------HP---------------------------------------------------

gi|32766339 --------D---------------------------------------------------

gi|387515006 -------HP---------------------------------------------------

gi|46137467 -------HP---------------------------------------------------

gi|320583104 -------HP---------------------------------------------------

gi|350585594 --------A---------------------------------------------------

gi|315053415 -------HP---------------------------------------------------

gi|322706886 -------HP---------------------------------------------------

gi|402082530 -------HE---------------------------------------------------

gi|317137159 -------HP---------------------------------------------------

gi|147902014 ------------------------------------------------------------

gi|344283521 --------A---------------------------------------------------

gi|76637216 --------A---------------------------------------------------

gi|255937259 -------HP---------------------------------------------------

gi|366998257 -------HP---------------------------------------------------

gi|320588782 -------HA---------------------------------------------------

gi|254580735 -------HP---------------------------------------------------

gi|301620173 ------------------------------------------------------------

gi|258572568 -------HP---------------------------------------------------

gi|225710012 ------------------------------------------------------------

gi|19112807 -------HP---------------------------------------------------

gi|398411809 -------HP---------------------------------------------------

gi|379728455 -------YG---------------------------------------------------

gi|149239971 -------HP---------------------------------------------------

gi|119175541 -------HP---------------------------------------------------

gi|320039089 -------HP---------------------------------------------------

gi|74138635 --------A---------------------------------------------------

gi|238489149 -------HP---------------------------------------------------

gi|255725052 -------HP---------------------------------------------------

gi|322701141 -------HP---------------------------------------------------

gi|359378073 -------HP---------------------------------------------------

gi|358378881 -------HP---------------------------------------------------

gi|50286095 -------HP---------------------------------------------------

gi|358391288 -------HP---------------------------------------------------

gi|401623242 -------HP---------------------------------------------------

gi|6325326 -------HP---------------------------------------------------

gi|151942852 -------HP---------------------------------------------------

gi|302912624 -------HE---------------------------------------------------

gi|261190484 ------------------------------------------------------------

gi|336262713 -------HP---------------------------------------------------

gi|296814308 -------HP---------------------------------------------------

gi|126138914 -------HP---------------------------------------------------

gi|50420767 -------HP---------------------------------------------------

gi|68482416 -------HP---------------------------------------------------

gi|169615146 -------HP---------------------------------------------------

gi|396485345 -------HP---------------------------------------------------

gi|241951762 -------HP---------------------------------------------------

gi|400597126 -------HP---------------------------------------------------

gi|345561897 -------HP---------------------------------------------------

gi|310793583 -------HP---------------------------------------------------

gi|340520373 -------HP---------------------------------------------------

gi|47228825 --------D---------------------------------------------------

gi|156843587 -------HP---------------------------------------------------

gi|330907629 -------HP---------------------------------------------------

gi|189207669 -------HP---------------------------------------------------

gi|50554613 -------HP---------------------------------------------------

gi|375086054 ------------------------------------------------------------

gi|366996717 -------HP---------------------------------------------------

gi|380353232 -------HP---------------------------------------------------

gi|154304795 ------------------------------------------------------------

gi|171693823 -------HS---------------------------------------------------

gi|260781328 ------------------------------------------------------------

gi|344234284 -------HP---------------------------------------------------

gi|260951089 -------HP---------------------------------------------------

gi|367012209 -------HP---------------------------------------------------

gi|403214093 -------HP---------------------------------------------------

gi|354548134 -------HP---------------------------------------------------

gi|372463636 -------HP---------------------------------------------------

gi|284929129 -------YG---------------------------------------------------

gi|291533315 ------------------------------------------------------------

gi|384501417 -------HP---------------------------------------------------

gi|401838282 -------HP---------------------------------------------------

gi|344302668 -------HP---------------------------------------------------

gi|365982711 -------HP---------------------------------------------------

gi|296421657 -------HP---------------------------------------------------

gi|149695532 ------------------------------------------------------------

gi|50307673 -------HP---------------------------------------------------

gi|50303991 -------HP---------------------------------------------------

gi|380483699 ------------------------------------------------------------

gi|325660851 ------------------------------------------------------------

gi|160932658 ------------------------------------------------------------

gi|363751094 -------HP---------------------------------------------------

gi|254569166 -------HP---------------------------------------------------

gi|268612001 ------------------------------------------------------------

gi|225018465 ------------------------------------------------------------

gi|326427880 -------MD---------------------------------------------------

gi|297617720 -------ED---------------------------------------------------

gi|295665965 -------HP---------------------------------------------------

gi|317131721 ------------------------------------------------------------

gi|346467789 -------RF---------------------------------------------------

gi|328766899 ------------------------------------------------------------

gi|323486581 ------------------------------------------------------------

gi|326476682 -------HP---------------------------------------------------

gi|164660446 ------------------------------------------------------------

gi|169351543 ------------------------------------------------------------

gi|346326304 -------HP---------------------------------------------------

gi|167519370 -------VD---------------------------------------------------

gi|321468222 -------MD---------------------------------------------------

gi|404329717 ------------------------------------------------------------

gi|283798449 ------------------------------------------------------------

gi|300121366 --------M---------------------------------------------------

gi|227112962 ------------------------------------------------------------

gi|388582960 ------------------------------------------------------------

gi|225713606 ------------------------------------------------------------

gi|346466755 -------RF---------------------------------------------------

gi|355720872 ------------------------------------------------------------

gi|167755034 ------------------------------------------------------------

gi|193215178 -------SG---------------------------------------------------

gi|403059603 ------------------------------------------------------------

gi|388854692 ------------------------------------------------------------

gi|227329706 ------------------------------------------------------------

gi|253689498 ------------------------------------------------------------

gi|300715359 ------------------------------------------------------------

gi|343427335 ------------------------------------------------------------

gi|1167999 --------A---------------------------------------------------

gi|261369006 ------------------------------------------------------------

gi|327405610 -------NY---------------------------------------------------

gi|254573952 -------HP---------------------------------------------------

gi|251788647 ------------------------------------------------------------

gi|327309372 -------HP---------------------------------------------------

gi|313896579 ------------------------------------------------------------

gi|342871552 -------HP---------------------------------------------------

gi|295101693 ------------------------------------------------------------

gi|302665792 -------HP---------------------------------------------------

gi|71023471 ------------------------------------------------------------

gi|50122255 ------------------------------------------------------------

gi|320583760 -------HP---------------------------------------------------

gi|328351802 ------------------------------------------------------------

gi|331082383 ------------------------------------------------------------

gi|320529049 ------------------------------------------------------------

gi|255658393 ------------------------------------------------------------

gi|302500029 -------HP---------------------------------------------------

gi|307132173 ------------------------------------------------------------

gi|357057706 ------------------------------------------------------------

gi|271501669 ------------------------------------------------------------

gi|348689589 --------M---------------------------------------------------

gi|387510768 -------HP---------------------------------------------------

gi|260588048 ------------------------------------------------------------

gi|160943874 ------------------------------------------------------------

gi|188532978 ------------------------------------------------------------

gi|145362282 -------FS---------------------------------------------------

gi|394757460 ------------------------------------------------------------

gi|397614365 -------GL---------------------------------------------------

gi|170025697 ------------------------------------------------------------

gi|401624651 -------HP---------------------------------------------------

gi|51595070 ------------------------------------------------------------

gi|257439175 ------------------------------------------------------------

gi|238926770 ------------------------------------------------------------

gi|392423567 ------------------------------------------------------------

gi|151941307 -------HP---------------------------------------------------

gi|270263031 ------------------------------------------------------------

gi|153813543 ------------------------------------------------------------

gi|21957501 ------------------------------------------------------------

gi|325184913 -------NL---------------------------------------------------

gi|292670864 ------------------------------------------------------------

gi|108808904 ------------------------------------------------------------

gi|154623414 -------FS---------------------------------------------------

gi|259148132 -------HP---------------------------------------------------

gi|402833951 ------------------------------------------------------------

gi|242240254 ------------------------------------------------------------

gi|196007934 --------A---------------------------------------------------

gi|391628126 ------------------------------------------------------------

gi|323308143 -------HP---------------------------------------------------

gi|377578674 ------------------------------------------------------------

gi|402570735 ------------------------------------------------------------

gi|355378207 ------------------------------------------------------------

gi|401565378 ------------------------------------------------------------

gi|6323175 -------HP---------------------------------------------------

gi|354559941 ------------------------------------------------------------

gi|359463278 -------HG---------------------------------------------------

gi|295108885 ------------------------------------------------------------

gi|158319435 ------------------------------------------------------------

gi|334128073 ------------------------------------------------------------

gi|312162112 -------YP---------------------------------------------------

gi|313114511 ------------------------------------------------------------

gi|349579866 -------HP---------------------------------------------------

gi|116785514 -------YP---------------------------------------------------

gi|333929035 ------------------------------------------------------------

gi|260887129 ------------------------------------------------------------

gi|386825267 ------------------------------------------------------------

gi|37524842 ------------------------------------------------------------

gi|198427208 ------------------------------------------------------------

gi|291545477 ------------------------------------------------------------

gi|182418090 ------------------------------------------------------------

gi|259907494 ------------------------------------------------------------

gi|291549279 ------------------------------------------------------------

gi|157372235 ------------------------------------------------------------

gi|297845432 -------FS---------------------------------------------------

gi|12230014 -------FS---------------------------------------------------

gi|170032648 ------------------------------------------------------------

gi|198453209 --------D---------------------------------------------------

gi|284049217 ------------------------------------------------------------

gi|238754470 ------------------------------------------------------------

gi|257126567 ------------------------------------------------------------

gi|385785731 ------------------------------------------------------------

gi|238759889 ------------------------------------------------------------

gi|238750530 ------------------------------------------------------------

gi|6094336 -------IS---------------------------------------------------

gi|332160447 ------------------------------------------------------------

gi|377851579 ------------------------------------------------------------

gi|388391237 ------------------------------------------------------------

gi|91209189 ------------------------------------------------------------

gi|169837854 ------------------------------------------------------------

gi|317046947 ------------------------------------------------------------

gi|256273016 -------HP---------------------------------------------------

gi|218547577 ------------------------------------------------------------

gi|374579091 ------------------------------------------------------------

gi|373245798 ------------------------------------------------------------

gi|387605598 ------------------------------------------------------------

gi|187935464 ------------------------------------------------------------

gi|384470785 ------------------------------------------------------------

gi|329298049 ------------------------------------------------------------

gi|110803121 ------------------------------------------------------------

gi|212639268 -------IA---------------------------------------------------

gi|20807781 ------------------------------------------------------------

gi|253988276 ------------------------------------------------------------

gi|315294649 ------------------------------------------------------------

gi|383191575 ------------------------------------------------------------

gi|386228114 ------------------------------------------------------------

gi|330009512 ------------------------------------------------------------

gi|296086541 -------MS---------------------------------------------------

gi|392297662 -------HS---------------------------------------------------

gi|292489273 ------------------------------------------------------------

gi|225424695 -------MS---------------------------------------------------

gi|24111564 ------------------------------------------------------------

gi|194433428 ------------------------------------------------------------

gi|333978511 ------------------------------------------------------------

gi|378194398 ------------------------------------------------------------

gi|238791204 ------------------------------------------------------------

gi|297841771 -------MS---------------------------------------------------

gi|15799805 ------------------------------------------------------------

gi|110799842 ------------------------------------------------------------

gi|324112468 ------------------------------------------------------------

gi|260890661 ------------------------------------------------------------

gi|401840422 -------HA---------------------------------------------------

gi|312970216 ------------------------------------------------------------

gi|16128114 ------------------------------------------------------------

gi|386248282 ------------------------------------------------------------

gi|366159436 ------------------------------------------------------------

gi|323975716 ------------------------------------------------------------

gi|384503184 -------IS---------------------------------------------------

gi|18309532 ------------------------------------------------------------

gi|372276039 ------------------------------------------------------------

gi|333009123 ------------------------------------------------------------

gi|332097889 ------------------------------------------------------------

gi|381405644 ------------------------------------------------------------

gi|303325156 ------------------------------------------------------------

gi|377919674 ------------------------------------------------------------

gi|300905534 ------------------------------------------------------------

gi|15223115 -------MS---------------------------------------------------

gi|145336078 -------FS---------------------------------------------------

gi|206578746 ------------------------------------------------------------

gi|42524959 -------NY---------------------------------------------------

gi|323157811 ------------------------------------------------------------

gi|391275496 ------------------------------------------------------------

gi|195446427 --------D---------------------------------------------------

gi|251778321 ------------------------------------------------------------

gi|152968708 ------------------------------------------------------------

gi|371605278 ------------------------------------------------------------

gi|345395892 ------------------------------------------------------------

gi|385302583 ------------------------------------------------------------

gi|386206487 ------------------------------------------------------------

gi|238761600 ------------------------------------------------------------

gi|194746211 --------D---------------------------------------------------

gi|331226164 ------------------------------------------------------------

gi|340742010 ------------------------------------------------------------

gi|50552163 -------HA---------------------------------------------------

gi|238786761 ------------------------------------------------------------

gi|386033187 ------------------------------------------------------------

gi|6094327 -------IS---------------------------------------------------

gi|82775410 ------------------------------------------------------------

gi|110762382 -------MD---------------------------------------------------

gi|333011508 ------------------------------------------------------------

gi|322834377 ------------------------------------------------------------

gi|374992883 ------------------------------------------------------------

gi|308185706 ------------------------------------------------------------

gi|157147448 ------------------------------------------------------------

gi|12229958 -------LS---------------------------------------------------

gi|33340515 -------IS---------------------------------------------------

gi|295098625 ------------------------------------------------------------

gi|380021423 -------MD---------------------------------------------------

gi|238797200 ------------------------------------------------------------

gi|293394516 ------------------------------------------------------------

gi|99083515 -------IS---------------------------------------------------

gi|388608380 ------------------------------------------------------------

gi|320173653 ------------------------------------------------------------

gi|161612515 ------------------------------------------------------------

gi|378073834 ------------------------------------------------------------

gi|161504706 ------------------------------------------------------------

gi|195499508 --------D---------------------------------------------------

gi|2821961 ------------------------------------------------------------

gi|359376594 ------------------------------------------------------------

gi|374297740 ------------------------------------------------------------

gi|239827059 ------------------------------------------------------------

gi|403383804 ------------------------------------------------------------

gi|304396607 ------------------------------------------------------------

gi|397166809 ------------------------------------------------------------

gi|123441066 ------------------------------------------------------------

gi|82542724 ------------------------------------------------------------

gi|12229957 -------VS---------------------------------------------------

gi|376394468 ------------------------------------------------------------

gi|218687999 ------------------------------------------------------------

gi|170768493 ------------------------------------------------------------

gi|365156921 ------------------------------------------------------------

gi|195330342 --------D---------------------------------------------------

gi|340356265 ------------------------------------------------------------

gi|388336724 ------------------------------------------------------------

gi|188589068 ------------------------------------------------------------

gi|376400744 ------------------------------------------------------------

gi|28317069 --------D---------------------------------------------------

gi|389842198 ------------------------------------------------------------

gi|260596552 ------------------------------------------------------------

gi|6094335 ------------------------------------------------------------

gi|365988413 -------HP---------------------------------------------------

gi|194446066 ------------------------------------------------------------

gi|350536257 -------IS---------------------------------------------------

gi|357485917 -------LS---------------------------------------------------

gi|296127808 ------------------------------------------------------------

gi|74310739 ------------------------------------------------------------

gi|323166003 ------------------------------------------------------------

gi|126153731 -------LS---------------------------------------------------

gi|291280945 ------------------------------------------------------------

gi|49425361 -------IS---------------------------------------------------

gi|334341918 ------------------------------------------------------------

gi|187731639 ------------------------------------------------------------

gi|156935354 ------------------------------------------------------------

gi|376377905 ------------------------------------------------------------

gi|392391837 ------------------------------------------------------------

gi|331645238 ------------------------------------------------------------

gi|378578181 ------------------------------------------------------------

gi|24645443 --------D---------------------------------------------------

gi|335578432 ------------------------------------------------------------

gi|374383060 ------------------------------------------------------------

gi|358248320 -------IS---------------------------------------------------

gi|300919677 ------------------------------------------------------------

gi|194902914 --------D---------------------------------------------------

gi|226492509 -------IS---------------------------------------------------

gi|391290786 ------------------------------------------------------------

lcl|84191 ----------------------STLDFMLGA-KADG--ETILKGLQS-------------

gi|168177377 ----------------------STLDFMLGA-KADG--ETILKGLQS-------------

gi|21264341 ----------------------STLDFMLGA-KADG--ETILKGLQS-------------

gi|791051 ----------------------STLDFMLGA-KADG--ETILKGLQS-------------

gi|332860397 ----------------------STLDFMLGA-KADG--ETILKGLQS-------------

gi|119619392 ----------------------STLDFMLGA-KADG--ETILKGLQS-------------

gi|402909680 ----------------------STLDFMLGA-KADG--ETILKGLQS-------------

gi|332864823 ----------------------STLDFMLGA-KADG--ETILKGLQS-------------

gi|354474372 ----------------------STLDFTLGA-KADG--EAILKGLQS-------------

gi|291407209 ----------------------SAQLSELGK-TANG--ETILKGLQS-------------

gi|297303473 ---------------------------MLGA-KADG--ETILKGLQS-------------

gi|403263885 ----------------------WPRDLFLFLQAADG--ETILKGLQS-------------

gi|395753759 ----------------------CPRDLFLFLQAADG--ETILKGLQS-------------

gi|355704663 ------------------------LPSSLCS-PADG--ETILKGLQS-------------

gi|4164136 ----------------------------------DG--ETILKGLQS-------------

gi|149030940 ----------------------STLDFTLGA-KADG--EAILKGLQS-------------

gi|76559933 ----------------------STLDFTLGA-KADG--EAILKGLQS-------------

gi|78369282 ----------------------STLDFMLGA-KADG--ETILKGLQS-------------

gi|118600998 ----------------------STLDFKLGA-KADG--EAILKGLQS-------------

gi|345806962 ----------------------STLDFKLGA-RADG--ETILKGLQS-------------

gi|154425706 ----------------------STLDFMLGA-KADG--ETILKGLQS-------------

gi|26340516 ----------------------STLDFKLGA-KADG--EAILKGLQS-------------

gi|384948950 ----------------------STLDFMLGA-KADG--ETILKGLQS-------------

gi|397497639 ------------------------------------------------------------

gi|348561339 ----------------------LAKDLSCPA--LAGFLATVGEAVPPERDEGREK--EAI

gi|354473432 ----------------------GGGSAPLGA-KADG--EAILRGLQP-------------

gi|344288661 ----------------------GRRWISPGGGGADG--ETILKGLQS-------------

gi|338728955 ----------------------AVLKEEIVL-AADG--ETILKGLQS-------------

gi|71895235 ------------------------------------------------MAAARHSTLDFM

gi|297678474 ----------------------STLDFMLGA-KADS--ETILKGLQS-------------

gi|301756254 ----------------------LPGDIKLHL-GADG--ETILKGLQS-------------

gi|296470508 ----------------------STLDFMLGA-KADG--ETILKGLQS-------------

gi|351712477 ---------------------------------ADG--ETILKGLQS-------------

gi|344242683 ----------------------STLDFTLGA-KADADSEAILKGLQS-------------

gi|387018788 ----------------------STLDFMLSA-TADC--NAIVKGLQS-------------

gi|327268286 ----------------------STLDLTLAA-TADC--NAIFKGLQS-------------

gi|334329503 ALLHPCPPLCILPLEAHPVSPAPSLPERFDSSWMGG----LEVSVPSPSRGRGFVSPPAF

gi|350595577 ----------------------SPLCLKGGA---DG--ETILKGLQS-------------

gi|351706018 ----------------------STLNFMLGA-KADG--ETILKGLQS-------------

gi|395518817 -----------------------MHDLIMLL-QADG--DAILKGLQS-------------

gi|149042406 ------------------------------------------------------------

gi|326913546 --------------------------------------------------------MDNF

gi|224042691 ------------------------------------------------MADACQK-----

gi|148708890 ------------------------------------------------------------

gi|345327044 ----------------------RSLPEQIREYVADG--SVIVKGLQS-------------

gi|385862226 ----------------------NTLDFQLSV-TADG--NTIIKDLQP-------------

gi|354482601 ----------------------STLDFTLGA-KADG--ETILKGLQS-------------

gi|281352943 ---------------------------------ADG--ETILKGLQS-------------

gi|18858203 -----------------MAVLHYTLDFKLRA-PADV--SATVRGLQS-------------

gi|395838143 ----------------------STLDFMLGA-KAAAPGREGTLGVPADRAALPGP--DCP

gi|213511272 -----------------MALRHYTLDFNLSA-P-DC--PTTVHGLQS-------------

gi|348518728 -----------------MAVRHYTLDFNVST-AVDP--TSTVSGLLS-------------

gi|386643030 ----------------------STLDFMLGA-KADG--ETILKGLQS-------------

gi|402909682 ----------------------STLDFMLGA-KADG--ETILKGLQS-------------

gi|4007861 -----------------MAVLHHSLDFILSS-PVDS--ASTVPGLLS-------------

gi|351706975 ------------------------------------------------------------

gi|4007046 -----------------MAVLHHSLDFILFS-PVDS--ASTVPSLLS-------------

gi|114687978 ----------------------STLDFMLGA-KADG--ETILKGLQS-------------

gi|2916761 ----------------------STLDFKLGA-KADG--EAILKGLQS-------------

gi|194373711 ------------------------------------------------------------

gi|332860399 ------------------------------------------------------------

gi|125978661 -----------------MAAQTILFDFTLDSAKTAD--EKQRQEVAK-------------

gi|225581217 -----------------MAAQTILFDFTLDSAKTGD--EKQRQEVAK-------------

gi|195376249 -----------------MAAQTILFDFTLDAGKTAT--AIQRQEVAR-------------

gi|195126140 -----------------MAAQTILFDFTLDAGKTAT--SIQRQEVAR-------------

gi|194747133 -----------------MAAQTILFDFTLDKDKTGD--EKQRQDVAK-------------

gi|195012869 -----------------MAAQTILFDFTLDAGKTAT--AMQRQEVAR-------------

gi|24663238 -----------------MAAQTILFDFTLDKDKTAD--EEARLQVAK-------------

gi|194869781 -----------------MAAQTILFDFTLDKDKTAD--EEARLQVAK-------------

gi|195327091 -----------------MAAQTILFDFTLDKDKTAD--EEARLQVAK-------------

gi|390176333 -----------------MAAQTILFDFTLDSAKTAD--EKQRQEVAK-------------

gi|21358269 -----------------MAAQTILFDFTLDKDKTAD--EEARLQVAK-------------

gi|74211624 ----------------------STLDFKLGA-KADG--EAILKGLQS-------------

gi|195589784 -----------------MAAQTILFDFTLDKDKTAD--EEARLQVAK-------------

gi|289742457 -----------------MAAQTILFDFTVDKERTTD--AKERQEIAK-------------

gi|157118336 -----------------MSANSVLLDFSLDPTRIND--EVSRKDIVK-------------

gi|158293995 -----------------MSANSILLDFSLDPARIID--EVSRKDIVR-------------

gi|170032109 -----------------MSANSVLLDFSLDPTRISD--EVSRKDIVK-------------

gi|327290877 ------------------------------------------------------------

gi|4927220 ------------------------------------------------------------

gi|195493801 -----------------MAAQTILFDFTLDKDKTAD--EEARLQVAK-------------

gi|242024750 -----------------MAVNTFLLDFTVNPNACET--VEDRYMIKE-------------

gi|312372613 -----------------MSANTVLLDFSLDPARIND--EVSRKDIVK-------------

gi|119619391 ----------------------STLDFMLGA-KADG--ETILKGLQS-------------

gi|260818384 -----------------------------------------------------MPAFHTL

gi|340709405 -----------------MVAHTVLLDFTVPSNVIVD--VEKRSNLKI-------------

gi|350407163 -----------------MVAHTVLLDFTVPSNVIVD--VEKRSNLKI-------------

gi|383856563 -----------------MVAHTVLLDFTVPPNVIVD--VEKRSSLKL-------------

gi|357613964 -----------------MSVQTILLDFSLDPARLGD--ESGQKTVFS-------------

gi|156542873 -----------------MVAHTVLLDFTVPSGQIMD--VDKRANLKA-------------

gi|332025522 -----------------MVAHTVLLDFTVSSSVIAD--MEKRSGLKS-------------

gi|110750077 -----------------MVAHTVLLDFTVPSNVIVD--VEKRSTLKL-------------

gi|91085293 -----------------MSVNTILMDFSVDPSAVKN--DSQVSVVSS-------------

gi|307188353 -----------------MVAHTVLLDFTVSSSVIAD--MEKRSGLKS-------------

gi|390338494 -------------------------------------------MDVRTVLLDFRVESEIL

gi|195440770 -----------------MAAQTILFDFTLDKDKTAD--AQQRQEVAK-------------

gi|380023489 -----------------MVAHTVLLDFTVPSNVIVD--VEKRSTLKL-------------

gi|156394332 -------------------------------------------M----------------

gi|167538084 ------------------------------------------------------------

gi|326435685 --------------------------------MSEG-SEVAVRGLHASPCLAAHNGSDRV

gi|47227186 -----------------MAVLHHSLDFILSS-PVDS--ASXXXXXXX-------------

gi|321461882 --------------METESHSTFLMEFHTQQEKFSS--PD---ELIS-------------

gi|344241635 ------------------------------------------------------------

gi|389613513 ------------------------------------------------------------

gi|313219992 ------------------------------------------------------------

gi|313225743 ------------------------------------------------------------

gi|198418193 ------------------------------------------------------------

gi|391343942 ---------------------------------------------MSCVSRSILIDVHLP

gi|196014283 ------------------------------------------------------------

gi|307204802 ------------------------------------------------------------

gi|225718096 -------------------------------------------MVKKIYTALFDYQLEFL

gi|324096424 ------------------------------------------------------------

gi|327352193 ------------------------------------------------------------

gi|226293104 ------------------------------------------------------------

gi|154277382 ------------------------------------------------------------

gi|212532035 ------------------------------------------------------------

gi|242117904 ------------------------------------------------------------

gi|134077286 ------------------------------------------------------------

gi|348570938 ------------------------------------------------------------

gi|389628860 ------------------------------------------------------------

gi|119495240 ------------------------------------------------------------

gi|67516811 ------------------------------------------------------------

gi|242774578 ------------------------------------------------------------

gi|225683737 ------------------------------------------------------------

gi|255716758 ------------------------------------------------------------

gi|395218408 ------------------------------------------------------------

gi|348514634 ------------------------------------------------------------

gi|347828643 ------------------------------------------------------------

gi|361131873 ------------------------------------------------------------

gi|392864754 ------------------------------------------------------------

gi|115492019 ------------------------------------------------------------

gi|212532039 ------------------------------------------------------------

gi|340914898 ------------------------------------------------------------

gi|156044358 ------------------------------------------------------------

gi|342887616 ------------------------------------------------------------

gi|302307177 ------------------------------------------------------------

gi|367038823 ------------------------------------------------------------

gi|358371985 ------------------------------------------------------------

gi|317030551 ------------------------------------------------------------

gi|303318439 ------------------------------------------------------------

gi|85100119 ------------------------------------------------------------

gi|380088740 ------------------------------------------------------------

gi|395840954 ------------------------------------------------------------

gi|16758208 ------------------------------------------------------------

gi|327287238 ------------------------------------------------------------

gi|146414243 ------------------------------------------------------------

gi|190348490 ------------------------------------------------------------

gi|367025809 ------------------------------------------------------------

gi|121701283 ------------------------------------------------------------

gi|119592082 ------------------------------------------------------------

gi|70995928 ------------------------------------------------------------

gi|6678131 ------------------------------------------------------------

gi|531202 ------------------------------------------------------------

gi|114553926 ------------------------------------------------------------

gi|63253298 ------------------------------------------------------------

gi|122921244 ------------------------------------------------------------

gi|116197943 ------------------------------------------------------------

gi|378733877 ------------------------------------------------------------

gi|26390475 ------------------------------------------------------------

gi|402852915 ------------------------------------------------------------

gi|347447556 ------------------------------------------------------------

gi|383873318 ------------------------------------------------------------

gi|213409616 ------------------------------------------------------------

gi|32766339 ------------------------------------------------------------

gi|387515006 ------------------------------------------------------------

gi|46137467 ------------------------------------------------------------

gi|320583104 ------------------------------------------------------------

gi|350585594 ------------------------------------------------------------

gi|315053415 ------------------------------------------------------------

gi|322706886 ------------------------------------------------------------

gi|402082530 ------------------------------------------------------------

gi|317137159 ------------------------------------------------------------

gi|147902014 ------------------------------------------------------------

gi|344283521 ------------------------------------------------------------

gi|76637216 ------------------------------------------------------------

gi|255937259 ------------------------------------------------------------

gi|366998257 ------------------------------------------------------------

gi|320588782 ------------------------------------------------------------

gi|254580735 ------------------------------------------------------------

gi|301620173 ------------------------------------------------------------

gi|258572568 ------------------------------------------------------------

gi|225710012 -------------------------------------------MSQKIYTALFDYRLPSL

gi|19112807 ------------------------------------------------------------

gi|398411809 ------------------------------------------------------------

gi|379728455 ------------------------------------------------------------

gi|149239971 ------------------------------------------------------------

gi|119175541 ------------------------------------------------------------

gi|320039089 ------------------------------------------------------------

gi|74138635 ------------------------------------------------------------

gi|238489149 ------------------------------------------------------------

gi|255725052 ------------------------------------------------------------

gi|322701141 ------------------------------------------------------------

gi|359378073 ------------------------------------------------------------

gi|358378881 ------------------------------------------------------------

gi|50286095 ------------------------------------------------------------

gi|358391288 ------------------------------------------------------------

gi|401623242 ------------------------------------------------------------

gi|6325326 ------------------------------------------------------------

gi|151942852 ------------------------------------------------------------

gi|302912624 ------------------------------------------------------------

gi|261190484 ------------------------------------------------------------

gi|336262713 ------------------------------------------------------------

gi|296814308 ------------------------------------------------------------

gi|126138914 ------------------------------------------------------------

gi|50420767 ------------------------------------------------------------

gi|68482416 ------------------------------------------------------------

gi|169615146 ------------------------------------------------------------

gi|396485345 ------------------------------------------------------------

gi|241951762 ------------------------------------------------------------

gi|400597126 ------------------------------------------------------------

gi|345561897 ------------------------------------------------------------

gi|310793583 ------------------------------------------------------------

gi|340520373 ------------------------------------------------------------

gi|47228825 ------------------------------------------------------------

gi|156843587 ------------------------------------------------------------

gi|330907629 ------------------------------------------------------------

gi|189207669 ------------------------------------------------------------

gi|50554613 ------------------------------------------------------------

gi|375086054 ------------------------------------------------------------

gi|366996717 ------------------------------------------------------------

gi|380353232 ------------------------------------------------------------

gi|154304795 ------------------------------------------------------------

gi|171693823 ------------------------------------------------------------

gi|260781328 ------------------------------------------------------------

gi|344234284 ------------------------------------------------------------

gi|260951089 ------------------------------------------------------------

gi|367012209 ------------------------------------------------------------

gi|403214093 ------------------------------------------------------------

gi|354548134 ------------------------------------------------------------

gi|372463636 ------------------------------------------------------------

gi|284929129 ------------------------------------------------------------

gi|291533315 ------------------------------------------------------------

gi|384501417 ------------------------------------------------------------

gi|401838282 ------------------------------------------------------------

gi|344302668 ------------------------------------------------------------

gi|365982711 ------------------------------------------------------------

gi|296421657 ------------------------------------------------------------

gi|149695532 ------------------------------------------------------------

gi|50307673 ------------------------------------------------------------

gi|50303991 ------------------------------------------------------------

gi|380483699 ------------------------------------------------------------

gi|325660851 ------------------------------------------------------------

gi|160932658 ------------------------------------------------------------

gi|363751094 ------------------------------------------------------------

gi|254569166 ------------------------------------------------------------

gi|268612001 ------------------------------------------------------------

gi|225018465 ------------------------------------------------------------

gi|326427880 ------------------------------------------------------------

gi|297617720 ------------------------------------------------------------

gi|295665965 ------------------------------------------------------------

gi|317131721 ------------------------------------------------------------

gi|346467789 ------------------------------------------------------------

gi|328766899 ------------------------------------------------------------

gi|323486581 ------------------------------------------------------------

gi|326476682 ------------------------------------------------------------

gi|164660446 ------------------------------------------------------------

gi|169351543 ------------------------------------------------------------

gi|346326304 ------------------------------------------------------------

gi|167519370 ------------------------------------------------------------

gi|321468222 ------------------------------------------------------------

gi|404329717 ------------------------------------------------------------

gi|283798449 ------------------------------------------------------------

gi|300121366 ------------------------------------------------------------

gi|227112962 ------------------------------------------------------------

gi|388582960 ------------------------------------------------------------

gi|225713606 -------------------------------------------MVQKVYTVLFDYRLDII

gi|346466755 ------------------------------------------------------------

gi|355720872 ------------------------------------------------------------

gi|167755034 ------------------------------------------------------------

gi|193215178 ------------------------------------------------------------

gi|403059603 ------------------------------------------------------------

gi|388854692 ------------------------------------------------------------

gi|227329706 ------------------------------------------------------------

gi|253689498 ------------------------------------------------------------

gi|300715359 ------------------------------------------------------------

gi|343427335 ------------------------------------------------------------

gi|1167999 ------------------------------------------------------------

gi|261369006 ------------------------------------------------------------

gi|327405610 ------------------------------------------------------------

gi|254573952 ------------------------------------------------------------

gi|251788647 ------------------------------------------------------------

gi|327309372 ------------------------------------------------------------

gi|313896579 ------------------------------------------------------------

gi|342871552 ------------------------------------------------------------

gi|295101693 ------------------------------------------------------------

gi|302665792 ------------------------------------------------------------

gi|71023471 ------------------------------------------------------------

gi|50122255 ------------------------------------------------------------

gi|320583760 ------------------------------------------------------------

gi|328351802 ------------------------------------------------------------

gi|331082383 ------------------------------------------------------------

gi|320529049 ------------------------------------------------------------

gi|255658393 ------------------------------------------------------------

gi|302500029 ------------------------------------------------------------

gi|307132173 ------------------------------------------------------------

gi|357057706 ------------------------------------------------------------

gi|271501669 ------------------------------------------------------------

gi|348689589 ------------------------------------------------------------

gi|387510768 ------------------------------------------------------------

gi|260588048 ------------------------------------------------------------

gi|160943874 ------------------------------------------------------------

gi|188532978 ------------------------------------------------------------

gi|145362282 ------------------------------------------------------------

gi|394757460 ------------------------------------------------------------

gi|397614365 ------------------------------------------------------------

gi|170025697 ------------------------------------------------------------

gi|401624651 ------------------------------------------------------------

gi|51595070 ------------------------------------------------------------

gi|257439175 ------------------------------------------------------------

gi|238926770 ------------------------------------------------------------

gi|392423567 ------------------------------------------------------------

gi|151941307 ------------------------------------------------------------

gi|270263031 ------------------------------------------------------------

gi|153813543 ------------------------------------------------------------

gi|21957501 ------------------------------------------------------------

gi|325184913 ------------------------------------------------------------

gi|292670864 ------------------------------------------------------------

gi|108808904 ------------------------------------------------------------

gi|154623414 ------------------------------------------------------------

gi|259148132 ------------------------------------------------------------

gi|402833951 ------------------------------------------------------------

gi|242240254 ------------------------------------------------------------

gi|196007934 ------------------------------------------------------------

gi|391628126 ------------------------------------------------------------

gi|323308143 ------------------------------------------------------------

gi|377578674 ------------------------------------------------------------

gi|402570735 ------------------------------------------------------------

gi|355378207 ------------------------------------------------------------

gi|401565378 ------------------------------------------------------------

gi|6323175 ------------------------------------------------------------

gi|354559941 ------------------------------------------------------------

gi|359463278 ------------------------------------------------------------

gi|295108885 ------------------------------------------------------------

gi|158319435 ------------------------------------------------------------

gi|334128073 ------------------------------------------------------------

gi|312162112 ------------------------------------------------------------

gi|313114511 ------------------------------------------------------------

gi|349579866 ------------------------------------------------------------

gi|116785514 ------------------------------------------------------------

gi|333929035 ------------------------------------------------------------

gi|260887129 ------------------------------------------------------------

gi|386825267 ------------------------------------------------------------

gi|37524842 ------------------------------------------------------------

gi|198427208 ------------------------------------------------------------

gi|291545477 ------------------------------------------------------------

gi|182418090 ------------------------------------------------------------

gi|259907494 ------------------------------------------------------------

gi|291549279 ------------------------------------------------------------

gi|157372235 ------------------------------------------------------------

gi|297845432 ------------------------------------------------------------

gi|12230014 ------------------------------------------------------------

gi|170032648 ------------------------------------------------------------

gi|198453209 ------------------------------------------------------------

gi|284049217 ------------------------------------------------------------

gi|238754470 ------------------------------------------------------------

gi|257126567 ------------------------------------------------------------

gi|385785731 ------------------------------------------------------------

gi|238759889 ------------------------------------------------------------

gi|238750530 ------------------------------------------------------------

gi|6094336 ------------------------------------------------------------

gi|332160447 ------------------------------------------------------------

gi|377851579 ------------------------------------------------------------

gi|388391237 ------------------------------------------------------------

gi|91209189 ------------------------------------------------------------

gi|169837854 ------------------------------------------------------------

gi|317046947 ------------------------------------------------------------

gi|256273016 ------------------------------------------------------------

gi|218547577 ------------------------------------------------------------

gi|374579091 ------------------------------------------------------------

gi|373245798 ------------------------------------------------------------

gi|387605598 ------------------------------------------------------------

gi|187935464 ------------------------------------------------------------

gi|384470785 ------------------------------------------------------------

gi|329298049 ------------------------------------------------------------

gi|110803121 ------------------------------------------------------------

gi|212639268 ------------------------------------------------------------

gi|20807781 ------------------------------------------------------------

gi|253988276 ------------------------------------------------------------

gi|315294649 ------------------------------------------------------------

gi|383191575 ------------------------------------------------------------

gi|386228114 ------------------------------------------------------------

gi|330009512 ------------------------------------------------------------

gi|296086541 ------------------------------------------------------------

gi|392297662 ------------------------------------------------------------

gi|292489273 ------------------------------------------------------------

gi|225424695 ------------------------------------------------------------

gi|24111564 ------------------------------------------------------------

gi|194433428 ------------------------------------------------------------

gi|333978511 ------------------------------------------------------------

gi|378194398 ------------------------------------------------------------

gi|238791204 ------------------------------------------------------------

gi|297841771 ------------------------------------------------------------

gi|15799805 ------------------------------------------------------------

gi|110799842 ------------------------------------------------------------

gi|324112468 ------------------------------------------------------------

gi|260890661 ------------------------------------------------------------

gi|401840422 ------------------------------------------------------------

gi|312970216 ------------------------------------------------------------

gi|16128114 ------------------------------------------------------------

gi|386248282 ------------------------------------------------------------

gi|366159436 ------------------------------------------------------------

gi|323975716 ------------------------------------------------------------

gi|384503184 ------------------------------------------------------------

gi|18309532 ------------------------------------------------------------

gi|372276039 ------------------------------------------------------------

gi|333009123 ------------------------------------------------------------

gi|332097889 ------------------------------------------------------------

gi|381405644 ------------------------------------------------------------

gi|303325156 ------------------------------------------------------------

gi|377919674 ------------------------------------------------------------

gi|300905534 ------------------------------------------------------------

gi|15223115 ------------------------------------------------------------

gi|145336078 ------------------------------------------------------------

gi|206578746 ------------------------------------------------------------

gi|42524959 ------------------------------------------------------------

gi|323157811 ------------------------------------------------------------

gi|391275496 ------------------------------------------------------------

gi|195446427 ------------------------------------------------------------

gi|251778321 ------------------------------------------------------------

gi|152968708 ------------------------------------------------------------

gi|371605278 ------------------------------------------------------------

gi|345395892 ------------------------------------------------------------

gi|385302583 ------------------------------------------------------------

gi|386206487 ------------------------------------------------------------

gi|238761600 ------------------------------------------------------------

gi|194746211 ------------------------------------------------------------

gi|331226164 ------------------------------------------------------------

gi|340742010 ------------------------------------------------------------

gi|50552163 ------------------------------------------------------------

gi|238786761 ------------------------------------------------------------

gi|386033187 ------------------------------------------------------------

gi|6094327 ------------------------------------------------------------

gi|82775410 ------------------------------------------------------------

gi|110762382 ------------------------------------------------------------

gi|333011508 ------------------------------------------------------------

gi|322834377 ------------------------------------------------------------

gi|374992883 ------------------------------------------------------------

gi|308185706 ------------------------------------------------------------

gi|157147448 ------------------------------------------------------------

gi|12229958 ------------------------------------------------------------

gi|33340515 ------------------------------------------------------------

gi|295098625 ------------------------------------------------------------

gi|380021423 ------------------------------------------------------------

gi|238797200 ------------------------------------------------------------

gi|293394516 ------------------------------------------------------------

gi|99083515 ------------------------------------------------------------

gi|388608380 ------------------------------------------------------------

gi|320173653 ------------------------------------------------------------

gi|161612515 ------------------------------------------------------------

gi|378073834 ------------------------------------------------------------

gi|161504706 ------------------------------------------------------------

gi|195499508 ------------------------------------------------------------

gi|2821961 ------------------------------------------------------------

gi|359376594 ------------------------------------------------------------

gi|374297740 ------------------------------------------------------------

gi|239827059 ------------------------------------------------------------

gi|403383804 ------------------------------------------------------------

gi|304396607 ------------------------------------------------------------

gi|397166809 ------------------------------------------------------------

gi|123441066 ------------------------------------------------------------

gi|82542724 ------------------------------------------------------------

gi|12229957 ------------------------------------------------------------

gi|376394468 ------------------------------------------------------------

gi|218687999 ------------------------------------------------------------

gi|170768493 ------------------------------------------------------------

gi|365156921 ------------------------------------------------------------

gi|195330342 ------------------------------------------------------------

gi|340356265 ------------------------------------------------------------

gi|388336724 ------------------------------------------------------------

gi|188589068 ------------------------------------------------------------

gi|376400744 ------------------------------------------------------------

gi|28317069 ------------------------------------------------------------

gi|389842198 ------------------------------------------------------------

gi|260596552 ------------------------------------------------------------

gi|6094335 ------------------------------------------------------------

gi|365988413 ------------------------------------------------------------

gi|194446066 ------------------------------------------------------------

gi|350536257 ------------------------------------------------------------

gi|357485917 ------------------------------------------------------------

gi|296127808 ------------------------------------------------------------

gi|74310739 ------------------------------------------------------------

gi|323166003 ------------------------------------------------------------

gi|126153731 ------------------------------------------------------------

gi|291280945 ------------------------------------------------------------

gi|49425361 ------------------------------------------------------------

gi|334341918 ------------------------------------------------------------

gi|187731639 ------------------------------------------------------------

gi|156935354 ------------------------------------------------------------

gi|376377905 ------------------------------------------------------------

gi|392391837 ------------------------------------------------------------

gi|331645238 ------------------------------------------------------------

gi|378578181 ------------------------------------------------------------

gi|24645443 ------------------------------------------------------------

gi|335578432 ------------------------------------------------------------

gi|374383060 ------------------------------------------------------------

gi|358248320 ------------------------------------------------------------

gi|300919677 ------------------------------------------------------------

gi|194902914 ------------------------------------------------------------

gi|226492509 ------------------------------------------------------------

gi|391290786 ------------------------------------------------------------

lcl|84191 ----------------IFQEQGMAESVHTWQ---------------DHGYLATYTNKNG-

gi|168177377 ----------------IFQEQGMAESVHTWQ---------------DHGYLATYTNKNG-

gi|21264341 ----------------IFQEQGMAESVHTWQ---------------DHGYLATYTNKNG-

gi|791051 ----------------IFQEQGMAESVHTWQ---------------DHGYLATYTNKNG-

gi|332860397 ----------------IFQEQGMAESVHTWQ---------------DHGYLATYTNKNG-

gi|119619392 ----------------IFQEQGMAESVHTWQ---------------DHGYLATYTNKNG-

gi|402909680 ----------------IFQEQGMAESVHTWQ---------------DHGYLATYTNKNG-

gi|332864823 ----------------IFQEQGMAESVHTWQ---------------DHGYLATYTNKNG-

gi|354474372 ----------------IFQEQGMAESVHTWQ---------------DHGYLATYTNKNG-

gi|291407209 ----------------IFQEQGMAESVHTWQ---------------DHGYLATYTNKNG-

gi|297303473 ----------------IFQEQGMAESVHTWQ---------------DHGYLATYTNKNG-

gi|403263885 ----------------IFQEQGMAESVHTWQ---------------DHGYLATYTNKNG-

gi|395753759 ----------------IFQEQGMAESVHTWQ---------------DHGYLATYTNKNG-

gi|355704663 ----------------IFQEQGMAESVHTWQ---------------DHGYLATYTNKNG-

gi|4164136 ----------------IFQEQGMAESVHTWQ---------------DHGYLATYTNKNG-

gi|149030940 ----------------IFQEQGMAESVHTWQ---------------DHGYLATYTNKNG-

gi|76559933 ----------------IFQEQGMAESVHTWQ---------------DHGYLATYTNKNG-

gi|78369282 ----------------IFQEQGMTESVHTWQ---------------DHGYLATYINKNG-

gi|118600998 ----------------IFQEQGMTESVHTWQ---------------DHGYLATYTNKNG-

gi|345806962 ----------------IFQEQGMTESVHTWQ---------------DHGYLATYINKNG-

gi|154425706 ----------------IFQEQGMTESVHTWQ---------------DHGYLATYINKNG-

gi|26340516 ----------------IFQEQGMTESVHTWQ---------------DHGYLATYTNKNG-

gi|384948950 ----------------IFQEQGMAESVHTWQ---------------DHGYLATYTNKNG-

gi|397497639 ----------------------MAESVHTWQ---------------DHGYLATYTNKNG-

gi|348561339 IIIGADGETILKGLQSIFQEQGMAESVHTWQ---------------DHGYLATYTNKNG-

gi|354473432 ----------------IFQEQGMAESVHTWQ---------------DHGYLATYTNKNG-

gi|344288661 ----------------IFQEQGMTESVHTWQ---------------DHGYLATYTNKNG-

gi|338728955 ----------------IFQEQGMTESVHTWQ---------------DHGYLATYINKNG-

gi|71895235 LGATADCNAVLKALQPVFQEQGMTETVHNWE---------------DHGYLATYIKKNG-

gi|297678474 ----------------IFQEQGMAKSVHTWQ---------------DHGYLATYTNKNG-

gi|301756254 ----------------IFQEQGMTESVHNWQ---------------DHGYLATYINKNG-

gi|296470508 ----------------IFQEQGMTESVHTWQ---------------DHGYLATYINKNG-

gi|351712477 ----------------IFQEQGMAESVHTWQ---------------DHGYLATYTNKNG-

gi|344242683 ----------------IFQEQGMAESVHTWQ---------------DHGYLATYTNKNG-

gi|387018788 ----------------IFQKEGMTETIHNWE---------------NHGYLATYVNKNG-

gi|327268286 ----------------IFQKQGMTETIHNWE---------------DHGYLATYVNKNG-

gi|334329503 YWTSPDGDAILKGLQSVFEEQAMTETIHTWE---------------DHGYLATYINKNG-

gi|350595577 ----------------IFQEQGMTESVHTWQ---------------DHGYLATYINKNG-

gi|351706018 ----------------IFQEQGMAESVHTWE---------------DHAYLATYTNKNG-

gi|395518817 ----------------VFEEQAMTETIHSWE---------------EHGYLATYINKNG-

gi|149042406 ----------------------MAESVHTWQ---------------DHGYLATYTNKNG-

gi|326913546 SGR-ADCNAVLKALQPIFQEQGMTETVHNWE---------------DHGYLATYIKKNG-

gi|224042691 LQVQSDCNAVLKALQPIFQEQGMTETVHNWE---------------DHGYLVTYIKKNG-

gi|148708890 ----------------------MTESVHTWQ---------------DHGYLATYTNKNG-

gi|345327044 ----------------VFQKRGMTETVHSWE---------------DHGYLATYVNKNG-

gi|385862226 ----------------IFQEQGMTETVHAWE---------------DHGYLATYTGKKG-

gi|354482601 ----------------IFQEQGMAESVHTWQ---------------DHGYLATYTNKNG-

gi|281352943 ----------------IFQEQGMTESVHNWQ---------------DHGYLATYINKNG-

gi|18858203 ----------------IFQEQEMTENVHDSE---------------GHGYLATFIGKNS-

gi|395838143 PVPHSGSPPPCPCHRVLFRLPGPGGPPRGRR---------------CSGCVERGVTRNG-

gi|213511272 ----------------IFQEQEMTETVHDTE---------------GHGYLATFIGKNG-

gi|348518728 ----------------IFHEQELTETIHDTA---------------GHGYLATFVGKNG-

gi|386643030 ----------------IFQEQGMAESVHTWQ---------------DHGYLATYTNKNG-

gi|402909682 ----------------IFQEQGMAESVHTWQ---------------DHGYLATYTNKNG-

gi|4007861 ----------------IFHEQGMTETVHDTQ---------------GHGYLATLVGKNG-

gi|351706975 ----------------------MAESVHTWQ---------------DHGYLATYTNKIG-

gi|4007046 ----------------IFHEQEMTETVHDTK---------------GHGYLATLVGKHG-

gi|114687978 ----------------IFQEQGMAESVHTWQ---------------DHGYLATYTNKNG-

gi|2916761 ----------------IFQEQGMTESVHTWQ---------------DHGYLATYTNKNG-

gi|194373711 ------------------------------------------------------------

gi|332860399 ------------------------------------------------------------

gi|125978661 --------ILRNELEHVFPQLELAYSMESPE----------------NGYFSVLHENKA-

gi|225581217 --------ILRNELEHVFPQLELAYSMESPE----------------NGYFSVLHENKA-

gi|195376249 --------IVRNELEHVFSQLELAYSMESMD----------------NGYFSVLHENKE-

gi|195126140 --------IVRNELEHVFTQLELAYSMESMD----------------NGYFSVLHENKE-

gi|194747133 --------ILRNELEHIFPQLELAYCMESPE----------------NGYFAVLHENKD-

gi|195012869 --------IARNELEHVFSQLELAYSLESMD----------------NGYFSVLHENKE-

gi|24663238 --------ILRNELEQLFPQLELAYSMESPE----------------NGYFAVLHENKD-

gi|194869781 --------ILRNELEQLFPQLELAYSMESLE----------------NGYFAVLHENKD-

gi|195327091 --------ILRNELEQLFPQLELAYSMESPE----------------NGYFAVLHENKD-

gi|390176333 --------ILRNELEHVFPQLELAYSMESPE----------------NGYFSVLHENKA-

gi|21358269 --------ILRNELEQLFPQLELAYSMESPE----------------NGYFAVLHENKD-

gi|74211624 ----------------IFQEQGMTESVHTWQ---------------DHGYLATYTNKNG-

gi|195589784 --------ILRNELEQLFPQLELAYSMESPE----------------NGYFAVLHENKD-

gi|289742457 --------LLRQELEHIFPQLNLMYQMP-ME----------------HGYYCVLSENQD-

gi|157118336 --------LCKEHLEKYISGLKITYDML-TE----------------DGYLCILNAPGA-

gi|158293995 --------VCKEGLEKYLAGLKISYDML-TT----------------DGYLCILSETGTG

gi|170032109 --------LCKEHLEKYLDTLKITYDML-TE----------------DGYLCVLSS-GP-

gi|327290877 ------------------------------------------------------------

gi|4927220 ------------------------------------------------------------

gi|195493801 --------ILRNELEQLFPQLELAYSMESPE----------------NGYFAVLHENKD-

gi|242024750 --------NVESCLKEYIKDIKCD--SFREL----------------DGGFLSIFFGAKG

gi|312372613 --------LCKESLEKYLPEVKITYDML-TT----------------DGYLCMLSD-AA-

gi|119619391 ----------------IFQEQGMAESVHTWQ---------------DHGYLATYTNKNG-

gi|260818384 LDFQFEPGGPKNPLRDQAVYSDLCALVSGLVQSSGASSLPPVSL-GDSGSLGVFTAENG-

gi|340709405 --------AIANVLQEHFASLKPL--TESSI----------------DGSFLVLYTGPKG

gi|350407163 --------AIANVLQEHFDSLKPL--TESSI----------------DGSFLVLYTGPKG

gi|383856563 --------AIANVLGEHFNGLKPL--TESSI----------------DGSLLILYTGPRG

gi|357613964 --------HLETVLKDYVPNLILAADIK--I----------------DDGSLKLLTGKRG

gi|156542873 --------NITNVLKEHFIGLKHL--TESMV----------------DGSLIILFTGPRG

gi|332025522 --------AIANVLAEHFTGLKPL--TESNI----------------DGSLLILYTGPRG

gi|110750077 --------AITNVLQEYFDSLKPL--TESSI----------------DGSFLVLYTGPRG

gi|91085293 --------NIENVLREYLANLKTLTTIR-LE----------------TDVLRLYSSDGG-

gi|307188353 --------AIGNVLAEHFIGLKPL--TESNI----------------DGSLLVLYTGPRG

gi|390338494 DNLCQTGSKINEQLKGALSSVGLGDNIQ---------------------------VHSS-

gi|195440770 --------ILRNELEHVFPQLELAYSMESEE----------------NGYFSVLHENKD-

gi|380023489 --------AITNVLQEYFDSLKPL--TESSI----------------DGSFLVLYTGPRG

gi|156394332 -------------------FVFVGPN--------------------------------S-

gi|167538084 ---------------MAALTLASRACLNAWLLCLLCTDISFFQAQGPKSALALLTSPNSP

gi|326435685 HHILIDLRA--SKQRMCDLSMTATRMREALQAVEGLALDHVRDCNSDNAAVAFLCS-NAG

gi|47227186 ----------------XXXXXXXXXXXXXXX---------------XXXXXXXXXXXXX-

gi|321461882 --------PIKELLEKYIGEVKEVY-RRCSD----------------QSVSLVLDCAQK-

gi|344241635 ------------------------------------------------------------

gi|389613513 ------------------------------------------------------------

gi|313219992 ---------------------------------------------MIKSVLVDFNCEKPC

gi|313225743 ---------------------------------------------MIKSVLVDFNCEKLC

gi|198418193 -----------------------------------------------------------M

gi|391343942 ASDEGTKHRLLEVLDDQLEQLGLTRTSFTHKLA--------------SSQLYVYVSDNDI

gi|196014283 ------------------------------------------------------------

gi|307204802 ------------------------------------------------------------

gi|225718096 PSAAQIEEHVLGPLCKALKASFQMEERSGCE---------------NTSAENAYDFSGD-

gi|324096424 ------------------------------------------------------------

gi|327352193 ------------------------------------------------------------

gi|226293104 ------------------------------------------------------------

gi|154277382 ------------------------------------------------------------

gi|212532035 ------------------------------------------------------------

gi|242117904 ------------------------------------------------------------

gi|134077286 ------------------------------------------------------------

gi|348570938 ------------------------------------------------------------

gi|389628860 ------------------------------------------------------------

gi|119495240 ------------------------------------------------------------

gi|67516811 ------------------------------------------------------------

gi|242774578 ------------------------------------------------------------

gi|225683737 ------------------------------------------------------------

gi|255716758 ------------------------------------------------------------

gi|395218408 ------------------------------------------------------------

gi|348514634 ------------------------------------------------------------

gi|347828643 ------------------------------------------------------------

gi|361131873 ------------------------------------------------------------

gi|392864754 ------------------------------------------------------------

gi|115492019 ------------------------------------------------------------

gi|212532039 ------------------------------------------------------------

gi|340914898 ------------------------------------------------------------

gi|156044358 ------------------------------------------------------------

gi|342887616 ------------------------------------------------------------

gi|302307177 ------------------------------------------------------------

gi|367038823 ------------------------------------------------------------

gi|358371985 ------------------------------------------------------------

gi|317030551 ------------------------------------------------------------

gi|303318439 ------------------------------------------------------------

gi|85100119 ------------------------------------------------------------

gi|380088740 ------------------------------------------------------------

gi|395840954 ------------------------------------------------------------

gi|16758208 ------------------------------------------------------------

gi|327287238 ------------------------------------------------------------

gi|146414243 ------------------------------------------------------------

gi|190348490 ------------------------------------------------------------

gi|367025809 ------------------------------------------------------------

gi|121701283 ------------------------------------------------------------

gi|119592082 ------------------------------------------------------------

gi|70995928 ------------------------------------------------------------

gi|6678131 ------------------------------------------------------------

gi|531202 ------------------------------------------------------------

gi|114553926 ------------------------------------------------------------

gi|63253298 ------------------------------------------------------------

gi|122921244 ------------------------------------------------------------

gi|116197943 ------------------------------------------------------------

gi|378733877 ------------------------------------------------------------

gi|26390475 ------------------------------------------------------------

gi|402852915 ------------------------------------------------------------

gi|347447556 ------------------------------------------------------------

gi|383873318 ------------------------------------------------------------

gi|213409616 ------------------------------------------------------------

gi|32766339 ------------------------------------------------------------

gi|387515006 ------------------------------------------------------------

gi|46137467 ------------------------------------------------------------

gi|320583104 ------------------------------------------------------------

gi|350585594 ------------------------------------------------------------

gi|315053415 ------------------------------------------------------------

gi|322706886 ------------------------------------------------------------

gi|402082530 ------------------------------------------------------------

gi|317137159 ------------------------------------------------------------

gi|147902014 ------------------------------------------------------------

gi|344283521 ------------------------------------------------------------

gi|76637216 ------------------------------------------------------------

gi|255937259 ------------------------------------------------------------

gi|366998257 ------------------------------------------------------------

gi|320588782 ------------------------------------------------------------

gi|254580735 ------------------------------------------------------------

gi|301620173 ------------------------------------------------------------

gi|258572568 ------------------------------------------------------------

gi|225710012 PSGAEVNEHVCEPLSKALRSSLRCQKRN-VE---------------DDSASEAYELIGG-

gi|19112807 ------------------------------------------------------------

gi|398411809 ------------------------------------------------------------

gi|379728455 ------------------------------------------------------------

gi|149239971 ------------------------------------------------------------

gi|119175541 ------------------------------------------------------------

gi|320039089 ------------------------------------------------------------

gi|74138635 ------------------------------------------------------------

gi|238489149 ------------------------------------------------------------

gi|255725052 ------------------------------------------------------------

gi|322701141 ------------------------------------------------------------

gi|359378073 ------------------------------------------------------------

gi|358378881 ------------------------------------------------------------

gi|50286095 ------------------------------------------------------------

gi|358391288 ------------------------------------------------------------

gi|401623242 ------------------------------------------------------------

gi|6325326 ------------------------------------------------------------

gi|151942852 ------------------------------------------------------------

gi|302912624 ------------------------------------------------------------

gi|261190484 ------------------------------------------------------------

gi|336262713 ------------------------------------------------------------

gi|296814308 ------------------------------------------------------------

gi|126138914 ------------------------------------------------------------

gi|50420767 ------------------------------------------------------------

gi|68482416 ------------------------------------------------------------

gi|169615146 ------------------------------------------------------------

gi|396485345 ------------------------------------------------------------

gi|241951762 ------------------------------------------------------------

gi|400597126 ------------------------------------------------------------

gi|345561897 ------------------------------------------------------------

gi|310793583 ------------------------------------------------------------

gi|340520373 ------------------------------------------------------------

gi|47228825 ------------------------------------------------------------

gi|156843587 ------------------------------------------------------------

gi|330907629 ------------------------------------------------------------

gi|189207669 ------------------------------------------------------------

gi|50554613 ------------------------------------------------------------

gi|375086054 ------------------------------------------------------------

gi|366996717 ------------------------------------------------------------

gi|380353232 ------------------------------------------------------------

gi|154304795 ------------------------------------------------------------

gi|171693823 ------------------------------------------------------------

gi|260781328 ------------------------------------------------------------

gi|344234284 ------------------------------------------------------------

gi|260951089 ------------------------------------------------------------

gi|367012209 ------------------------------------------------------------

gi|403214093 ------------------------------------------------------------

gi|354548134 ------------------------------------------------------------

gi|372463636 ------------------------------------------------------------

gi|284929129 ------------------------------------------------------------

gi|291533315 ------------------------------------------------------------

gi|384501417 ------------------------------------------------------------

gi|401838282 ------------------------------------------------------------

gi|344302668 ------------------------------------------------------------

gi|365982711 ------------------------------------------------------------

gi|296421657 ------------------------------------------------------------

gi|149695532 ------------------------------------------------------------

gi|50307673 ------------------------------------------------------------

gi|50303991 ------------------------------------------------------------

gi|380483699 ------------------------------------------------------------

gi|325660851 ------------------------------------------------------------

gi|160932658 ------------------------------------------------------------

gi|363751094 ------------------------------------------------------------

gi|254569166 ------------------------------------------------------------

gi|268612001 ------------------------------------------------------------

gi|225018465 ------------------------------------------------------------

gi|326427880 ------------------------------------------------------------

gi|297617720 ------------------------------------------------------------

gi|295665965 ------------------------------------------------------------

gi|317131721 ------------------------------------------------------------

gi|346467789 ------------------------------------------------------------

gi|328766899 ------------------------------------------------------------

gi|323486581 ------------------------------------------------------------

gi|326476682 ------------------------------------------------------------

gi|164660446 ------------------------------------------------------------

gi|169351543 ------------------------------------------------------------

gi|346326304 ------------------------------------------------------------

gi|167519370 ------------------------------------------------------------

gi|321468222 ------------------------------------------------------------

gi|404329717 ------------------------------------------------------------

gi|283798449 ------------------------------------------------------------

gi|300121366 ------------------------------------------------------------

gi|227112962 ------------------------------------------------------------

gi|388582960 ------------------------------------------------------------

gi|225713606 PTDKDIQEHVVSPLQKVLNVVLTTEKKV--E---------------DPAATVAYQFIGG-

gi|346466755 ------------------------------------------------------------

gi|355720872 ------------------------------------------------------------

gi|167755034 ------------------------------------------------------------

gi|193215178 ------------------------------------------------------------

gi|403059603 ------------------------------------------------------------

gi|388854692 ------------------------------------------------------------

gi|227329706 ------------------------------------------------------------

gi|253689498 ------------------------------------------------------------

gi|300715359 ------------------------------------------------------------

gi|343427335 ------------------------------------------------------------

gi|1167999 ------------------------------------------------------------

gi|261369006 ------------------------------------------------------------

gi|327405610 ------------------------------------------------------------

gi|254573952 ------------------------------------------------------------

gi|251788647 ------------------------------------------------------------

gi|327309372 ------------------------------------------------------------

gi|313896579 ------------------------------------------------------------

gi|342871552 ------------------------------------------------------------

gi|295101693 ------------------------------------------------------------

gi|302665792 ------------------------------------------------------------

gi|71023471 ------------------------------------------------------------

gi|50122255 ------------------------------------------------------------

gi|320583760 ------------------------------------------------------------

gi|328351802 ------------------------------------------------------------

gi|331082383 ------------------------------------------------------------

gi|320529049 ------------------------------------------------------------

gi|255658393 ------------------------------------------------------------

gi|302500029 ------------------------------------------------------------

gi|307132173 ------------------------------------------------------------

gi|357057706 ------------------------------------------------------------

gi|271501669 ------------------------------------------------------------

gi|348689589 ------------------------------------------------------------

gi|387510768 ------------------------------------------------------------

gi|260588048 ------------------------------------------------------------

gi|160943874 ------------------------------------------------------------

gi|188532978 ------------------------------------------------------------

gi|145362282 ------------------------------------------------------------

gi|394757460 ------------------------------------------------------------

gi|397614365 ------------------------------------------------------------

gi|170025697 ------------------------------------------------------------

gi|401624651 ------------------------------------------------------------

gi|51595070 ------------------------------------------------------------

gi|257439175 ------------------------------------------------------------

gi|238926770 ------------------------------------------------------------

gi|392423567 ------------------------------------------------------------

gi|151941307 ------------------------------------------------------------

gi|270263031 ------------------------------------------------------------

gi|153813543 ------------------------------------------------------------

gi|21957501 ------------------------------------------------------------

gi|325184913 ------------------------------------------------------------

gi|292670864 ------------------------------------------------------------

gi|108808904 ------------------------------------------------------------

gi|154623414 ------------------------------------------------------------

gi|259148132 ------------------------------------------------------------

gi|402833951 ------------------------------------------------------------

gi|242240254 ------------------------------------------------------------

gi|196007934 ------------------------------------------------------------

gi|391628126 ------------------------------------------------------------

gi|323308143 ------------------------------------------------------------

gi|377578674 ------------------------------------------------------------

gi|402570735 ------------------------------------------------------------

gi|355378207 ------------------------------------------------------------

gi|401565378 ------------------------------------------------------------

gi|6323175 ------------------------------------------------------------

gi|354559941 ------------------------------------------------------------

gi|359463278 ------------------------------------------------------------

gi|295108885 ------------------------------------------------------------

gi|158319435 ------------------------------------------------------------

gi|334128073 ------------------------------------------------------------

gi|312162112 ------------------------------------------------------------

gi|313114511 ------------------------------------------------------------

gi|349579866 ------------------------------------------------------------

gi|116785514 ------------------------------------------------------------

gi|333929035 ------------------------------------------------------------

gi|260887129 ------------------------------------------------------------

gi|386825267 ------------------------------------------------------------

gi|37524842 ------------------------------------------------------------

gi|198427208 ------------------------------------------------------------

gi|291545477 ------------------------------------------------------------

gi|182418090 ------------------------------------------------------------

gi|259907494 ------------------------------------------------------------

gi|291549279 ------------------------------------------------------------

gi|157372235 ------------------------------------------------------------

gi|297845432 ------------------------------------------------------------

gi|12230014 ------------------------------------------------------------

gi|170032648 ------------------------------------------------------------

gi|198453209 ------------------------------------------------------------

gi|284049217 ------------------------------------------------------------

gi|238754470 ------------------------------------------------------------

gi|257126567 ------------------------------------------------------------

gi|385785731 ------------------------------------------------------------

gi|238759889 ------------------------------------------------------------

gi|238750530 ------------------------------------------------------------

gi|6094336 ------------------------------------------------------------

gi|332160447 ------------------------------------------------------------

gi|377851579 ------------------------------------------------------------

gi|388391237 ------------------------------------------------------------

gi|91209189 ------------------------------------------------------------

gi|169837854 ------------------------------------------------------------

gi|317046947 ------------------------------------------------------------

gi|256273016 ------------------------------------------------------------

gi|218547577 ------------------------------------------------------------

gi|374579091 ------------------------------------------------------------

gi|373245798 ------------------------------------------------------------

gi|387605598 ------------------------------------------------------------

gi|187935464 ------------------------------------------------------------

gi|384470785 ------------------------------------------------------------

gi|329298049 ------------------------------------------------------------

gi|110803121 ------------------------------------------------------------

gi|212639268 ------------------------------------------------------------

gi|20807781 ------------------------------------------------------------

gi|253988276 ------------------------------------------------------------

gi|315294649 ------------------------------------------------------------

gi|383191575 ------------------------------------------------------------

gi|386228114 ------------------------------------------------------------

gi|330009512 ------------------------------------------------------------

gi|296086541 ------------------------------------------------------------

gi|392297662 ------------------------------------------------------------

gi|292489273 ------------------------------------------------------------

gi|225424695 ------------------------------------------------------------

gi|24111564 ------------------------------------------------------------

gi|194433428 ------------------------------------------------------------

gi|333978511 ------------------------------------------------------------

gi|378194398 ------------------------------------------------------------

gi|238791204 ------------------------------------------------------------

gi|297841771 ------------------------------------------------------------

gi|15799805 ------------------------------------------------------------

gi|110799842 ------------------------------------------------------------

gi|324112468 ------------------------------------------------------------

gi|260890661 ------------------------------------------------------------

gi|401840422 ------------------------------------------------------------

gi|312970216 ------------------------------------------------------------

gi|16128114 ------------------------------------------------------------

gi|386248282 ------------------------------------------------------------

gi|366159436 ------------------------------------------------------------

gi|323975716 ------------------------------------------------------------

gi|384503184 ------------------------------------------------------------

gi|18309532 ------------------------------------------------------------

gi|372276039 ------------------------------------------------------------

gi|333009123 ------------------------------------------------------------

gi|332097889 ------------------------------------------------------------

gi|381405644 ------------------------------------------------------------

gi|303325156 ------------------------------------------------------------

gi|377919674 ------------------------------------------------------------

gi|300905534 ------------------------------------------------------------

gi|15223115 ------------------------------------------------------------

gi|145336078 ------------------------------------------------------------

gi|206578746 ------------------------------------------------------------

gi|42524959 ------------------------------------------------------------

gi|323157811 ------------------------------------------------------------

gi|391275496 ------------------------------------------------------------

gi|195446427 ------------------------------------------------------------

gi|251778321 ------------------------------------------------------------

gi|152968708 ------------------------------------------------------------

gi|371605278 ------------------------------------------------------------

gi|345395892 ------------------------------------------------------------

gi|385302583 ------------------------------------------------------------

gi|386206487 ------------------------------------------------------------

gi|238761600 ------------------------------------------------------------

gi|194746211 ------------------------------------------------------------

gi|331226164 ------------------------------------------------------------

gi|340742010 ------------------------------------------------------------

gi|50552163 ------------------------------------------------------------

gi|238786761 ------------------------------------------------------------

gi|386033187 ------------------------------------------------------------

gi|6094327 ------------------------------------------------------------

gi|82775410 ------------------------------------------------------------

gi|110762382 ------------------------------------------------------------

gi|333011508 ------------------------------------------------------------

gi|322834377 ------------------------------------------------------------

gi|374992883 ------------------------------------------------------------

gi|308185706 ------------------------------------------------------------

gi|157147448 ------------------------------------------------------------

gi|12229958 ------------------------------------------------------------

gi|33340515 ------------------------------------------------------------

gi|295098625 ------------------------------------------------------------

gi|380021423 ------------------------------------------------------------

gi|238797200 ------------------------------------------------------------

gi|293394516 ------------------------------------------------------------

gi|99083515 ------------------------------------------------------------

gi|388608380 ------------------------------------------------------------

gi|320173653 ------------------------------------------------------------

gi|161612515 ------------------------------------------------------------

gi|378073834 ------------------------------------------------------------

gi|161504706 ------------------------------------------------------------

gi|195499508 ------------------------------------------------------------

gi|2821961 ------------------------------------------------------------

gi|359376594 ------------------------------------------------------------

gi|374297740 ------------------------------------------------------------

gi|239827059 ------------------------------------------------------------

gi|403383804 ------------------------------------------------------------

gi|304396607 ------------------------------------------------------------

gi|397166809 ------------------------------------------------------------

gi|123441066 ------------------------------------------------------------

gi|82542724 ------------------------------------------------------------

gi|12229957 ------------------------------------------------------------

gi|376394468 ------------------------------------------------------------

gi|218687999 ------------------------------------------------------------

gi|170768493 ------------------------------------------------------------

gi|365156921 ------------------------------------------------------------

gi|195330342 ------------------------------------------------------------

gi|340356265 ------------------------------------------------------------

gi|388336724 ------------------------------------------------------------

gi|188589068 ------------------------------------------------------------

gi|376400744 ------------------------------------------------------------

gi|28317069 ------------------------------------------------------------

gi|389842198 ------------------------------------------------------------

gi|260596552 ------------------------------------------------------------

gi|6094335 ------------------------------------------------------------

gi|365988413 ------------------------------------------------------------

gi|194446066 ------------------------------------------------------------

gi|350536257 ------------------------------------------------------------

gi|357485917 ------------------------------------------------------------

gi|296127808 ------------------------------------------------------------

gi|74310739 ------------------------------------------------------------

gi|323166003 ------------------------------------------------------------

gi|126153731 ------------------------------------------------------------

gi|291280945 ------------------------------------------------------------

gi|49425361 ------------------------------------------------------------

gi|334341918 ------------------------------------------------------------

gi|187731639 ------------------------------------------------------------

gi|156935354 ------------------------------------------------------------

gi|376377905 ------------------------------------------------------------

gi|392391837 ------------------------------------------------------------

gi|331645238 ------------------------------------------------------------

gi|378578181 ------------------------------------------------------------

gi|24645443 ------------------------------------------------------------

gi|335578432 ------------------------------------------------------------

gi|374383060 ------------------------------------------------------------

gi|358248320 ------------------------------------------------------------

gi|300919677 ------------------------------------------------------------

gi|194902914 ------------------------------------------------------------

gi|226492509 ------------------------------------------------------------

gi|391290786 ------------------------------------------------------------

lcl|84191 S--FANLRIYPHGLVLL------D-----LQSYDG--------------DAQGKEEI---

gi|168177377 S--FANLRIYPHGLVLL------D-----LQSYDG--------------DAQGKEEI---

gi|21264341 S--FANLRIYPHGLVLL------D-----LQSYDG--------------DAQGKEEI---

gi|791051 S--FANLRIYPHGLVLL------D-----LQSYDG--------------DAQGKEEI---

gi|332860397 S--FANLRIYPHGLVLL------D-----LQSYDG--------------DAQGKEEI---

gi|119619392 S--FANLRIYPHGLVLL------D-----LQSYDG--------------DAQGKEEI---

gi|402909680 S--FANLRIYPHGLVLL------D-----LQSYDG--------------DAQGKEEI---

gi|332864823 N--FANLRIYPHGLVLL------D-----LQSYDG--------------DAQGKEEI---

gi|354474372 S--FANLRIYPHGLVLL------D-----LQSYDS--------------DAQGKQET---

gi|291407209 S--FANLRIYPHGLVLL------D-----LQSYDS--------------DAQGKEEI---

gi|297303473 S--FANLRIYPHGLVLL------D-----LQSYDG--------------DAQGKEEI---

gi|403263885 S--FANLRIYPHGLVLL------D-----LQSYDG--------------DAQGKEEI---

gi|395753759 S--FANLRIYPHGLVLL------D-----LQSYDG--------------DAQGKEEI---

gi|355704663 S--FANLRIYPHGLVLL------D-----LQSYDG--------------DAQGKEEI---

gi|4164136 S--FANLRIYPHGLVLL------D-----LQSYDG--------------DAQGKEEI---

gi|149030940 S--FANLRIYPHGLVLL------D-----LQSYDS--------------DVQGKQET---

gi|76559933 S--FANLRIYPHGLVLL------D-----LQSYDS--------------DVQGKQET---

gi|78369282 S--FANLRIYPHGLVLL------D-----LQSYDG--------------DAQGKE-V---

gi|118600998 S--FANLRIYPHGLVLL------D-----LQSYDS--------------DVQGKQET---

gi|345806962 S--FANLRIYPHGLVLL------D-----LQSYDG--------------DSQGQE-V---

gi|154425706 S--FANLRIYPHGLVLL------D-----LQSYDG--------------DAQGKE-V---

gi|26340516 S--FANLRIYPHGLVLL------D-----LQSYDS--------------DVQGKQET---

gi|384948950 S--FANLRIYPHGLVLL------D-----LQSYDG--------------DAQGKEEI---

gi|397497639 S--FANLRIYPHGLVLL------D-----LQSYDG--------------DAQGKEEI---

gi|348561339 S--FANLRIYPHGLVLF------D-----LQSYDS--------------DSQGKEEI---

gi|354473432 S--FANLRIYPHGLVLL------D-----LQSYDS--------------DAQGKQET---

gi|344288661 S--FANLRIYPHGLVLL------D-----LQSYDN--------------DVQGNKEI---

gi|338728955 S--FANLRIYPHGLVLL------D-----LQSYDS--------------DAQG-KEV---

gi|71895235 S--FANLRIHPHGLVLV------D-----LQSYND--------------HTKGREET---

gi|297678474 S--FANLRIYPHGLVLL------D-----LQSYDG--------------DAQGKEEI---

gi|301756254 S--FANLRIYPHGLVLF------D-----LQSYDG--------------DSQG-QEV---

gi|296470508 S--FANLRIYPHGLVLL------D-----LQSYDG--------------DAQGKE-V---

gi|351712477 S--FANLRIYPHGLVLL------D-----LQSYDS--------------DIQGKEEI---

gi|344242683 S--FANLRIYPHGLVLL------D-----LQSYDS--------------DAQGKQET---

gi|387018788 C--FANLRIYPHGLVLV------D-----VQSCN---------------DLNEREEI---

gi|327268286 S--FANLRIYPHGLVLV------D-----IQTYN---------------DLNGGEDA---

gi|334329503 S--FASLRIYPHGLVLM------D-----VQSYNN--------------DLKGKEEI---

gi|350595577 S--FANLRIYPHGLVLL------D-----LQSYDG--------------DAQG-KEV---

gi|351706018 S--FTNLRICPHGLVLL------D-----LQSYNS--------------DIQGKEEI---

gi|395518817 S--FASLRIYPHGLVLM------D-----VQSYNN--------------DLKGREEI---

gi|149042406 S--FANLRIYPHGLVLL------D-----LQSYDS--------------DVQGKQET---

gi|326913546 S--FANLRIHPHGLVLV------D-----LQSYND--------------HTKGREET---

gi|224042691 S--FANLRIHPHGLVLV------D-----LQSYSD--------------DMKGREEA---

gi|148708890 S--FANLRIYPHGLVLL------D-----LQSYDS--------------DVQGKQET---

gi|345327044 S--FANLRIYPHGLVLM------D-----LQSFGN--------------VAAGREEI---

gi|385862226 S--FATVRIYPHGLVLI------D-----VQCRD---------------EPEEKASV---

gi|354482601 S--FANLRIYPHGLVLL------D-----LQSYDS--------------DVQGKQET---

gi|281352943 S--FANLRIYPHGLVLF------D-----LQSYDG--------------DSQG-QEV---

gi|18858203 R--FAILRMHSHGLVTF------D-----LQCLEG--------------DDAA--QV---

gi|395838143 GPGFANLRIYPHGLVLL------D-----LQSYDS--------------DAQSKEEI---

gi|213511272 R--LVILRVTALGLLTI------D-----LQCCEG--------------DNIV--QV---

gi|348518728 R--FVVLRVHSHGLVTV------D-----LQCYEE--------------DDIA--QL---

gi|386643030 ------------------------------------------------------------

gi|402909682 ------------------------------------------------------------

gi|4007861 R--FAFLRVHSHGTVTI------D-----LQCYQE--------------DSIA--EV---

gi|351706975 S--FTNLRIYPHGLVLL------D-----LQSYDS--------------DIQGKEEI---

gi|4007046 R--LALLRVHSHGMVTI------D-----LQCYQE--------------DSVA--EV---

gi|114687978 S--FANLRIYPHGLVLL------D-----LQSYDG--------------DAQGKEEI---

gi|2916761 S--FANLRIYPHGLVLL------D-----LQSYDS--------------DVQGKQET---

gi|194373711 ------------------------------------------------------------

gi|332860399 ------------------------------------------------------------

gi|125978661 TI--ITCRIFQHGLLTVNVEYFLPAGKEPIMSFD----------------------TM--

gi|225581217 TI--ITCRIFQHGLLTVNVEYFLPAGKEPIMSFD----------------------TM--

gi|195376249 TI--ITCRIFQQGLLTINVEYYLADGKEPIMSFD--------------------------

gi|195126140 TI--ITCRIFQQGLLTINVEYYLAAGKEPVLSLD--------------------------

gi|194747133 TV--ITVRIFQHGLLTLNVEYFLPEGKEPVLSFE--------------------------

gi|195012869 TI--ITCRIFPQGLLTINVEYYLAAGKEPIMSFD--------------------------

gi|24663238 TV--ITCRIFQHGLLTLNVEYFLPDGKEPSISFD--------------------------

gi|194869781 TV--ITCRIFQHGLLTLNVEYFLPDGKEPIISFD--------------------------

gi|195327091 TV--ITCRIFQHGLLTLNVEYFLPDGKEPSISFD--------------------------

gi|390176333 TI--ITCRIFQHGLLTVNVEYFLPAGKEPIMSFD----------------------GLV-

gi|21358269 TV--ITCRIFQHGLLTLNVEYFLPDGKEPSISFD--------------------------

gi|74211624 S--FANLRIYPHGLVLL------D-----LQSYDS--------------DVQGKQET---

gi|195589784 TV--ITCRIFQHGLLTLNVEYFLPDGKEPSISFD--------------------------

gi|289742457 TI--ITMRIFQQGLVTINVEYYLENGKEPLMSFD--------------------------

gi|157118336 VI--TTIRFFNQGLITVNIEYFRAESEAPKITFE--------------------------

gi|158293995 TI--VTIRFFEQGLITINVEYYRKDGDEAKISFE--------------------------

gi|170032109 II--TTIRFFNQGLITINIEYYRAECEAPKISFE--------------------------

gi|327290877 ------------------------------------------------------------

gi|4927220 ---FANLRIYPHGLVLL------D-----LQSYDS--------------DVQGKQET---

gi|195493801 TV--ITCRIFQHGLLTLNVEYFLPDGKEPIISFDVXXXXMESPENGYFAVLHENKDTVIT

gi|242024750 TF--ITVRGLKEGIVTINIEYYKSETEDDMLTFE--------------------------

gi|312372613 TI--VTIRFFLQGLITINIEYYRQDGEEPKISFE--------------------------

gi|119619391 S--FANLRIYPHGLVLL------D-----LQSYDG--------------DAQGKEEI---

gi|260818384 SH--ATLRIYPQGLLTVDAEMWAAEGEQPSITAEH---------------------VA--

gi|340709405 SL--ITVRGYTEGLITVNIEYYKRDEEEALLDFE--------------------------

gi|350407163 SL--ITVRGYTEGLITVNIEYYKRDEEEALLDFE--------------------------

gi|383856563 SL--ITIRGYTEGLITLNIEYYKRDDEEALLDFE--------------------------

gi|357613964 TT--VSVRLFDRGLVTVNIEYYKEDSEEPLINFK--------------------------

gi|156542873 SI--ITVRGYAEGLVTINIEYYKQDDEEALLSFE--------------------------

gi|332025522 SL--ITVRGYTEGLITLNIEYYKQDDQEALLTFE--------------------------

gi|110750077 SL--ITVRGYTEGLITINIEYYKRDEEEALLDFE--------------------------

gi|91085293 LS--ATLRIFNTGLITLNIEYLKGESQEALLSFE--------------------------

gi|307188353 SL--ITVRGYTEGLITLNIEYYKQDDQEALLTFE--------------------------

gi|390338494 GE-QLFICSFGHARSAMFSGHRPD-----LVTLTF-----QYDRKAKP--SPTNENL---

gi|195440770 TI--ITCRIFQHGLLTLNVEYYLPDGKEPIISFD--------------------------

gi|380023489 SL--ITVRGYTEGLITINIEYYKRDEEEALLDFE--------------------------

gi|156394332 GH-WT-LRSFKNGHACL------D-----IVTVGA-----E---------SNNNDWY---

gi|167538084 H--SVQIKAFEETV-TADISL--S-----LAAGEA-----------APDLTDALERV---

gi|326435685 S--FGTVRALATGLCTVDISL--S-----LTAGEE-----PITEEQVAELKSTLQQVVDE

gi|47227186 X--FALLRVHSHGVVTI------D-----LQCYQE--------------DSIA--EV---

gi|321461882 SI--ATVQLKSSGLVLMNIDI----GGEDSSAFNQ-------------------------

gi|344241635 ------------------------------------------------------------

gi|389613513 ------------------------------------------------------------

gi|313219992 GE---TIELFSNSAAFVDLGL--T-----LQTWNV-----KEIAGTVL--YTGSDGVSLL

gi|313225743 GE---TIELFSNSAAFVDLGL--T-----LQTWNV-----KEIAGTVL--YTGSDGVSLL

gi|198418193 DSWLLEFRFKKEILKDSKLSETFIDGLVEAVHFMIDGGKVVFKNNHDCVNQGFTVFIQEP

gi|391343942 S---GSLRVYPSNKLATLTLE---------ESSDE--------------QAMSNEDI---

gi|196014283 ------------------------------------------------------------

gi|307204802 -------------------------------MFE--------------------------

gi|225718096 GY-FAHLRLYRRDSPQE-----CD-----LMTLLI-----ESKKPIDL--DDDGLEL---

gi|324096424 ------------------------------------------------------------

gi|327352193 TI-------------------------------------K--------------------

gi|226293104 TI-------------------------------------K--------------------

gi|154277382 TI-------------------------------------K--------------------

gi|212532035 TI-------------------------------------K--------------------

gi|242117904 NI-------------------------------------K--------------------

gi|134077286 TI-------------------------------------K--------------------

gi|348570938 AI-------------------------------------R--------------------

gi|389628860 TI-------------------------------------K--------------------

gi|119495240 TI-------------------------------------K--------------------

gi|67516811 TI-------------------------------------K--------------------

gi|242774578 TI-------------------------------------K--------------------

gi|225683737 TI-------------------------------------K--------------------

gi|255716758 TI-------------------------------------K--------------------

gi|395218408 SS-------------------------------------MELRRGQLS---------LLK

gi|348514634 HI-------------------------------------K--------------------

gi|347828643 TI-------------------------------------K--------------------

gi|361131873 TI-------------------------------------K--------------------

gi|392864754 TI-------------------------------------K--------------------

gi|115492019 TI-------------------------------------K--------------------

gi|212532039 TI-------------------------------------K--------------------

gi|340914898 TI-------------------------------------K--------------------

gi|156044358 TI-------------------------------------K--------------------

gi|342887616 TI-------------------------------------K--------------------

gi|302307177 TI-------------------------------------K--------------------

gi|367038823 TI-------------------------------------K--------------------

gi|358371985 TI-------------------------------------K--------------------

gi|317030551 TI-------------------------------------K--------------------

gi|303318439 TI-------------------------------------K--------------------

gi|85100119 TI-------------------------------------Q--------------------

gi|380088740 TI-------------------------------------Q--------------------

gi|395840954 TI-------------------------------------R--------------------

gi|16758208 AI-------------------------------------R--------------------

gi|327287238 AV-------------------------------------K--------------------

gi|146414243 SI-------------------------------------K--------------------

gi|190348490 SI-------------------------------------K--------------------

gi|367025809 TI-------------------------------------K--------------------

gi|121701283 TI-------------------------------------K--------------------

gi|119592082 AI-------------------------------------R--------------------

gi|70995928 TI-------------------------------------K--------------------

gi|6678131 AI-------------------------------------R--------------------

gi|531202 AI-------------------------------------R--------------------

gi|114553926 AI-------------------------------------R--------------------

gi|63253298 AI-------------------------------------R--------------------

gi|122921244 AI-------------------------------------R--------------------

gi|116197943 ------------------------------------------------------------

gi|378733877 TI-------------------------------------K--------------------

gi|26390475 AI-------------------------------------R--------------------

gi|402852915 AI-------------------------------------R--------------------

gi|347447556 AI-------------------------------------R--------------------

gi|383873318 AI-------------------------------------R--------------------

gi|213409616 SI-------------------------------------K--------------------

gi|32766339 NI-------------------------------------K--------------------

gi|387515006 TI-------------------------------------V--------------------

gi|46137467 TI-------------------------------------V--------------------

gi|320583104 MI-------------------------------------K--------------------

gi|350585594 AI-------------------------------------R--------------------

gi|315053415 TI-------------------------------------K--------------------

gi|322706886 TI-------------------------------------K--------------------

gi|402082530 TI-------------------------------------K--------------------

gi|317137159 TI-------------------------------------Q--------------------

gi|147902014 -V-------------------------------------Q--------------------

gi|344283521 AI-------------------------------------R--------------------

gi|76637216 AI-------------------------------------R--------------------

gi|255937259 TI-------------------------------------Q--------------------

gi|366998257 TI-------------------------------------V--------------------

gi|320588782 TI-------------------------------------Q--------------------

gi|254580735 TI-------------------------------------V--------------------

gi|301620173 -V-------------------------------------Q--------------------

gi|258572568 TI-------------------------------------K--------------------

gi|225710012 GF-FALLRLYKRETPKD-----CD-----LMTLMI-----ESRKPIDL--GEDGLEL---

gi|19112807 LI-------------------------------------K--------------------

gi|398411809 TI-------------------------------------K--------------------

gi|379728455 SA-------------------------------------MEINRGQAD---------LLK

gi|149239971 SI-------------------------------------Q--------------------

gi|119175541 TI-------------------------------------K--------------------

gi|320039089 TI-------------------------------------K--------------------

gi|74138635 AI-------------------------------------R--------------------

gi|238489149 TI-------------------------------------Q--------------------

gi|255725052 AI-------------------------------------K--------------------

gi|322701141 TI-------------------------------------K--------------------

gi|359378073 TI-------------------------------------K--------------------

gi|358378881 TI-------------------------------------Q--------------------

gi|50286095 TI-------------------------------------V--------------------

gi|358391288 TI-------------------------------------Q--------------------

gi|401623242 TI-------------------------------------V--------------------

gi|6325326 TI-------------------------------------V--------------------

gi|151942852 TI-------------------------------------V--------------------

gi|302912624 TI-------------------------------------K--------------------

gi|261190484 ------------------------------------------------------------

gi|336262713 TI-------------------------------------Q--------------------

gi|296814308 TI-------------------------------------K--------------------

gi|126138914 SI-------------------------------------K--------------------

gi|50420767 SI-------------------------------------K--------------------

gi|68482416 AI-------------------------------------K--------------------

gi|169615146 TI-------------------------------------K--------------------

gi|396485345 TI-------------------------------------K--------------------

gi|241951762 AI-------------------------------------K--------------------

gi|400597126 TI-------------------------------------K--------------------

gi|345561897 TI-------------------------------------V--------------------

gi|310793583 TI-------------------------------------Q--------------------

gi|340520373 TI-------------------------------------Q--------------------

gi|47228825 HI-------------------------------------K--------------------

gi|156843587 TI-------------------------------------V--------------------

gi|330907629 TI-------------------------------------K--------------------

gi|189207669 TI-------------------------------------K--------------------

gi|50554613 SI-------------------------------------V--------------------

gi|375086054 ------------------------------------------------------------

gi|366996717 TI-------------------------------------V--------------------

gi|380353232 SI-------------------------------------K--------------------

gi|154304795 AI-------------------------------------S--------------------

gi|171693823 TI-------------------------------------K--------------------

gi|260781328 ------------------------------------------------------------

gi|344234284 SI-------------------------------------K--------------------

gi|260951089 SI-------------------------------------K--------------------

gi|367012209 TI-------------------------------------V--------------------

gi|403214093 TI-------------------------------------V--------------------

gi|354548134 SI-------------------------------------K--------------------

gi|372463636 TI-------------------------------------V--------------------

gi|284929129 SA-------------------------------------LELNRGQLE---------LLE

gi|291533315 ------------------------------------------------------------

gi|384501417 CI-------------------------------------V--------------------

gi|401838282 TI-------------------------------------V--------------------

gi|344302668 SI-------------------------------------K--------------------

gi|365982711 TI-------------------------------------V--------------------

gi|296421657 TI-------------------------------------Q--------------------

gi|149695532 ------------------------------------------------------------

gi|50307673 CI-------------------------------------K--------------------

gi|50303991 TI-------------------------------------V--------------------

gi|380483699 ------------------------------------------------------------

gi|325660851 ------------------------------------------------------------

gi|160932658 ------------------------------------------------------------

gi|363751094 SI-------------------------------------K--------------------

gi|254569166 LI-------------------------------------K--------------------

gi|268612001 ------------------------------------------------------------

gi|225018465 ------------------------------------------------------------

gi|326427880 AM-------------------------------------K--------------------

gi|297617720 II-------------------------------------RE-------------------

gi|295665965 TI-------------------------------------K--------------------

gi|317131721 ------------------------------------------------------------

gi|346467789 RL-------------------------------------CIGSCPASH---------LSP

gi|328766899 NI-------------------------------------E--------------------

gi|323486581 ------------------------------------------------------------

gi|326476682 TI-------------------------------------K--------------------

gi|164660446 ------------------------------------------------------------

gi|169351543 ------------------------------------------------------------

gi|346326304 TI-------------------------------------KGTFCWLCL---------EAA

gi|167519370 RL-------------------------------------A--------------------

gi|321468222 SL-------------------------------------K--------------------

gi|404329717 ------------------------------------------------------------

gi|283798449 ------------------------------------------------------------

gi|300121366 S-----------------------------------------------------------

gi|227112962 ------------------------------------------------------------

gi|388582960 ------------------------------------------------------------

gi|225713606 GF-FVLLRIYDKDSSDE-----QV-----LMTLMI-----ESKKPIDL--NENGLEL---

gi|346466755 RF-------------------------------------AFGSCPASH---------LSP

gi|355720872 ------------------------------------------------------------

gi|167755034 ------------------------------------------------------------

gi|193215178 ST-------------------------------------MELRRGQEK---------LLN

gi|403059603 ------------------------------------------------------------

gi|388854692 ------------------------------------------------------------

gi|227329706 ------------------------------------------------------------

gi|253689498 ------------------------------------------------------------

gi|300715359 ------------------------------------------------------------

gi|343427335 ------------------------------------------------------------

gi|1167999 -I-------------------------------------R--------------------

gi|261369006 ------------------------------------------------------------

gi|327405610 SA-------------------------------------LEMKRGAVQ---------LLT

gi|254573952 RV-------------------------------------I--------------------

gi|251788647 ------------------------------------------------------------

gi|327309372 TI-------------------------------------K--------------------

gi|313896579 ------------------------------------------------------------

gi|342871552 TI-------------------------------------K--------------------

gi|295101693 ------------------------------------------------------------

gi|302665792 TI-------------------------------------K--------------------

gi|71023471 ------------------------------------------------------------

gi|50122255 ------------------------------------------------------------

gi|320583760 LI-------------------------------------K--------------------

gi|328351802 ------------------------------------------------------------

gi|331082383 ------------------------------------------------------------

gi|320529049 ------------------------------------------------------------

gi|255658393 ------------------------------------------------------------

gi|302500029 TI-------------------------------------K--------------------

gi|307132173 ------------------------------------------------------------

gi|357057706 ------------------------------------------------------------

gi|271501669 ------------------------------------------------------------

gi|348689589 S-----------------------------------------------------------

gi|387510768 AI-------------------------------------K--------------------

gi|260588048 ------------------------------------------------------------

gi|160943874 ------------------------------------------------------------

gi|188532978 ------------------------------------------------------------

gi|145362282 TV-------------------------------------I--------------------

gi|394757460 ------------------------------------------------------------

gi|397614365 RR-------------------------------------RLIVLASAW---------LAG

gi|170025697 ------------------------------------------------------------

gi|401624651 CI-------------------------------------R--------------------

gi|51595070 ------------------------------------------------------------

gi|257439175 ------------------------------------------------------------

gi|238926770 ------------------------------------------------------------

gi|392423567 ------------------------------------------------------------

gi|151941307 YI-------------------------------------K--------------------

gi|270263031 ------------------------------------------------------------

gi|153813543 ------------------------------------------------------------

gi|21957501 ----------------------------------------MTVQPQAE---------LAM

gi|325184913 S-----------------------------------------------------------

gi|292670864 ------------------------------------------------------------

gi|108808904 ------------------------------------------------------------

gi|154623414 SV-------------------------------------I--------------------

gi|259148132 YI-------------------------------------K--------------------

gi|402833951 ------------------------------------------------------------

gi|242240254 ------------------------------------------------------------

gi|196007934 PF-------------------------------------C--------------------

gi|391628126 ------------------------------------------------------------

gi|323308143 YI-------------------------------------K--------------------

gi|377578674 ------------------------------------------------------------

gi|402570735 ------------------------------------------------------------

gi|355378207 ------------------------------------------------------------

gi|401565378 ------------------------------------------------------------

gi|6323175 YI-------------------------------------K--------------------

gi|354559941 ------------------------------------------------------------

gi|359463278 SA-------------------------------------LELNRGQLH---------LLE

gi|295108885 ------------------------------------------------------------

gi|158319435 ------------------------------------------------------------

gi|334128073 ------------------------------------------------------------

gi|312162112 SV-------------------------------------I--------------------

gi|313114511 ------------------------------------------------------------

gi|349579866 YI-------------------------------------K--------------------

gi|116785514 SV-------------------------------------I--------------------

gi|333929035 ----------------------------------------MMLR-QFF---------MAV

gi|260887129 ------------------------------------------------------------

gi|386825267 ------------------------------------------------------------

gi|37524842 ------------------------------------------------------------

gi|198427208 KL----------------------------------------------------------

gi|291545477 ------------------------------------------------------------

gi|182418090 ------------------------------------------------------------

gi|259907494 ------------------------------------------------------------

gi|291549279 ------------------------------------------------------------

gi|157372235 ------------------------------------------------------------

gi|297845432 TV-------------------------------------I--------------------

gi|12230014 TV-------------------------------------I--------------------

gi|170032648 ------------------------------------------------------------

gi|198453209 TI-------------------------------------S--------------------

gi|284049217 ------------------------------------------------------------

gi|238754470 ------------------------------------------------------------

gi|257126567 ------------------------------------------------------------

gi|385785731 ------------------------------------------------------------

gi|238759889 ------------------------------------------------------------

gi|238750530 ------------------------------------------------------------

gi|6094336 SV-------------------------------------L--------------------

gi|332160447 ------------------------------------------------------------

gi|377851579 ------------------------------------------------------------

gi|388391237 ------------------------------------------------------------

gi|91209189 ------------------------------------------------------------

gi|169837854 ------------------------------------------------------------

gi|317046947 ------------------------------------------------------------

gi|256273016 YI-------------------------------------K--------------------

gi|218547577 ------------------------------------------------------------

gi|374579091 ------------------------------------------------------------

gi|373245798 ------------------------------------------------------------

gi|387605598 ------------------------------------------------------------

gi|187935464 ------------------------------------------------------------

gi|384470785 ------------------------------------------------------------

gi|329298049 ------------------------------------------------------------

gi|110803121 ------------------------------------------------------------

gi|212639268 IF-------------------------------------SPVVLTLCK---------LMH

gi|20807781 -------------------------------------------M----------------

gi|253988276 ------------------------------------------------------------

gi|315294649 ------------------------------------------------------------

gi|383191575 -----------------------------------------MSR----------------

gi|386228114 ------------------------------------------------------------

gi|330009512 ------------------------------------------------------------

gi|296086541 SV-------------------------------------I--------------------

gi|392297662 YI-------------------------------------K--------------------

gi|292489273 ------------------------------------------------------------

gi|225424695 SV-------------------------------------I--------------------

gi|24111564 ------------------------------------------------------------

gi|194433428 ------------------------------------------------------------

gi|333978511 -------------------------------------------M----------------

gi|378194398 ------------------------------------------------------------

gi|238791204 ------------------------------------------------------------

gi|297841771 SV-------------------------------------I--------------------

gi|15799805 ------------------------------------------------------------

gi|110799842 ------------------------------------------------------------

gi|324112468 ------------------------------------------------------------

gi|260890661 ------------------------------------------------------------

gi|401840422 YI-------------------------------------K--------------------

gi|312970216 ------------------------------------------------------------

gi|16128114 ------------------------------------------------------------

gi|386248282 ------------------------------------------------------------

gi|366159436 ------------------------------------------------------------

gi|323975716 ------------------------------------------------------------

gi|384503184 AV-------------------------------------I--------------------

gi|18309532 ------------------------------------------------------------

gi|372276039 ------------------------------------------------------------

gi|333009123 ------------------------------------------------------------

gi|332097889 ------------------------------------------------------------

gi|381405644 ------------------------------------------------------------

gi|303325156 ------------------------------------------------------------

gi|377919674 ------------------------------------------------------------

gi|300905534 ------------------------------------------------------------

gi|15223115 SI-------------------------------------I--------------------

gi|145336078 TV-------------------------------------I--------------------

gi|206578746 ------------------------------------------------------------

gi|42524959 SA-------------------------------------LEMNRGSLH---------VIQ

gi|323157811 ------------------------------------------------------------

gi|391275496 ------------------------------------------------------------

gi|195446427 LI-------------------------------------S--------------------

gi|251778321 ------------------------------------------------------------

gi|152968708 ------------------------------------------------------------

gi|371605278 ------------------------------------------------------------

gi|345395892 ------------------------------------------------------------

gi|385302583 ------------------------------------------------------------

gi|386206487 ------------------------------------------------------------

gi|238761600 ------------------------------------------------------------

gi|194746211 SL-------------------------------------N--------------------

gi|331226164 ------------------------------------------------------------

gi|340742010 ------------------------------------------------------------

gi|50552163 SI-------------------------------------K--------------------

gi|238786761 ------------------------------------------------------------

gi|386033187 ------------------------------------------------------------

gi|6094327 SI-------------------------------------L--------------------

gi|82775410 ------------------------------------------------------------

gi|110762382 AI-------------------------------------K--------------------

gi|333011508 ------------------------------------------------------------

gi|322834377 -----------------------------------------MSR----------------

gi|374992883 ------------------------------------------------------------

gi|308185706 ------------------------------------------------------------

gi|157147448 ------------------------------------------------------------

gi|12229958 SV-------------------------------------I--------------------

gi|33340515 SV-------------------------------------L--------------------

gi|295098625 ------------------------------------------------------------

gi|380021423 AI-------------------------------------K--------------------

gi|238797200 ------------------------------------------------------------

gi|293394516 ----------------------------------------MMLR-QFF---------MAV

gi|99083515 SV-------------------------------------I--------------------

gi|388608380 ------------------------------------------------------------

gi|320173653 ------------------------------------------------------------

gi|161612515 ------------------------------------------------------------

gi|378073834 ------------------------------------------------------------

gi|161504706 ------------------------------------------------------------

gi|195499508 SL-------------------------------------N--------------------

gi|2821961 ------------------------------------------------------------

gi|359376594 ---------------------------------------------------------MGK

gi|374297740 ------------------------------------------------------------

gi|239827059 ------------------------------------------------------------

gi|403383804 ------------------------------------------------------------

gi|304396607 ------------------------------------------------------------

gi|397166809 ------------------------------------------------------------

gi|123441066 ------------------------------------------------------------

gi|82542724 ------------------------------------------------------------

gi|12229957 SV-------------------------------------I--------------------

gi|376394468 ------------------------------------------------------------

gi|218687999 ------------------------------------------------------------

gi|170768493 ------------------------------------------------------------

gi|365156921 ------------------------------------------------------------

gi|195330342 SL-------------------------------------N--------------------

gi|340356265 ------------------------------------------------------------

gi|388336724 ------------------------------------------------------------

gi|188589068 ------------------------------------------------------------

gi|376400744 ------------------------------------------------------------

gi|28317069 SL-------------------------------------N--------------------

gi|389842198 ------------------------------------------------------------

gi|260596552 ------------------------------------------------------------

gi|6094335 SV-------------------------------------I--------------------

gi|365988413 LI-------------------------------------K--------------------

gi|194446066 ------------------------------------------------------------

gi|350536257 SV-------------------------------------L--------------------

gi|357485917 SV-------------------------------------I--------------------

gi|296127808 ------------------------------------------------------------

gi|74310739 ------------------------------------------------------------

gi|323166003 -----------------------------------------------M---------LAI

gi|126153731 AV-------------------------------------I--------------------

gi|291280945 ------------------------------------------------------------

gi|49425361 SV-------------------------------------I--------------------

gi|334341918 -------------------------------------------M----------------

gi|187731639 ------------------------------------------------------------

gi|156935354 ------------------------------------------------------------

gi|376377905 ------------------------------------------------------------

gi|392391837 ------------------------------------------------------------

gi|331645238 ------------------------------------------------------------

gi|378578181 ------------------------------------------------------------

gi|24645443 SL-------------------------------------N--------------------

gi|335578432 ------------------------------------------------------------

gi|374383060 ------------------------------------------------------------

gi|358248320 AV-------------------------------------I--------------------

gi|300919677 ------------------------------------------------------------

gi|194902914 SL-------------------------------------N--------------------

gi|226492509 AV-------------------------------------I--------------------

gi|391290786 ------------------------------------------------------------

lcl|84191 ---------------------DSI-----LNKVEERMKE--LSQDSTGR----VKRLPPI

gi|168177377 ---------------------DSI-----LNKVEERMKE--LSQDSTGR----VKRLPPI

gi|21264341 ---------------------DSI-----LNKVEERMKE--LSQDSTGR----VKRLPPI

gi|791051 ---------------------DSI-----LNKVEERMKE--LSQDSTGR----VKRLPPI

gi|332860397 ---------------------DSI-----LNKVEERMKE--LSQDSTGR----VKRLPPI

gi|119619392 ---------------------DSI-----LNKVEERMKE--LSQDSTGR----VKRLPPI

gi|402909680 ---------------------DSI-----LNKVEERMKE--LSQDSTGR----VKRLPPI

gi|332864823 ---------------------DSI-----LNKVEERMKE--LSQDSTGR----VKRLPPI

gi|354474372 ---------------------DSL-----LNKIEEKMKE--LSQDSTGR----VKRLPPI

gi|291407209 ---------------------DSL-----LNKVEERMKE--LSQDSTGR----VKRLPPI

gi|297303473 ---------------------DSI-----LNKVEERMKE--LSQDSTGR----VKRLPPI

gi|403263885 ---------------------DSI-----LNKVEERMKE--LSQDSTGR----VKRLPPI

gi|395753759 ---------------------DSI-----LNKVEERMKE--LSQDSTGR----VKRLPPI

gi|355704663 ---------------------DSI-----LNKVEERMKE--LSQDSTGR----VKRLPPI

gi|4164136 ---------------------DSI-----LNKVEERMKE--LSQDSTGR----VKRLPPI

gi|149030940 ---------------------DSL-----LNKIEEKMKE--LSQDSTGR----VKRLPPI

gi|76559933 ---------------------DCL-----LNKIEEKMKE--LSQDSTGR----VKRLPPI

gi|78369282 ---------------------DSL-----LNKVEERMKE--LSQDSTER----VKRLPPI

gi|118600998 ---------------------DSL-----LNKIEEKMKE--LSQDSTGR----VKRLPPI

gi|345806962 ---------------------DSL-----LNKVEERMKE--LSQDSTGR----VKRLPPI

gi|154425706 ---------------------DSL-----LNKVEERMKE--LSQDSTER----VKRLPPI

gi|26340516 ---------------------DSL-----LNKIEEKMKE--LSQDSTGR----VKRLPPI

gi|384948950 ---------------------DSI-----LNKVEERMKE--LSQDSTGR----VKRLPPI

gi|397497639 ---------------------DSI-----LNKVEERMKE--LSQDSTGR----VKRLPPI

gi|348561339 ---------------------DNL-----LNKVEERMKE--LSQASIGR----VKRLPTI

gi|354473432 ---------------------DSL-----LNKIEEKMKE--LGPDSTVR----AKRLPPI

gi|344288661 ---------------------DSL-----LNKVEERMKE--LSQDFTGR----VKRLPPL

gi|338728955 ---------------------DSL-----LNKVEERMKE--LSQDSTGR----VKRLPPI

gi|71895235 ---------------------DQL-----LNKVEERMKE--LFHGNLKR----VKRLPAI

gi|297678474 ---------------------DSI-----LNKVEERMKE--LSQDSTGR----VKRLPPI

gi|301756254 ---------------------DSL-----LNKVEERMKE--LSQDSTVR----VKRLPPI

gi|296470508 ---------------------DSL-----LNKVEERMKE--LSQDSTER----VKRLPPI

gi|351712477 ---------------------DNL-----LNKVEEKMKE--LSQASIGR----VKRLPTI

gi|344242683 ---------------------DSL-----LNKIEEKMKE--LSQDSTGR----VKRLPPI

gi|387018788 ---------------------EQL-----LNKVEEKMKE--LFHKSIKR----IKRLPVI

gi|327268286 ---------------------GQL-----LNKVEERMKE--LFHRNIKR----VKGLPAI

gi|334329503 ---------------------DNL-----LNKLEERMKE--LCQGNIGR----LKRLPAL

gi|350595577 ---------------------DSL-----LNKVEERMKE--LSQDSTER----VKRLPPI

gi|351706018 ---------------------DNL-----LNKVEERMKE--LSQASIEQ----VKCLPTI

gi|395518817 ---------------------DNL-----LNKLEERMKE--LCEGSIAR----LKRLPAL

gi|149042406 ---------------------DCL-----LNKIEEKMKE--LSQDSTGR----VKRLPPI

gi|326913546 ---------------------DQL-----LNKVEERMKE--FFHSNLKR----VKRLPAI

gi|224042691 ---------------------DQL-----LNKVEERMKE--LIHGNIKR----VKRLPAI

gi|148708890 ---------------------DSL-----LNKIEEKMKE--LSQDSTGR----VKRLPPI

gi|345327044 ---------------------NNL-----LNKVEERMKE--LYPGCISR----VKRLPAI

gi|385862226 ---------------------HKI-----LSKIEEKMQE--ILHNSIRQ----VKRLPAI

gi|354482601 ---------------------DSL-----LNKLEEKMKE--LSQDSIVR----VKRLPPI

gi|281352943 ---------------------DSL-----LNKVEERMKE--LSQDSTVR----VKRLPPI

gi|18858203 ---------------------DNL-----LNALEKKLKA--LLDGNIQR----IKRLPAL

gi|395838143 ---------------------DRL-----LNKVEERMKE--LSQDSTGR----VKRLPPI

gi|213511272 ---------------------ENL-----LNTLEEKLRS--LLHGNIKR----VKRLPAL

gi|348518728 ---------------------DNL-----LNALENKLKV--LLNGNITR----IKKLPAL

gi|386643030 -------------------------------------------------------RLPPI

gi|402909682 -------------------------------------------------------RLPPI

gi|4007861 ---------------------DNL-----LNALEKKLKT--LLNGNIKR----IKRLPAL

gi|351706975 ---------------------DNL-----LNKVEKRMKE--LSQASIGR----VKCLPTI

gi|4007046 ---------------------DNL-----LNALEKKLKA--LLSGNIKR----FKRLPAL

gi|114687978 ---------------------DSI-----LNKVEERMKE--LSQDSTGR----VKRLPPI

gi|2916761 ---------------------DSL-----LNKIEEKMKE--LSQDSTGR----VKRLPPI

gi|194373711 ------------------------------------MKE--LSQDSTGR----VKRLPPI

gi|332860399 ------------------------------------MKE--LSQDSTGR----VKRLPPI

gi|125978661 ------------------------------RTMELILRQKL---D-SDRS----KYLPPI

gi|225581217 ------------------------------RTMELILRQKL---D-SDRS----KYLPPI

gi|195376249 ----------------------------TMRTMELILRQKL---D-SDRS----KYLPPI

gi|195126140 ----------------------------TMRTMELILRQKL---D-SDRS----KYLPPI

gi|194747133 ----------------------------TMRTMELILRQKF---D-SDRS----KYLPPI

gi|195012869 ----------------------------TMRTMELILRQKL---D-SDRS----KYLPPI

gi|24663238 ----------------------------TMRTMELILRQKF---D-SDRS----KYLPPI

gi|194869781 ----------------------------TMRTMELILRQKF---E-SDRS----KYLPPI

gi|195327091 ----------------------------TMRTMELILRQKF---D-SDRS----KYLPPI

gi|390176333 -----HG-----------------------KSLENALAVKL---K-AIRG----QKLPAL

gi|21358269 ----------------------------SGKSLENVLAIKL---K-AIKA----QKLPTL

gi|74211624 ---------------------DSL-----LNKIEEKMKE--LSQDSTGR----VKRLPPI

gi|195589784 ----------------------------TMRTMELILRQKF---D-SDRS----KYLPPI

gi|289742457 ----------------------------SSKRLENTIAKKL---K-VTSG----QVLPTL

gi|157118336 ----------------------------QTRELENSLVQNL---K-LNHG----QSLPTL

gi|158293995 ----------------------------QIRELENNLVQKL---K-FNHG----QSLPPL

gi|170032109 ----------------------------QTRELENSLVQNL---K-LNHG----QSLPAL

gi|327290877 --------------------------------------------------------LPAI

gi|4927220 ---------------------DSL-----LNKIEEKMKE--LSQDSTGR----VKRLPPI

gi|195493801 CRIFQHGLLTLNVEYFLPDGKEPIISFDTMRTMELILRQKF---D-SDRS----KYLPPI

gi|242024750 ----------------------------QLRLLELSIGKAL---A-SARS----KHLPPI

gi|312372613 ----------------------------NMKSLENGLRIRL---E-AKRS----KHLPPI

gi|119619391 ---------------------DSI-----LNKVEERMKE--LSQDSTGR----VKRLPPI

gi|260818384 ----------------------SA---------QQKISELL----GCKKS----KRFPFI

gi|340709405 ----------------------------LARELETALQAAV---A-STRS----HSLTPI

gi|350407163 ----------------------------LARELETALQVAV---A-STRS----HSLTPI

gi|383856563 ----------------------------QWRYLEADVAMAL---N-SQRS----KRLPPV

gi|357613964 ----------------------------NTKLLENSVKISL---E-CERI----KLMPTI

gi|156542873 ----------------------------QWRDLEVDVAMAL---N-SQRS----KRLPPV

gi|332025522 ----------------------------QWRYLEADVAMAL---N-SQRS----KRLPPV

gi|110750077 ----------------------------QWRYLEADVAMAL---N-SQRS----KRLPPV

gi|91085293 ----------------------------RCRELDRDLVERV---VGIHRS----QALVPI

gi|307188353 ----------------------------QLRYLEADVAMAL---N-SQRS----KHLPPI

gi|390338494 ---------------------PVL-----EEGSMEALKEELKKVFNPKK----CKLFPEI

gi|195440770 ----------------------------TIRTLELILRQKL---D-SDRS----KYLPPI

gi|380023489 ----------------------------QWRYLEADVAMAL---N-SQRS----KRLPPV

gi|156394332 ---------------------NNR-----YEES-KAIKESLAKVFATEK----SRALPPI

gi|167538084 ----------------RATATESL-----K---AAPVQHLD-----------ATTLYPPV

gi|326435685 TCGGSEA----SAEASTATSTRSS-----SNTVPSSAQHNEPSEDNETQTSVSAKVFPPI

gi|47227186 ---------------------DNL-----LNALEKKLKT--LLNGNIKR----IKRLPAL

gi|321461882 ---------------------------EASKKFETDVKEKL---A-VVAS----KTICPI

gi|344241635 ------------------------------------------------------------

gi|389613513 ------------------------------------------------------------

gi|313219992 IQWYPGA----AGKLFATVSGPAR-----VEQIASLLKKELEANKATSTVF-----TPPL

gi|313225743 IQWYPGA----AGKLFATVSGPAR-----VEQIASLLKKELEANKATSTVF-----TPPL

gi|198418193 NISSVLIQGMRDGVLTFDLHLLSMVEPEVFDKKVLTFHDKLLEHEGIVRRK--TSEFEPL

gi|391343942 ---------------------ECF-----RKQLQKELEM---------R----VVRVPHL

gi|196014283 ---------------------------------------------------------PAI

gi|307204802 ----------------------------HLRRL---------------RS----KLIKPH

gi|225718096 ---------------------GNL-----QHEL------SNRMVTDERN----FRSLGCL

gi|324096424 ------------------------------------------------------------

gi|327352193 -------------------------------------------------D----------

gi|226293104 -------------------------------------------------D----------

gi|154277382 -------------------------------------------------D----------

gi|212532035 -------------------------------------------------D----------

gi|242117904 -------------------------------------------------D----------

gi|134077286 -------------------------------------------------D----------

gi|348570938 -------------------------------------------------E----------

gi|389628860 -------------------------------------------------D----------

gi|119495240 -------------------------------------------------D----------

gi|67516811 -------------------------------------------------D----------

gi|242774578 -------------------------------------------------D----------

gi|225683737 -------------------------------------------------D----------

gi|255716758 -------------------------------------------------D----------

gi|395218408 RNDFNLETLRDATA-KTIEA----DGS-------------------ITRD----------

gi|348514634 -------------------------------------------------D----------

gi|347828643 -------------------------------------------------D----------

gi|361131873 -------------------------------------------------D----------

gi|392864754 -------------------------------------------------D----------

gi|115492019 -------------------------------------------------D----------

gi|212532039 -------------------------------------------------D----------

gi|340914898 -------------------------------------------------D----------

gi|156044358 -------------------------------------------------D----------

gi|342887616 -------------------------------------------------D----------

gi|302307177 -------------------------------------------------D----------

gi|367038823 -------------------------------------------------D----------

gi|358371985 -------------------------------------------------D----------

gi|317030551 -------------------------------------------------D----------

gi|303318439 -------------------------------------------------D----------

gi|85100119 -------------------------------------------------D----------

gi|380088740 -------------------------------------------------D----------

gi|395840954 -------------------------------------------------E----------

gi|16758208 -------------------------------------------------E----------

gi|327287238 -------------------------------------------------E----------

gi|146414243 -------------------------------------------------D----------

gi|190348490 -------------------------------------------------D----------

gi|367025809 -------------------------------------------------D----------

gi|121701283 -------------------------------------------------D----------

gi|119592082 -------------------------------------------------E----------

gi|70995928 -------------------------------------------------D----------

gi|6678131 -------------------------------------------------E----------

gi|531202 -------------------------------------------------E----------

gi|114553926 -------------------------------------------------E----------

gi|63253298 -------------------------------------------------E----------

gi|122921244 -------------------------------------------------E----------

gi|116197943 -------------------------------------------------N----------

gi|378733877 -------------------------------------------------D----------

gi|26390475 -------------------------------------------------E----------

gi|402852915 -------------------------------------------------E----------

gi|347447556 -------------------------------------------------E----------

gi|383873318 -------------------------------------------------E----------

gi|213409616 -------------------------------------------------D----------

gi|32766339 -------------------------------------------------D----------

gi|387515006 -------------------------------------------------D----------

gi|46137467 -------------------------------------------------D----------

gi|320583104 -------------------------------------------------D----------

gi|350585594 -------------------------------------------------E----------

gi|315053415 -------------------------------------------------D----------

gi|322706886 -------------------------------------------------D----------

gi|402082530 -------------------------------------------------D----------

gi|317137159 -------------------------------------------------D----------

gi|147902014 -------------------------------------------------D----------

gi|344283521 -------------------------------------------------E----------

gi|76637216 -------------------------------------------------E----------

gi|255937259 -------------------------------------------------D----------

gi|366998257 -------------------------------------------------D----------

gi|320588782 -------------------------------------------------D----------

gi|254580735 -------------------------------------------------D----------

gi|301620173 -------------------------------------------------D----------

gi|258572568 -------------------------------------------------D----------

gi|225710012 ---------------------GNL-----SRVL------SEGLISNERD----LQFIGCL

gi|19112807 -------------------------------------------------D----------

gi|398411809 -------------------------------------------------D----------

gi|379728455 RVNIDHLLKERQGTESQLEH----KVK-------------------FSRN----------

gi|149239971 -------------------------------------------------D----------

gi|119175541 -------------------------------------------------D----------

gi|320039089 -------------------------------------------------D----------

gi|74138635 -------------------------------------------------E----------

gi|238489149 -------------------------------------------------D----------

gi|255725052 -------------------------------------------------D----------

gi|322701141 -------------------------------------------------D----------

gi|359378073 -------------------------------------------------D----------

gi|358378881 -------------------------------------------------D----------

gi|50286095 -------------------------------------------------D----------

gi|358391288 -------------------------------------------------D----------

gi|401623242 -------------------------------------------------D----------

gi|6325326 -------------------------------------------------D----------

gi|151942852 -------------------------------------------------D----------

gi|302912624 -------------------------------------------------D----------

gi|261190484 ------------------------------------------------------------

gi|336262713 -------------------------------------------------D----------

gi|296814308 -------------------------------------------------D----------

gi|126138914 -------------------------------------------------D----------

gi|50420767 -------------------------------------------------D----------

gi|68482416 -------------------------------------------------D----------

gi|169615146 -------------------------------------------------D----------

gi|396485345 -------------------------------------------------D----------

gi|241951762 -------------------------------------------------D----------

gi|400597126 -------------------------------------------------D----------

gi|345561897 -------------------------------------------------D----------

gi|310793583 -------------------------------------------------D----------

gi|340520373 -------------------------------------------------D----------

gi|47228825 -------------------------------------------------E----------

gi|156843587 -------------------------------------------------D----------

gi|330907629 -------------------------------------------------D----------

gi|189207669 -------------------------------------------------D----------

gi|50554613 -------------------------------------------------D----------

gi|375086054 ------------------------------------------------MD----------

gi|366996717 -------------------------------------------------D----------

gi|380353232 -------------------------------------------------D----------

gi|154304795 -------------------------------------------------N----------

gi|171693823 -------------------------------------------------D----------

gi|260781328 -----------------------------------------------------LSRLPFL

gi|344234284 -------------------------------------------------D----------

gi|260951089 -------------------------------------------------D----------

gi|367012209 -------------------------------------------------D----------

gi|403214093 -------------------------------------------------D----------

gi|354548134 -------------------------------------------------D----------

gi|372463636 -------------------------------------------------D----------

gi|284929129 RIDIDLGELRDETTNKLV------IPK-------------------ISRS----------

gi|291533315 ------------------------------------------------MD----------

gi|384501417 -------------------------------------------------D----------

gi|401838282 -------------------------------------------------D----------

gi|344302668 -------------------------------------------------D----------

gi|365982711 -------------------------------------------------D----------

gi|296421657 -------------------------------------------------D----------

gi|149695532 -------------------------------------------------M----------

gi|50307673 -------------------------------------------------D----------

gi|50303991 -------------------------------------------------D----------

gi|380483699 ------------------------------------------------------------

gi|325660851 ------------------------------------------------ME----------

gi|160932658 ------------------------------------------------ME----------

gi|363751094 -------------------------------------------------D----------

gi|254569166 -------------------------------------------------D----------

gi|268612001 ------------------------------------------------ME----------

gi|225018465 ------------------------------------------------ME----------

gi|326427880 -------------------------------------------------K----------

gi|297617720 ---------ESMVDLW---------------------------------N----------

gi|295665965 -------------------------------------------------D----------

gi|317131721 ------------------------------------------------ME----------

gi|346467789 SRSG-QPAARTLLETSMN----------------------------VMKE----------

gi|328766899 -------------------------------------------------G----------

gi|323486581 ------------------------------------------------MN----------

gi|326476682 -------------------------------------------------D----------

gi|164660446 -----------MSNGNVVLT----HPN-------------------I-HD----------

gi|169351543 ------------------------------------------------ME----------

gi|346326304 ATPPLLPLLIGRSEGR--------------------------------KD----------

gi|167519370 -------------------------------------------------K----------

gi|321468222 -------------------------------------------------N----------

gi|404329717 ------------------------------------------------ME----------

gi|283798449 ------------------------------------------------ME----------

gi|300121366 -----------------------------------------------IEN----------

gi|227112962 -----------------M----------------------------SQKE----------

gi|388582960 -----------MS-----LT----HPA-------------------VDNN----------

gi|225713606 ---------------------GNL-----KSNL------SCRMGLTLKD----VHLVGSF

gi|346466755 SHSG-QPAARTLLETSMN----------------------------VMKE----------

gi|355720872 ------------------------------------------------------------

gi|167755034 ------------------------------------------------ME----------

gi|193215178 KQ-YNIPVFGEETPQREMQY----SPQ-------------------YARN----------

gi|403059603 -----------------M----------------------------SQKE----------

gi|388854692 -----------MG----VIT----HPN-------------------I-RD----------

gi|227329706 -----------------M----------------------------SQKE----------

gi|253689498 -----------------M----------------------------SQKE----------

gi|300715359 -----------------M----------------------------AEKE----------

gi|343427335 -----------MG----VIT----HPN-------------------I-RD----------

gi|1167999 -------------------------------------------------E----------

gi|261369006 ------------------------------------------------ME----------

gi|327405610 RNDFDISSMRQKAG-EWSN-----PEM-------------------YTRN----------

gi|254573952 -------------------------------------------------D----------

gi|251788647 -----------------M----------------------------SRKE----------

gi|327309372 -------------------------------------------------D----------

gi|313896579 ------------------------------------------------ME----------

gi|342871552 -------------------------------------------------D----------

gi|295101693 ------------------------------------------------ME----------

gi|302665792 -------------------------------------------------D----------

gi|71023471 -----------MG----VIT----HPN-------------------I-RD----------

gi|50122255 -----------------M----------------------------SQKE----------

gi|320583760 -------------------------------------------------D----------

gi|328351802 ------------------------------------------------------------

gi|331082383 ------------------------------------------------MD----------

gi|320529049 ------------------------------------------------ME----------

gi|255658393 ------------------------------------------------ME----------

gi|302500029 -------------------------------------------------D----------

gi|307132173 -----------------M----------------------------SRKE----------

gi|357057706 ------------------------------------------------ME----------

gi|271501669 -----------------M----------------------------SRKE----------

gi|348689589 -----------------------------------------------TEK----------

gi|387510768 -------------------------------------------------D----------

gi|260588048 ------------------------------------------------MD----------

gi|160943874 ------------------------------------------------ME----------

gi|188532978 -----------------M----------------------------ATNE----------

gi|145362282 -------------------------------------------------P----------

gi|394757460 ------------------------------------------------MD----------

gi|397614365 QINSHRMVEFPPPKDYSH----------------------------LLVD----------

gi|170025697 -----------------M----------------------------SQKE----------

gi|401624651 -------------------------------------------------D----------

gi|51595070 -----------------M----------------------------SQKE----------

gi|257439175 ------------------------------------------------ME----------

gi|238926770 ------------------------------------------------ME----------

gi|392423567 ------------------------------------------------ME----------

gi|151941307 -------------------------------------------------D----------

gi|270263031 -----------------M----------------------------TQKE----------

gi|153813543 ----------------------------------------------MTME----------

gi|21957501 ILPPIFHGCYCEGEAPNM----------------------------SQKE----------

gi|325184913 -----------------------------------------------DTK----------

gi|292670864 -----------------------------------------------MME----------

gi|108808904 -----------------M----------------------------SQKE----------

gi|154623414 -------------------------------------------------P----------

gi|259148132 -------------------------------------------------D----------

gi|402833951 ------------------------------------------------MN----------

gi|242240254 -----------------M----------------------------SRKE----------

gi|196007934 -------------------------------------------------D----------

gi|391628126 -----------------M----------------------------SQKE----------

gi|323308143 -------------------------------------------------D----------

gi|377578674 -----------------M----------------------------AEAK----------

gi|402570735 ------------------------------------------------ME----------

gi|355378207 -----------------------------------------------MME----------

gi|401565378 ------------------------------------------------ME----------

gi|6323175 -------------------------------------------------D----------

gi|354559941 ------------------------------------------------ME----------

gi|359463278 TEDVDFKRPEDVAAYEYS------DPQ-------------------EKRT----------

gi|295108885 ------------------------------------------------ME----------

gi|158319435 ------------------------------------------------ME----------

gi|334128073 ------------------------------------------------ME----------

gi|312162112 -------------------------------------------------P----------

gi|313114511 ----------------------------------------------MQME----------

gi|349579866 -------------------------------------------------D----------

gi|116785514 -------------------------------------------------P----------

gi|333929035 IT-----------EFPHM----------------------------TQKE----------

gi|260887129 ------------------------------------------------MN----------

gi|386825267 -----------------M----------------------------TQKE----------

gi|37524842 -----------------M----------------------------SQKE----------

gi|198427208 ------------------------------------------------------------

gi|291545477 ------------------------------------------------ME----------

gi|182418090 ------------------------------------------------ME----------

gi|259907494 -----------------M----------------------------AANE----------

gi|291549279 ------------------------------------------------ME----------

gi|157372235 -----------------M----------------------------TQKE----------

gi|297845432 -------------------------------------------------P----------

gi|12230014 -------------------------------------------------P----------

gi|170032648 -------------------------------------------------S----------

gi|198453209 -------------------------------------------------C----------

gi|284049217 ------------------------------------------------ME----------

gi|238754470 -----------------M----------------------------SQKE----------

gi|257126567 ------------------------------------------------ME----------

gi|385785731 -----------------M----------------------------AANE----------

gi|238759889 -----------------M----------------------------SQEE----------

gi|238750530 -----------------M----------------------------SQKE----------

gi|6094336 -------------------------------------------------P----------

gi|332160447 -----------------M----------------------------SQKE----------

gi|377851579 -----------------M----------------------------AEKK----------

gi|388391237 -----------------M----------------------------AEKK----------

gi|91209189 -----------------M----------------------------AEKK----------

gi|169837854 ------------------------------------------------ME----------

gi|317046947 -----------------M----------------------------AQNE----------

gi|256273016 -------------------------------------------------D----------

gi|218547577 -----------------M----------------------------AEKK----------

gi|374579091 --------MNIKLPA----Y----FSK-------------------KGHD----------

gi|373245798 -----------------M----------------------------AEKK----------

gi|387605598 -----------------M----------------------------AEKK----------

gi|187935464 ------------------------------------------------ME----------

gi|384470785 -----------------M----------------------------AEKK----------

gi|329298049 -----------------M----------------------------ANKE----------

gi|110803121 ------------------------------------------------ME----------

gi|212639268 KCINIKKVQNTLLEEEAMNY----IKE-------------------QNGQ----------

gi|20807781 -------------------------------------------------E----------

gi|253988276 -----------------M----------------------------SQKE----------

gi|315294649 -----------------M----------------------------AEKK----------

gi|383191575 ----------------NS----------------------------SSKE----------

gi|386228114 -----------------M----------------------------AEKK----------

gi|330009512 -----------------M----------------------------ADNP----------

gi|296086541 -------------------------------------------------P----------

gi|392297662 -------------------------------------------------D----------

gi|292489273 -----------------M----------------------------TANE----------

gi|225424695 -------------------------------------------------P----------

gi|24111564 -----------------M----------------------------AEKK----------

gi|194433428 -----------------M----------------------------AEKK----------

gi|333978511 -------------------------------------------------N----------

gi|378194398 -----------------M----------------------------AEKK----------

gi|238791204 -----------------M----------------------------SQKE----------

gi|297841771 -------------------------------------------------P----------

gi|15799805 -----------------M----------------------------AEKK----------

gi|110799842 ------------------------------------------------ME----------

gi|324112468 -----------------M----------------------------AENK----------

gi|260890661 ------------------------------------------------ME----------

gi|401840422 -------------------------------------------------D----------

gi|312970216 -----------------M----------------------------AEKK----------

gi|16128114 -----------------M----------------------------AEKK----------

gi|386248282 -----------------M----------------------------AEKK----------

gi|366159436 -----------------M----------------------------AEKK----------

gi|323975716 -----------------M----------------------------AEKK----------

gi|384503184 -------------------------------------------------P----------

gi|18309532 ------------------------------------------------ME----------

gi|372276039 -----------------M----------------------------AHNE----------

gi|333009123 ---------MLTKEVSTH----------------------------GRKK----------

gi|332097889 ---------MLTKEVSTH----------------------------GRKK----------

gi|381405644 -----------------M----------------------------AHNE----------

gi|303325156 -----------HHHHHHM----------------------------AEKK----------

gi|377919674 ---------MLTKEVSTH----------------------------GRKK----------

gi|300905534 -----------------M----------------------------AEKK----------

gi|15223115 -------------------------------------------------P----------

gi|145336078 -------------------------------------------------P----------

gi|206578746 -----------------M----------------------------ADNP----------

gi|42524959 KSDFQPKIMREKPSFDLSK-----GHQ-------------------IDRN----------

gi|323157811 ---------MLTKEVSTH----------------------------GRKK----------

gi|391275496 ---------MLTKEVSTH----------------------------GRKK----------

gi|195446427 -------------------------------------------------N----------

gi|251778321 ------------------------------------------------ME----------

gi|152968708 -----------------M----------------------------ADNP----------

gi|371605278 -----------------M----------------------------AEKK----------

gi|345395892 ---------MLTKEVSTH----------------------------GRKK----------

gi|385302583 ------------------------------------------------------------

gi|386206487 -----------------M----------------------------AEKK----------

gi|238761600 -----------------M----------------------------SQKE----------

gi|194746211 -------------------------------------------------N----------

gi|331226164 -----------MAT---TLT----HPA-------------------I-KD----------

gi|340742010 -----------------M----------------------------AEKK----------

gi|50552163 -------------------------------------------------N----------

gi|238786761 -----------------M----------------------------SQKE----------

gi|386033187 -----------------M----------------------------ADNP----------

gi|6094327 -------------------------------------------------P----------

gi|82775410 -----------------M----------------------------AEKK----------

gi|110762382 -------------------------------------------------P----------

gi|333011508 -----------------M----------------------------AEKK----------

gi|322834377 ----------------NS----------------------------SSKE----------

gi|374992883 ------------------------------------------------ME----------

gi|308185706 -----------------M----------------------------AHNE----------

gi|157147448 -----------------M----------------------------AENT----------

gi|12229958 -------------------------------------------------P----------

gi|33340515 -------------------------------------------------P----------

gi|295098625 -----------------M----------------------------TEKT----------

gi|380021423 -------------------------------------------------P----------

gi|238797200 -----------------M----------------------------SQKE----------

gi|293394516 IT-----------EFPDM----------------------------TQKD----------

gi|99083515 -------------------------------------------------P----------

gi|388608380 -----------------M----------------------------TEKT----------

gi|320173653 -----------------M----------------------------AEKK----------

gi|161612515 -----------------M----------------------------AENT----------

gi|378073834 -----------------M----------------------------AEKK----------

gi|161504706 -----------------M----------------------------AENT----------

gi|195499508 -------------------------------------------------N----------

gi|2821961 ------------------------------------------------------------

gi|359376594 IEAQNIDLASFKHSSVYK----------------------------LDDQ----------

gi|374297740 ------------------------------------------------MD----------

gi|239827059 ----MKHVQNVLPP-----Y----VKV-------------------QNGE----------

gi|403383804 ------------------------------------------------ME----------

gi|304396607 -----------------M----------------------------AQNE----------

gi|397166809 -----------------M----------------------------ADNT----------

gi|123441066 -----------------M----------------------------SQKE----------

gi|82542724 -----------------M----------------------------AEKK----------

gi|12229957 -------------------------------------------------P----------

gi|376394468 -----------------M----------------------------ADSN----------

gi|218687999 -----------------M----------------------------AEKK----------

gi|170768493 -----------------M----------------------------AEKK----------

gi|365156921 --------MNTNLE-----W----LQE-------------------IDGS----------

gi|195330342 -------------------------------------------------N----------

gi|340356265 ------------------------------------------------MN----------

gi|388336724 -----------------M----------------------------AEKK----------

gi|188589068 ------------------------------------------------ME----------

gi|376400744 -----------------M----------------------------ADNN----------

gi|28317069 -------------------------------------------------N----------

gi|389842198 -----------------M----------------------------TENP----------

gi|260596552 -----------------M----------------------------TENP----------

gi|6094335 -------------------------------------------------P----------

gi|365988413 -------------------------------------------------D----------

gi|194446066 -----------------M----------------------------AENT----------

gi|350536257 -------------------------------------------------P----------

gi|357485917 -------------------------------------------------P----------

gi|296127808 ------------------------------------------------ME----------

gi|74310739 -----------------M----------------------------AEKK----------

gi|323166003 MLRPFLR--VLTKEVSTH----------------------------GRKK----------

gi|126153731 -------------------------------------------------P----------

gi|291280945 -----------------M----------------------------AEKK----------

gi|49425361 -------------------------------------------------P----------

gi|334341918 -------------------------------------------------E----------

gi|187731639 -----------------M----------------------------AEKK----------

gi|156935354 ------------------------------------------------------------

gi|376377905 -----------------M----------------------------ADSN----------

gi|392391837 ------------------------------------------------ME----------

gi|331645238 -----------------M----------------------------AEKK----------

gi|378578181 -----------------M----------------------------AQNE----------

gi|24645443 -------------------------------------------------N----------

gi|335578432 ---------MLTKEVSTH----------------------------GRKK----------

gi|374383060 ------------------------------------------------ME----------

gi|358248320 -------------------------------------------------P----------

gi|300919677 -----------------M----------------------------AEKK----------

gi|194902914 -------------------------------------------------N----------

gi|226492509 -------------------------------------------------P----------

gi|391290786 ---------MLTKEVSTH----------------------------GRKK----------

lcl|84191 VRG----GAI--D--RYWP---TA------------------------------------

gi|168177377 VRG----GAI--D--RYWP---TA------------------------------------

gi|21264341 VRG----GAI--D--RYWP---TA------------------------------------

gi|791051 VRG----GAI--D--RYWP---TA------------------------------------

gi|332860397 VRG----GAI--D--RYWP---TA------------------------------------

gi|119619392 VRG----GAI--D--RYWP---TA------------------------------------

gi|402909680 VRG----GAI--D--RYWP---TA------------------------------------

gi|332864823 VRG----GAI--D--RYWP---TA------------------------------------

gi|354474372 VRG----GAF--D--RYWP---TA------------------------------------

gi|291407209 VRG----GAI--D--RYWP---TA------------------------------------

gi|297303473 VRG----GAI--D--RYWP---TA------------------------------------

gi|403263885 VRG----GAI--D--RYWP---TA------------------------------------

gi|395753759 VRG----GAI--D--RYWP---TA------------------------------------

gi|355704663 VRG----GAI--D--RYWP---TA------------------------------------

gi|4164136 VRG----GAI--D--RYWP---TA------------------------------------

gi|149030940 VRG----GAI--D--RYWP---TA------------------------------------

gi|76559933 VRG----GAI--D--RYWP---TA------------------------------------

gi|78369282 VRG----GAI--D--RYWP---TA------------------------------------

gi|118600998 VRG----GAI--D--RYWP---TA------------------------------------

gi|345806962 VRG----GAI--D--RYWP---TA------------------------------------

gi|154425706 VRG----GAI--D--RYWP---TA------------------------------------

gi|26340516 VRG----GAI--D--RYWP---TA------------------------------------

gi|384948950 VRG----GAI--D--RYWP---TA------------------------------------

gi|397497639 VRG----GAI--D--RYWP---TA------------------------------------

gi|348561339 VRG----GAI--D--RYWP---TA------------------------------------

gi|354473432 VRG----GAI--D--RYWP---TA------------------------------------

gi|344288661 VRG----GAI--D--RYWP---TA------------------------------------

gi|338728955 VRG----GAI--D--RYWP---TA------------------------------------

gi|71895235 LRG----GVI--D--RYWP---TA------------------------------------

gi|297678474 VRG----EAI--D--RYWP---TA------------------------------------

gi|301756254 VRG----GAI--D--RYWP---TA------------------------------------

gi|296470508 VRG----GAI--D--RYWP---TA------------------------------------

gi|351712477 VRG----GAI--D--RYWP---TA------------------------------------

gi|344242683 VRG----GAF--D--RYWP---TA------------------------------------

gi|387018788 MRG----DAI--D--RYWP---TA------------------------------------

gi|327268286 VRG----DTI--D--RYWP---TA------------------------------------

gi|334329503 VRG----GKI--D--KYWP---TA------------------------------------

gi|350595577 VRG----GAI--D--RYWP---TA------------------------------------

gi|351706018 VQR----GAI--D--RYWP---TP------------------------------------

gi|395518817 VRG----GKI--D--KYWP---TA------------------------------------

gi|149042406 VRG----GAI--D--RYWP---TA------------------------------------

gi|326913546 LRG----GVI--D--RYWP---TA------------------------------------

gi|224042691 LRG----GVI--D--RYWP---TA------------------------------------

gi|148708890 VRG----GAI--D--RYWP---TA------------------------------------

gi|345327044 TRG----GAV--D--RYWP---TA------------------------------------

gi|385862226 VRG----ASV--D--RYWP---TA------------------------------------

gi|354482601 VRG----GAI--D--RYWP---TA------------------------------------

gi|281352943 VRG----GAI--D--RYWP---TA------------------------------------

gi|18858203 IRG----SDV--D--RYWP---TA------------------------------------

gi|395838143 VRG----GAI--D--RYWP---TA------------------------------------

gi|213511272 TRG----AAV--D--RYWP---TA------------------------------------

gi|348518728 LRG----AKV--D--RYWP---TA------------------------------------

gi|386643030 VRG----GAI--D--RYWP---TA------------------------------------

gi|402909682 VRG----GAI--D--RYWP---TA------------------------------------

gi|4007861 IRG----AEV--D--RYWP---TS------------------------------------

gi|351706975 VQG----GAI--D--RYWP---TA------------------------------------

gi|4007046 IRG----AEV--D--RYWP---TS------------------------------------

gi|114687978 VRG----GAI--D--RYWP---TA------------------------------------

gi|2916761 VRG----GAI--D--RYWP---TA------------------------------------

gi|194373711 VRG----GAI--D--RYWP---TA------------------------------------

gi|332860399 VRG----GAI--D--RYWP---TA------------------------------------

gi|125978661 KR-----GGY-ID--IYMT---SS------------------------------------

gi|225581217 KR-----GGY-ID--IYMT---SS------------------------------------

gi|195376249 KR-----GGY-ID--IYMT---SS------------------------------------

gi|195126140 KR-----GGY-ID--IYMT---SS------------------------------------

gi|194747133 KR-----GGY-ID--NYMT---ST------------------------------------

gi|195012869 KR-----GGY-ID--IYMT---SS------------------------------------

gi|24663238 KR-----GGY-ID--IYMT---SS------------------------------------

gi|194869781 KR-----GGY-ID--IYMT---SS------------------------------------

gi|195327091 KR-----GGY-ID--IYMT---SS------------------------------------

gi|390176333 RR-----GD--VP--RYFP---SS------------------------------------

gi|21358269 KR-----GD--VS--RYFP---SS------------------------------------

gi|74211624 VRG----GAI--D--RYWP---TA------------------------------------

gi|195589784 KR-----GGY-ID--IYMT---SSEFEPPPKLCTSPKPPDHSGKSLENVLAIKLKAIKAQ

gi|289742457 KR-----GA--VA--RYFP---SS------------------------------------

gi|157118336 QR-----GP--IT--KYFP---TA------------------------------------

gi|158293995 QR-----GP--LT--RYFP---TA------------------------------------

gi|170032109 QR-----GP--VT--KYFP---TA------------------------------------

gi|327290877 VRG----DTI--D--RYWP---TA------------------------------------

gi|4927220 VRG----GAI--D--RYWP---TA------------------------------------

gi|195493801 KR-----GGY-ID--IYMT---SSALH-------IAKATDHSGKSLENVLAIKLKAIKAQ

gi|242024750 KRGGAIDVYL--------T---TS------------------------------------

gi|312372613 KR-----GSN-VD--VYLT---SS------------------------------------

gi|119619391 VRG----GAI--D--RYWP---TA------------------------------------

gi|260818384 KRG----ARV--D--PYVP---TA------------------------------------

gi|340709405 KR-----GGP-FE--RYFP---TA------------------------------------

gi|350407163 KR-----GGP-FE--RYFP---TA------------------------------------

gi|383856563 RR-----GTV-YD--LYLT---LS------------------------------------

gi|357613964 KR-----GYK------YDS---TKLLE------------SQLQKYIT-V-------IKSQ

gi|156542873 RR-----GTI-YD--LYLT---LS------------------------------------

gi|332025522 RR-----GTI-YD--LYLT---LS------------------------------------

gi|110750077 RR-----GTI-YD--LYLT---LS------------------------------------

gi|91085293 KR-----GT--FK--RYYP---TS------------------------------------

gi|307188353 RR-----GTI-YD--LYLT---LS------------------------------------

gi|390338494 SRG----AAV--D--RYIP---TA------------------------------------

gi|195440770 KR-----GGY-ID--IYMT---SS------------------------------------

gi|380023489 RR-----GTI-YD--LYLT---LS------------------------------------

gi|156394332 IRK----GPL--N--PYLP---TV------------------------------------

gi|167538084 VRG----GSF--P--TVVP---SS------------------------------------

gi|326435685 VRG----GAF--D--PYVP---SA------------------------------------

gi|47227186 IRG----AEV--D--RYWP---TS------------------------------------

gi|321461882 KR-----GGP-LDRFRYLT---SS------------------------------------

gi|344241635 ------------------------------------------------------------

gi|389613513 -----------------------------------------MSEYQTDI-------IDVS

gi|313219992 -------KRG--L--EWNPYIQNC------------------------------------

gi|313225743 -------KRG--L--EWNPYIQNC------------------------------------

gi|198418193 GRIKR-GRTF--S--PYER---AA------------------------------------

gi|391343942 KRG----PPV--P--NYYT---SA------------------------------------

gi|196014283 QRC----RDL--DI--YVP---TC------------------------------------

gi|307204802 TP-----FKIDFE--MYNF---IF------------------------------------

gi|225718096 KRC----LKD--S--PYFK---AS------------------------------------

gi|324096424 ------------------------------------------------------------

gi|327352193 -------GWF--S--EVSD-M--W------------------------------------

gi|226293104 -------GWF--S--EISD-M--W------------------------------------

gi|154277382 -------GWF--S--EISD-M--W------------------------------------

gi|212532035 -------GWF--S--ETSD-M--W------------------------------------

gi|242117904 -------GWF--T--ETCT-L--W------------------------------------

gi|134077286 -------GWF--S--EQSD-M--W------------------------------------

gi|348570938 -------GWF--R--ETCS-L--W------------------------------------

gi|389628860 -------GWF--R--EISD-M--W------------------------------------

gi|119495240 -------GWF--S--EQSD-M--W------------------------------------

gi|67516811 -------GWF--S--EQSE-M--W------------------------------------

gi|242774578 -------GWF--S--EASD-M--W------------------------------------

gi|225683737 -------GWF--S--EISD-M--W------------------------------------

gi|255716758 -------GWF--R--EISDTM--W------------------------------------

gi|395218408 -------VWF--T--ERDE-----------------------------------------

gi|348514634 -------GWF--T--ESCT-L--W------------------------------------

gi|347828643 -------GWF--R--EISE-M--W------------------------------------

gi|361131873 -------GWF--R--EISE-M--W------------------------------------

gi|392864754 -------GWF--S--EISD-M--W------------------------------------

gi|115492019 -------GWF--S--EQSD-M--W------------------------------------

gi|212532039 -------GWF--S--ETSD-M--W------------------------------------

gi|340914898 -------GWF--R--EISD-M--W------------------------------------

gi|156044358 -------GWF--R--EISE-M--W------------------------------------

gi|342887616 -------GWF--R--EISD-M--W------------------------------------

gi|302307177 -------GWF--R--EISDTM--W------------------------------------

gi|367038823 -------GWF--R--EISD-M--W------------------------------------

gi|358371985 -------GWF--S--EQSD-M--W------------------------------------

gi|317030551 -------GWF--S--EQSD-M--W------------------------------------

gi|303318439 -------GWF--S--EISD-M--W------------------------------------

gi|85100119 -------GWF--R--EISN-M--W------------------------------------

gi|380088740 -------GWF--R--EISN-M--W------------------------------------

gi|395840954 -------GWF--R--ETCS-L--W------------------------------------

gi|16758208 -------GWF--R--ETCS-L--W------------------------------------

gi|327287238 -------GWF--R--ETCS-L--W------------------------------------

gi|146414243 -------GWF--A--EVSDTM--W------------------------------------

gi|190348490 -------GWF--A--EVSDTM--W------------------------------------

gi|367025809 -------GWF--R--EISD-M--W------------------------------------

gi|121701283 -------GWF--S--EQSG-M--W------------------------------------

gi|119592082 -------GWF--R--ETCS-L--W------------------------------------

gi|70995928 -------GWF--S--EQSD-M--W------------------------------------

gi|6678131 -------GWF--R--ETCS-L--W------------------------------------

gi|531202 -------GWF--R--ETCS-L--W------------------------------------

gi|114553926 -------GWF--R--ETCS-L--W------------------------------------

gi|63253298 -------GWF--R--ETCS-L--W------------------------------------

gi|122921244 -------GWF--R--ETCS-L--W------------------------------------

gi|116197943 -------GWF--R--EISE-M--W------------------------------------

gi|378733877 -------GWF--R--EISD-M--W------------------------------------

gi|26390475 -------GWF--R--ETCS-L--W------------------------------------

gi|402852915 -------GWF--R--ETCS-L--W------------------------------------

gi|347447556 -------GWF--R--ETCS-L--W------------------------------------

gi|383873318 -------GWF--R--ETCS-L--W------------------------------------

gi|213409616 -------GWF--R--EINK-M--W------------------------------------

gi|32766339 -------GWF--T--ETCT-L--W------------------------------------

gi|387515006 -------GWF--R--EISDTM--W------------------------------------

gi|46137467 -------GWF--R--EISD-M--W------------------------------------

gi|320583104 -------GWF--A--EVSDTM--W------------------------------------

gi|350585594 -------GWF--H--ETCS-L--W------------------------------------

gi|315053415 -------GWF--S--EVSD-M--W------------------------------------

gi|322706886 -------GWF--R--EISD-M--W------------------------------------

gi|402082530 -------GWF--R--EISD-M--W------------------------------------

gi|317137159 -------GWF--S--EKSN-M--W------------------------------------

gi|147902014 -------GWF--R--ETCS-L--W------------------------------------

gi|344283521 -------GWF--R--ETCS-L--W------------------------------------

gi|76637216 -------GWF--R--ETCS-L--W------------------------------------

gi|255937259 -------GWF--S--EKSG-M--W------------------------------------

gi|366998257 -------GWF--R--EISDTM--W------------------------------------

gi|320588782 -------GWF--R--EISE-M--W------------------------------------

gi|254580735 -------GWF--R--EISDTM--W------------------------------------

gi|301620173 -------GWF--R--ETCS-L--W------------------------------------

gi|258572568 -------GWF--S--EISE-M--W------------------------------------

gi|225710012 KRC----RQD--S--TYYK---SS------------------------------------

gi|19112807 -------GWF--R--EINN-M--W------------------------------------

gi|398411809 -------GWF--R--EQSD-M--W------------------------------------

gi|379728455 -------VWF--T--DKDE-----------------------------------------

gi|149239971 -------GWF--A--EKSATM--W------------------------------------

gi|119175541 -------GWF--S--EISD-M--W------------------------------------

gi|320039089 -------GWF--S--EISD-M--W------------------------------------

gi|74138635 -------GWF--R--ETCS-L--W------------------------------------

gi|238489149 -------GWF--S--EKSN-M--W------------------------------------

gi|255725052 -------GWF--A--EISDTM--W------------------------------------

gi|322701141 -------GWF--R--EISD-M--W------------------------------------

gi|359378073 -------GWF--A--EISDTM--W------------------------------------

gi|358378881 -------GWY--R--EISD-M--W------------------------------------

gi|50286095 -------GWF--R--EISDTM--W------------------------------------

gi|358391288 -------GWF--R--EISN-M--W------------------------------------

gi|401623242 -------GWF--R--EISDTM--W------------------------------------

gi|6325326 -------GWF--R--EISDTM--W------------------------------------

gi|151942852 -------GWF--R--EISDTM--W------------------------------------

gi|302912624 -------GWF--R--EISE-M--W------------------------------------

gi|261190484 --------------------M--W------------------------------------

gi|336262713 -------GWF--R--EISN-M--W------------------------------------

gi|296814308 -------GWF--S--EISD-M--W------------------------------------

gi|126138914 -------GWF--A--EVSDTM--W------------------------------------

gi|50420767 -------GWF--A--EISDTM--W------------------------------------

gi|68482416 -------GWF--A--EISDTM--W------------------------------------

gi|169615146 -------GWF--R--EISN-M--W------------------------------------

gi|396485345 -------GWF--R--EISN-M--W------------------------------------

gi|241951762 -------GWF--A--EISDTM--W------------------------------------

gi|400597126 -------GWF--R--EISN-M--W------------------------------------

gi|345561897 -------GWF--R--EINE-M--W------------------------------------

gi|310793583 -------GWF--R--EISD-M--W------------------------------------

gi|340520373 -------GWF--R--EISD-M--W------------------------------------

gi|47228825 -------GWF--M--EKSA-L--W------------------------------------

gi|156843587 -------GWF--R--EISDTM--W------------------------------------

gi|330907629 -------GWF--R--EISG-M--W------------------------------------

gi|189207669 -------GWF--R--EISG-M--W------------------------------------

gi|50554613 -------GWF--R--EISDTM--W------------------------------------

gi|375086054 -------LWF--S--ELHT-----------------------------------------

gi|366996717 -------GWF--R--EISDTM--W------------------------------------

gi|380353232 -------GWF--A--EISETM--W------------------------------------

gi|154304795 -------GWF--R--EISE-M--W------------------------------------

gi|171693823 -------GWF--R--EISN-M--W------------------------------------

gi|260781328 KRG----MRL--D--PYIT---TT------------------------------------

gi|344234284 -------GWF--A--EISNTM--W------------------------------------

gi|260951089 -------GWF--A--EVSDTM--W------------------------------------

gi|367012209 -------GWF--R--EISDTM--W------------------------------------

gi|403214093 -------GWF--R--EISDTM--W------------------------------------

gi|354548134 -------GWF--A--EISETM--W------------------------------------

gi|372463636 -------GWF--R--EISDTM--W------------------------------------

gi|284929129 -------VWL--T--DRNE-----------------------------------------

gi|291533315 -------LWF--S--ELHT-----------------------------------------

gi|384501417 -------GWF--M--EKS-TM--W------------------------------------

gi|401838282 -------GWF--R--EISDTM--W------------------------------------

gi|344302668 -------GWF--A--EISETM--W------------------------------------

gi|365982711 -------GWF--R--EISDTM--W------------------------------------

gi|296421657 -------GWF--R--EVSE-M--W------------------------------------

gi|149695532 -------GLF--R--ETCS-L--W------------------------------------

gi|50307673 -------GWF--N--EVSDKS--F------------------------------------

gi|50303991 -------GWF--R--EISDTM--W------------------------------------

gi|380483699 --------------------M--W------------------------------------

gi|325660851 -------LWF--S--EVQT-----------------------------------------

gi|160932658 -------LWF--T--ERHT-----------------------------------------

gi|363751094 -------GWF--R--EISETI--C------------------------------------

gi|254569166 -------GWF--M--EISDTM--W------------------------------------

gi|268612001 -------LWF--T--ERHT-----------------------------------------

gi|225018465 -------LWF--T--ERHT-----------------------------------------

gi|326427880 -------GWF--S--ELSPEM--W------------------------------------

gi|297617720 -------VWY--S--ELHQ-----------------------------------------

gi|295665965 -------GWF--S--EISD-M--W------------------------------------

gi|317131721 -------LWF--T--ELHT-----------------------------------------

gi|346467789 -------GWF--T--EIVN-T--N------------------------------------

gi|328766899 -------DWF--K--ETTA-L--W------------------------------------

gi|323486581 -------FWF--S--EKHT-----------------------------------------

gi|326476682 -------GWF--S--EVSD-M--W------------------------------------

gi|164660446 -------GWF--H--EENP-Q--W------------------------------------

gi|169351543 -------LWF--S--ERHT-----------------------------------------

gi|346326304 -------GWF--R--EISH-M--W------------------------------------

gi|167519370 -------GWF--S--EVSSEM--W------------------------------------

gi|321468222 -------GWF--S--ELSP-L--W------------------------------------

gi|404329717 -------LWF--T--EKHT-----------------------------------------

gi|283798449 -------YWF--S--EEQT-----------------------------------------

gi|300121366 -------GWF--Y--ESET-L--W------------------------------------

gi|227112962 -------IWY--E--TLHA-----------------------------------------

gi|388582960 -------GWF--H--EISP-Q--W------------------------------------

gi|225713606 NRC----QKE--S--PYLR---SS------------------------------------

gi|346466755 -------GWF--T--EIVN-T--N------------------------------------

gi|355720872 ------------------------------------------------------------

gi|167755034 -------LWF--T--ERHT-----------------------------------------

gi|193215178 -------IWF--T--EKGH-----------------------------------------

gi|403059603 -------IWY--E--TLHA-----------------------------------------

gi|388854692 -------GWF--H--ETNS-Q--W------------------------------------

gi|227329706 -------IWY--E--TLHA-----------------------------------------

gi|253689498 -------IWY--E--TLHA-----------------------------------------

gi|300715359 -------MWY--E--TLHT-----------------------------------------

gi|343427335 -------GWF--H--ETNS-Q--W------------------------------------

gi|1167999 -------GWF--R--ETCS-L---------------------------------------

gi|261369006 -------MWF--S--EFHT-----------------------------------------

gi|327405610 -------VWF--T--DKDD-----------------------------------------

gi|254573952 -------GWF--R--EISDEY--F------------------------------------

gi|251788647 -------IWY--E--TLHA-----------------------------------------

gi|327309372 -------GWF--S--EVSD-M--W------------------------------------

gi|313896579 -------FWY--T--EQHT-----------------------------------------

gi|342871552 -------GWF--Q--EISD-M--W------------------------------------

gi|295101693 -------FWF--S--EFHT-----------------------------------------

gi|302665792 -------GWF--S--EVSD-M--W------------------------------------

gi|71023471 -------GWF--H--ETNS-Q--W------------------------------------

gi|50122255 -------IWY--E--TLHA-----------------------------------------

gi|320583760 -------GWF--R--EESDEF--F------------------------------------

gi|328351802 --------------------M--W------------------------------------

gi|331082383 -------LWF--S--EKQT-----------------------------------------

gi|320529049 -------FWY--T--EQHT-----------------------------------------

gi|255658393 -------LWF--T--EHHT-----------------------------------------

gi|302500029 -------GWF--S--EVSD-M--W------------------------------------

gi|307132173 -------IWY--E--TLHA-----------------------------------------

gi|357057706 -------FWY--T--EQHT-----------------------------------------

gi|271501669 -------IWY--E--TLHA-----------------------------------------

gi|348689589 -------QWF--S--ETEA-M--W------------------------------------

gi|387510768 -------GWF--R--EISDAH--F------------------------------------

gi|260588048 -------LWF--S--EKQT-----------------------------------------

gi|160943874 -------FWF--S--EFHT-----------------------------------------

gi|188532978 -------MWY--E--TLHT-----------------------------------------

gi|145362282 -------GWF--S--EMSP-M--W------------------------------------

gi|394757460 -------LWF--T--EKHA-----------------------------------------

gi|397614365 -------GWF--H--ERGP-L--W------------------------------------

gi|170025697 -------LWY--E--TLHA-----------------------------------------

gi|401624651 -------GWF--R--EINDKS--F------------------------------------

gi|51595070 -------LWY--E--TLHA-----------------------------------------

gi|257439175 -------FWF--S--EFHT-----------------------------------------

gi|238926770 -------FWY--T--EEHT-----------------------------------------

gi|392423567 -------LWY--T--EQHT-----------------------------------------

gi|151941307 -------GWF--R--EINDKS--F------------------------------------

gi|270263031 -------IWY--E--TLHA-----------------------------------------

gi|153813543 -------MWF--S--EFHT-----------------------------------------

gi|21957501 -------LWY--E--TLHA-----------------------------------------

gi|325184913 -------EWF--T--ETEA-L--W------------------------------------

gi|292670864 -------FWY--T--EQHT-----------------------------------------

gi|108808904 -------LWY--E--TLHA-----------------------------------------

gi|154623414 -------GWF--S--EMSP-M--W------------------------------------

gi|259148132 -------GWF--R--EINDKS--F------------------------------------

gi|402833951 -------LWF--T--EKHT-----------------------------------------

gi|242240254 -------IWY--E--TLHA-----------------------------------------

gi|196007934 -------GWF--R--EFSS-L--W------------------------------------

gi|391628126 -------LWY--E--TLHA-----------------------------------------

gi|323308143 -------GWF--R--EINDKS--F------------------------------------

gi|377578674 -------VWQ--E--TLHD-----------------------------------------

gi|402570735 -------LWY--T--EQHT-----------------------------------------

gi|355378207 -------FWY--T--EQHT-----------------------------------------

gi|401565378 -------FWY--T--EQHT-----------------------------------------

gi|6323175 -------GWF--R--EINDKS--F------------------------------------

gi|354559941 -------LWY--T--EQHT-----------------------------------------

gi|359463278 -------VWF--T--DRND-----------------------------------------

gi|295108885 -------MWF--S--EFHT-----------------------------------------

gi|158319435 -------LWY--T--EQHT-----------------------------------------

gi|334128073 -------FWY--T--EQHT-----------------------------------------

gi|312162112 -------GWF--A--EISP-M--W------------------------------------

gi|313114511 -------FWF--S--EFHT-----------------------------------------

gi|349579866 -------GWF--R--EINDKS--F------------------------------------

gi|116785514 -------GWF--A--EISP-M--W------------------------------------

gi|333929035 -------IWY--E--TLHA-----------------------------------------

gi|260887129 -------LWF--T--EKHT-----------------------------------------

gi|386825267 -------IWY--E--TLHA-----------------------------------------

gi|37524842 -------IWS--E--TLHA-----------------------------------------

gi|198427208 -------NWF--S--ELNS-L--W------------------------------------

gi|291545477 -------MWF--S--EFHT-----------------------------------------

gi|182418090 -------LWY--T--EQHT-----------------------------------------

gi|259907494 -------MWY--E--TLHA-----------------------------------------

gi|291549279 -------MWF--S--EFHT-----------------------------------------

gi|157372235 -------IWY--E--TLHA-----------------------------------------

gi|297845432 -------GWF--S--EMSP-M--W------------------------------------

gi|12230014 -------GWF--S--EMSP-M--W------------------------------------

gi|170032648 -------EWF--S--EVSDVL--W------------------------------------

gi|198453209 -------GWF--S--EIQAEL--W------------------------------------

gi|284049217 -------MWF--S--EFHT-----------------------------------------

gi|238754470 -------LWY--E--TLHA-----------------------------------------

gi|257126567 -------LWY--T--EEHT-----------------------------------------

gi|385785731 -------MWY--E--TLHA-----------------------------------------

gi|238759889 -------LWY--E--TLHA-----------------------------------------

gi|238750530 -------LWY--E--TLHA-----------------------------------------

gi|6094336 -------GWF--S--EISP-L--W------------------------------------

gi|332160447 -------LWY--E--TLHA-----------------------------------------

gi|377851579 -------QWH--E--TLHD-----------------------------------------

gi|388391237 -------QWH--E--TLHD-----------------------------------------

gi|91209189 -------QWH--E--TLHD-----------------------------------------

gi|169837854 -------LWY--T--EEHT-----------------------------------------

gi|317046947 -------MWY--E--TLHA-----------------------------------------

gi|256273016 -------GWF--R--EINDKS--F------------------------------------

gi|218547577 -------QWH--E--TLHD-----------------------------------------

gi|374579091 -------LWL--T--EDER-----------------------------------------

gi|373245798 -------QWH--E--TLHD-----------------------------------------

gi|387605598 -------QWH--E--TLHD-----------------------------------------

gi|187935464 -------LWY--T--EKHT-----------------------------------------

gi|384470785 -------QWH--E--TLHD-----------------------------------------

gi|329298049 -------TWH--E--TLHS-----------------------------------------

gi|110803121 -------LWY--T--EEHT-----------------------------------------

gi|212639268 -------LWL--T--EDER-----------------------------------------

gi|20807781 -------LWF--T--EHQD-----------------------------------------

gi|253988276 -------IWH--E--TLHA-----------------------------------------

gi|315294649 -------QWH--E--TLHD-----------------------------------------

gi|383191575 -------IWY--E--TLHA-----------------------------------------

gi|386228114 -------QWH--E--TLHD-----------------------------------------

gi|330009512 -------LWH--E--TLHD-----------------------------------------

gi|296086541 -------GWF--S--EISP-M--W------------------------------------

gi|392297662 -------GWF--R--EINDKS--F------------------------------------

gi|292489273 -------MWY--E--TLHA-----------------------------------------

gi|225424695 -------GWF--S--EISP-M--W------------------------------------

gi|24111564 -------QWH--E--TLHD-----------------------------------------

gi|194433428 -------QWH--E--TLHD-----------------------------------------

gi|333978511 -------CWF--T--ELQT-----------------------------------------

gi|378194398 -------QWH--E--TLHD-----------------------------------------

gi|238791204 -------LWY--E--TLHA-----------------------------------------

gi|297841771 -------GWF--S--EISP-M--W------------------------------------

gi|15799805 -------QWH--E--TLHD-----------------------------------------

gi|110799842 -------LWY--T--EEHT-----------------------------------------

gi|324112468 -------LWH--E--TLHD-----------------------------------------

gi|260890661 -------LWY--T--EEHT-----------------------------------------

gi|401840422 -------GWF--R--EINDKS--F------------------------------------

gi|312970216 -------QWH--E--TLHD-----------------------------------------

gi|16128114 -------QWH--E--TLHD-----------------------------------------

gi|386248282 -------QWH--E--TLHD-----------------------------------------

gi|366159436 -------LWH--E--TLHD-----------------------------------------

gi|323975716 -------QWH--E--TLHD-----------------------------------------

gi|384503184 -------GWF--S--EISP-M--W------------------------------------

gi|18309532 -------LWY--T--EEHT-----------------------------------------

gi|372276039 -------TWY--E--TLHA-----------------------------------------

gi|333009123 -------QWH--E--TLHD-----------------------------------------

gi|332097889 -------QWH--E--TLHD-----------------------------------------

gi|381405644 -------TWH--E--TLHA-----------------------------------------

gi|303325156 -------QWH--E--TLHD-----------------------------------------

gi|377919674 -------QWH--E--TLHD-----------------------------------------

gi|300905534 -------QWH--E--TLHD-----------------------------------------

gi|15223115 -------GWF--S--EISP-M--W------------------------------------

gi|145336078 -------GWF--S--EMSP-M--W------------------------------------

gi|206578746 -------LWH--E--TLHD-----------------------------------------

gi|42524959 -------VWF--T--DKDE-----------------------------------------

gi|323157811 -------QWH--E--TLHD-----------------------------------------

gi|391275496 -------QWH--E--TLHD-----------------------------------------

gi|195446427 -------GWF--S--EVQNEL--W------------------------------------

gi|251778321 -------LWY--T--EKHT-----------------------------------------

gi|152968708 -------LWH--E--TLHD-----------------------------------------

gi|371605278 -------QWH--E--TLHD-----------------------------------------

gi|345395892 -------QWH--E--TLHD-----------------------------------------

gi|385302583 ------------------------------------------------------------

gi|386206487 -------HWH--E--TLHD-----------------------------------------

gi|238761600 -------LWY--E--TLHA-----------------------------------------

gi|194746211 -------GWF--S--ELQADL--W------------------------------------

gi|331226164 -------GWF--R--EESN-L--W------------------------------------

gi|340742010 -------QWH--E--TLHD-----------------------------------------

gi|50552163 -------SWF--T--EVSD-S--F------------------------------------

gi|238786761 -------LWY--E--TLHA-----------------------------------------

gi|386033187 -------LWH--E--TLHD-----------------------------------------

gi|6094327 -------GWF--S--EISP-L--W------------------------------------

gi|82775410 -------QWH--E--TLHD-----------------------------------------

gi|110762382 -------GWF--S--EIND-L--W------------------------------------

gi|333011508 -------QWH--E--TLHD-----------------------------------------

gi|322834377 -------IWY--E--TLHA-----------------------------------------

gi|374992883 -------LWY--T--EEQT-----------------------------------------

gi|308185706 -------TWY--E--TLHA-----------------------------------------

gi|157147448 -------KWH--E--TLHD-----------------------------------------

gi|12229958 -------GWF--S--EISP-M--W------------------------------------

gi|33340515 -------GWF--S--EISP-L--W------------------------------------

gi|295098625 -------VWQ--E--TLHD-----------------------------------------

gi|380021423 -------GWF--S--EIND-L--W------------------------------------

gi|238797200 -------LWY--E--TLHA-----------------------------------------

gi|293394516 -------IWH--E--TLHA-----------------------------------------

gi|99083515 -------GWF--S--EISP-M--W------------------------------------

gi|388608380 -------VWQ--E--TLHD-----------------------------------------

gi|320173653 -------QWH--E--TLHD-----------------------------------------

gi|161612515 -------MWH--E--TLHD-----------------------------------------

gi|378073834 -------QWH--E--TLHD-----------------------------------------

gi|161504706 -------MWH--E--TLHD-----------------------------------------

gi|195499508 -------GWF--S--ELQADL--W------------------------------------

gi|2821961 --------WF--S--EISP-M--W------------------------------------

gi|359376594 -------YWF--Q--EKSD-S--F------------------------------------

gi|374297740 -------LWF--T--EYHT-----------------------------------------

gi|239827059 -------LWL--T--EDDR-----------------------------------------

gi|403383804 -------LWY--S--EHHT-----------------------------------------

gi|304396607 -------TWY--E--TLHA-----------------------------------------

gi|397166809 -------MWQ--E--TLHD-----------------------------------------

gi|123441066 -------LWY--E--TLHA-----------------------------------------

gi|82542724 -------QWH--E--TLHD-----------------------------------------

gi|12229957 -------GWF--S--EISP-M--W------------------------------------

gi|376394468 -------LWH--E--TLHD-----------------------------------------

gi|218687999 -------QWH--E--TLHD-----------------------------------------

gi|170768493 -------RWH--E--TLHD-----------------------------------------

gi|365156921 -------LWL--T--ENDR-----------------------------------------

gi|195330342 -------GWF--S--ELQADL--W------------------------------------

gi|340356265 -------LWY--T--EDHS-----------------------------------------

gi|388336724 -------QWH--E--TLHD-----------------------------------------

gi|188589068 -------LWY--T--EKHT-----------------------------------------

gi|376400744 -------LWH--E--TLHD-----------------------------------------

gi|28317069 -------GWF--S--ELQADL--W------------------------------------

gi|389842198 -------MWH--E--TLHD-----------------------------------------

gi|260596552 -------MWH--E--TLHD-----------------------------------------

gi|6094335 -------GWF--S--EISP-M--W------------------------------------

gi|365988413 -------GWF--R--EINDKC--F------------------------------------

gi|194446066 -------MWH--E--TLHD-----------------------------------------

gi|350536257 -------GWF--S--EISP-L--W------------------------------------

gi|357485917 -------GWF--S--EISP-M--W------------------------------------

gi|296127808 -------LWY--T--ENHT-----------------------------------------

gi|74310739 -------QWH--E--TLHD-----------------------------------------

gi|323166003 -------QWH--E--TLHD-----------------------------------------

gi|126153731 -------GWF--S--EISP-M--W------------------------------------

gi|291280945 -------QWH--E--TLHD-----------------------------------------

gi|49425361 -------GWF--S--EISP-M--W------------------------------------

gi|334341918 -------LWF--T--EKDT----V------------------------------------

gi|187731639 -------QWH--E--TLHD-----------------------------------------

gi|156935354 -------MWH--E--TLHD-----------------------------------------

gi|376377905 -------LWH--E--TLHD-----------------------------------------

gi|392391837 -------LWY--T--EEHT-----------------------------------------

gi|331645238 -------QWH--E--TLHD-----------------------------------------

gi|378578181 -------MWY--E--TLHT-----------------------------------------

gi|24645443 -------GWF--S--ELQADL--W------------------------------------

gi|335578432 -------QWH--E--TLHD-----------------------------------------

gi|374383060 -------LWY--T--EEHT-----------------------------------------

gi|358248320 -------GWF--S--EISP-M--W------------------------------------

gi|300919677 -------QWH--E--TLHD-----------------------------------------

gi|194902914 -------GWF--S--ELQADL--W------------------------------------

gi|226492509 -------GWF--S--EISP-M--W------------------------------------

gi|391290786 -------QWH--E--TLHD-----------------------------------------

**S167D**

lcl|84191 -----------------D--GRLVEYD-IDEVVYDEDSPYQNIKILHSKQFGNILILSGD

gi|168177377 -----------------D--GRLVEYD-IDEVVYDEDSPYQNIKILHSKQFGNILILSGD

gi|21264341 -----------------D--GRLVEYD-IDEVVYDEDSPYQNIKILHSKQFGNILILSGD

gi|791051 -----------------D--GRLVEYD-IDEVVYDEDSPYQNIKILHSKQFGNILILSGD

gi|332860397 -----------------D--GRLVEYD-IDEVVYDEDSPYQNIKILHSKQFGNILILSGD

gi|119619392 -----------------D--GRLVEYD-IDEVVYDEDSPYQNIKILHSKQFGNILILSGD

gi|402909680 -----------------D--GRLVEYD-IDEVVYDEDSPYQNIKILHSKQFGNILILSGD

gi|332864823 -----------------D--GRLVEYD-IDEVVYDEDSPYQNIKILHSKQFGNILILSGD

gi|354474372 -----------------D--GRLVEYD-IDEVVYDEDSPYQNIKILHSKQFGNILILSGD

gi|291407209 -----------------D--GRLVEYD-IDEVVYDEDSPYQNIKILHSKQFGNILILSGD

gi|297303473 -----------------D--GRLVEYD-IDEVVYDEDSPYQNIKILHSKQFGNILILSGD

gi|403263885 -----------------D--GRLVEYD-IDEVVYDEDSPYQNIKILHSKQFGNILILSGD

gi|395753759 -----------------D--GRLVEYD-IDEVVYDEDSPYQNIKILHSKQFGNILILSGD

gi|355704663 -----------------D--GRLVEYD-IDEVVYDEDSPYQNIKILHSKQFGNILILSGD

gi|4164136 -----------------D--GRLVEYD-IDEVVYDEDSPYQNIKILHSKQFGNILILSGD

gi|149030940 -----------------D--GRLVEYD-IDEVVYDEDSPYQNIKILHSKQFGNILILSGD

gi|76559933 -----------------D--GRLVEYD-IDEVVYDEDSPYQNIKILHSKQFGNILILSGD

gi|78369282 -----------------D--GRLVEYD-IDKVVYDEDSPYQNIKILHSKQFGNILILSGD

gi|118600998 -----------------D--GRLVEYD-IDEVVYDEDSPYQNIKILHSKQFGNILILSGD

gi|345806962 -----------------D--GRLVEYD-IDEVVYDEDSPYQNIKILHSKQFGNILILSGD

gi|154425706 -----------------D--GRLVEYD-IDKVVYDEDSPYQNIKILHSKQFGNILILSGD

gi|26340516 -----------------D--GRLVEYD-IXEVVYDEDSPYQNIKILHSKQFGNILILSGD

gi|384948950 -----------------D--GRLVEYD-IDEVVYDEDSPYQNIKILHSKQFGNILILSGD

gi|397497639 -----------------D--GRLVEYD-IDEVVYDEDSPYQNIKILHSKQFGNILILSGD

gi|348561339 -----------------D--GRLVEYD-IDEVVYDEDSPYQNIKILHSKQFGNILILSGD

gi|354473432 -----------------D--GRLVEYD-IDEVVYDEDSPYQNIKILHSKQFGNILILSGD

gi|344288661 -----------------D--GRLVEYD-IDEVVYDEDSPYQNIKILHSKQFGNILILSGD

gi|338728955 -----------------D--GRLVEYD-IDEVVYDEDSPYQNIKILHSKQFGNILILSGD

gi|71895235 -----------------D--GRLVEYD-IDEVVYDEDSPFQNIKILHSKQFGNILILSGD

gi|297678474 -----------------D--GRLVEYD-MDEVVYDEDSPYQNIKILHSKQFGNILILSGD

gi|301756254 -----------------D--GRLVEYD-IDEVVYDEDSPYQNIKILHSKQFGNILILSGD

gi|296470508 -----------------D--GRLVEYD-IDKVVYDEDSPYQNIKILHSKQFGNILILSGD

gi|351712477 -----------------D--GRLVEYD-IDEVVYDEDSPYQNIKILHSKQFGNILILSGD

gi|344242683 -----------------D--GRLVEYD-IDEVVYDEDSPYQNIKILHSKQFGNILILSGD

gi|387018788 -----------------D--GRLVEYD-IDEVIYDEESAYQNIKILHSKQFGNILILSGD

gi|327268286 -----------------D--GRLVEYD-IDEVVYDEESPYQNIKILHSKQFGNILILSGD

gi|334329503 -----------------D--GRLVEYD-IDEVVYDEDSPYQNIKILHSKQFGNILILSGD

gi|350595577 -----------------D--GRLVEYD-IDEVVYDEDSPYQNIKILHSKQFGNILILSGD

gi|351706018 -----------------D--GCLVGYD-IDEVVSDGDSPYQNIKILHSKQFGNILILSGD

gi|395518817 -----------------D--GRLVEYD-IDEVVYDEDSPYQNIKILHSKQFGNILILSGD

gi|149042406 -----------------D--GRLVEYD-IDEVVYDEDSPYQNIKILHSKQFGNILILSGD

gi|326913546 -----------------D--GRLVEYD-IDEVVYDEDSPFQNIKILHSKQFGNILILSGD

gi|224042691 -----------------D--GRLVEYD-IDEVVYDEDSPFQNIKILHSKQFGNILILSGD

gi|148708890 -----------------D--GRLVEYD-IDEVVYDEDSPYQNIKILHSKQFGNILILSGD

gi|345327044 -----------------D--GRLVEYD-IDRVVFDQDSPFQNIKILHSKQFGNILVLNGD

gi|385862226 -----------------D--GRLVEYD-IDEIVYSKESPYQNIKIMHSKQFGNILILNGD

gi|354482601 -----------------D--GRLVEYD-IDEVVYDEDSPYQNIKILHSKQFGNILILSGD

gi|281352943 -----------------D--GRLVEYD-IDEVVYDEDSPYQNIKILHSKQFGNILILSGD

gi|18858203 -----------------D--GRLMEYD-IDEVVYEKDSAYQNIKILHSRQFGNMLILNGD

gi|395838143 -----------------D--GRLVEYD-IDEVVYDEDSPYQNIKILHSKQFGNILILSGD

gi|213511272 -----------------D--GRLVEYD-IDRVVYDEDSAYQNIKIMHSRQFGNILILNGD

gi|348518728 -----------------D--GRLIEYD-IDKVVYEEDSEYQNIKILHSQQYGNILVLDGD

gi|386643030 -----------------D--GRLVEYD-IDEVVYDEDSPYQNIKILHSKQFGNILILSGD

gi|402909682 -----------------D--GRLVEYD-IDEVVYDEDSPYQNIKILHSKQFGNILILSGD

gi|4007861 -----------------D--GRLMEYD-IDKVVYDEDSVYQKIQILHSKQYGNILVLNGN

gi|351706975 -----------------D--GRLVEYD-IDEVVYDEDSPYQNIKILHSKQFGMGMLI---

gi|4007046 -----------------D--DRLIEYD-IDQVLYEEDSAYQNIQILHSKQYGNILVLNGD

gi|114687978 -----------------D--GRLVEYD-IDEVVYDEDSPYQNIKILHSKQFGNILILSGD

gi|2916761 -----------------D--GRLVEYD-IDEVVYDEDSPYQNIKILHSKQFGNILILSGD

gi|194373711 -----------------D--GRLVEYD-IDEVVYDEDSPYQNIKILHSKQFGNILILSGD

gi|332860399 -----------------D--GRLVEYD-IDEVVYDEDSPYQNIKILHSKQFGNILILSGD

gi|125978661 -----------------D--ERIIEYD-IDKLVYEVRSPFQKIQILHSKTLGNMLLLDEL

gi|225581217 -----------------D--ERIIEYD-IDKLVYEVRSPFQKIQILHSKTLGNMLLLDEL

gi|195376249 -----------------D--ERIIEYD-IDTLVYEARSPFQKIQIMHSKTLGNMLILDEL

gi|195126140 -----------------D--ERIIEYD-IDTLVYEARSPFQKIQILHSKTLGNMLILDEL

gi|194747133 -----------------D--ERIIEYD-IDKVVYEARSPFQKIQIMHSKTLGNMLLLDEL

gi|195012869 -----------------D--ERIIEYD-IDTLVYETRSPFQKIQIMHSKTLGNMLILDEL

gi|24663238 -----------------D--ERIIEYD-IDKVVFEARSPFQKIQIMHSKTLGNMLLLDEL

gi|194869781 -----------------D--ERIIEYD-IDKVVFEERSPFQKIQIMHSKTLGNMLLLDEL

gi|195327091 -----------------D--ERIIEYD-IDKVVFEARSPFQKIQIMHSKSLGNMLLLDEL

gi|390176333 -----------------D--ERIIEYD-IDKLVYEVRSPFQKIQILHSKTLGNMLLLDEL

gi|21358269 -----------------D--ERIIEYD-IDKVVFEARSPFQKIQIMHSKTLGNMLLLDEL

gi|74211624 -----------------D--GRLVEYD-IDEVVYDEDSPYQNIKILHSKQFGNILILSGD

gi|195589784 KLPTLKRGDVSRYFPSSD--ERIIEYD-IDKVVFEARSPFQKIQIMHSKTLGNMLLLDEL

gi|289742457 -----------------D--ERIIEYD-IDSLLYEARSPFQKIQIMHSKTLGNMLILDEL

gi|157118336 -----------------D--ERVIEYD-IDKVLYDKRSDFQKIQIVHSKSLGNMLVLDEL

gi|158293995 -----------------D--ERVIEYD-IDRVLFDKRSEFQKIQIVHSRSLGNMLVLDEL

gi|170032109 -----------------D--ERVIEYD-IDKVLYDKRSDFQKIQIVHSKSLGNMLVLDEL

gi|327290877 -----------------D--GRLVEYD-IDEVVYDEESSYQNIKILHSKQFGNILILSGD

gi|4927220 -----------------D--GRLVEYD-IDEVVYDEDSPYQNIKILHSKQFGNILILSGD

gi|195493801 KLPTLKRGDVSRYFPSSD--ERIIEYD-IDKVVFEARSPFQKIQIMHSKTLGNMLLLDEL

gi|242024750 -----------------D--ERILEYD-IDKVVFEETSPYQKIQIVHSKSLGNILLLDDL

gi|312372613 -----------------D--ERVIEYD-IDRVLFDKRSEFQKIQIVHSK-----------

gi|119619391 -----------------D--GRLVEYD-IDEVVYDEDSPYQNIKILHSKQFGNILILSGD

gi|260818384 -----------------D--NKLIEYD-FDEVVYEADSPYQNIKIMHSPQFGNMLLLDND

gi|340709405 -----------------D--DRLLEYD-IDKLVFEARSPYQKVQIVHSKSLGNLLVLDEL

gi|350407163 -----------------D--DRLLEYD-IDKLVFEARSPYQKVQIVHSKSLGNLLVLDEL

gi|383856563 -----------------D--DRLLEYD-IDKLVFEARSPYQKVQIVHSKSLGNLLVLDEL

gi|357613964 PYPSIKRCLFSKYYTTSD--ERLLEYD-IDGVVFDERSPFQRVQVVHSRTFGNMLVLDDL

gi|156542873 -----------------D--ERLLEYD-IDKLVFEARSPYQKVQIVHSKSLGNMLILDEL

gi|332025522 -----------------D--ERLLEYD-IDKLVFEARSPYQKVQIVHSKSLGNLLVLDEL

gi|110750077 -----------------D--DRLLEYD-IDKLVFEARSPYQKVQIVHSKSLGNLLVLDEL

gi|91085293 -----------------D--DRLLEYD-IDELVFEERTPYQKIQIVHSKSLGNMLVLDDL

gi|307188353 -----------------D--ERLLEYD-IDKLVFEARSPYQKVQIVHSKSLGNLLVLDEL

gi|390338494 -----------------D--RRLVEYD-FDAATFEADSEYQNVKIMHSPQYGNMLILDDD

gi|195440770 -----------------D--ERIIEYD-IDTLVYETRSPFQKIQIMHSKTLGNMLILDEL

gi|380023489 -----------------D--DRLLEYD-IDKLVFEARSAYQKVQIVHSKSLGNLLVLDEL

gi|156394332 -----------------D--NLIMQYD-IDREIVNVDSKYQNIKILHSNQFGNMLVLNND

gi|167538084 -----------------D--GLWIQYD-FDELVFHEKSPYQDVKIYHSRQFGNMLLLDDD

gi|326435685 -----------------D--GLLIQYD-FDKTVFHEQSPYQDVKIMHSRQFGNILLLDDD

gi|47227186 -----------------D--GRLMEYD-IDKVVYDEDSAYQKIQILHSKQYGNILVLNGD

gi|321461882 -----------------D--ERLLEYD-VDRVVADVHSPFQHIQIFHTINFGNLLVLDDL

gi|344241635 ------------------------------------------------------------

gi|389613513 DIALLKNP--SRTIPTTY--ERLLEYD-IDGVLFDEQSPFQRVQIVHSKSLGNMLVLDDL

gi|313219992 -----------------D--GTVVEND-FQELLFHQKSPFQDVKIFRSNNFGSYLLLDDD

gi|313225743 -----------------D--GTVVEND-FQELLFHQKSPFQDVKIFRSNNFGSYLLLDDD

gi|198418193 -----------------T--GLVIEHD-FDKLVFDGKSPYQTVRILHSMQFGNCLVLDND

gi|391343942 -----------------D--ERLLEYD-FDKILFEKQSDFQEVKILSSPTLGAVLFLDDL

gi|196014283 -----------------D--NRLVEYD-FDKVVVNVDTQFQNVKICHSPQYGNMLLLDDD

gi|307204802 -----------------EAYERLLEYD-IDKLVFEARSPYQKVQIVHSKSLGNLLVLDEL

gi|225718096 -----------------D--HRILEYP-IKEVLFSQRSPFQHVQIVDTNDFGKLLILDDM

gi|324096424 ---------------------------------------------MHSKTLGNMLLLDEL

gi|327352193 -----------------P--GQALTLK-VNQILHHEKSKYQDVLVFESSDHGTVLVLDNV

gi|226293104 -----------------P--GQAMTLK-VNQILHHEKSKYQDVLVFESSDHGTVLVLDNV

gi|154277382 -----------------P--GQAMTLK-VNQILHHEKSKYQDVLVFESSDHGTVLVLDNV

gi|212532035 -----------------P--GQAMTLK-VKEVLHHEKSQYQDVLVFESTDYGTVLVLDNV

gi|242117904 -----------------P--GQAMSLQ-VEEVLYHKKSKFQDVMVFKSKTYGNVLILDGV

gi|134077286 -----------------P--GQAMNLK-VNQILHHEKSKYQDVLVFESSDYGTVLVLDNV

gi|348570938 -----------------P--GQALSLQ-VEQLLHHQRSRYQDILVFRSKTYGNVLVLDGV

gi|389628860 -----------------P--GQAMTLK-VEKVLHHERSKYQDVLIFQSTDYGKVLVLDNV

gi|119495240 -----------------P--GQAMNLK-VNQILHHEKSKYQDVLVFESTDYGTVLVLDNV

gi|67516811 -----------------P--GQAMNLR-VNQILHHEKSKYQDVLVFESSDYGTVLVLDNV

gi|242774578 -----------------P--GQAMTLK-VKEVLHHEKSQYQDVLVFESTDHGTVLVLDNV

gi|225683737 -----------------P--GQAMTLK-VNQILHHEKSKYQDVLVFESSDHGTVLVLDNV

gi|255716758 -----------------P--GQAMTLR-VEKVLHHEKSKYQDVLIFKSTDYGNVLVLDNA

gi|395218408 --------------------NIALSLKHTGNQLYKKDSEYQRVEIYETLAYGNMLTLDGM

gi|348514634 -----------------P--GQAMSLQ-VEEVLYHKKSKFQDVMVFKSKTYGNVLVLDGV

gi|347828643 -----------------P--GQAMTLK-VKNVLHHEKSKYQDVLIFESTDYGTVLVLDNV

gi|361131873 -----------------P--GQAMTLK-VKNIIHHEKSKYQDVLIFESTDYGTVLVLDNV

gi|392864754 -----------------P--GQAMTLR-VNQILHHEKSKYQDILIFESTDHGTCLVLDNV

gi|115492019 -----------------P--GQAMNLK-VNQILHHEKSKYQDVLVFESSDYGTVLVLDNV

gi|212532039 -----------------P--GQAMTLK-VKEVLHHEKSQYQDVLVFESTDYGTVLVLDNV

gi|340914898 -----------------A--GQAMTLR-VKKVLHHEKSKYQDVLIFESTDHGRVLVLDNV

gi|156044358 -----------------P--GQAMTLK-VKNVLHHEKSKFQDVLIFESTDYGTVLVLDNV

gi|342887616 -----------------P--GHAMTLR-VEKVLVHEKSKYQDVLIFKSTDFGNVLVLDNV

gi|302307177 -----------------P--GQAMTLR-VEKVLHHEKSKYQDVLVFKSTNHGNVLVLDNV

gi|367038823 -----------------P--GQAMTLQ-VKKVLHHEKSKYQDVLIFESTNHGNVLVLDNV

gi|358371985 -----------------P--GQAMNLK-VNQILHHEKSKYQDVLVFESSDYGTVLVLDNV

gi|317030551 -----------------P--GQAMNLK-VNQILHHEKSKYQDVLVFESSDYGTVLVLDNV

gi|303318439 -----------------P--GQAMTLR-VNQILHHEKSKYQDILIFESTDHGTCLVLDNV

gi|85100119 -----------------P--GQAMTLK-VEKVLHHEKSLYQDVLIFKSTDHGNVLVLDNV

gi|380088740 -----------------P--GQAMTLK-VEKVLHHEKSLYQDVLIFKSTDHGNVLVLDNV

gi|395840954 -----------------P--GQALSLQ-VEQLLHHQRSRYQDILVFRSKTYGNVLVLDGV

gi|16758208 -----------------P--GQALSLQ-VEQLLHHRRSRYQDILVFRSKTYGNVLVLDGV

gi|327287238 -----------------P--GQALSLQ-VEQVLHQHRSPFQEILVFRSKTYGNVLVLDGV

gi|146414243 -----------------P--GQAMSLK-VEKVLHVEKSKYQDVLVFKSTNYGNVLVLDNC

gi|190348490 -----------------P--GQAMSLK-VEKVLHVEKSKYQDVLVFKSTNYGNVLVLDNC

gi|367025809 -----------------P--GQAMTLR-VKKVLHHEKSKYQDVLIFESTDHGNVLVLDNV

gi|121701283 -----------------P--GQAMNLK-VNQILHHEKSKYQDVLVFESTDYGTVLVLDNV

gi|119592082 -----------------P--GQALSLQ-VEQLLHHRRSRYQDILVFRSKTYGNVLVLDGV

gi|70995928 -----------------P--GQAMNLK-VNQILHHEKSKYQDVLVFESTDYGTVLVLDNV

gi|6678131 -----------------P--GQALSLQ-VEQLLHHRRSRYQDILVFRSKTYGNVLVLDGV

gi|531202 -----------------P--GQALSLQ-VEQLLHHRRSRYQDILVFRSKTYGNVLVLDGV

gi|114553926 -----------------P--GQALSLQ-VEQLLHHRRSRYQDILVFRSKTYGNVLVLDGV

gi|63253298 -----------------P--GQALSLQ-VEQLLHHRRSRYQDILVFRSKTYGNVLVLDGV

gi|122921244 -----------------P--GQALSLQ-VEQLLHHRRSRYQDILVFRSKTYGNVLVLDGV

gi|116197943 -----------------P--GQAMTLR-VKKVLHHEKSKFQDVLIFESTNHGNVLVLDNV

gi|378733877 -----------------P--GQAMILK-VNQVLHHEKSKYQDVLVFESSDHGTVLVLDNV

gi|26390475 -----------------P--GQALSLQ-VEQLLHHRRSRYQDILVFRSKTYGNVLVLDGV

gi|402852915 -----------------P--GQALSLQ-VEQLLHHRRSRYQDILVFRSKTYGNVLVLDGV

gi|347447556 -----------------P--GQALSLQ-VEQLLHHRRSRYQDILVFRSKTYGNVLVLDGV

gi|383873318 -----------------P--GQALSLQ-VEQLLHHRRSRYQDILVFRSKTYGNVLVLDGV

gi|213409616 -----------------P--GQAMTLK-VKKVLHAKRSKFQDVLIFESETYGRVLVLDGV

gi|32766339 -----------------P--GQAMSLQ-VEEVLYHKKSKFQDVMVFKSKTYGNVLILDGV

gi|387515006 -----------------P--GQAMTLK-VEKVLHHEKSKYQDVLIFKSTDYGNVLVLDNV

gi|46137467 -----------------P--GHAMTLR-VEKVLVHEKSKYQDVLIFKSTDFGNVLVLDNV

gi|320583104 -----------------P--GEAMSLK-VNKILYSQRSKYQDVLVFESSNYGNVLVLDGA

gi|350585594 -----------------P--GQALSLQ-VEQLLHHQRSRYQDILVFRSKSYGNVLVLDGV

gi|315053415 -----------------P--GQAMNLR-VNQILHHEKSKYQDVLVFESSDHGTVLVLDNV

gi|322706886 -----------------P--GQAMTLK-VDKVLHHEKSQYQDVLIFKSTDYGTVLVLDNV

gi|402082530 -----------------P--GQAMTLK-VDKVLHHEKSQYQDVLIFKSTDYGNVLVLDNV

gi|317137159 -----------------P--GQAMSLK-VNQILHHEKSKYQDVLVFESSDYGTVLVLDNV

gi|147902014 -----------------P--GQALSLE-VEEVLYHQRSPFQEILVLRSKTYGNVLVLDGL

gi|344283521 -----------------P--GQALSLQ-VEQLLHHQRSQYQDILVFRSKTYGNVLVLDGV

gi|76637216 -----------------P--GQALSLQ-VEQLLHHQRSRYQDILVFRSKSYGNVLVLDGV

gi|255937259 -----------------P--GQAMSLK-VNQIIHHEKSKYQDVLVFESSDYGTVLVLDNV

gi|366998257 -----------------P--GQAMTLK-VEKVLHHEKSKYQDVLIFKSTTYGNVLVLDNV

gi|320588782 -----------------P--GQAMTLR-VKKVLHHEKSKYQDVLVFESTDYGKVLVLDNV

gi|254580735 -----------------P--GQAMTLK-VEKVLHHEKSKYQDVLIFKSTDYGNVLVLDNV

gi|301620173 -----------------P--GQAMSLE-VEEVLYHKQSSFQEILVFRSKTYGNVLVLDGL

gi|258572568 -----------------P--GQAMTLR-VNQILHHEKSKYQDVLVFESSDHGTVLVLDNV

gi|225710012 -----------------D--HRILEYP-VKEVLFSDRSPFQYVQVVDTNDFGKMLILDDM

gi|19112807 -----------------P--GQAMTLK-VKKVLYAGKSKYQDVLVFESETYGHVLVLDGA

gi|398411809 -----------------P--GQAMTLK-VNQVVHHEKSQYQDVLIFESSDHGMVLVLDNV

gi|379728455 --------------------NLALSLRHTGNLLYRKKSEFQTVQVLESHAFGKMLTIDNL

gi|149239971 -----------------P--GQAMSLE-VDKVLHVEKSKYQDVLVFKSKTYGNVLVLDNC

gi|119175541 -----------------P--GQAMTLR-VNQILHHEKSKYQDILIFESTDHGTCLVLDNV

gi|320039089 -----------------P--GQAMTLR-VNQILHHEKSKYQDILIFESTDHGTCLVLDNV

gi|74138635 -----------------P--GQALSLQ-VEQLLHHRRSRYQDILVFRSKTYGNVLVLDGV

gi|238489149 -----------------P--GQAMSLK-VNQILHHEKSKYQDVLVFESSDYGTVLVLDNV

gi|255725052 -----------------P--GQAMSLK-VEKVLHVEKSKYQDVLVFKSTTYGNVLVLDNC

gi|322701141 -----------------P--GQAMTLK-VEKVLHHEKSQYQDVLIFKSTDYGTVLVLDNV

gi|359378073 -----------------P--GQAMSLR-VEKVLHVEKSKYQDVLVFKSTDYGNVLVLDGC

gi|358378881 -----------------P--GQAMTLK-VEKVLHHEKSLYQDVLIFKSTDYGNVLVLDNV

gi|50286095 -----------------P--GQAMTLK-VEKVLHHEKSKYQDVLIFKSTNYGNVLVLDNV

gi|358391288 -----------------P--GQAMTLK-VEKVLHHEKSQYQDVLIFKSTDYGNVLVLDNV

gi|401623242 -----------------P--GQAMTLK-VEKVLHHEKSKFQDVLIFKSTTYGNVLVLDNV

gi|6325326 -----------------P--GQAMTLK-VEKVLHHEKSKYQDVLIFKSTTYGNVLVLDNV

gi|151942852 -----------------P--GQAMTLK-VEKVLHHEKSKYQDVLIFKSTTYGNVLVLDNV

gi|302912624 -----------------P--GQAMTLR-VEKVLVHEKSKYQDVLIFKSTDFGNVLVLDNV

gi|261190484 -----------------P--GQALTLK-VNQILHHEKSKYQDVLVFESSDHGTVLVLDNV

gi|336262713 -----------------P--GQAMTLK-VEKVLHHEKSLYQDVLIFKSTDHGNVLVLDNV

gi|296814308 -----------------P--GQAMNLR-VNQILHHEKSKYQDVLVFESSDHGTVLVLDNV

gi|126138914 -----------------P--GQAMSLK-VEKVLHTEKSKYQDVLVFKSETYGNVLVLDNC

gi|50420767 -----------------P--GQAMSLK-VEKVLHVEKSKYQDVLVFKSTDYGNVLVLDDC

gi|68482416 -----------------P--GQAMSLK-VEKVLHVEKSKYQDVLVFKSTTYGNVLVLDNC

gi|169615146 -----------------P--GQAMTLK-VKEVIHHEKSKYQDVLIFESTDYGMVLVLDNV

gi|396485345 -----------------P--GQAMTLQ-VKEVLHHEKSLYQDILIFESTDYGMVLVLDNV

gi|241951762 -----------------P--GQAMSLK-VEKVLHVEKSKYQDVLVFKSTTYGNVLVLDNC

gi|400597126 -----------------P--GQAMTLK-VEKVLHHEKSKYQDVLVFKSTDYGHVLVLDNV

gi|345561897 -----------------R--GQAMTLK-VEKILHHEKSLYQDVLVFKSTDYGNVLVLDNV

gi|310793583 -----------------P--GQAMTLK-VKKVVHHEKSQYQDVLIFESTDYGMVLVLDNV

gi|340520373 -----------------P--GQAMTLK-VEKVLHHEKSLYQDVLIFKSTDYGNVLVLDNV

gi|47228825 -----------------P--GQAMSLR-VKEVLYNKKSKFQDVLLFASETYGNVLVLDGV

gi|156843587 -----------------P--GQAMTLK-VEKVLHHEKSKYQDVLIFKSTTYGNVLVLDNV

gi|330907629 -----------------P--GQAMTLQ-VKEVLHHEKSKYQDVLIFESTNYGTVLVLDNV

gi|189207669 -----------------P--GQAMTLQ-VKEVLHHEKSKYQDVLIFESTNYGTVLVLDNV

gi|50554613 -----------------P--GQAMALR-VNQILHVEKSKYQDVLVFESTDYGNVLVLDGA

gi|375086054 -----------------P--YVKFSIQ-IDKQLHSEQTEFQRIDIFESKEFGRMMVLDGY

gi|366996717 -----------------P--GQAMTLK-VEKVLHHEKSKYQDVLIFKSTTYGNVLVLDNV

gi|380353232 -----------------P--GQAMSLK-VDKVLHVEKSKYQDVLVFKSETYGNVLVLDNC

gi|154304795 -----------------P--GQAMTLK-VKNVLHHEKSKYQDVLIFESTDYGTVLVLDNV

gi|171693823 -----------------P--GQAMTLK-VEKVLHHEKSKYQDVLIFKSTDYGNVLVLDNV

gi|260781328 -----------------S--EQIVEYD-FDRVVYEENSPYQNIKILHSPQYGNLLLLDDD

gi|344234284 -----------------P--GQAMSLR-VKKVLHAERSKYQDVLVFESTDYGNVLVLDNC

gi|260951089 -----------------P--GSAMSLK-VQEVLYAGKSKYQDVLVFKSTNWGNVLVLDNC

gi|367012209 -----------------P--GQAMTLK-VDKVLHHEKSKYQDVLVFKSTDYGNVLVLDNV

gi|403214093 -----------------P--GEAMTLK-VDKVLHHEKSKYQDVLIFKSTDFGNVLVLDNA

gi|354548134 -----------------P--GQAMSLK-VDKVLHVEKSKYQDVLVFKSETYGNVLVLDNC

gi|372463636 -----------------P--GQAMTLK-VEKVLHHEKSKYQDVLVFKSTDYGNVLVLDNA

gi|284929129 --------------------NVALSIRHKGEHIFYEKSPYQTVEIFDTFEYGKMLTIDKM

gi|291533315 -----------------P--YVKFSIQ-IDKQLHSEQTEFQRIDIFESKEFGRMMVLDGY

gi|384501417 -----------------P--GQAMSIK-VEEILHVEKSKYQDVLVFQSSNYGNVLVLDNV

gi|401838282 -----------------P--GQAMTLK-VEKVLHHEKSKYQDVLIFKSTTYGNVLVLDNV

gi|344302668 -----------------P--GQAMSLQ-VEKVLHVEQSKYQDVLVFKSSTYGNVLVLDGC

gi|365982711 -----------------P--GQAMTLK-VEKVLHHEKSKYQDVLIFKSTTYGNVLVLDNV

gi|296421657 -----------------P--GQAMTLR-VKKVLHHEKSKYQDVLIFESTDYGNVLVLDNV

gi|149695532 -----------------P--GQALSLQ-VEQLLHHQRSRYQDILVFRSKSYGNVLVLDGV

gi|50307673 -----------------P--GQAFSLK-VNKILFHEKTQFQDILIFESSNYGNVLVLDGI

gi|50303991 -----------------P--GQAMTLR-VEKILHHEKSKYQDVLVFKSTDYGNVLVLDNA

gi|380483699 -----------------P--GQAMTLK-VKKVVHHEKSQYQDVLIFESTDYGMVLVLDNV

gi|325660851 -----------------P--HVKFSVR-VDRQLYSGKSEFQRIDVFESPEFGRMLVLDGY

gi|160932658 -----------------P--SVKFSIK-VDRQLYTAQSEFQRIDVFDSKEFGRFLTLDGY

gi|363751094 -----------------P--GQASTLK-VEKVLHHEKSLYQDVLVFKSTNFGNVLVLDNM

gi|254569166 -----------------P--GQAMSLK-VEKILHVEQSLYQDVLIFQSTDYGNVLVLDGA

gi|268612001 -----------------P--NVKFSIK-VDHQLYSGNSEFQRIDVFDSKEFGRFLTLDGY

gi|225018465 -----------------P--HVKFSIK-VDRQLYCGQSEFQRIDVFDSKEFGRFLTLDGY

gi|326427880 -----------------P--GQCMSLE-YEKVLEDIKSDYQHIVVFKSKTYGNVLVLDGV

gi|297617720 -----------------G--RAGLTLK-VKQVIYSGQSPFQRIDILETYEFGRMLVLYGS

gi|295665965 -----------------P--GQAMTLK-VNQILHHEKSKYQDVLVFESSDHGTVLVLDNV

gi|317131721 -----------------D--NVRFSIK-VDRQLYSAQSQFQRIDIFESIEFGRILVLDGY

gi|346467789 -----------------N--NPVLSVD-VEKVLHQERSNFQDILVFKSKSFGTVLALDNA

gi|328766899 -----------------P--GQAMTLQ-VKEILHHEKSLYQDVLVFESTHHGNVLVLDGV

gi|323486581 -----------------P--GVKLSIR-VDRQLYSGKSEFQRIDVFDSPEFGRFLTLDGY

gi|326476682 -----------------P--GQAMNLR-VNQILHHEKSDYQDVLVFESSDHGTVLVLDNV

gi|164660446 -----------------P--GQAMSLR-VRRILHHEKSQYQDVLVFESETYGNVLVLDGA

gi|169351543 -----------------K--GVKFSIK-VDRQLFSGHSEFQRIDIFDSKEFGRFLALDGY

gi|346326304 -----------------P--GQAMTLK-VEKVLHHEKSKYQDVLVFKSTDYGHVLVLDNV

gi|167519370 -----------------P--GQAMSLA-YEEILEDVQSDFQHVQILKTKTYGNALCLDGV

gi|321468222 -----------------P--GQCISLE-VEEVLHQEKSEFQDILVLKSKTYGKVLVLDGV

gi|404329717 -----------------D--HVRFSIQ-VDRQLYSGQSEFQRIDIFDSPEFGRFLTLDGY

gi|283798449 -----------------S--NVKLSIR-VDRQLYSGKSEFQRIDVFDSPEFGRFLTLDGY

gi|300121366 -----------------S--GQRFGLK-VKEVLYHEKSKYQDILVFDSESYGRVLVLDGV

gi|227112962 --------------------NFGQYFS-VDRVLYHEKTDHQDLIIFENDALGRVMALDGV

gi|388582960 -----------------P--GQALSLK-VNKILHVEKSKYQDVLVFESESYGNVLVLDGA

gi|225713606 -----------------D--SRVQEYR-YKEILFSEKSPFQYVQIVDTYDFGKMLILDNM

gi|346466755 -----------------N--NPVLSVD-VEKVLHQERSNFQDILVFKSKSFGTVLALDNA

gi|355720872 ------------------------------------------------------------

gi|167755034 -----------------N--GVNFSIK-VDCQLFSGQSEFQKIDIFDSKEFGRFLALDGY

gi|193215178 --------------------NIALSLRHTGDLLYRKRSPYQRVEIYDTFAYGKMLTIDGL

gi|403059603 --------------------NFGQYFS-VDRVLYHEKTDHQDLIIFENDALGRVMALDGV

gi|388854692 -----------------P--GQAMSLQ-VQRILHHERSLYQDVLVFESTTFGNVLVLDGV

gi|227329706 --------------------NFGQYFS-VDRVLYHEKTDHQDLIIFENDALGRVMALDGV

gi|253689498 --------------------NFGQYFS-VDRVLYHEKTDHQDLIIFENDALGRVMALDGV

gi|300715359 --------------------GFGQYFS-VDKILYREQTGHQDLVIFENAALGRVMALDGV

gi|343427335 -----------------P--GQAMSLE-VQRILHHEKSLFQDVLVFESTTFGNVLVLDGV

gi|1167999 -----------------S--CQALSLQ-VEQLLHHRRSRYQDILVFRSKTYGNVLVLDGV

gi|261369006 -----------------P--DVKHSIR-VDRQLYSKQSDYQRIDIFETPEFGRVLTLDGN

gi|327405610 --------------------NQGLSLRYTGEVFFDVQSPFQRVRILESPKYGKLLTLDDM

gi|254573952 -----------------P--GQAMMLR-VEEILLAEKSEFQDILVFKSTDYGNVLVLDGI

gi|251788647 --------------------NFGQYFS-IEKVLYHEKTDHQDLIIFENAALGRVMALDGV

gi|327309372 -----------------P--GQAMNLR-VNQILHHEKSDYQDVLVFESSDHGTVLVLDNV

gi|313896579 -----------------P--DVRFSIR-VDRQLYSGQSEFQRIDVFESPEFGRFLTLDGY

gi|342871552 -----------------P--GHAMTLR-VEKVLVHEKSEYQDALIFKSTDFGNVLVLDNI

gi|295101693 -----------------P--DVKHSIR-VNKQLYSKQSDYQRIDIFETPEFGRVLTLDGN

gi|302665792 -----------------P--GQAMNLR-VNQILHHEKSDYQDVLVFESSDHGTVLVLDNV

gi|71023471 -----------------P--GQAMSLE-VQRILHHEKSLFQDVLVFESTTFGNVLVLDGV

gi|50122255 --------------------NFGQYFS-VDRVLYHEKTDHQDLIIFENDALGRVMALDGV

gi|320583760 -----------------P--GQAMRLR-VAEILHAEKSKFQDVLVFRSTDFGNVLVLDGI

gi|328351802 -----------------P--GQAMSLK-VEKILHVEQSLYQDVLIFQSTDYGNVLVLDGA

gi|331082383 -----------------N--NVKFSIR-VDKQLYSGQSEFQRIDVFESPEFGKFLTLDGY

gi|320529049 -----------------P--DVRLSIR-VDRQLYSGQSEFQRIDVFESPEFGRFLTLDGY

gi|255658393 -----------------P--NVRFSIK-VDRQLYSGRSEFQRIDIFDSKEFGRFLTLDGY

gi|302500029 -----------------P--GQAMNLR-VNQILHHEKSDYQDVLVFESSDHGTVLVLDNV

gi|307132173 --------------------NFGQYFS-IDQVLYHEKTDHQDLIIFENAALGRVMALDGV

gi|357057706 -----------------P--DVRLSIR-VNRQLYSRQSEFQRIDVFESPEFGRFLTLDGY

gi|271501669 --------------------NFGQYFS-IDHVLYHEKTDHQDLIIFENAALGRVMALDGV

gi|348689589 -----------------P--GQKFSLE-MEEVLFQGKSDFQDVLVFKSATYGNVLVLDGV

gi|387510768 -----------------P--GQAFALK-VEKILHHSRSEFQDILVFKSTTFGNVLVLDGI

gi|260588048 -----------------N--NVKFSIR-VDKQLYSGQSEFQRIDVFESPEFGKFLTLDGY

gi|160943874 -----------------P--DVKHSIR-VNKQLYSKQSDYQRIDIFETPEFGRVLTLDGN

gi|188532978 --------------------GFGQYFS-VDKIIYREKTDHQDLVIFENAALGRVMALDGV

gi|145362282 -----------------P--GEAHSLK-VEKVLFQGKSDYQDVIVFQSATYGKVLVLDGV

gi|394757460 -----------------P--GVKLSIR-VDRQLYSGQSEFQRIDVFESEEFGRFLTLDGY

gi|397614365 -----------------P--GQAFTLQ-VKEVLYHQKSKFQDVLVFDSETHGRVLVLDGV

gi|170025697 --------------------NFGQYFS-VENVLYREKTEHQDLVIFENPELGRVMALDGV

gi|401624651 -----------------P--GQAFTLA-VDSILYHGQSEFQDILIFNNKVYGTVLVLDGI

gi|51595070 --------------------NFGQYFS-VENVLYREKTEHQDLVIFENPELGRVMALDGV

gi|257439175 -----------------P--DVKHSIR-VNKQLYSKQSDYQRIDIFETPEFGRVLTLDGN

gi|238926770 -----------------P--NVRFSIR-VDRQLYSGQSEFQRIDVFESPEFGRFLTLDGY

gi|392423567 -----------------E--NVRFSIK-VDKPLYTGQSEFQRIDIFKSKEFGTIFTLDGL

gi|151941307 -----------------P--GQAFTMT-VDSILYEARSEFQDILIFRNKVYGTVLVLDGI

gi|270263031 --------------------NFGQYFS-VENVLYREKTEHQDLVIFENPVLGRVMALDGV

gi|153813543 -----------------P--DVKHSIR-VNRQLYSRQSEYQRIDIFETPEFGRVLTLDGN

gi|21957501 --------------------NFGQYFS-VENVLFREKTEHQDLVIFENPELGRVMALDGV

gi|325184913 -----------------P--GQRFALQ-MEKVLYRGKSDFQDILVFESSTYGNVLVLDGI

gi|292670864 -----------------P--DVRFSIR-VDHQLYSGQSEFQRIDVFESPEFGRFLTLDGY

gi|108808904 --------------------NFGQYFS-VENVLFREKTEHQDLVIFENPELGRVMALDGV

gi|154623414 -----------------P--GEAHSLK-VEKVLFQGKSDYQDVIVFKSATYGKVLVLDGV

gi|259148132 -----------------P--GQAFTMT-VDSILYEARSEFQDILIFRNKVYGTVLVLDGI

gi|402833951 -----------------K--NAGFSIK-VDRQIYSGRSEFQRIDIFESPEFGRFLTIDGY

gi|242240254 --------------------NFGQYFS-IDQVLYHEKTDHQDLIIFENAALGRVMALDGV

gi|196007934 -----------------P--GQAFTLQ-VDEILYQGRSKYQDIAVFKSKTYGNVLVLDGV

gi|391628126 --------------------NFGQYFS-VENVLFREKTEHQDLVIFENPELGRVMALDGV

gi|323308143 -----------------P--GQAFTMT-VDSILYEARSEFQDILIFRNKVYGTVLVLDGI

gi|377578674 --------------------QFGQYFT-VDNVLYHEKTDHQDLIIFENRAFGRIMALDGV

gi|402570735 -----------------E--NVRFSIK-VDKCLYTDQSEFQRIDVFHSMEFGNFFTLDGL

gi|355378207 -----------------P--DVRFSIR-VDHQLYSGQSEFQRIDVFESPEFGRFLTLDGY

gi|401565378 -----------------P--DVRFSIR-VNRQLYSGQSEFQRIDVFESPEFGRFLTLDGY

gi|6323175 -----------------P--GQAFTMT-VDSILYEARSEFQDILIFRNKVYGTVLVLDGI

gi|354559941 -----------------D--NVRFSIK-VDKPLYTGQSEFQRIDVFNSKEFGNFFTLDGL

gi|359463278 --------------------NIALSIRHRGGWIFREKSPYQTVEVLDTFEYGKMLVIDNM

gi|295108885 -----------------P--DVKHSIR-VNRHLYSRQSEYQRIDIFETPEFGRVLTLDGN

gi|158319435 -----------------E--DVRFSIK-VDNQLYSGQSEFQRIDVFESKEFGKFFTLDGL

gi|334128073 -----------------P--DVRFSIR-VDHQLYSGQSEFQRIDVFESPEFGRFLTLDGY

gi|312162112 -----------------P--GEAHSLK-VEKVLFQGKSKYQNVMVFQSLTYGKVLVLDGV

gi|313114511 -----------------P--DVKHSIR-VNKQLYSKQSDYQRIDIFETPEFGRVLTLDGN

gi|349579866 -----------------P--GQAFTMT-VDSILYEARSEFQDILIFRNKVYGTVLVLDGI

gi|116785514 -----------------P--GEAHSLK-VEKVLFQGKSKYQDVMVFQSLTYGKVLVLDGV

gi|333929035 --------------------NFGQYFS-VENVLYREKTEHQDLVIFENPVLGRVMALDGV

gi|260887129 -----------------K--NAGFSIK-VDRQIYSGRSDFQRIDIFESPEFGRFLTIDGY

gi|386825267 --------------------NFGQYFS-VENVLYREKTEHQDLVIFENPVLGRVMALDGV

gi|37524842 --------------------NFGQYFS-VDKVLYHDKTEHQDLIIFENATLGRIMALDGT

gi|198427208 -----------------P--GQCMSLE-VEEVLFHERSKYQDVLVFKSKTYGNVLVLDGV

gi|291545477 -----------------P--DVKHSIR-VNRQLYSKQSDYQRIDIFETPEFGRVLTLDGN

gi|182418090 -----------------E--NVRFSIK-VEKQLHSEQTEFQRIDILESKEFGRFFTLDGM

gi|259907494 --------------------GFGQYFS-VDKVVYREQTDHQDLIIFENAALGRVMALDGV

gi|291549279 -----------------P--DVKHSIR-VNRQLFSRQSDYQRIDIFETPEFGRVLTLDGN

gi|157372235 --------------------NFGQYFS-VENVLYREKTEHQDLVIFENPVLGRVMALDGV

gi|297845432 -----------------P--GEAHSLK-VEKVLFQGKSDYQDVIVFQSATYGKVLVLDGV

gi|12230014 -----------------P--GEAHSLK-VEKVLFQGKSDYQDVIVFQSATYGKVLVLDGV

gi|170032648 -----------------P--GQCFSVK-IKKVLHEERSKFQDIKVFESESHGTVLVLDGI

gi|198453209 -----------------P--GQAFSLK-VKNVIHKEKSKYQDIQIIETETYGKCLILDGI

gi|284049217 -----------------P--DVKHSLR-VNRHLFSKQSDFQRIDIFETPEFGRVLTLDGN

gi|238754470 --------------------NFGQYFS-VENVLYREKTEHQDLVIFENPVLGRVMALDGV

gi|257126567 -----------------K--NVRFSIK-VDKQLVSVKSDFQRIDIFESQEFGRLLTLDGF

gi|385785731 --------------------GFGQYFS-VDKVVYREQTDHQDLIIFENAALGRVMALDGV

gi|238759889 --------------------NFGQYFS-VENVLYREKTAHQDLVIFENPVLGRVMALDGV

gi|238750530 --------------------NFGQYFS-VENILYREKTEHQDLVIFENPVLGRVMALDGV

gi|6094336 -----------------P--GEAHSLK-VEKILFQGKSDYQNVMVFQSSTYGKVLVLDGV

gi|332160447 --------------------NFGQYFS-VENVLYREKTEHQDLVIFENPVLGRVMALDGV

gi|377851579 --------------------QFGQYFA-VDNVLYHEKTDHQDLIIFENAAFGRVMALDGV

gi|388391237 --------------------QFGQYFA-VDNVLYHEKTDHQDLIIFENAAFGRVMALDGV

gi|91209189 --------------------QFGQYFA-VDNVLYHEKTDHQDLIIFENAAFGRVMALDGV

gi|169837854 -----------------K--NVRFSIK-VDKQLVSVKSDFQRIDIFESQEFGRFLTLDGF

gi|317046947 --------------------GFGQYFT-VEKELYREKTAHQDLVIFENAAFGRVMALDGV

gi|256273016 -----------------P--GQAFTMT-VDSILYEARSEFQDILIFRNKVYGTVLVLDGI

gi|218547577 --------------------QFGQYFA-VDNVLYHEKTDHQDLIIFENAAFGRVMALDGV

gi|374579091 -----------------E--DLKISYR-IKEILYEEQSEFQHVMILDSYSFGRMLVLDGI

gi|373245798 --------------------QFGQYFA-VDNVLYHEKTDHQDLIIFENAAFGRVMALDGV

gi|387605598 --------------------QFGQYFA-VDNVLYHEKTDHQDLIIFENAAFGRVMALDGV

gi|187935464 -----------------E--YVKFSIK-VDRELYTEQSKFQRIDILESKEFGKFFTLDGL

gi|384470785 --------------------QFGQYFA-VDNVLYHEKTDHQDLIIFENAAFGRVMALDGV

gi|329298049 --------------------GFGQYFS-VDNLLYRDKTAHQDLVIFENAAFGRVMALDGV

gi|110803121 -----------------D--KVRFSIK-VDEQLYSGKSDFQRIDVFKSQEFGTFFTLDGL

gi|212639268 -----------------E--NLKISYR-IKEIIYSERSPFQHVMILDSYDFGRMLVLDGV

gi|20807781 -----------------E--NLRFSIK-VKETLVVEKTPYQHLAILDSYQFGRVLVLDGI

gi|253988276 --------------------NFGQYFS-VDKVLYHDKTDHQDLIIFENAELGRIMALDGT

gi|315294649 --------------------QFGQYFA-VDNVLYHEKTDHQDLIIFENAAFGRVMALDGV

gi|383191575 --------------------HFGQYFR-VDKELYREKTDHQDLVIFENAALGRVMALDGV

gi|386228114 --------------------QFGQYFA-VDNVLYHEKTEHQDLIIFENAAFGRVMALDGV

gi|330009512 --------------------HFGQYFS-VDNVLYHEKTDHQDLIIFDNRAFGRVMALDGV

gi|296086541 -----------------P--GEAHSLK-VEKILFQEKSDYQNVMVFQSATYGKVLVLDGV

gi|392297662 -----------------P--GQAFTMT-VDSILYEARSEFQDILIFRNKVYGTVLVLDGI

gi|292489273 --------------------GFGQYFS-VDKVIYRQQTDHQDLIIFENAALGRVMALDGV

gi|225424695 -----------------P--GEAHSLK-VEKILFQEKSDYQNVMVFQSATYGKVLVLDGV

gi|24111564 --------------------QFGQYFA-VDNVLYHEKTDHQDLIIFENAAFGRVMALDGV

gi|194433428 --------------------QFGQYFA-VDNVLYHEKTDHQDLIIFENTAFGRVMALDGV

gi|333978511 -----------------D--HMKLSCR-VKKVLCEEKTPFQHLAVYDTVQFGRMLALDDV

gi|378194398 --------------------QFGQYFA-VDNVLYHEKTDHQDLIIFENAAFGRVMALDGV

gi|238791204 --------------------NFGQYFS-VENVLYREKTEHQDLVIFDNPVLGRVMALDGV

gi|297841771 -----------------P--GEAHSLK-VEKILFQGKSDYQDVIVFQSATYGKVLVLDGV

gi|15799805 --------------------QFGQYFA-VDNVLYHEKTDHQDLIIFENAAFGRVMALDGV

gi|110799842 -----------------D--KVRFSIK-VDEQLYSGKSDFQRIDVFKSQEFGTFFTLDGL

gi|324112468 --------------------QFGQYFA-VDNVLYHEKTDHQDLIIFENAAFGRVMALDGV

gi|260890661 -----------------K--NVRFSIK-IDKQLVSAKSDFQRIDIFESPEFGRFLTLDGF

gi|401840422 -----------------P--GQAFTMA-VDSILYQAQSEFQDILIFRNKVYGTVLVLDGI

gi|312970216 --------------------QFGQYFA-VDNVLYHEKTDHQDLIIFENAAFGRVMALDGV

gi|16128114 --------------------QFGQYFA-VDNVLYHEKTDHQDLIIFENAAFGRVMALDGV

gi|386248282 --------------------QFGQYFA-VDNVLYHEKTDHQDLIIFENAAFGRVMALDGV

gi|366159436 --------------------QFGQYFA-VDNVLYHEKTDHQDLIIFENAAFGRVMALDGV

gi|323975716 --------------------QFGQYFA-VDNVLYHEKTDHQDLIIFENAAFGRVMALDGV

gi|384503184 -----------------P--GEAHSLK-VEKILFQGKSDYQNVMVFQSSSYGKVLVLDGV

gi|18309532 -----------------D--KVRFSIK-VDEQLYSGKSDFQRIDVFKSQEFGTFFTLDGL

gi|372276039 --------------------GFGQYFT-VDKELYREKTAHQDLIIFENAAMGRVMALDGV

gi|333009123 --------------------QFGQYFA-VDNVLYHEKTDHQDLIIFENAAFGRVMALDGV

gi|332097889 --------------------QFGQYFA-VDNVLYHEKTDHQDLIIFENTAFGRVMALDGV

gi|381405644 --------------------GFGQYFT-VDKELYREKTAHQDLIIFENAAMGRVMALDGV

gi|303325156 --------------------QFGQYFA-VDNVLYHEKTDHQDLIIFENAAFGRVMALDGV

gi|377919674 --------------------QFGQYFA-VDNVLYHEKTDHQDLIIFENAAFGRVMALDGV

gi|300905534 --------------------QFGQYFA-VDNVLYHEKTDHQDLIIFENAAFGRVMALDGV

gi|15223115 -----------------P--GEAHSLK-VEKILFQGKSDYQDVIVFQSATYGKVLVLDGV

gi|145336078 -----------------P--GEAHSLK-VEKVLFQGKSDYQDVIVFQSATYGKVLVLDGV

gi|206578746 --------------------HFGQYFS-VDNVLYHEKTDHQDLIIFDNRAFGRVMALDGV

gi|42524959 --------------------NQALSLRYTGDVLFDETNPFQRVRVLDSYSHGKFLAINNM

gi|323157811 --------------------QFGQYFA-VDNVLYHEKTDHQDLIIFENAAFGRVMALDGV

gi|391275496 --------------------QFGQYFA-VDNVLYHEKTDHQDLIIFENAAFGRVMALDGV

gi|195446427 -----------------P--GQSFSLK-VKKVLHKEDSKFQNIQIVETENYGMCLILDGI

gi|251778321 -----------------E--DVKFSIR-VDRELYTEQSKFQRIDILESKEFGRFFTLDGL

gi|152968708 --------------------HFGQYFS-VDNVLYHEKTDHQDLIIFDNRAFGRVMALDGV

gi|371605278 --------------------QFGQYFA-VDNVLYHEKTDHQDLIIFENAAFGRVMALDGV

gi|345395892 --------------------QFGQYFA-VDNVLYHEKTDHQDLIIFENAAFGRVMALDGV

gi|385302583 ---------------------------------MSKKSKYQDVLVFKSTDYGNVLVLDGV

gi|386206487 --------------------QFGQYFA-VDNVLYHEKTDHQDLIIFENAAFGRVMALDGV

gi|238761600 --------------------NFGQYFS-VENVLYREKTEHQDLVIFENPVLGRVMALDGV

gi|194746211 -----------------P--GQSFSLK-VKEVIHKEKSKFQDIQIVETETYGRCLILDGI

gi|331226164 -----------------P--GQAMSLQ-VKKVLHVEKSKFQDVLVFESTSHGNVLVLDGA

gi|340742010 --------------------QFGQYFA-VDNVLYHEKTDHQDLIIFENAAFGRVMALDGV

gi|50552163 -----------------P--GQGLMLK-VEEILHVSQSEFQDVLVFRSTEYGNVLVLDGV

gi|238786761 --------------------NFGQYFS-VENILYREKTEHQDLVIFENPVLGRVMALDGV

gi|386033187 --------------------HFGQYFS-VDNVLYHEKTDHQDLIIFDNRAFGRVMALDGV

gi|6094327 -----------------P--GEAHSLK-VEKILFQGKSDYQNVMVFQSSTYGKVLILDGV

gi|82775410 --------------------QFGQYFA-VDNVLYHEKTDHQDLIIFENAAFGRVMALDGV

gi|110762382 -----------------P--GVSLSLE-VVKVLHRERSQYQDVMVLETKSHGRTLILDGI

gi|333011508 --------------------QFGQYFA-VDNVLYHEKTDHQDLIIFENAAFGRVMALDGV

gi|322834377 --------------------HFGQYFS-VDKELYREKTDHQDLVIFENAALGRVMALDGV

gi|374992883 -----------------D--SVRFSIK-VDKPLYTGKSEFQRIDVFHSKEFGNFFTLDGL

gi|308185706 --------------------GFGQYFT-VDKELYREKTAHQDLIIFENAAMGRVMALDGV

gi|157147448 --------------------QFGQYFA-VDNVLYHEKTDHQDLIIFENAAFGRVMALDGV

gi|12229958 -----------------P--GEAHSLK-VEKILFQGKSDYQDVMVFQSATYGKVLILDGV

gi|33340515 -----------------P--GEAHSLK-VEKILFQGKSDYQNVMVFQSSTYGKVLVLDGV

gi|295098625 --------------------QFGQYFA-VDNVLYHEKTDHQDLIIFENAAFGRVMALDGV

gi|380021423 -----------------P--GVSLSLE-VVKVLHRERSQYQDVMVLETKSHGRTLILDGI

gi|238797200 --------------------NFGQYFS-VENVLYREKTEHQDLVIFENPVLGRVMALDGV

gi|293394516 --------------------SFGQYFS-VEKVLYREKTDHQDLVIFENPVLGRVMALDGV

gi|99083515 -----------------P--GEAHSLK-VEKILFQGKSEYQNVLVFQSSTYGKVLVLDGV

gi|388608380 --------------------QFGQYFA-VDNVLYHEKTDHQDLIIFENAAFGRVMALDGV

gi|320173653 --------------------QFGQYFA-VDNVLYHEKTDHQDLIIFENAAFGRVMALDGV

gi|161612515 --------------------QFGQYFA-VDNVLYHEKTDHQDLIIFENAAFGRVMALDGV

gi|378073834 --------------------QFGQYFA-VDNVLYHEKTDHQDLIIFENAAFGRVMALDGV

gi|161504706 --------------------QFGQYFA-VDNVLYHEKTDHQDLIIFENAAFGRVMALDGV

gi|195499508 -----------------P--GQSFSLK-VKEVIHKEKSKFQDIQIVETETYGRCLILDGI

gi|2821961 -----------------P--GEAHSLK-VEKILFQGKSDYQDVIVFQSATYGKVLVLDGV

gi|359376594 -----------------A--GQAFGLE-VKEILYHVNSEYQDILVFKSATFGNVLVLNGI

gi|374297740 -----------------E--NARFSFK-IDKQVVSLKSEFQRIDVFDSYDFGRVLVLDGF

gi|239827059 -----------------E--NLKISYR-IKDIIFSERSDFQHVMILDSYDFGRMLVLDGV

gi|403383804 -----------------K--DVKFSIK-VDRQLYSGQSEFQRIDVFESSEFGRFLTLDGY

gi|304396607 --------------------GFGQYFT-VDKELYREKTAHQDLIIFENAAMGRVMALDGV

gi|397166809 --------------------HFGQYFA-VDKVLYHEKTDHQDLIIFENAAFGRVMALDGV

gi|123441066 --------------------NFGQYFS-VENVLYREKTEHQDLVIFENPVLGRVMALDGV

gi|82542724 --------------------QFGQYFA-VDNVLYHEKTDHQDLIIFENAAFGRVMALDGV

gi|12229957 -----------------P--GEAHSLK-IEKILFQGKSEYQKVMVFQSSTYGKVLVLDGV

gi|376394468 --------------------HFGQYFS-VDNVLYHEKTDHQDLIIFENAAFGRVMALDGV

gi|218687999 --------------------QFGQYFA-VDNVLYHEKTDHQDLIIFENAAFGRVMALDGV

gi|170768493 --------------------QFGQYFA-VDNVLYHEKTDLQDLIIFENAAFGRVMALDGV

gi|365156921 -----------------N--TLKISYK-LKEILFSEQSPYQHVMIVDSYDFGRMLVLDGV

gi|195330342 -----------------P--GQSFSLK-VKEVIHKEKSRFQDIQIVETETYGRCLILDGI

gi|340356265 -----------------P--HVRFSFR-VEEHLYTATSEFQKIDVFQSTEFGKILTLDGY

gi|388336724 --------------------QFGQYFA-VDNVLYHEKTDHQDLIIFENAAFGRVMALDGV

gi|188589068 -----------------E--DVKFSIR-VDRELYTEQSKFQRIDILESKEFGRFFTLDGL

gi|376400744 --------------------HFGQYFA-VDNVLYHEKTDHQDLIIFENAAFGRVMALDGV

gi|28317069 -----------------P--GQSFSLK-VKEVIHKEKSRFQDIQIVETETYGRCLILDGI

gi|389842198 --------------------QFGQYFA-VDKVLYREKTDHQDLIIFENSAFGRVMALDGV

gi|260596552 --------------------QFGQYFA-VDKVLYREKTDHQDLIIFENSAFGRVMALDGV

gi|6094335 -----------------P--GEAHSLK-VEKILFQGKSDYQNVMVFQSSTYGKVLVLDGV

gi|365988413 -----------------P--GQAFAIK-VSEILHTSQSDFQDILVFQSESYGVVLVLDGI

gi|194446066 --------------------QFGQYFA-VDNVLYHEKTDHQDLIIFENAAFGRVMALDGV

gi|350536257 -----------------P--GEAHSLK-VEKILFQGKSDYQNVLVFQSSTYGKVLVLDGV

gi|357485917 -----------------P--GEAHSLK-VEKILFQGKSDYQNVMVFQSATYGKVLVLDGV

gi|296127808 -----------------E--ESKFSIK-VEKQLYSAQTNFQRIDVFESKEFGKFFTLDGL

gi|74310739 --------------------QFGQYFA-VDNVLYHEKTDHQDLIIFENAAFGRVMALDGV

gi|323166003 --------------------QFGQYFA-VDNVLYHEKTDHQDLIIFENAAFGRVMALDGV

gi|126153731 -----------------P--GEAHSLK-VEKILFQGKSDYQNVMVFQSSTYGKVLVLDGV

gi|291280945 --------------------QFGQYFA-VDNALYHEKTDHQDLIIFENAAFGRVMALDGV

gi|49425361 -----------------P--GEAHSLK-VEKVLFQGKSDYQDVLVFQSSTYGKVLVLDGV

gi|334341918 -----------------P--GLALSVK-VNKVLHREKTDFQDLAIVDTPIFGRMLFLDNI

gi|187731639 --------------------QFGQYFA-VDNVLYHEKTDHQDLIIFENAAFGRVMALDGV

gi|156935354 --------------------QFGQYFA-VDKVLYREKTDHQDLIIFENSAFGRVMALDGV

gi|376377905 --------------------HFGQYFS-VDNVLYHEKTDHQDLIIFENAAFGRVMALDGV

gi|392391837 -----------------E--NVRFSIK-VDRQLYSGQSEFQRIDVFESKEFGTFFTLDGL

gi|331645238 --------------------QFGQYFA-VDNVLYHEKTDHQDLIIFENAAFGHVMALDGV

gi|378578181 --------------------GFGQYFT-VDKELYRDKTAHQDLIIFENAAMGRVMALDGV

gi|24645443 -----------------P--GQSFSLK-VKEVIHKEKSRFQDIQIVETETYGRCLILDGI

gi|335578432 --------------------QFGQYFA-VDNFLYHEKTDHQDLIIFENAAFGRVMALDGV

gi|374383060 -----------------E--NVRFSIK-VDRQLYSNQSEFQRIDVFESREFGTFFTLDGL

gi|358248320 -----------------P--GEAHSLK-VEKILFQGKSDYQNVMVFQSSTYGKVLVLDGV

gi|300919677 --------------------QFGQYFA-VDNVLYHEKTDHQDLIIFENAAFGRVMALDGV

gi|194902914 -----------------P--GQSFSLK-VKEVIHKEKSQFQDIQIVETETYGRCLILDGI

gi|226492509 -----------------P--GEAHSLK-VEKVLFQGKSDYQNVMVFQSSTYGKVLVLDGV

gi|391290786 --------------------QFGQYFA-VDNVLYHEKTDHQDLIIFENAAFGRVMALDGV

**T180H**

**L177E**

**C221R**

lcl|84191 VNLAESDL-AYTRAIMGSGKE---DYTGKDVLILGGGDGGILCEIVKL---KPKMVTMVE

gi|168177377 VNLAESDL-AYTRAIMGSGKE---DYTGKDVLILGGGDGGILCEIVKL---KPKMVTMVE

gi|21264341 VNLAESDL-AYTRAIMGSGKE---DYTGKDVLILGGGDGGILCEIVKL---KPKMVTMVE

gi|791051 VNLAESDL-AYTRAIMGSGKE---DYTGKDVLILGGGDGGILCEIVKL---KPKMVTMVE

gi|332860397 VNLAESDL-AYTRAIMGSGKE---DYTGKDVLILGGGDGGILCEIVKL---KPKMVTMVE

gi|119619392 VNLAESDL-AYTRAIMGSGKE---DYTGKDVLILGGGDGGILCEIVKL---KPKMVTMAD

gi|402909680 VNLAESDL-AYTRAIMGSGKE---DYTGKDVLILGGGDGGILYEIVKL---KPKMVTMVE

gi|332864823 VNLAESDL-AYTRAIMGSGKE---DYTGKDVLILGGGDGGILCEIVKL---KPKMVTMVE

gi|354474372 VNLAESDL-AYTRAIMGSGKE---DYTGKDVLILGCGDGGILCEIVKL---KPKMVTMVE

gi|291407209 VNLAESDL-AYTRAIMGSGKE---DYTGKDVLILGGGDGGILCEIVKL---KPKMVTMVE

gi|297303473 VNLAESDL-AYTRAIMGSGKE---DYTGKDVLILGGGDGGILYEIVKL---KPKMVTMVE

gi|403263885 VNLAESDL-AYTRAIMGSGKE---DYTGKDVLILGGGDGGILCEIVKL---KPKMVTMVE

gi|395753759 VNLAESDL-AYTRAIMGSGKE---DYTGKDVLILGGGDGGILCEIVKL---KPKMVTMVE

gi|355704663 VNLAESDL-AYTRAIMGSGKE---DYTGKDVLILGGGDGGILYEIVKL---KPKMVTMVE

gi|4164136 VNLAESDL-AYTRAIMGSGKE---DYTGKDVLILGGGDGGILCEIVKL---KPKMVTMVE

gi|149030940 VNLAESDL-AYTRAIMGNGKE---DYTGKDVLILGGGDGGILCEIVKL---KPKMVTMVE

gi|76559933 VNLAESDL-AYTRAIMGNGKE---DYTGKDVLILGGGDGGILCEIVKL---KPKMVTMVE

gi|78369282 VNLAESDL-AYTRAIMGSGKE---DYSGKDVLILGGGDGGILCEIVKL---KPKMVTMVE

gi|118600998 VNLAESDL-AYTRAIMGSGKE---DYTGKDVLILGGGDGGILCEIVKL---KPKMVTMVE

gi|345806962 VNLAESDL-AYTQAIMGSGKE---DYSGKDVLILGGGDGGILCEIVKL---KPKMVTMVE

gi|154425706 VNLAESDL-AYTRAIMGSGKE---DYSGKDVLILGGGDGGILCEIVKL---KPKMVTMVE

gi|26340516 VNLAESDL-AYTRAIMGSGKE---HYTGKDVLILGGGDGGILCEIVKL---KPKMVTMVE

gi|384948950 VNLAESDL-AYTRAIMGSGKE---DYTGKDVLILGGGDGGILYEIVKL---KPKMVTMVE

gi|397497639 VNLAESDL-AYTRAIMGSGKE---DYTGKDVLILGGGDGGILCEIVKL---KPKMVTMVE

gi|348561339 VNLAESDL-AYTRAIMGSGKE---DYTGKDVLILGGGDGGILCEIVKL---KPKMVTMVE

gi|354473432 VNLAESDL-AYTRAIMGSGKE---DYTGKDVLILGGGDGGILCETVKL---KPKMVTMVE

gi|344288661 VNLAESDL-AYTQAIMGSGKE---DYTGKDVLILGGGDGGILCEIVKL---KPKMVTMVE

gi|338728955 VNLAESDL-AYTRAIMGSGKE---DYTGKDVLILGGGDGGILCEIVKL---KPKMVTMVE

gi|71895235 VNLAESDL-AYTRAIMGSGKE---DYTGKEVLILGGGDGGILYEIVKL---KPKMVTMVE

gi|297678474 VNLAESDL-AYTRATMGSGKE---DYTGKDVLILGGGDGGILCETVKL---KPKMVTMVE

gi|301756254 VNLAESDL-AYTQAIMGSGKE---DYSGKDVLILGGGDGGILCEIVKL---KPKMVTMVE

gi|296470508 VNLAESDL-AYTRAIMGSGKE---DYSGKDVLILGGGDGGILCEIVKL---KPKMVTMVE

gi|351712477 VNLAESDL-AYTRAIMGSGKE---DYTGKDVLILGGGDGGILCEIVKL---KPKMVTMVE

gi|344242683 VNLAESDL-AYTRAIMGSGKE---DYTGKDVLILGCGDGGILCEIVKL---KPKMVTM--

gi|387018788 VNLAESDL-AYTKAIMGSGKE---DYTNKEVLILGGGDGGILYEIVKM---KPKMVTMVE

gi|327268286 VNLAESDL-AYTQAIMGSGKE---DYADKEVLILGGGDGGILYEIVKL---KPKMVTMVE

gi|334329503 VNLAESDL-AYTQAIMGSGKE---DYTDKEVLILGGGDGGILYEIVKL---KPKMVTMVE

gi|350595577 VNLAESDL-AYTRAIMGSGKE---DYTGKDVLILGGGDGGILCEIVKL---KPKMVTMVE

gi|351706018 VNVAESDL-AYTWAIMDSGKE---EYTGKDLLILGGRNGGKSREIVKL---KPKMATMVE

gi|395518817 VNLAESDL-AYTQAIMGSGKE---DYTDKEVLILGGGDGGILYEIVKL---KPKMVTMVE

gi|149042406 VNLAESDL-AYTRAIMGNGKE---DYTGKDVLILGGGDGGILCEIVKL---KPKMVTMVE

gi|326913546 VNLAESDL-AYTRAIMGSGKE---DYTGKEVLILGGGDGGILYEIVKL---KPKMVTMVE

gi|224042691 VNLAESDL-AYTQAIMGSGKE---DYTGKEVLILGGGDGGILYEIVKL---KPKMVTMVE

gi|148708890 VNLAESDL-AYTRAIMGSGKE---DYTGKDVLILGGGDGGILCEIVKL---KPKMVTMVE

gi|345327044 VNLAESDL-AYTQAIMGSGKE---DYAGKEVLILGGGDGGILYEIVKL---KPKMVTMVE

gi|385862226 VNLAESDL-AYTQAIMGSGKE---DYCGKEVLILGGGDGGILYELVKL---NPKMVTMVE

gi|354482601 VNLAESDS-AYTRAIMGSGKE---DYTGKDVLILGGGDGGILCEIVKL---KPKMVTMVE

gi|281352943 VNLAESDL-AYTQAIMGSGKE---DYSGKDVLILGGGDGGILCEIVKL---KPKMVTMVE

gi|18858203 VNLAESDL-PYTQAIMGSGKE---HYAGKEVLILGGGDGGILHEAVKL---KPKMITMVE

gi|395838143 VNLAESDL-PYTRAIMGSGKE---DYTGKDVLILGGGDGGILCEIVKL---KPKMVTMVE

gi|213511272 VNLAESDL-PYTQAIMGRGKE---NYAGKEVLILGGGDGGILAEAVKL---KPKMITMVE

gi|348518728 VNLAESDS-AYTRAITGSGKE---NYAGKEVLILGGGDGGILAELVKQ---KPKMITMVE

gi|386643030 VNLAESDL-AYTRAIMGSGKE---DYTGKDVLILGGGDGGILCEIVKL---KPKMVTMVE

gi|402909682 VNLAESDL-AYTRAIMGSGKE---DYTGKDVLILGGGDGGILYEIVKL---KPKMVTMVE

gi|4007861 VNLAESDL-AYTKAIMGSGKE---NYAGKEVLILGGGDGGILAEVVKQ---KPKMITMLE

gi|351706975 ---WQSDL-AYTWAIMGSGKE---DYTDKDVLILGGGDGGILCEIVKL---KPKMVTMVE

gi|4007046 INLADSDT-AYTHAIMGSGKE---SYSGKEVLILGGGDGGILAEVVKQ---KPKMITMLE

gi|114687978 VNLAESDL-AYTRAIMGSGKE---DYTGKDVLILGGGDGGILCEIVKL---KPKMVTMVE

gi|2916761 VNLAESDL-AYTRAIMGSGKE---DYTGKDVLILGGGDGGILCEIVKL---KPKMVTMVE

gi|194373711 VNLAESDL-AYTRAIMGSGKE---DYTGKDVLILGGGDGGILCEIVKL---KPKMVTMVE

gi|332860399 VNLAESDL-AYTRAIMGSGKE---DYTGKDVLILGGGDGGILCEIVKL---KPKMVTMVE

gi|125978661 QNIAESDL-IYTETLMGRGIE---NYEGKEICILGGGDGALLYELLKE---NPKHVVMLE

gi|225581217 QNIAESDL-IYTETLMGRGIE---NYEGKEICILGGGDGALLYELLKE---NPKHVVMLE

gi|195376249 QNIAESDL-IYTETLMGRGIE---NYEGKEICILGGGDGALLYELLKE---NPKHVVMLE

gi|195126140 QNIAESDL-IYTETLMGRGIE---NYEGKEICILGGGDGALLYELLKE---KPKHVVMLE

gi|194747133 QNIAESDL-IYTETLMCRGAE---NYEGKEICILGGGDGALLYELLKE---NPKHVVMLE

gi|195012869 QNIAESDL-IYTETLMGRGIE---NYEGKEICILGGGDGALLYELLKE---NPKHVVMLE

gi|24663238 QNIAESDL-IYTETLMCRGVE---NYEGKEICILGGGDGALLYELLKE---NPKHVVMLE

gi|194869781 QNIAESDL-IYTETLMCRGVE---NYEGKEICILGGGDGALLYELLKE---NPKHVVMLE

gi|195327091 QNIAESDL-IYTETLMCRGVE---NYEGKEICILGGGDGALLYELLKE---NPKHVVMLE

gi|390176333 QNIAESDL-IYTETLMGRGIE---NYEGKEICILGGGDGALLYELLKE---NPKHVVMLE

gi|21358269 QNIAESDL-IYTETLMCRGVE---NYEGKEICILGGGDGALLYELLKE---NPKHVVMLE

gi|74211624 VNLAESDL-AYTRAIMGSGKE---DYTGKDVLILGGGDGGILCEIVKL---KPKMVTMVE

gi|195589784 QNIAESDL-IYTETLMCRGVE---NYEGKEICILGGGDGALLYELLKE---NPKHVVMLE

gi|289742457 QNIAESDL-IYTETLMGRGNE---NYEGKEICILGGGDGALLYELLKE---NPKHVVMLE

gi|157118336 QNIAEADL-IYTETLMRRGVE---NYKDKEICILGGGDGALLYELLKE---EPKHVVMLE

gi|158293995 QNIAEADL-IYTETLMCRGKE---DYAGKEICILGGGDGALLYELLKE---GPKMVVMLE

gi|170032109 QNIAEADL-IYTETLMRRGIE---NYKGKEICILGGGDGALLYELLKE---EPKHVVMLE

gi|327290877 VNLAESDL-AYTQAIMGSGKE---DYADKEVLILGGGDGGILYEIVKL---KPKMVTMVE

gi|4927220 VNLAESDL-AYTRAIMGSGKE---DYTGKDVLILGGGDGGILCEIVKL---KPKMVTMVE

gi|195493801 QNIAESDL-IYTETLMCRGVE---NYEGKEICILGGGDGALLYELLKE---NPKHVVMLE

gi|242024750 QNMSESDL-IYTETLMQRGVE---NYENKEIIILGGGDGCLLWELLKE---NPKFITMLE

gi|312372613 -NIAEADL-IYTETLMRRGVE---EYAGKEICILGGGDGALLYELLKE---KPKHVVMLE

gi|119619391 VNLAESDL-AYTRAIMGSGKE---DYTGKDVLILGGGDGGILCEIVKL---KPKMVTMRT

gi|260818384 PNLAESDI-AYTRAILGNGREDF---------------NAFVTILLRS------NLNDAH

gi|340709405 QNISEADL-IYTETLMQRGKE---NYTGKEIVILGGGDGGLLWELLKE---KPKFITMLE

gi|350407163 QNISEADL-IYTETLMQRGKE---NYTGKEIVILGGGDGGLLWELLKE---KPKFVTMLE

gi|383856563 QNISEADL-IYTETLMQRGKE---SYTGKEIVILGGGDGGLLWELLKE---KPKFVTMLE

gi|357613964 QNISEADL-IYTETLMQRGKE---SYEGKEIIILGGGDGALLYELLKE---NPKYVWMLE

gi|156542873 QNMSEADL-IYTETLMQRGKE---NYEGKEIVILGGGDGGLLWELLKE---NPKHVTMLE

gi|332025522 QNMSEADL-IYTETLMQRGKE---NYAGKEIVILGGGDGGLLWELLKE---KPKFVTMLE

gi|110750077 QNISEADL-IYTETLMQRGKE---NYTGKEIVILGGGDGGLLWELLKE---KPKFVTMLE

gi|91085293 QNISEADL-IYTETLMGRGIE---NYKDKEIVILGGGDGALLYELLKE---KPKEVIMLE

gi|307188353 QNMSEADL-IYTETLMQRGKE---NYAGKEIVILGGGDGGLLWELLKE---KPKFVTMLE

gi|390338494 PNLAESDL-AYTQAITGNGRE---SYTGKEVLILGGGDGGILHEVLKE---NPKSIIMVE

gi|195440770 QNIAESDL-IYTETLMGRGIE---NYEGKEICILGGGDGALLYELLKE---NPKHVVMLE

gi|380023489 QNISEADL-IYTETLMQRGKE---NYTGKEIVILGGGDGGLLWELLKE---KPKFVTMLE

gi|156394332 INLAESDL-SYTKAITGNGKE---NYKDKTVLILGGGDGGILHHVLKE---EPKQVIMAE

gi|167538084 PNLAESDL-AYTQAILGSGRF---NFEGKSVLVLGAGDGGVLHQILKL---NPANVIMIE

gi|326435685 VNLGESDI-AYTKAIMGSGSI---DYTGKDVLILGGGDGGILREIVKL---NPKMVTMVE

gi|47227186 INLAESDL-AYTKAIMGSGKE---NYAGKEVLILGGGDGGILAEVGQT---KAK------

gi|321461882 QNLAESDL-VYTETLIQRGKI---DYTGKDVLILGAGDGALLWELLKE---NPNMVTMVE

gi|344241635 ---------------MGSGKED--YT-GKDVLILGGGDGGILCEIVKL---KPKMVTMVE

gi|389613513 QNISEADL-VYTETLMQRGRE---NYEGKEIVILGGGDGALLFELLKE---KPKFVWMLE

gi|313219992 PNLADSDL-AYTKAICGSETF---DFSGKNVFILGGGDGGIINYLRNLSPTKPKYIEMID

gi|313225743 PNLADSDL-AYTKAICGSETF---DFSGKNVLILGGGDGGIINYLRNLSPTKPKYIEMID

gi|198418193 MNLAESDI-SYTVAITGGGRE---DYRGKNVLILGGGDGGILHHLRDK---GARMITMID

gi|391343942 QNLGECDL-PYTHGIMDFGNY---SYTDKDILILGGGDGGLLHELLKE---SPKFVTMID

gi|196014283 PNLAESDI-AYTKAITGNDTQ---DYTDKTVLILGGGDGGILNELLKQ---SPKYVTMV-

gi|307204802 QNMSEADL-IYTETLMQRGKE---NYAGKEIVILGGGDGGLLWELLKE---KPKFITMLE

gi|225718096 PNLAENDTKTYTHNLMALPMGLGPKYKDANILILGGGDGALLKEIFAQPN-KPAMVTMVE

gi|324096424 QNIAESDL-IYTETLMCRGVE---NYEGKEICILGGGDGALLYELLKE---NPKHVVMLE

gi|327352193 IQCTERDEFSYQEMITHLAMNS--HPNPKKVLVIGGGDGGVLREVVKH--ESVEEAVLCD

gi|226293104 IQCTERDEFSYQEMITHLAMNS--HPNPKKVLVIGGGDGGVLREVVKH--ETVEEAILCD

gi|154277382 IQCTERDEFSYQEMITHLAMNS--HPNPKKVLVIGGGDGGVLREVVKH--ETVEEAILCD

gi|212532035 IQCTERDEFSYQEMITHLAMNS--HPNPKKVLVIGGGDGGVLREVVKH--ETVEEAILCD

gi|242117904 IQCTERDEFSYQEMIANLPLCC--HPCPKKVLIIGGGDGGVLREVVKH--PLVESVVQCE

gi|134077286 IQCTERDEFSYQEMITHLAMNS--HPNPKKVLVIGGGDGGVLREVVKH--ETVEEATLCD

gi|348570938 IQCTERDEFSYQEMIANLPLCS--HPNPRKVLIIGGGDGGVLREVVKH--PSVESVVQCE

gi|389628860 IQCTERDEFSYQEMITHLAMNS--HPNPKKVLVIGGGDGGVLREVVKH--DCVEEAILCD

gi|119495240 IQCTERDEFSYQEMITHLAMNS--HPNPKKVLVIGGGDGGVLREVVKH--ESVEEAILCD

gi|67516811 IQCTERDEFSYQEMITHLAMNS--HPNPKKVLVIGGGDGGVLREVVKH--ETVEEAILCD

gi|242774578 IQCTERDEFSYQEMITHLAMNS--HPNPKKVLVIGGGDGGVLREVVKH--ESVEEAILCD

gi|225683737 IQCTERDEFSYQEMITHLAMNS--HPNPKKVLVIGGGDGGVLREVVKH--ETVEEAILCD

gi|255716758 IQVTERDEFSYQEMIAHLGLNS--HPNPKKVLVIGGGDGGVLREIVKH--ESVEEAWLCD

gi|395218408 VMCTQKDEYVYHEMITHVPMFS--NRSAKRALVIGGGDGGTVRELLRH--EQLEEVTLVE

gi|348514634 IQCTERDEFAYQEMIANLPLCS--HPCPKKVLIIGGGDGGVLREVVKH--PLVESAVLCE

gi|347828643 IQCTERDEFSYQEMITHLAMNS--HPNPKKVLVIGGGDGGVLREVVKH--DCVEEAILCD

gi|361131873 IQCTERDEFAYQEMITHLAMNS--HPDPKKVLVIGGGDGGVLREVIKH--DCVEEAILCD

gi|392864754 IQCTERDEFSYQEMITHLAMNS--HPNPKKVLVIGGGDGGVLREIVKH--ESVEEAILCD

gi|115492019 IQCTERDEFSYQEMITHLAMNS--HPDPKKVLVIGGGDGGVLREVVKH--ECVEEAILCD

gi|212532039 IQCTERDEFSYQEMITHLAMNS--HPNPKKVLVIGGGDGGVLREVVKH--ETVEEAILCD

gi|340914898 IQCTERDEFSYQEMITHLAMNS--HPEPKKVLVIGGGDGGVLREVVKH--ECVEEAILCD

gi|156044358 IQCTERDEFSYQEMITHLAMNS--HPNPKKVLVIGGGDGGVLREVVKH--DCVEEAILCD

gi|342887616 IQCTERDEFSYQEMIAHLALNS--HPNPKKVLVIGGGDGGVLREIVKH--DCVEEATLCD

gi|302307177 VQCSERDEFAYQEMITHLAMNL--HANPKKVLVIGGGDGGVLREVLKH--ESVEEAWLCD

gi|367038823 IQCTERDEFSYQEMITHLAMNS--HPNPKKVLVIGGGDGGVLREVLKH--ECVEEAVLCD

gi|358371985 IQCTERDEFSYQEMITHLAMNS--HPNPKKVLVIGGGDGGVLREVVKH--ETVEEATLCD

gi|317030551 IQCTERDEFSYQEMITHLAMNS--HPNPKKVLVIGGGDGGVLREVVKH--ETVEEATLCD

gi|303318439 IQCTERDEFSYQEMITHLAMNS--HPNPKKVLVIGGGDGGVLREIVKH--ESVEEAILCD

gi|85100119 IQCTERDEFSYQEMITHLAMNS--HPNPKKVLVIGGGDGGVLREVVKH--DCVEEAILCD

gi|380088740 IQCTERDEFSYQEMITHLAMNS--HPNPKKVLVIGGGDGGVLREVVKH--DVVEEAILCD

gi|395840954 IQCTERDEFSYQEMIANLPLCS--HPSPRKVLIIGGGDGGVLREVVKH--PSVESVVQCE

gi|16758208 IQCTERDEFSYQEMIANLPLCS--HPNPRKVLIIGGGDGGVLREVVKH--PSVESVVQCE

gi|327287238 IQCTERDEFSYQEMIANLPLCS--HPNPRKVLIIGGGDGGVLREVVKH--PSVESVIQCE

gi|146414243 IQVTERDEFSYQEMITHLALNS--HPNPKKALVIGGGDGGVLREILKH--SSIEEAWLCD

gi|190348490 IQVTERDEFSYQEMITHLALNS--HPNPKKALVIGGGDGGVLREILKH--SSIEEAWLCD

gi|367025809 IQCTERDEFSYQEMITHLAMNS--HPNPKKVLVIGGGDGGVLREVVKH--ECVEEAILCD

gi|121701283 IQCTERDEFSYQEMITHLAMNS--HPNPKKVLVIGGGDGGVLREVVKH--ETVEEAILCD

gi|119592082 IQCTERDEFSYQEMIANLPLCS--HPNPRKVLIIGGGDGGVLREVVKH--PSVESVVQCE

gi|70995928 IQCTERDEFSYQEMITHLAMNS--HPNPKKVLVIGGGDGGVLREVVKH--ESVEEAILCD

gi|6678131 IQCTERDEFSYQEMIANLPLCS--HPNPRKVLIIGGGDGGVLREVVKH--PSVESVVQCE

gi|531202 IQCTERDEFSYQEMIANLPLCS--HPNPRKVLIIGGGDGGVLREVVKH--PSVESVVQCE

gi|114553926 IQCTERDEFSYQEMIANLPLCS--HPNPRKVLIIGGGDGGVLREVVKH--PSVESVVQCE

gi|63253298 IQCTERDEFSYQEMIANLPLCS--HPNPRKVLIIGGGDGGVLREVVKH--PSVESVVQCE

gi|122921244 IQCTERDEFSYQEMIANLPLCS--HPNPRKVLIIGGGDGGVLREVVKH--PSVESVVQCE

gi|116197943 IQCTERDEFSYQEMITHLAMNS--HPNPKKVLVIGGGDGGVLREVVKH--ECVEEATLCD

gi|378733877 IQCTERDEFSYQEMITHLAMNS--HPNPKSVLVIGGGDGGVLREVVKH--ESVEKAILCD

gi|26390475 IQCTERDEFSYQEMIANLPLCS--HPNPRKVLIIGGGDGGVLREVVKH--PSVESVVQCE

gi|402852915 IQCTERDEFSYQEMIANLPLCS--HANPRKVLIIGGGDGGVLREVVKH--PSVESVVQCE

gi|347447556 IQCTERDEFSYQEMIANLPLCS--HPNPRKVLIIGGGDGGVLREVVKH--PSVESVVQCE

gi|383873318 IQCTERDEFSYQEMIANLPLCS--HPNPRKVLIIGGGDGGVLREVVKH--PSVESVVQCE

gi|213409616 IQATERDEFSYQEMIAHLALNS--HANPKKVLVIGGGDGGVLREVVKH--ESVEEVVLCD

gi|32766339 IQCTERDEFSYQEMIANLPLCC--HPCPKKVLIIGGGDGGVLREVVKH--PLVESVVQCE

gi|387515006 IQATERDEFSYQEMITHLGLNS--HPNPKKVLVIGGGDGGVLREVVKH--ESVEEAWICE

gi|46137467 IQCTERDEFSYQEMIAHLALNS--HPNPKKVLVIGGGDGGVLREIVKH--DCVEEATLCD

gi|320583104 IQATERDEFSYQEMIAHLALNS--HPNPKKVLVIGGGDGGVLREVVKH--ECVEEAVLCD

gi|350585594 IQCTERDEFSYQEMIANLPLCS--HPNPRKVLIIGGGDGGVLREVVKH--PAVESVVQCE

gi|315053415 IQCTERDEFSYQEMITHLAMNS--HPNPKKVLVIGGGDGGVLREIVKH--DTVEEAVLCD

gi|322706886 IQATERDEFSYQEMITHLAMNS--HPNPKKVLVIGGGDGGVLREVVKH--DCVEEATLCD

gi|402082530 IQATERDEFAYQEMITHLALNS--HPNPKKVLVIGGGDGGVLREVVKH--DCVEEAILCD

gi|317137159 IQCTERDEFSYQEMITHLAMNS--HPNPEKVLVIGGGDGGVLREVVKH--DTVKKAILCD

gi|147902014 IQCTERDEFSYQEMIANLPLCS--HPNPRKVLIIGGGDGGVLREVVKH--PSVESVVQCE

gi|344283521 IQCTERDEFSYQEMIANLPLCS--HPNPRKVLIIGGGDGGVLREVVKH--PSVEAVVQCE

gi|76637216 IQCTERDEFSYQEMIANLPLCS--HPNPRKVLIIGGGDGGVLREVVKH--SSVEAVVQCE

gi|255937259 IQCTERDEFSYQEMITHLAMNS--HPNPEKVLVIGGGDGGVLREVVKH--ASVKEATLCD

gi|366998257 IQVTERDEFSYQEMITHLAMNS--HPNPKKVLVIGGGDGGVLREVVKH--ESVEEAWLCD

gi|320588782 IQCTERDEFSYQEMITHLAMHS--HPDPKRVLVIGGGDGGVLREVVKH--ECLEEATLCD

gi|254580735 IQATERDEFSYQEMIAHLGLNS--HPEPKKVLVIGGGDGGVLREIVKH--ESVEEAWLCD

gi|301620173 IQCTERDEFSYQEMIANLPLYS--HPNPRKVLIIGGGDGGVLREVVKH--PSVESVVQCE

gi|258572568 IQCTERDEFSYQEMITHLAMNS--HPNPKKVLVIGGGDGGVLREVVKH--ESVEEAVLCD

gi|225710012 PNLAESDTETYTHNLMGLPLGIGPKYKDANILILGGGDGALLKEIFAQPH-TPAMVTMVE

gi|19112807 IQATERDEFSYQEMIAHLALNS--HPNPKKVLVIGGGDGGVLREVVKH--ECVEEAILCD

gi|398411809 IQCTERDEFAYQEMITHLAMFA--HPNPKKVLVIGGGDGGVLREVVKH--ESVEEATLCD

gi|379728455 IMTTEKDEFIYHEMITHPAMLS--HAAPKNILVIGGGDGGTVRELFRH--DSVEKVTMVE

gi|149239971 IQVTERDEFSYQEMITHLAMNS--HPNPKKALVIGGGDGGVLREILKH--SSIEEAWLCD

gi|119175541 IQCTERDEFSYQEMITHLAMNS--HPNPKKVLVIGGGDGGVLREIVKH--ESVEEAILCD

gi|320039089 IQCTERDEFSYQEMITHLAMNS--HPNPKKVLVIGGGDGGVLREIVKH--ESVEEAILCD

gi|74138635 IQCTERDEFSYQEMIANLPLCS--HPNQRKVLIIGGGDGGVLREVVKH--PSVESVVQCE

gi|238489149 IQCTERDEFSYQEMITHLAMNS--HPNPEKVLVIGGGDGGVLREVVKH--DTVKKAILCD

gi|255725052 IQVTERDEFSYQEMITHLALNS--HPNPKKALVIGGGDGGVLREILKH--ESIEEAWLCD

gi|322701141 IQATERDEFSYQEMITHLAMNS--HPNPKKVLVIGGGDGGVLREVVKH--DCVEEATLCD

gi|359378073 IQCTERDEFSYQEMITHLALNS--HPNPKRALVIGGGDGGVLREILKH--SSVEEAWLCD

gi|358378881 VQATERDEFSYQEMITHLAMNS--HPNPKKVLVIGGGDGGVLREVVKH--ECVEEAILCD

gi|50286095 IQATERDEFSYQEMITHLAMNS--HPNPKKVLVIGGGDGGVLREVVKH--ECVEEAWLCD

gi|358391288 IQATERDEFSYQEMITHLAMNS--HPNPKKVLVIGGGDGGVLREVVKH--DCVEEAILCD

gi|401623242 IQATERDEFAYQEMIAHLALNS--HPNPKKVLVIGGGDGGVLREVVKH--ESVEEAWLCD

gi|6325326 IQATERDEFAYQEMIAHLALNS--HPNPKKVLVIGGGDGGVLREVVKH--DSVEEAWLCD

gi|151942852 IQATERDEFAYQEMIAHLALNS--HPNPKKVLVIGGGDGGVLREVVKH--DSVEEAWLCD

gi|302912624 VQCTERDEFSYQEMITHLAMNS--HPNPKKVLVIGGGDGGVLREIVKH--DCVEEAILCD

gi|261190484 IQCTERDEFSYQEMITHLAMNS--HPNPKKVLVIGGGDGGVLREVVKH--ESVEEAVLCD

gi|336262713 IQCTERDEFSYQEMITHLAMNS--HPNPKKVLVIGGGDGGVLREVVKH--DVVEEAILCD

gi|296814308 IQCTERDEFSYQEMITHLAMNS--HPNPKKVLVIGGGDGGVLREIVKH--DTVEEAVLCD

gi|126138914 IQVTERDEFSYQEMITHLALNS--HPNPKKALVIGGGDGGVLREILKH--ESIEEAWLCD

gi|50420767 IQCTERDEFSYQEMIAHLALNS--HPNPKKALVIGGGDGGVLREILKH--ESIEEAWLCD

gi|68482416 IQVTERDEFSYQEMITHLALNS--HPNPKKALVIGGGDGGVLREILKH--ESIEEAWLCD

gi|169615146 IQCTERDEFSYQEMITHLAMNA--HPNPEKVLVIGGGDGGVLREVVKH--DCVKEAVLCD

gi|396485345 IQCTERDEFSYQEMITHLGMNA--HPNPERVLVIGGGDGGVLREIVKH--DCVKEAVLCD

gi|241951762 IQVTERDEFSYQEMITHLALNS--HPNPKKALVIGGGDGGVLREILKH--ESIEEAWLCD

gi|400597126 IQCTERDEFSYQEMIAHLALNS--HPNPKKVLVIGGGDGGVLREVVKH--DCVEEAILCD

gi|345561897 IQCTERDEFSYQEMITHLAMNS--HPEPKKVLVIGGGDGGVLREVIKH--ECVEEAILCD

gi|310793583 IQATERDEFSYQEMITHLAMNS--HPNPKKVLVIGGGDGGVLREVVKH--ECVEEAILCD

gi|340520373 VQATERDEFSYQEMITHLAMNS--HPNPKKVLVIGGGDGGVLREVVKH--DSVEEAILCD

gi|47228825 IQCTERDEFAYQEMIANLPLCS--HPSPKKVLIIGGGDGGVLREVVKH--PLVESVVQCE

gi|156843587 IQATERDEFSYQEMITHLAMNS--HPNPKKVLVIGGGDGGVLREVVKH--ESVEEAWLCD

gi|330907629 IQCTERDEFSYQEMITHLAMNA--HPNPEKVLVIGGGDGGVLREVVKH--DCVKEAVLCD

gi|189207669 IQCTERDEFSYQEMITHLAMNA--HPNPEKVLVIGGGDGGVLREVVKH--DCVKEAVLCD

gi|50554613 IQATERDEYAYQEMIAHLALNS--HPNPKKVLVIGGGDGGVLREIVKH--DCVEEAVLCD

gi|375086054 VMLTEKDEFIYHEMIVHVPMAV--HPNPKRVLIIGGGDGGTARELLRY--KNVESVDLVE

gi|366996717 IQATERDEFSYQEMITHLAMNS--HPNPKKVLVIGGGDGGVLREVVKH--ECVEEAVLCD

gi|380353232 IQVTERDEFSYQEMIAHLALNS--HPNPKKALVIGGGDGGVLREILKH--ESIEEAWLCD

gi|154304795 IQCTERDEFSYQEMITHLAMNS--HPNPKKVLVIGGGDGGVLREVVKH--DCVEEAILCD

gi|171693823 IQATERDEFAYQEMITNLAMMS--HPEPKKVLVIGGGDGGVLREVVKH--DCVEEAILCD

gi|260781328 QNLAESDV-AYTYAMTGNGRED---FTDKDVLILGGGDGGILYHLLQQ---SPKFVTMLE

gi|344234284 IQVTERDEFAYQEMIAHLAINS--HPNPKKALVIGGGDGGVLREILKH--SSIEEAWLCD

gi|260951089 IQVTERDEYSYQEMITHLAMNS--HPNPKKALVIGGGDGGVLREILKH--SSIEEAWLCD

gi|367012209 IQATERDEFSYQEMISHLALNS--HPNPKKVLVIGGGDGGVLREIVKH--ESIEEAWLCD

gi|403214093 IQVTERDEFSYQEMIAHLALNS--HPNPKKVLVIGGGDGGVLREIVKH--ECVEEAVLCD

gi|354548134 IQVTERDEFSYQEMIAHLALNS--HPNPKKALVIGGGDGGVLREILKH--ESIEEAWLCD

gi|372463636 IQVTERDEFSYQEMITHLAMNS--HPNPKKVLVIGGGDGGVLREVVKH--ESVEEAVLCD

gi|284929129 VMCTEKDENAYHEMIVHVPMLL--NPSFSNVLVIGGGDGGSVRELIRH--SHIQKITIVE

gi|291533315 VMLTEKDEFIYHEMIVHVPMAV--HPNPKRVLIIGGGDGGTARELLRY--KNVESVDLVE

gi|384501417 IQATERDEFSYQEMITHLAMNS--HPCPKKVLVIGGGDGGVLREVVKH--ESVEEAILVD

gi|401838282 IQATERDEFAYQEMIAHLALNS--HPSPKKVLVIGGGDGGVLREVVKH--ESVVEAWLCD

gi|344302668 IQVTERDEFSYQEMITHLALNS--HPNPKKALVIGGGDGGVLREILKH--ESIEEAWLCD

gi|365982711 IQATERDEFSYQEMITHLAMNS--HPNPKKVLVIGGGDGGVLREVVKH--ESVEEAVLCD

gi|296421657 IQCTERDEFAYQEMITHLAMNS--HPNPKKVLVIGGGDGGVLREVVKH--EC--------

gi|149695532 IQCTERDEFSYQEMIANLPLCS--HPNPRKVLIIGGGDGGVLREVVKH--SSLESVVQCE

gi|50307673 IQVSEKDEFAYQELISHVPLYS--HENPKKILVIGGGDGGVLREVVKH--SCVEEATLVE

gi|50303991 IQVTERDEFSYQEMIAHLALNS--HPNPKKVLVIGGGDGGVLREIVKH--DSVQEAWLCD

gi|380483699 IQATERDEFSYQEMITHLAMNS--HPNPKKVLVIGGGDGGVLREVVKH--ECVEEAILCD

gi|325660851 IMLTEKDEFIYHEMMTHIPMAV--HPSVKNVLVIGAGDGGIIRELTKY--PEIAHIDMVE

gi|160932658 MMLTEKDEFIYHEMMVHVPMAV--HPNARRVLIIGAGDGGVVRELTRY--HQIEHIDLVE

gi|363751094 LQCTEANEYSYHEMITNLAMNS--HANPKKVLIIGGGDGGVLREVLKH--ESVEEAWLCE

gi|254569166 IQVTERDEFSYQEMIAHLALNS--HPNPRKVLVIGGGDGGVLREVIKH--ECVEKAVLCD

gi|268612001 MMLTEKDEFIYHEMITHVPMAV--HPNPKNILVIGAGDGGVVRELTRY--SSVENIDLVE

gi|225018465 MMLTEKDEFIYHEMIVHVPMAV--HPNPKRVLVIGAGDGGVLRELTRY--STIERIDLVE

gi|326427880 IQITERDECGYQEMITHLPMFC--HPNPERVLIIGGGDGGVLREVLRH--PCVKEVTQCE

gi|297617720 IMITEKDEFVYHEMISHVPLFT--HPNPRRVLVIGGGDGGTVREVLKH--PEVEQVTLVE

gi|295665965 IQCTERDEFSYQEMITHLAMNS--HPNPKKVLVIGGGDGGVLREVVKH--ETVEEAILCD

gi|317131721 IMLTEKDEFIYHEMIAHVPMAV--HPNPRRILVIGGGDGGAVRELTKY--RSVERIDLIE

gi|346467789 IQCTERDEFSYQEMITFLPLNC--HPDPKDVLIIGGGDGGVAREVVKH--PSVVSVTQCE

gi|328766899 IQATERDEFSYQEMIAHLPLNA--HPNPKQVLVIGGGDGGVLREVVKH--DCVEHVTLVE

gi|323486581 MMLTEKDEFIYHEMITHVPLAV--HPAVKQVLVIGAGDGGVIRELTRY--HEIEHIDMVE

gi|326476682 IQCTERDEFSYQEMITHLAMNS--HPNPKKVLVIGGGDGGVLREIVKH--DTVEEAVLCD

gi|164660446 IQCTERDEFAYQEMIAHLPINS--HPNPRRVLVIGGGDGGVLREIVKH--ESVEEAILCD

gi|169351543 MMLTEKDEFIYHEMIVHVPMAV--HPNVKKVLVIGAGDGGVIRELCRY--ETIETIDMVE

gi|346326304 IQCTERDEFSYQEMITHLAMNS--HPNPKKVLVIGGGDGGVLREVVKH--DCVEEAILCD

gi|167519370 IQITERDEFSYQEMMVHVPMFS--HPNPEHVLIIGGGDGGVLREVLKH--PALKSVTQCE

gi|321468222 IQCTELDEFSYQEMISFLPITC--HPNPQKVLIVGGGDGGVARELVKH--PLVQSVVQCE

gi|404329717 MMLTEKDEFIYHEMIVHVPMAV--HPAAKKVLVIGGGDGGAVRELTRY--PGIEQIDLVE

gi|283798449 MMLTEKDEFIYHEMITHVPMAV--HPAVKQVLVIGAGDGGVIRELTRY--PEIEHIDLVE

gi|300121366 IQVTERDEFAYQEMITHLPLFA--HRNPKNVLIVGGGDGGVLREVAKH--PGVERIVMCE

gi|227112962 VQTTERDEFIYHEMLTHVPLLS--HGSAKRVLIIGGGDGGMLREVCRH--RGVEHITMVE

gi|388582960 VQCTERDEFSYQEMIAHLPMAS--HPNPERVLVIGGGDGGVLREVIKH--PQVKEAVLCD

gi|225713606 PNLSEVDTVSYTHNLMGLPLGLGPKYKDANILILGGGDGALLKEIFSQPH-KPAMVTMLE

gi|346466755 IQCTERDEFSYQEMITFLPLNC--HPDPKDVLIIGGGDGGVAREVVKH--PSVVSVTQCE

gi|355720872 ------------------------------------------------------MVTMVE

gi|167755034 MMLTEKDEFIYHEMIVHVPMAV--HPEVKKVLVIGAGDGGVIRELCRY--ETIEKIDMVE

gi|193215178 VMCTEKDEYVYHEMISHVPMLT--HPNPKRVLVIGGGDGGTIREILRH--ESLEEVVMVE

gi|403059603 VQTTERDEFIYHEMLTHVPLLS--HGSAKRVLIIGGGDGGMLREVSRH--RGVEHITMVE

gi|388854692 IQCTERDEFSYQEMIAHLPIAS--HPNPERVLVIGGGDGGVLREVVKH--DSVKEAVLCD

gi|227329706 VQTTERDEFIYHEMLTHVPLLS--HGNAKRVLIIGGGDGGMLREVSRH--RGVEHITMVE

gi|253689498 VQTTERDEFIYHEMLTHVPLLS--HGNAKRVLIIGGGDGGMLREVSRH--HGVEHITMVE

gi|300715359 VQTTERDEFIYHEMMTHVPLLA--HGAAKRVLIIGGGDGAMLREVSRH--KNIEQITMVE

gi|343427335 IQCTERDEFSYQEMIAHLPIAS--HPNPERVLVIGGGDGGVLREVVKH--DSVKEAVLCD

gi|1167999 IQCTEKDEFSYQEMIANLPLCS--HPNPRKVLIIGGGDGGVLREVVKH--PSVESVVQCE

gi|261369006 VMLTERDEFIYDEMITHVPMSV--HRQAKDILVIGAGDGGVVRELTRY--DRVERIDLVE

gi|327405610 FMTTEKDEFHYHEMISHPAMFT--HGNAKNILVIGGGDGGTVREFLRH--EGVEKVTMVE

gi|254573952 IQVTERDEFAYQEMITHLPMFS--HPNPKRVLVIGGGDGGVLREVVKH--PSVESATLVE

gi|251788647 VQTTERDEFIYHEMMTHVPLLA--HGNAKRVLIIGGGDGGMLREVCRH--RNIEQITMVE

gi|327309372 IQCTERDEFSYQEMITHLAMNS--HPNPKKVLVIGGGDGGVLREIVKH--DTVEEAVLCD

gi|313896579 MMLTEKDEFIYHEMIAHVPMCV--RPDAENILVIGGGDGGTVRELLRY--PSVRHIDLVE

gi|342871552 IQCTERDEFSYQEMIAHLALNS--HPNPKKVLVIGGGDGGVLREIVKH--DCVEEATLCD

gi|295101693 VMLTERDEFIYDEMIVHVPMAV--HQEAKDILVIGAGDGGVVRELTRY--DRVERIDLVE

gi|302665792 IQCTERDEFSYQEMITHLAMNS--HPNPKKVLVIGGGDGGVLREIVKH--DTVEEAVLCD

gi|71023471 IQCTERDEFSYQEMIAHLPIAS--HPNPERVLVIGGGDGGVLREVVKH--DSVKEAVLCD

gi|50122255 VQTTERDEFIYHEMLTHVPLLS--HGNAKRVLIIGGGDGGMLREVSRH--HGVEQITMVE

gi|320583760 IQVTERDEFAYQEMITHVPLFA--HPNPKKVLVIGGGDGGVLREVLKH--DCVERATLVE

gi|328351802 IQVTERDEFSYQEMIAHLALNS--HPNPRKVLVIGGGDGGVLREVIKH--ECVEKAVLCD

gi|331082383 MMFTEKDEFIYHEMITHIPMAV--HPHVENILVIGAGDGGVVRELTRY--EEIKRIDLVE

gi|320529049 MMLTEKDEFIYHEMIAHVPMCV--RPDAENILVIGGGDGGTVRELLRY--SSVRHIDLVE

gi|255658393 MMLTEMDEFIYHEMITHVPMCV--NPDAHDILVIGGGDGGTVRELLRY--PTVRHIDLVE

gi|302500029 IQCTERDEFSYQEMITHLAMNS--HPNPKKVLVIGGGDGGVLREIVKH--DTVEEAVLCD

gi|307132173 VQTTERDEFIYHEMMTHVPLLA--HGNAKRVLIIGGGDGGMLREVCRH--RNIEQVTMVE

gi|357057706 MMLTEKDEFIYHEMITHVPMCV--HPEAENILVIGGGDGGTVRELLRY--PSIRHIDLVE

gi|271501669 VQTTERDEFIYHEMMTHVPLLA--HGNAKRVLIIGGGDGGMLREVCRH--RNIEQITMVE

gi|348689589 IQLTERDEFAYQEMITHLPMFA--HKQPKRVLIVGGGDGGVLREVTKH--ACVEQVVMCE

gi|387510768 IQCTERDEFAYQEMIAHVSLYS--HERPKRVLVIGGGDGGVLREVVKH--HSVESVTLVE

gi|260588048 MMFTEKDEFIYHEMITHIPMAV--HPHVENILVIGAGDGGVVRELTRY--EEIKRIDLVE

gi|160943874 VMLTERDEFIYDEMIVHVPMAV--HKEAKDILVIGAGDGGVVRELTRY--DRVERIDLVE

gi|188532978 VQTTERDEFIYHEMMTHVPLLA--HGAPKRVLIIGGGDGAMLREVCRH--KNIEQITMVE

gi|145362282 IQLTERDECAYQEMITHLPLCS--IPNPKKVLVIGGGDGGVLREVARH--ASIEQIDMCE

gi|394757460 MMLTERDEFIYHEMIVHVPMAV--HPKVRRVLVIGGGDGGAVRELIRY--PEIERIDLVE

gi|397614365 IQCTQRDEYSYQEMIAHVPLFS--HPNPERVLVIGGGDGGVLREIARH--PCVKDIVICE

gi|170025697 VQTTERDEFIYHEMMTHVPLLA--HGQAKKVLIIGGGDGAMLREVSRH--KNIEQITMVE

gi|401624651 IQCTEFDEFAYQEMITHIAMFA--HSNPKRVLIIGGGDGGVLREVAKH--SCVEDITMVE

gi|51595070 VQTTERDEFIYHEMMTHVPLLA--HGQAKKVLIIGGGDGAMLREVSRH--KNIEQITMVE

gi|257439175 VMLTERDEFIYDEMIVHVPMAV--HREAKDILVIGAGDGGVVRELTRY--DRVERIDLVE

gi|238926770 MMLTEKDEFIYHEMITHVPMCV--HPEAENILVIGGGDGGTVRELLRY--PSVRHIDLVE

gi|392423567 MMVTEKDEFIYHDMIVHVPMAT--NPAIKNVLVIGAGDGGTVRELTRY--NSIERIDMVE

gi|151941307 VQCTEFDEFAYQEMITHIAMFA--HSNPKRVLIIGGGDGGVLREVAKH--SCVEDITMVE

gi|270263031 VQTTERDEFIYHEMMTHVPLLA--HGQAKKVLIIGGGDGGMLREVSRH--RGVEQITMVE

gi|153813543 VMLTERDEFIYDEMITHVPMSV--HKEAKDILVIGAGDGGVVRELTRY--DRVRHIDLVE

gi|21957501 VQTTERDEFIYHEMMTHVPLLA--HGQAKKVLIIGGGDGAMLREVSRH--KNIEQITMVE

gi|325184913 IQATERDEFAYQEMISHLPLFA--HAEPKNVLIVGGGDGGVLREVVKH--DCVERIVMCE

gi|292670864 MMLTEKDEFIYHEMITHVPMCV--HPEAADILVIGGGDGGTVRELLRY--PSIRHIDLVE

gi|108808904 VQTTERDEFIYHEMMTHVPLLA--HGQAKKVLIIGGGDGAMLREVSRH--KNIEQITMVE

gi|154623414 IQLTERDECAYQEMITHLPLCS--IPNPKKVLVIGGGDGGVLREVARH--ASVEHIDMCE

gi|259148132 VQCTEFDEFAYQEMITHIAMFA--HSNPKRVLIIGGGDGGVLREVAKH--SCVEDITMVE

gi|402833951 MMLTEKDEFIYHEMIVHVPMCV--NPEAKRILVIGGGDGGTVRELLRY--KSVERIDLVE

gi|242240254 VQTTERDEFIYHEMLTHVPLLA--HGDAKRVLIIGGGDGGMLREVCRH--RDIEQITMVE

gi|196007934 IQLTERDEFSYQEMISFLPLNS--HPNPSNVLIIGGGDGGVLREVVKH--PAVKKVTQCE

gi|391628126 VQTTERDEFIYHEMMTHVPLLA--HGQAKKVLIIGGGDGAMLREVSRH--KNIEQITMVE

gi|323308143 VQCTEFDEFAYQEMITHIAMFA--HSNPKRVLIIGGGDGGVLREVAKH--SCVEDITMVE

gi|377578674 VQTTERDEFIYHEMMTHVPLLA--HGNAKHVLIIGGGDGAMLREVSRH--KNVETITMVE

gi|402570735 MMVTEKDEFIYHDMIVHVPMAT--NLNIKNVLVIGAGDGGTVRELTRY--NTIEKIDMVE

gi|355378207 MMLTEKDEFIYHEMITHVPMCV--HPEAADILVIGGGDGGTVRELLRY--PSIRHIDLVE

gi|401565378 MMLTEKDEFIYHEMITHVPMCV--HPEAADILVIGGGDGGTVRELLRY--PSIRHIDLVE

gi|6323175 VQCTEFDEFAYQEMITHIAMFA--HSNPKRVLIIGGGDGGVLREVAKH--SCVEDITMVE

gi|354559941 MMVTQKDEFIYHDMIVHVPMAT--NPKIKNVLVIGAGDGGTVRELTRY--ATIEHIDMVE

gi|359463278 VMCSEGDEKAYHEMIVHVPML---TYSARKVLVIGGGDGGSVREILRH--PQVEEVTLVE

gi|295108885 VMLTERDEFIYDEMITHVPMSV--HKEAKDILVIGAGDGGVVRELTRY--DRVRHIDLVE

gi|158319435 MMVTEKDEFIYHDMITHIPMAT--NPKIKNVLVIGAGDGGTVRELTRY--ETIENIDMVE

gi|334128073 MMLTEKDEFIYHEMITHVPMCV--HPEAKDILVIGGGDGGTVRELLRY--PTIRRIDLVE

gi|312162112 IQLTERDECAYQEMITHLPLCS--VPNPKKVLVIGGGDGGVLREVSRH--MSVEQIDICE

gi|313114511 VMLTERDEFIYDEMIVHVPMAV--HKQAKDILVIGAGDGGVVRELTRY--DRVERIDLVE

gi|349579866 VQCTEFDEFAYQEMITHIAMFA--HSNPKRVLIIGGGDGGVLREVAKH--SCVEDITMVE

gi|116785514 IQLTERDECAYQEMITHLPLCS--VPNPRKVLVIGGGDGGVLREVSRH--MSVEQIDICE

gi|333929035 VQTTERDEFIYHEMMTHVPLLA--HGQAKKVLIIGGGDGGMLREVSRH--QGVEQITMVE

gi|260887129 MMLTEKDEFIYHEMIVHVPMCV--NPEAKRILVIGGGDGGTVRELLRY--KSVERIDLVE

gi|386825267 VQTTERDEFIYHEMMTHVPLLA--HGQVKKVLIIGGGDGGMLREVSRH--RGVEQITMVE

gi|37524842 VQTTERDEFIYHEMMAHVPLFA--HGNAKRVLIIGGGDGGMLREVCRH--QYLESITMVE

gi|198427208 IQCTERDEFAYQEMIAHLPLFS--HPNPKNVLVIGGGDGGVIREVLKH--SSVVSITQCE
[truncated: 527,925 more chars]
